# Photochemical Conversion of Indazoles into Benzimidazoles

**DOI:** 10.1002/anie.202423804

**Published:** 2025-06-10

**Authors:** Thiago dos Santos, Cornelia S. Buettner, Dilara Berna Yildiz, Martina Mamone, Alessandro Ruffoni, Daniele Leonori

**Affiliations:** ^1^ Institute of Organic Chemistry RWTH Aachen University Landoltweg 1 52074 Aachen Germany; ^2^ Department of Chemistry–Faculty of Science Gazi University Teknikokullar Ankara 06500 Turkey

**Keywords:** Benzimidazoles, Chemical permutation, Indazoles, Photochemical rearrangement, Tautomerization

## Abstract

Fragment‐based drug discovery relies on preparing diverse libraries of advanced building blocks, often incorporating heteroaromatic motifs. Altering the core of heteroaromatics to maximize library diversity typically requires de novo synthesis of each system. This can be often challenging when specific substitution patterns are needed. Here, we introduce a photochemical strategy for the direct permutation of 1*H*‐ and 2*H*‐indazoles into benzimidazoles. This transformation exploits the distinct photochemical properties of these heteroaromatics and proceeds under mild conditions. Through systematic experimental and computational studies, we have elucidated a two‐step mechanism involving excited‐state tautomerization of 1*H*‐indazoles, followed by photochemical rearrangement of the resulting 2*H*‐isomers. This approach demonstrates broad substrate scope, high yields, and compatibility with a variety of functional groups. This method can expand the structural diversity of heterocycle‐based libraries through the concept of chemical permutation for heteroaromatic interconversion.

## Graphical abstract

We present a photochemical strategy for transforming 1*H*‐ and 2*H*‐indazoles into benzimidazoles, enabling heteroaromatic interconversion under mild conditions. This approach, grounded in a two‐step mechanism of excited‐state tautomerization and photochemical rearrangement, offers broad scope, high yields, and functional group tolerance, expanding the diversity of heterocycle‐based libraries for fragment‐based drug discovery.

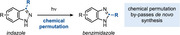

## Introduction

**Scheme 1:**
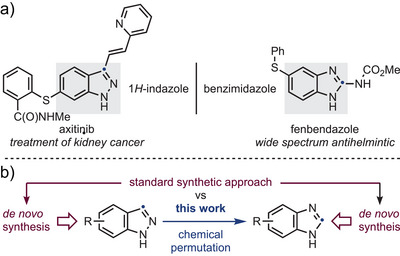
a) Examples of drugs with indazole and benzimidazole systems. b) Chemical permutation as an alternative to de novo synthesis of benzimidazoles.

Among the different strategies in drug discovery aimed at accelerating the hit‐to‐lead process, fragment‐based drug design has emerged as a particularly successful approach.^[^ ^1^, ^2^ ^]^ Central to this strategy are large libraries of advanced building blocks that can be readily diversified. These collections often focus on privileged motifs, with heteroaromatic units being especially prominent (Scheme 1a).^[^ ^3^, ^4^ ^]^ While peripheral modifications of these heteroaromatics are commonly achieved through reactions such as amidation, cross‐coupling, and C─H activation,^[^ ^5^, ^6^ ^]^ altering the core heteroaromatic framework typically requires de novo synthesis.^[^ ^7^ ^]^ This can pose significant synthetic challenges, particularly when specific substitution patterns are needed.

Developing strategies for the direct interconversion of heteroaromatic structures offers significant potential to streamline synthetic efforts. Furthermore, bioisosteric replacements of heterocycles in bioactive molecules provide a powerful means of fine‐tuning pharmacokinetic and pharmacodynamic properties, thereby accelerating the optimization of drug candidates.^[^ ^8^ ^]^

Recent advances have focused on methodologies that enable the “swapping” of individual atoms within heterocyclic rings,^[^ ^9^, ^10^ ^]^ modifying ring size,^[^ ^11^, ^12^ ^]^ or deconstructing and reconstructing rings to access alternative heteroaromatic frameworks.^[^ ^13^ ^]^ One yet underexplored avenue involves repositioning specific atoms within a ring system, a strategy that can be perceived as “chemical permutation”.^[^ ^14^, ^15^ ^]^ This approach would have the potential to repurpose compound libraries by rearranging atoms within heterocycles, offering a novel route to generate diverse heteroaromatic‐based materials. Overall, chemical permutation establishes a new way to access entirely different heterocycles but without adding or removing atoms.

In this manuscript, we present the successful application of permutation chemistry to the direct conversion of indazoles into benzimidazoles (Scheme 1b). This transformation exploits the distinct photochemical properties of these two heterocycles and proceeds under simple photochemical irradiation, without the need for additional reagents. The result is an efficient strategy to expand structural diversity within compound libraries, enabling the creation of diverse heterocycle‐based fragments from structural isomers.

**Scheme 2:**
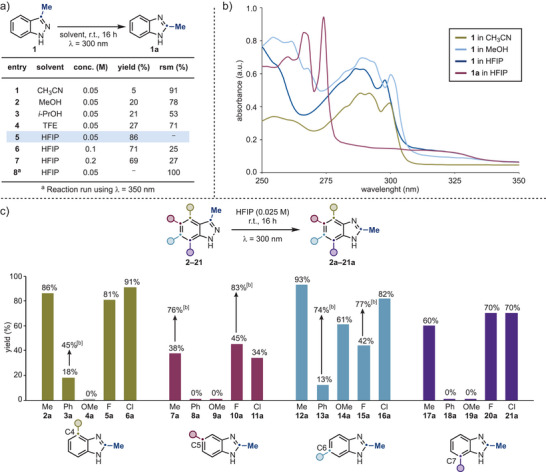
a) Optimization studies for the permutation of **1** into **1a** (NMR Yields, except for entry 5 – Isolated yield). b) UV–vis spectra of **1** and **1a**. c) Investigation of the influence of aromatic substitution patterns with common functional groups on photochemical reactivity (Isolated Yields).^[b]^ Reactions performed at 0.0075 M for 24 h.

## Results and Discussion

### Background and Design Plan

Individual examples of indazoles undergoing photochemical conversion into benzimidazoles have been previously reported in the literature.^[^ ^16^, ^17^, ^18^ ^]^ However, these methods typically required irradiation with high‐energy mercury (Hg) lamps under heating and generally resulted in low yields accompanied by significant decomposition.

Building on our group's recent work on achieving chemical permutation of thiazoles and isothiazoles,^[^ ^19^ ^]^ we sought to explore the photochemical reactivity of indazoles systematically. A key focus of our investigation was to evaluate the influence of various substituents on the photochemical behavior of indazoles. This systematic approach aimed to uncover the scope and limitations of the reactivity, while also providing valuable insights into the mechanistic aspects governing these transformations.

### Initial Reaction Optimization

We initiated our studies by evaluating the conversion of 3‐Me‐indazole **1** into the corresponding 2‐Me‐benzimidazole **1a**. Encouragingly, we found that high yields could be achieved upon irradiation at 300 nm for 16 h at room temperature, provided the appropriate solvent was used (Scheme 2a). Initially, CH_3_CN afforded low conversion (entry 1), but progressively more polar solvents (entries 2–4) led to significantly improved yields, with HFIP giving the best results (entry 5). As we will discuss below, this solvent effect is not due to changes in the absorption profile of **1** but on other mechanistic implications (Scheme 2b). It was essential to perform the reaction under dilute conditions (0.05 M), as higher concentrations led to decreased yields (entries 6 and 7). The necessity of direct photoexcitation of **1** was confirmed by running the reaction at 350 nm, where minimal light absorption occurs, resulting in quantitative mass recovery (entry 8).

### Substituent Effects

With a straightforward protocol for the indazole‐to‐benzimidazole conversion established, we sought to investigate how the aromatic substitution pattern might influence photochemical reactivity. To this end, we prepared a library of 20 indazoles (**2**–**21**) containing common functional groups (Me, Ph, OMe, F, and Cl) at all positions on the fused phenyl ring (C4–C7). Each compound was irradiated at *λ* = 300 nm in HFIP to assess the efficiency of the photo‐rearrangement as a function of the substituent's electronic properties.

This screening revealed remarkable effects based on both the position and nature of the substituents. Substrates with a Me group generally exhibited favorable reactivity, with C4‐ and C6‐substituted derivatives (**2a** and **12a**) yielding higher yields than those substituted at C7 (**17a**) and C5 (**7a**). Interestingly, the presence of a Ph group significantly reduced reactivity: while C4 and C6 derivatives afforded the desired products **3a** and **13a** in low yields, C5 and C7 derivatives (**8a** and **9a**) showed no reaction with quantitative recovery of starting material.

The inclusion of the strongly electron‐donating OMe group also produced notable site‐specific reactivity profiles. Only the C6‐substituted compound (**14a**) demonstrated reactivity, while derivatives substituted at C4, C5, and C7 (**4a**, **9a**, and **19a**) remained unreactive.

Halogenated substrates (F: **5a**, **10a**, **15a** and **20a** and Cl: **6a**, **11a**, **16a** and **21a**) showed strong compatibility with the protocol, achieving moderate to high reactivity. In analogy to Me‐substituted derivatives, C4‐ and C6‐halogenated indazoles demonstrated higher reactivity than those substituted at C5 and C7.

Interestingly, in some cases, **3a**, **7a**, **10a**, **13a** and **15a**, the permutation yield could be significantly improved by further decreasing the reaction concentration to 0.0075 M.

### Substrate Scope

**Scheme 3:**
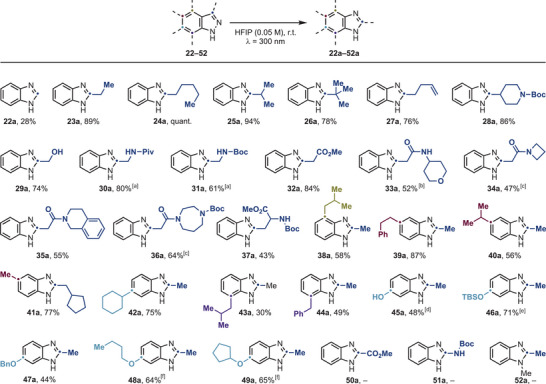
Substrate scope for the photo‐permutation of 1H‐indazoles (Isolated yields). ^[a]^ 24 h reaction time. ^[b]^ 20 h reaction time. ^[c]^ 22 h reaction time. ^[d]^ 0.015 M. ^[e]^ 0.025 M. ^[f]^ 0.0075 M for 24 h.

Based on the findings discussed above (Scheme 3), we expanded our substrate scope using a series of functionalized 1*H*‐indazoles (**22**–**52**) with a high likelihood of success (Scheme 3). While substitution at the C3 position was not essential, an unsubstituted substrate showed lower reactivity (**22a**). High yields were obtained with C3‐alkyl derivatives, covering a range of primary (**23a**, **24a**), secondary (**25a**), and tertiary (**26a**) groups.

We further demonstrated reactivity in the presence of various functional groups, including olefins (**27a**), a C4‐*N*‐Boc‐piperidinyl group (**28a**), free alcohol (**29a**), protected amines (amide: **30a** and carbamate: **31a**), esters (**32a**), secondary and tertiary amides (**33a** and **34a**–**36a**), and amino acid (**37a**).

We then demonstrated high reactivity by placing alkyl substituents at either C4 (**38a**), C5 (**39a**–**41a**), C6 (**42a**) and C7 (**43a** and **44a**). Additionally, we established this chemistry using indazoles with diverse C6‐O substituents, such as free phenol (**45a**), OTBS (**46a**), and ethers (**47a**–**49a**).

Regarding limitations, we were unable to achieve the desired transformation with substrates featuring C3‐ester (**50a**) and C3‐NHBoc (**51a**) functionalities with full recovery of the starting materials. Furthermore, N‐substitution (e.g., **52a**) also led to quantitative starting material recovery with no permutation.

### Mechanistic Studies

**Scheme 4:**
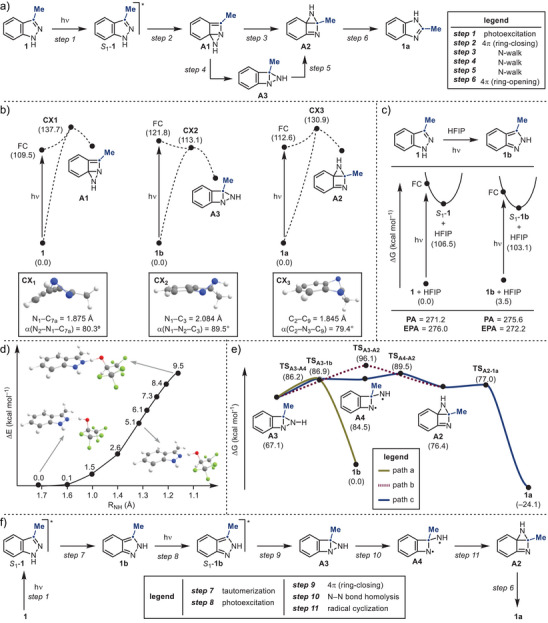
a) Initially proposed mechanism. b) Optimized conical intersection points for **1**, **1b** and **1a** at SA‐CAS(12,10)/cc‐pVDZ level of theory. c) Stability and basicity at ground and excited state for **1 **+ HFIP and **1b **+ HFIP at CAM‐B3LYP/cc‐pVTZ/SMD(HFIP)//cc‐pVDZ(gas) and TDCAM‐B3LYP/cc‐pVTZ/SMD(HFIP)//cc‐pVDZ(gas) level of theory. d) Excited state proton transfer path from HFIP to **1** at TDCAM‐B3LYP/cc‐pVTZ/SMD(HFIP)//cc‐pVDZ(gas) level of theory. e) Computed reaction energy profile for **1b** at UωB97XD/cc‐pVQZ/SMD(HFIP)//def2‐TZVP/SMD(HFIP) level of theory. f) Studied mechanism. Energies and proton affinity and excited state proton affinity are given in kcal mol^−1^.

Initially and based on literature suggestions,^[^ ^17^, ^18^, ^20^ ^]^ we hypothesized that the photochemical permutation of **1** into **1a** could proceed through a mechanism involving photoexcitation (Scheme 4a, step 1), followed by a 4π electrocyclic ring‐closing (step 2) to form the high‐energy Dewar intermediate **A1**. At this stage, an “N‐walk” process could generate the aziridine **A2**, either directly (step 3) or via the intermediate **A3** (steps 4 and 5). Finally, a 4π electrocyclic ring‐opening would yield the desired benzimidazole **1a** (step 6).

To test the feasibility of this pathway, we employed computational methods to evaluate the photochemical reactivity of **1** (Scheme 4b). Upon irradiation, **1** populates its singlet excited state (*S*_1_) with π,π* character. From the Franck–Condon region (FC), the excited‐state molecule can theoretically relax through two conical intersection points (**CX1** and **CX1’**) to generate **A1** and **A1’** (see Supporting Information), respectively. However, our calculations revealed that the energy required to access **CX1** and **CX1’** is significantly higher than that of FC, suggesting that this direct photochemical process is difficult to access. Consequently, our initial mechanistic hypothesis (Scheme 4a) was brought into question.

Previous studies have demonstrated that the *S*_1_ state of 1*H*‐indazoles exhibits increased basicity compared to the ground state (*S*_0_). This has been used to access the corresponding 2*H*‐isomers.^[^ ^21^, ^22^ ^]^ This evidence led us to consider whether the actual permutating species in the indazole‐benzimidazole process might involve a 2*H*‐indazole, formed via an initial photochemical tautomerization. This mechanism aligns with two key experimental observations: (1) the necessity of the weakly acidic HFIP solvent, and (2) the lack of reactivity observed for N‐substituted derivatives (e.g., **52**). In the first case, HFIP could act as a proton donor, facilitating tautomerization, while in the second case, the absence of tautomerization would effectively prevent reactivity.

Our computational studies confirmed that, in the ground state (*S*_0_), **1** is more stable than **1b**, whereas **1b** is more basic than **1** on their respective proton affinities (PAs) (Scheme 4c). Upon photoexcitation, the relative stability reverses, with *S*_1_‐**1b** becoming more stable, while *S*_1_‐**1** becomes more basic, as determined from excited‐state proton affinities (EPAs). These findings suggest that, in the excited state, 1*H*‐indazole undergoes tautomerization to form 2*H*‐indazole. To investigate the proton transfer between *S*_1_‐**1** and HFIP, we performed excited‐state calculations by systematically varying the distance between the N2 atom in **1** and the H‐atom in HFIP (Scheme 4d). These calculations revealed a proton transfer barrier of 9.5 kcal mol^−1^, indicating that tautomerization to **1b** is feasible under photochemical conditions.

Interestingly, once formed, **1b** can undergo further photoexcitation to populate its *S*_1_ π,π* excited‐state. From the FC region, *S*_1_‐**1b** can relax through **CX2** to produce the intermediate **A3**, a process that is now energetically favorable.

The Dewar intermediate **A3** can proceed via multiple pathways (Scheme 4e).^[^ ^20^ ^]^ A retro‐4π electrocyclization to regenerate **1b** (path a) was calculated to have a barrier of 19.8 kcal mol^−1^. Alternatively, an ionic “N‐walk” mechanism (1,3‐suprafacial process) could transform **A3** directly into **A2** (path b), but this pathway was found to be >9 kcal mol^−1^ higher in energy than path a. A more favorable option involves a stepwise process (path c), beginning with N─N bond homolysis to form the bis‐aminyl radical **A4**. This intermediate can now undergo a low‐barrier biradical cyclization to generate **A2**, which can then cyclorevert to **1a** via a nearly barrierless 4π electrocyclic ring‐opening.

A critical question remains: why does the benzimidazole product **1a** exhibit photostability and not revert back to 2*H*‐indazole **1b**? Our excited‐state calculations revealed that, upon photoexcitation to *S*_1_, the energy required to access **CX3** and regenerate the bicyclic **A2** is significantly higher, preventing reversion. We believe this is the ultimate aspect explaining the photostability of the benzimidazole product.

In summary, based on our experimental findings and computational studies, we propose that the 1*H*‐indazole to benzimidazole permutation involves two sequential photochemical processes (Scheme 4f). Initially, photoexcitation of **1** in HFIP induces tautomerization to **1b** (steps 1 and 7). This species is then photoexcited (step 8) to form the high‐energy Dewar intermediate **A3** (step 9). This is likely a reversible process but a competitive N─N bond homolysis can generate **A4** (step 10), en route to the aziridine intermediate **A2** (step 11). Subsequent electrocyclic ring‐opening furnishes the final benzimidazole product **1a** (step 6), which remains photostable under the reaction conditions.

### Permutation of N‐Alkyl 2*H*‐Indazoles: Reaction Development & Substrate Scope

Our proposed mechanistic analysis explains the lack of reactivity observed in *N*‐alkyl 1*H*‐indazoles (e.g., **52**) on the basis of their inability to tautomerize into the reactive 2*H*‐isomers. Interestingly, our computational studies revealed that upon photoexcitation, *N*‐Me‐2*H*‐indazole **53** also features an accessible relaxation pathway to the Dewar intermediate **A5** via the conical intersection **C4**.^[^ ^20^ ^]^ This finding prompted us to investigate whether the permutation of *N*‐alkyl‐2*H*‐indazoles to *N*‐alkyl‐benzimidazoles could be achieved. Successfully realizing this transformation would expand the scope of the process and provide additional evidence supporting the proposed photochemical reactivity discussed above. Also in this case, the resulting N‐Me‐benzimidazole **53a** was predicted to be photostable on the basis of a high‐energy **CX5** leading to the Dewar intermediate **A7** (see SI).

**Scheme 5:**
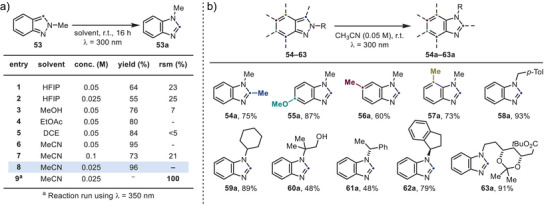
a) Optimization studies for the permutation of **53** into **53a** (NMR Yields, except for entry 8 – Isolated yield). b) Substrate scope for the photo‐permutation of 2*H*‐indazoles (Isolated Yields).

Encouragingly, irradiation of **53** in HFIP yielded **53a** in good yields with high overall mass recovery (entries 1 and 2, Scheme 5a). Notably, because tautomerization is not required for this process to take place, broad solvent compatibility was observed. Indeed, the reaction proceeds efficiently in a variety of solvents, including MeOH (entry 3), EtOAc (entry 4), DCE (entry 5), and CH_3_CN (entries 6–8). Similar to **1**, no photochemical reactivity was observed upon irradiation at *λ* = 350 nm.

We then explored the substrate scope of this transformation with a range of functionalized 2*H*‐indazoles (Scheme 5b). Substrates bearing a C3‐substituent were well‐tolerated (e.g., **54a**), and high reactivity was observed for derivatives featuring C5‐OMe (**55a**), C6‐Me (**56a**), and C7‐Me (**57a**) groups. The N‐substituent scope was successfully expanded to include diverse functional groups, such as a tolyl group (**58a**), a secondary cyclohexyl group (**59a**), and a tertiary group bearing a free alcohol (**60**).

Furthermore, we benchmarked this reactivity using substrates where the N‐substitution forms part of a stereogenic center (e.g., **61a** and **62a**) and observed complete retention of the absolute configuration. Finally, we tested a more complex alkyl chain incorporating both acetal and Boc functionalities (**63a**), which also proved compatible with this photochemical transformation.

## Conclusion

In this study, we have demonstrated a photochemical method for the permutation of 1*H*‐ and 2*H*‐indazoles into benzimidazoles. This transformation leverages the distinct photochemical properties of these heterocycles and proceeds under mild conditions, requiring only irradiation without the need for any additional reagent. Through a combination of experimental and computational studies, we elucidated a mechanism for the reactivity of 1*H*‐indazoles involving two sequential photochemical processes. This highlights the critical role of HFIP as a proton donor for excited‐state tautomerization and the key role of 2*H*‐indazoles as permutating species.

The applicability of this approach was demonstrated across a series of functionalized materials featuring a variety of functional groups, and stereogenic centers. Notably, the process provides high yields and excellent mass recovery, even with complex substrates, expanding the diversity and applicability of heterocycle‐based libraries in fragment‐based drug design.

We believe this work might provide new opportunities for heteroaromatic interconversion with applications in synthetic and medicinal chemistry. We hope that these results will spark interest in the development and application of permutation concepts in organic synthesis.

## Conflict of Interests

The authors declare no conflict of interest.

## Supporting information



Supporting Information

Supporting Information

## Data Availability

The data that support the findings of this study are available in the supplementary material of this article.
