# Supplementary material for: Photochemical Conversion of Indazoles into Benzimidazoles
Source: Angew Chem Int Ed Engl. 2025 Jun 10;64(29):e202423804. doi: 10.1002/anie.202423804 (PMC12258657; doi:10.1002/anie.202423804)
Supplement: Supplementary file 1 — Supporting Information [file ANIE-64-e202423804-s002.pdf]

Photochemical Conversion of Indazoles into Benzimidazoles

Supporting Information

Thiago dos Santos,[a#] Cornelia S. Buettner,[a#] Dilara Berna Yildiz,[a,b] Martina Mamone,[a] Alessandro Ruffoni,[a] and Daniele Leonori*[a]

[a]*Institute of Organic Chemistry, RWTH Aachen University, Landoltweg 1, 52074 Aachen, Germany*

[b]*Department of Chemistry - Faculty of Science, Gazi University Teknikokullar, 06500 Ankara, Turkey*

[#]*These authors contributed equally to this work*

*daniele.leonori@rwth-aachen.de

Table of contents

1. General experimental 2
2. General procedures 3
3. 1*H*-indazole synthesis 7
4. 2*H*-indazole synthesis 24
5. Overview of indazole starting materials 30
6. Reaction optimization for the photo-permutation of 1*H*-indazoles 33
7. Reaction optimization for the photo-permutation of 2*H*-indazoles 34
8. Pictures of reaction setup 35
9. Permutation products – 1*H*-indazoles 36
10. Permutation products – 2*H*-indazoles 50
11. Mechanistic studies 56
12. Computational studies 58
13. NMR Spectra 65
14. References 126

1. General experimental

All required fine chemicals were used directly without purification unless stated otherwise. All air and moisture sensitive reactions were carried out under nitrogen atmosphere using standard Schlenk manifold technique. All solvents were bought from Acros as 99.8% purity and degassed by N_2 bubbling. ^{1}H and ^{13}C Nuclear Magnetic Resonance (NMR) spectra were acquired at various field strengths as indicated and were referenced to $CHCl_3$ (7.27 and 77.16 ppm for ^{1}H and ^{13}C respectively). ^{1}H NMR coupling constants are reported in Hertz and refer to apparent multiplicities and not true coupling constants. The spectra measurements were specified when decoupled (e.g. $^{19}F\{^{1}H\}$), carbon spectra are always decoupled. Data are reported as follows: chemical shift, integration, multiplicity (s = singlet, br s = broad singlet, d = doublet, t = triplet, q = quartet, p = quintet, sx = sextet, sp = septet, m = multiplet, dd = doublet of doublets, etc.), proton assignment (determined by 2D NMR experiments: COSY, HSQC and HMBC) where possible. Due to the proximity of nitrogen and tautomerization of benzimidazole products, some quaternary carbon signals could not be detected by ^{13}C NMR spectroscopy, only distinct signals were reported. High-resolution mass spectra were obtained using a JEOL JMS-700 spectrometer or a Fissions VG Trio 2000 quadrupole mass spectrometer. Spectra were obtained using electron impact ionization (EI) and chemical ionization (CI) techniques, or positive electrospray (ESI). Analytical TLC: aluminium backed plates pre-coated (0.25 mm) with Merck Silica Gel 60 F254. Compounds were visualized by exposure to UV-light or by dipping the plates in permanganate ($KMnO_4$) stain followed by heating. Flash column chromatography was performed using Merck Silica Gel 60 (40–63 µm). All mixed solvent eluents are reported as v/v solutions. Absorption and emission spectra were obtained using an Horiba Duetta spectrometer and 1 mm High Precision Cell made of quartz from Hellma Analytics. Reactions were run in a RPR-200 Rayonet reactor using 300 nm light, with a fan placed above the photoreactor. No stirrer bars were added to the reaction mixtures. All the reactions were conducted in CEM 9 mL glass microwave tubes capped with a LABSOLUTE crimp seal with septum (PTFE/butyl) purchased from Th. Geyer.

2. General procedures

General procedure for disubstituted 1*H*-indazoles from indoles – GP1

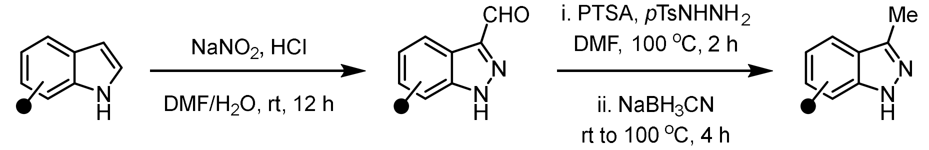

A 2 M HCl solution (2.7 equiv.) was slowly added to a solution of $NaNO_2$ (8 equiv.) in H_2O/DMF 6:4 (1.1 M) at 0 °C, and stirred for 10 minutes. A solution of indole (1 equiv.) in DMF (0.3 M) was then added at 0 °C over a period of 2 h via syringe pump, and the reaction mixture was stirred at room temperature for 12 h. It was then extracted with EtOAc (3×20 mL). The combined organic layers were washed with H_2O (30 mL) and brine (30 mL), dried ($MgSO_4$), filtered, and evaporated. The title compound was isolated by column chromatography using the conditions specified. A mixture of the respective aldehyde (1.0 equiv.), PTSA (0.1 equiv.), *p*TSNHNH$_2$ (1.3 equiv.) in DMF (0.45 M) was stirred at 100 °C for 1.5 h. After cooling to room temperature, $NaBH_3CN$ was added, and the reaction mixture was stirred at room temperature for 0.5 h and then at 100 °C for 3 h. It was then diluted with H_2O (10 mL) and extracted with EtOAc (3×20 mL). The combined organic layers were washed with brine (30 mL), dried ($MgSO_4$), filtered, and evaporated. The title compound was isolated by column chromatography using the conditions specified. Adapted from Cui et al.[1] and Choi et al.[2]

General procedure for the formation of 5-alkyl-3-methyl-1*H*-indazoles – GP2

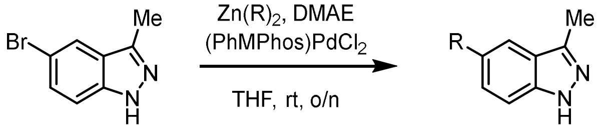

To a solution of 5-bromo-3-methyl-1*H*-indazole (1 eq.) and [1,1’-bis(diphenylphosphino)ferrocene]dichloropalladium complex (0.1 eq.) in dry THF (0.07 M) *N*,*N*-dimethylethanolamine (0.2 eq.) and a dialkyl zinc solution (2 eq.) was added under argon. The yellow solution was stirred for 1 h. Then additional *N*,*N*-dimethylethanolamine (0.2 eq.) and dialkyl zinc solution (2 eq.) were added and the mixture were heated to 60°C. After 16-24 h at 60 °C the solution was quenched with a saturated ammonium chloride solution. The layers were separated, and the aqueous layer was extracted with EtOAc (3×50 mL). The combined organic layers were dried ($MgSO_4$), filtered and evaporated. The crude material was isolated by column chromatography using the conditions specified. Adapted from Hertweck et al.[3]

General procedure for the formation of C3-substituted indazoles – GP3

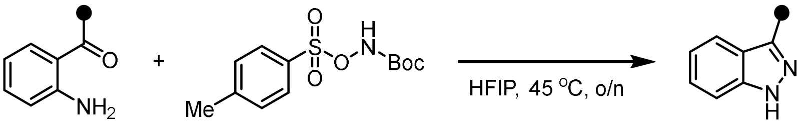

tert-Butyl (tosyloxy)carbamate (1.5 equiv.) and aniline (1 equiv.) were weighed into a microwave vial, the tube was capped with a Supelco aluminium crimp seal with septum (PTFE/butyl), and evacuated and refilled with N_2 (3×). Then HFIP (0.2 M) was added and the sealed vial stirred at 45 °C overnight. The crude reaction mixture was diluted with EtOAc, washed with sat. aq. $NaHCO_3$, dried over $MgSO_4$ and concentrated in vacuo. The title compound was isolated by column chromatography using the conditions specified. Adapted from Lou et al.[4]

General procedure for preparation of arylated 1*H*-indazoles – GP4

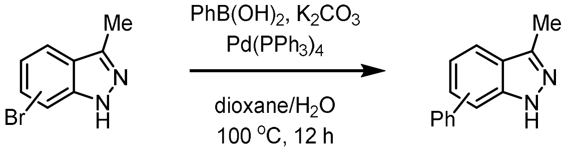

A mixture of the respective halogenated indazole (1.0 equiv.), phenylboronic acid (1.5 equiv.), K_2CO_3 (3.0 equiv.), and $Pd(PPh_3)_4$ (0.05 equiv.) in 1,4-dioxane/H_2O 3:1 (0.14 M) was stirred at 100 °C for 12 hours under argon. The mixture was diluted with water (5 mL), and then extracted with EtOAc (3×10 mL). The combined organic layers were dried ($MgSO_4$), filtered and evaporated. The title compound was isolated by column chromatography using the conditions specified.

General procedure for preparation of 1*H*-indazoles from 2-fluoroacetophenones – GP5

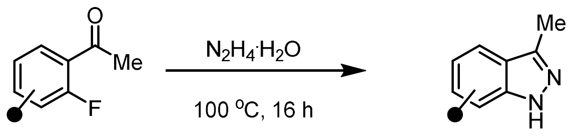

A mixture of the respective acetophenone (1 equiv.) and hydrazine hydrate (42 equiv.) was stirred at 100 °C for 20 h. The reaction mixture was slowly poured to a solution of iced H_2O (20 mL) and extracted with EtOAc (3×20 mL). The combined organic layers were dried ($MgSO_4$), filtered, and evaporated. The title compound was isolated by column chromatography using the conditions specified.

General procedure for amide coupling – GP6

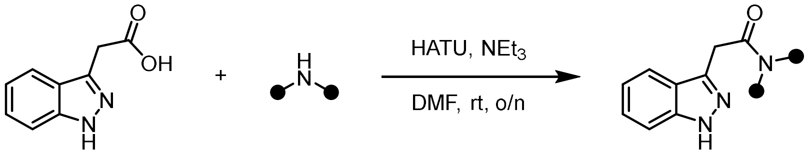

2-(1*H*-indazol-3-yl)-acetic acid (1 equiv.), amine (1.1 equiv.), triethylamine (5 equiv.) and HATU (1.2 equiv.) were stirred in DMF (0.1 M) overnight and at room temperature. The crude reaction mixture was diluted with EtOAc and extracted from brine (3×). The organic layer was dried over $MgSO_4$, concentrated in vacuo and the title compound was isolated by column chromatography using the conditions specified.

General procedure for alkyl substituted 3-methyl-1*H*-indazole – GP7

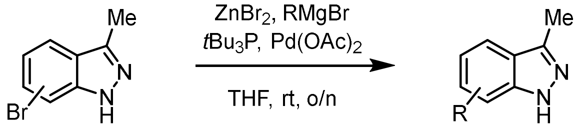

To a solution of bromo-3-methyl-1*H*-indazole (211 mg, 1.0 mmol, 1 eq.) and tri-tert-butylphosphonium tetrafluoroborate (0.12 mmol, 35 mg, 0.12 equiv) in dry THF (2 mL) under N_2 were added $Pd(OAc)_2$ (0.10 mmol, 23 mg, 0.1 equiv) and zinc bromide anhydrous (67 mg, 0.30 mmol, 0.3 equiv). Grignard reagent (2.0 mmol, 2 equiv) was added slowly over 30 min at room temperature. The reaction mixture was stirred for an additional 16-24h and monitored by GCMS. The crude was cooled to 0 °C, and water (10 mL) was added slowly. The layers were separated, and the aqueous layer was extracted with ethyl acetate (3×50 mL). The combined organic layers were dried ($MgSO_4$), filtered and evaporated. The crude material was isolated by column chromatography using the conditions specified. Adapted from Senanayake et al.[5]

General procedure for the etherification of indazoles – GP8

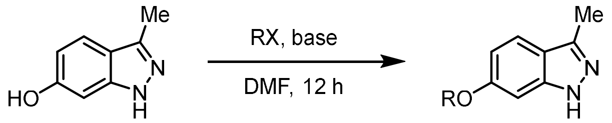

A mixture of 3-methyl-1*H*-indazol-6-ol (1.0 equiv.), alkyl or benzyl halide (1.3 equiv.), and base (1.5 equiv.) in DMF (0.6 M) was stirred at the specified temperature for 12 h. The reaction mixture was diluted with H_2O (10 mL) and extracted with EtOAc (3×10 mL). The combined organic layers were dried ($MgSO_4$), filtered, and evaporated. The title compound was isolated by column chromatography using the conditions specified.

General procedure for the methylation of indazoles – GP9

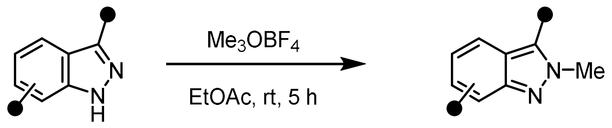

Trimethyloxonium tetrafluoroborate (1.3 equiv.) was added to a solution of 1*H*-indazole (1 equiv.) in EtOAc (0.3 M) and stirred at room temperature for 5 h under nitrogen. Once completed, the reaction mixture was diluted with EtOAc and washed with sat. aq. $NaHCO_3$. The organic layer was dried ($MgSO_4$), filtered, evaporated, and the title compound was isolated by column chromatography using the conditions specified. Adapted from Stafford et al.[6]

General procedure for formation of 2-substituted indazoles from nitroarenes – GP10

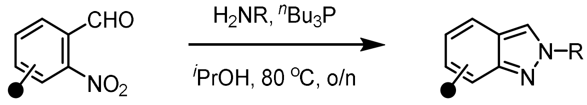

Amine (1.1 equiv) was added to a solution of nitrobenzaldehyde (1 equiv.) in *i*PrOH (0.4 M), and stirred at 80 °C for 4 h. After this time the reaction mixture was cooled to room temperature and *n*Bu_3P (3 equiv.) was added to the reaction, which was heated to 80 °C and stirred overnight. After cooling to room temperature, the reaction was diluted with EtOAc, and washed with sat. aq. NH_4Cl (3×), brine (3×), dried ($MgSO_4$), filtered, and evaporated. The title compound was isolated by column chromatography using the conditions specified. Adapted from Aspnes et al.[7]

General procedure for the photopermutation of indazoles – GP11

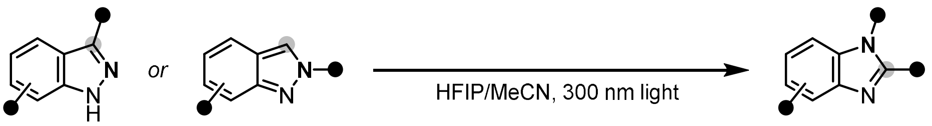

A microwave vial was charged with the indazole (1.0 equiv.) and the tube was capped with a Supelco aluminium crimp seal with septum (PTFE/butyl), evacuated and refilled with N_2 (3×). Then degassed HFIP (0.05 M, 1*H*-indazoles) or CH_3CN (0.025 M, 2*H*-indazoles) was added, the lid sealed with parafilm, and the reaction mixture placed under 300 nm light. After the specified time, the reaction mixture was removed, a solution of 1,3-dinitrobenzene in $CDCl_3$ (1.0 equiv, 0.2 M) was added as an internal standard and the reaction analysed by ^1H NMR. The crude material was isolated by column chromatography using the conditions specified.

3. 1*H*-indazole synthesis

4-Methyl-1*H*-indazole-3-carbaldehyde 2’

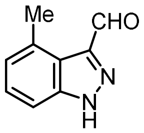

Following **GP1**, 4-methyl-1*H*-indole (394 mg, 3.0 mmol) gave, after purification by column chromatography on silica gel eluting with pentane–EtOAc (4:1), **2’** (43%, 207 mg) as a solid. R_f 0.27 [pentane:EtOAc (4:1)]; ^{1}H NMR (400 MHz, $CDCl_3$) δ 10.28 (1H, s), 7.41 (1H, d, J = 8.4 Hz), 7.37 (1H, dd, J = 8.4, 6.7 Hz), 7.12 (1H, d, J = 6.7 Hz), 2.90 (3H, s); ^{13}C NMR (101 MHz, $CDCl_3$) δ 186.7, 133.8, 128.9, 128.2, 125.2, 121.2, 114.3, 107.6, 22.5; HRMS (EI): Found M^+ 160.0630, $C_9H_8N_2O$ requires 160.0637.

3,4-Dimethyl-1*H*-indazole 2

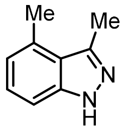

Following **GP1**, 4-methyl-1*H*-indazole-3-carbaldehyde **2’** (160 mg, 1.0 mmol) gave, after purification by column chromatography on silica gel eluting with pentane–EtOAc (3:2), **2** (20%, 29 mg) as a solid. R_f 0.29 [pentane:EtOAc (7:3)]; ^{1}H NMR (600 MHz, $CDCl_3$) δ 7.25 – 7.20 (2H, m), 6.86 (1H, d, J = 5.8 Hz), 2.74 (3H, s), 2.71 (3H, s); ^{13}C NMR (151 MHz, $CDCl_3$) 143.8, 141.9, 132.5, 127.2, 122.1, 121.3, 107.6, 19.8, 15.2; HRMS (ESI): Found MH^+ 147.0913, $C_9H_{10}N_2$ requires 147.0917.

3-Methyl-4-phenyl-1*H*-indazole 3

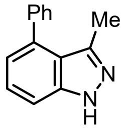

Following **GP4**, 4-bromo-3-methyl-1*H*-indazole (211 mg, 1.0 mmol) gave, after purification by column chromatography on silica gel, eluting with pentane–EtOAc (4:1 to 1:1), **3** (89%, 185 mg) as a solid. R_f 0.56 [cyclohexane:EtOAc (1:1)]; ^{1}H NMR ($CDCl_3$, 600 MHz) δ 7.50 – 7.37 (7H, m), 7.03 (1H, d, J = 6.8 Hz), 2.18 (9H, s); ^{13}CNMR (101 MHz, $CDCl_3$) δ 143.5, 141.9, 140.2, 137.2, 129.6, 128.0, 127.6, 126.7, 121.7, 120.6, 108.9, 15.1; HRMS (EI): Found M^+ 208.0989, $C_{14}H_{12}N_2$ requires 208.0995.

4-Methoxy-3-methyl-1H-indazole 4

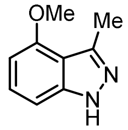

A mixture of 2′,6′-dimethoxyacetophenone (367 mg, 2.0 mmol, 1 eq.), hydrazine monohydrate 98% (200 µL, 2 eq.), polyphosphoric acid (PPA) (4.8 g) and acetic acid (3 drops) was stirred at 125 °C for 0.5 h. After cooling, PPA (4.8 g) was added and the mixture was stirred at 130 °C for 0.5 h. The cooled reaction mixture was poured into ice water (50 mL). The layers were separated, and the aqueous layer was extracted with ethyl acetate (3×40 mL). The combined organic layers were washed with $NaHCO_3$ (20 mL), dried ($MgSO_4$), filtered and evaporated. Purification by flash column chromatography on silica gel eluting with petroleum ether–EtOAc (9:1 to 7:3), gave **4** (50%, 162 mg) as a solid. R_f 0.23 [petroleum ether:EtOAc (4:1)]; ^{1}H NMR (600 MHz, $CDCl_3$) δ 10.28 (1H, br s) 7.28 – 7.18 (1H, m), 6.97 (1H, d, J = 8.3 Hz), 6.42 (1H, d, J = 7.7 Hz), 3.94 (3H, s), 2.69 (3H, s); ^{13}C NMR (151 MHz, $CDCl_3$) δ 155.4, 143.4, 128.4, 114.0, 102.6, 99.3, 55.4, 14.4; HRMS (ESI): Found MH^+ 162.0786, $C_9H_{10}ON_2$ requires 162.0787; Data in accordance with literature.[8]

4-Fluoro-3-methyl-1H-indazole 5

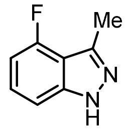

Following **GP3**, 1-(2-amino-6-fluorophenyl)ethan-1-one (107 mg, 0.70 mmol) gave, after purification by column chromatography on silica gel eluting with pentane–EtOAc (9:1 to 7:3), **5** (42%, 44 mg) as a solid. R_f 0.31 [pentane:EtOAc (7:3)]; ^{1}H NMR (600 MHz, $CDCl_3$) δ 10.56 (br s, 1H), 7.38 – 7.31 (m, 1H), 7.25 (d, J = 8.3 Hz, 1H), 6.80 (dd, J = 10.4, 7.6 Hz, 1H), 2.78 (s, 3H); ^{13}C NMR (151 MHz, $CDCl_3$) δ 157.16 (d, J = 251.6 Hz), 144.18 (d, J = 9.0 Hz), 141.76, 128.10 (d, J = 7.6 Hz), 112.95 (d, J = 22.1 Hz), 105.87 (d, J = 4.3 Hz), 104.88 (d, J = 18.9 Hz), 13.89; ^{19}F NMR (564 MHz, $CDCl_3$) δ -121.00; HRMS (ESI): Found MH^+ 151.0662, $C_8H_8N_2F$ requires 151.0666. Data in accordance with literature.[9]

3,5-Dimethyl-1*H*-indazole 7

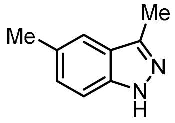

Following **GP2** for 24 h, 5-bromo-3-methyl-1*H*-indazole (424 mg, 2.0 mmol) gave, after purification by column chromatography on silica gel eluting with pentane–EtOAc (9:1 to 3:2), **7** (68%, 199 mg) as a solid. R_f 0.25 [petroleum ether:EtOAc (4:1)]; ^{1}H NMR (600 MHz, $CDCl_3$) δ 7.44 (1H, s), 7.32 (1H, d, J =8.5 Hz), 7.21 (1H, d, J =8.5 Hz), 2.56 (3H, d, J =1.7 Hz), 2.47 (3H, s); ^{13}C NMR (151 MHz, $CDCl_3$) δ 143.1, 139.1, 129.8, 128.9, 123.3, 119.4, 109.4, 21.4, 12.1; HRMS (ESI): Found MH^+ 147.0917, $C_9H_{11}N_2$ requires 147.0917.

3-Methyl-5-phenyl-1*H*-indazole 8

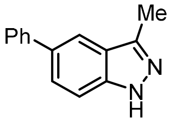

Following **GP4**, 5-bromo-3-methyl-1*H*-indazole (211 mg, 1.0 mmol) gave, after purification by column chromatography on silica gel, eluting with pentane–EtOAc (2:3), **8** (98%, 204 mg) as a solid. R_f 0.44 [pentane:EtOAc (4:6)]; ^{1}H NMR (400 MHz, $CDCl_3$) δ 7.86 (1H, s), 7.68 – 7.63 (3H, m), 7.52 – 7.44 (3H, m), 7.35 (1H, t, J = 7.2 Hz), 2.66 (3H, s); ^{13}C NMR (101 MHz, $CDCl_3$) δ 144.0, 141.7, 140.6, 134.2, 129.0 (2C), 127.5 (2C), 127.2, 127.0, 123.5, 118.6, 110.1, 12.2; HRMS (EI): Found M^+ 208.0991, $C_{14}H_{12}N_2$ requires 208.0995. Data in accordance with literature.[10]

5-Methoxy-3-methyl-1*H*-indazole 9

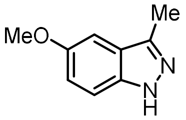

Following **GP3**, 1-(2-amino-5-methoxyphenyl)ethan-1-one (157 mg, 0.95 mmol) gave, after purification by column chromatography on silica gel, eluting with pentane–EtOAc (3:2), **9** (29%, 45 mg) as a solid. R_f 0.40 [pentane: EtOAc (3:2)]; ^{1}H NMR (400 MHz, $CDCl_3$) δ 9.72 (1H, s), 7.32 (1H, d, J = 9.0 Hz), 7.06 (1H, d, J = 9.0 Hz), 7.00 (1H, s), 3.88 (3H, s), 2.56 (3H, s); ^{13}C NMR (101 MHz, $CDCl_3$) δ 154.5, 143.0, 137.1, 123.1, 118.9, 110.8, 99.8, 55.9, 12.2; HRMS (ESI): Found MH^+ 163.0868, $C_9H_{10}N_2O$ requires 163.0866.

5-Fluoro-3-methyl-1*H*-indazole 10

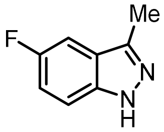

Following **GP3**, 1-(2-amino-5-fluorophenyl)ethan-1-one (107 mg, 0.70 mmol) gave, after purification by column chromatography on silica gel eluting with pentane–EtOAc (9:1 to 7:3), **10** (52%, 55 mg) as a solid. R_f 0.30 [pentane:EtOAc (7:3)]; ^{1}H NMR (600 MHz, $CDCl_3$) δ 10.84 (1H, br s), 7.37 (1H, dd, *J* = 9.0, 4.0 Hz), 7.29 (1H, d, *J* = 8.6 Hz), 7.15 (1H, t, *J* = 8.9 Hz), 2.58 (3H, s); ^{13}C NMR (151 MHz, $CDCl_3$) δ 157.7 (d, *J* = 237.9 Hz), 143.3 (d, *J* = 5.7 Hz), 138.2, 122.9 (d, *J* = 9.7 Hz), 116.5 (d, *J* = 27.5 Hz), 111.0 (d, *J* = 9.5 Hz), 104.4 (d, *J* = 23.1 Hz), 12.1; ^{19}F NMR (565 MHz, $CDCl_3$) δ -123.38; HRMS (ESI): Found MH^+ 151.0662, $C_8H_8N_2F$ requires 151.0666. Data in accordance with literature.[4]

3,6-Dimethyl-1*H*-indazole 12

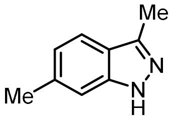

Following **GP3**, 1-(2-amino-4-methylphenyl)ethanone (60 mg, 0.40 mmol) gave, after purification by column chromatography on silica gel, eluting with pentane–EtOAc (4:1 to 1:1), **12** (99%, 58 mg) as a solid. R_f 0.53 [cyclohexane:EtOAc (1:1)]; ^{1}H NMR ($CDCl_3$, 600 MHz) δ 7.55 (1H, d, *J* = 8.2 Hz), 7.20 (1H, s), 6.98 (1H, d, *J* = 8.2 Hz), 2.57 (3H, s), 2.48 (3H, s); ^{13}C NMR (151 MHz, $CDCl_3$) δ 143.5, 141.9, 137.3, 122.7, 121.1, 119.9, 109.3, 22.1, 12.2; HRMS (ESI): Found MH^+ 147.0916, $C_9H_{11}N_2$ requires 147.0917. Data in accordance with literature.[4]

3-Methyl-6-phenyl-1*H*-indazole 13

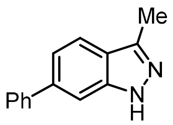

Following **GP4**, 6-bromo-3-methyl-1*H*-indazole (211 mg, 1.0 mmol) gave, after purification by column chromatography on silica gel, eluting with pentane–EtOAc (2:3), **13** (94%, 196 mg) as a solid. R_f 0.52 [pentane:EtOAc (4:6)]; ^{1}H NMR (600 MHz, DMSO-d_6) δ 12.68 (1H, s), 7.76 (1H, d, *J* = 8.3 Hz), 7.71 (2H, d, *J* = 7.6 Hz), 7.63 (1H, s), 7.48 (2H, t, *J* = 7.6 Hz), 7.39 – 7.35 (m, 2H), 2.50 (3H, s); ^{13}C NMR (151 MHz, DMSO-d_6) δ 141.5, 141.0, 140.8, 138.3, 128.9

(2C), 127.3, 127.1 (2C), 121.5, 120.4, 119.2, 107.5, 11.7; HRMS (ESI): Found MH^+ 209.1073, $C_{14}H_{13}N_2$ requires 209.1073.

6-Chloro-3-methyl-1*H*-indazole 16

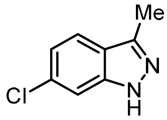

Following **GP5**, 1-(4-chloro-2-fluorophenyl)ethan-1-one (0.41 mL, 3.0 mmol) gave, after purification by column chromatography on silica gel eluting with pentane–EtOAc (4:1), **16** (67%, 167 mg) as a solid. R_f 0.25 [pentane:EtOAc (4:1)]; ^{1}H NMR (600 MHz, $CDCl_3$) δ 10.26 (1H, s), 7.58 (1H, d, J = 8.5 Hz), 7.42 (1H, s), 7.11 (1H, d, J = 8.5 Hz), 2.58 (3H, s); ^{13}C NMR (151 MHz, $CDCl_3$) δ 143.8, 141.6, 133.3, 121.6, 121.5, 121.4, 109.6, 12.1; HRMS (EI): Found M^+ 166.0290, $C_8H_7ClN_2$ requires 166.0298.

7-Methyl-1*H*-indazole-3-carbaldehyde 17'

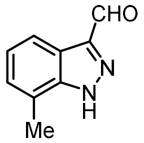

Following **GP1**, 7-methyl-1*H*-indole (394 mg, 3.0 mmol) gave, after purification by column chromatography on silica gel eluting with pentane–EtOAc (4:1), **17'** (72%, 346 mg) as a solid. R_f 0.29 [pentane:EtOAc (4:1)]; ^{1}H NMR (400 MHz, $CDCl_3$) δ 10.88 (1H, br s), 10.31 (1H, s), 8.18 – 8.13 (1H, m), 7.32 – 7.24 (2H, m), 2.62 (3H, s); ^{13}C NMR (101 MHz, $CDCl_3$) δ 187.7, 145.3, 141.5, 128.2, 124.7, 120.9, 120.1, 119.6, 16.8; HRMS (EI): Found M^+ 160.0629, $C_9H_8N_2O$ requires 160.0637. Data in accordance with literature.[11]

3,7-Dimethyl-1*H*-indazole 17

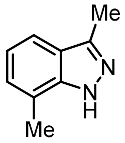

Following **GP1**, 7-methyl-1*H*-indazole-3-carbaldehyde **17'** (240 mg, 1.5 mmol) gave, after purification by column chromatography on silica gel eluting with pentane–EtOAc (3:2), **17** (55%, 121 mg) as a solid. R_f 0.32 [pentane:EtOAc (3:2)]; ^{1}H NMR (400 MHz, $CDCl_3$) δ 7.52 (1H, d, J = 8.0 Hz), 7.15 (1H, d, J = 7.0 Hz), 7.07 (1H, t, J = 7.0 Hz), 2.60 (3H, s), 2.53 (3H, s); ^{13}C NMR (101 MHz, $CDCl_3$) δ 144.1, 141.4, 126.9, 122.5, 120.8, 119.7, 117.8, 16.9, 12.3;

HRMS (ESI): Found MH^+ 147.0915, $C_9H_{11}N_2$ requires 147.0917. Data in accordance with literature.[12]

3-Methyl-7-phenyl-1*H*-indazole 18

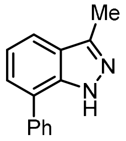

Following **GP4**, 7-bromo-3-methyl-1*H*-indazole (211 mg, 1.0 mmol) gave, after purification by column chromatography on silica gel, eluting with pentane–EtOAc (2:3), **18** (94%, 196 mg) as a solid. R_f 0.48 [pentane:EtOAc (2:3)]; ^{1}H NMR (600 MHz, $CDCl_3$) δ 10.17 (1H, s), 7.69 – 7.64 (3H, m), 7.53 (2H, t, *J* = 7.0 Hz), 7.42 (1H, t, *J* = 7.1 Hz), 7.42 (1H, d, *J* = 7.2 Hz), 7.25 (1H, t, *J* = 7.2 Hz), 2.63 (3H, s); ^{13}C NMR (151 MHz, $CDCl_3$) δ 144.1, 139.5, 138.3, 129.5 (2C), 128.0 (3C), 126.1, 124.6, 123.5, 121.1, 119.5, 12.3; HRMS (ESI): Found MH^+ 209.1071, $C_{14}H_{13}N_2$ requires 209.1073.

7-Methoxy-3-methyl-1*H*-indazole 19

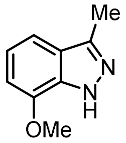

Following **GP3**, 1-(2-amino-3-methoxyphenyl)ethan-1-one (83 mg, 0.50 mmol) gave, after purification by column chromatography on silica gel, eluting with pentane–EtOAc (4:1 to 1:1), **19** (78%, 63 mg) as a solid. R_f 0.59 [cyclohexane:EtOAc (1:1)]; ^{1}H NMR ($CDCl_3$, 600 MHz) δ 7.25 (1H, d, *J* = 7.8 Hz), 7.06 (1H, t, *J* = 7.8 Hz), 6.74 (1H, d, *J* = 7.8 Hz), 3.98 (3H, s), 2.59 (3H, s); ^{13}C NMR (101 MHz, $CDCl_3$) δ 145.3, 143.5, 133.2, 124.4, 120.9, 112.4, 105.1, 55.5, 12.2; HRMS (EI): Found M^+ 162.0786, $C_9H_{10}N_2O$ requires 162.0788.

7-Fluoro-3-methyl-1*H*-indazole 20

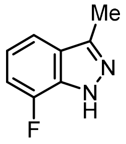

Following **GP5**, 1-(2,3-difluorophenyl)ethan-1-one (0.39 mL, 3.0 mmol) gave, after purification by column chromatography on silica gel eluting with pentane–EtOAc (4:1), **20** (314 mg, 70%, 315 mg) as a solid. R_f 0.36 [pentane:EtOAc (4:1)]; ^{1}H NMR (600 MHz, $CDCl_3$)

δ 10.22 (1H, s), 7.47 – 7.42 (1H, m), 7.09 – 7.03 (2H, m), 2.61 (3H, s); ^{13}C NMR (151 MHz, $CDCl_3$) δ 148.2 (J = 246.9 Hz), 144.2, 130.9 (J = 15.1 Hz), 126.8 (J = 4.5 Hz), 120.9 (J = 5.2 Hz), 116.1 (J = 4.2 Hz), 111.1 (J = 15.9 Hz), 12.2; ^{19}F NMR (565 MHz, $CDCl_3$) δ -132.68; HRMS (EI): Found M^+ 150.0585, $C_8H_7FN_2$ requires 150.0593.

3-Ethyl-1*H*-indazole 23

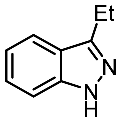

Following **GP3**, 1-(2-aminophenyl)propan-1-one (75 mg, 0.50 mmol) gave, after purification by column chromatography on silica gel, eluting with pentane–EtOAc (4:1 to 7:3), **23** (75%, 55 mg) as a solid. R_f 0.36 [cyclohexane:EtOAc (4:1)]; ^{1}H NMR ($CDCl_3$, 600 MHz) δ 7.73 (1H, d, J = 7.9 Hz), 7.44 (1H, d, J = 7.9 Hz), 7.38 (1H, t, J = 7.9 Hz), 7.15 (1H, t, J = 7.9 Hz), 3.04 (2H, q, J = 7.6 Hz), 1.43 (3H, t, J = 7.6 Hz); ^{13}C NMR (151 MHz, $CDCl_3$) δ 148.8, 141.4, 126.8, 122.1, 120.4, 120.2, 109.9, 20.6, 13.5; HRMS (EI): Found M^+ 146.0837, $C_9H_{10}N_2$ requires 146.0844. Data in accordance with literature.[4]

3-Pentyl-1*H*-indazole 24

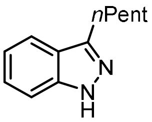

Following **GP3**, 1-(2-aminophenyl)hexan-1-one (96 mg, 0.50 mmol) gave, after purification by column chromatography on silica gel, eluting with pentane–EtOAc (9:1 to 4:1), **24** (61%, 57 mg) as an oil. R_f 0.42 [cyclohexane:EtOAc (4:1)]; ^{1}H NMR ($CDCl_3$, 600 MHz) δ 9.88 (1H, br s), 7.72 (1H, d, J = 8.1 Hz), 7.43 (1H, d, J = 8.1 Hz), 7.37 (1H, t, J = 7.6 Hz), 7.14 (1H, t, J = 7.6 Hz), 2.99 (2H, t, J = 7.8 Hz), 1.84 (2H, p, J = 7.6 Hz), 1.44 – 1.34 (4H, m), 0.90 (3H, t, J = 7.1 Hz); ^{13}C NMR (151 MHz, $CDCl_3$) δ 147.8, 141.3, 126.7, 122.4, 120.5, 120.2, 109.9, 31.9, 29.0, 27.2, 22.6, 14.1; HRMS (EI): Found M^+ 188.1311, $C_{12}H_{16}N_2$ requires 188.1308.

3-Isopropyl-1*H*-indazole 25

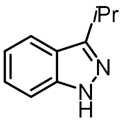

Following **GP3**, 1-(2-aminophenyl)-2-methylpropan-1-one (163 mg, 1.0 mmol) gave, after purification by column chromatography on silica gel, eluting with pentane–EtOAc (19:1 to

4:1), **25** (87%, 139 mg) as a solid. R*f* 0.39 [cyclohexane:EtOAc (4:1)]; ^{1}H NMR ($CDCl_3$, 600 MHz) δ 7.78 (1H, d, *J* = 8.0 Hz), 7.44 (1H, d, *J* = 8.0 Hz), 7.37 (1H, t, *J* = 8.0 Hz), 7.14 (1H, t, *J* = 8.0 Hz), 3.44 (1H, p, *J* = 6.9 Hz), 1.48 (6H, d, *J* = 6.9 Hz); ^{13}C NMR (101 MHz, $CDCl_3$) δ 152.3, 141.6, 126.6, 121.1, 120.7, 119.9, 110.1, 27.9, 22.3; HRMS (ESI): Found MH^+ 161.1069, $C_{10}H_{13}N_2$ requires 161.1073. Data in accordance with literature.[4]

3-(*tert*-Butyl)-1*H*-indazole 26

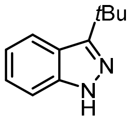

Following **GP3**, 1-(2-aminophenyl)-2,2-dimethylpropan-1-one (41 mg, 0.23 mmol) gave, after purification by column chromatography on silica gel, eluting with pentane–EtOAc (19:1 to 4:1), **26** (55% 22 mg) as a solid. R*f* 0.34 [cyclohexane:EtOAc (4:1)]; ^{1}H NMR ($CDCl_3$, 400 MHz) δ 9.87 (1H, br s), 7.91 (1H, d, *J* = 8.2 Hz), 7.44 (1H, d, *J* = 8.2 Hz), 7.35 (1H, ddd, *J* = 8.2, 6.9, 0.8 Hz), 7.15 – 7.10 (1H, m), 1.55 (9H, s); ^{13}C NMR (151 MHz, $CDCl_3$) δ 154.9, 142.1, 126.4, 122.3, 120.7, 120.0, 110.0, 33.9, 30.1; HRMS (ESI): Found MH^+ 175.1226, $C_{11}H_{15}N_2$ requires 175.1230. Data in accordance with literature.[13]

3-(But-3-en-1-yl)-1*H*-indazole 27

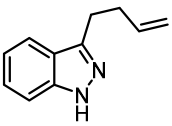

Following **GP3**, 1-(2-aminophenyl)pent-4-en-1-one (60 mg, 0.34 mmol) gave, after purification by column chromatography on silica gel, eluting with pentane–EtOAc (19:1 to 4:1), **27** (34%, 20 mg) as an oil. R*f* 0.31 [cyclohexane:EtOAc (4:1)]; ^{1}H NMR ($CDCl_3$, 600 MHz) δ 7.72 (1H, d, *J* = 7.8 Hz), 7.44 (1H, d, *J* = 7.8 Hz), 7.38 (1H, t, *J* = 7.8 Hz), 7.15 (1H, t, *J* = 7.8 Hz), 5.95 (1H, ddt, *J* = 17.0, 10.2, 6.7 Hz), 5.10 (1H, dd, *J* = 17.0, 1.7 Hz), 5.01 (1H, dd, *J* = 10.2, 1.7 Hz), 3.10 (2H, t, *J* = 7.8 Hz), 2.61 (2H, dt, *J* = 7.8, 6.7 Hz); ^{13}C NMR (101 MHz, $CDCl_3$) δ 147.0, 141.3, 138.1, 126.9, 122.4, 120.4, 120.4, 115.3, 109.8, 33.1, 26.8; HRMS (ESI): Found MH^+ 173.1077, $C_{11}H_{13}N_2$ requires 173.1073. Data in accordance with literature.[4]

N-((1*H*-Indazol-3-yl)methyl)pivalamide 30

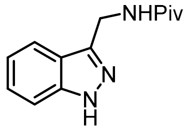

Triethylamine (0.11 mL, 0.75 mmol) and pivaloyl chloride (62 μL, 0.50 mmol) were added to a solution of 3-(aminomethyl)-1*H*-indazole (74 mg, 0.50 mmol) in CH_2Cl_2 (5 mL) and stirred at room temperature for 4 h. The reaction mixture was concentrated and gave, after purification by column chromatography on silica gel, eluting with pentane–EtOAc (3:2 to 1:4), **30** (72%, 83 mg) as a solid. R_f 0.32 [cyclohexane:EtOAc (1:1)]; ^{1}H NMR ($CDCl_3$, 400 MHz) δ 11.33 (1H, br s), 7.73 (1H, d, J = 7.9 Hz), 7.46 (1H, d, J = 7.9 Hz), 7.35 (1H, t, J = 7.9 Hz), 7.12 (1H, t, J = 7.9 Hz), 6.51 (1H, br s), 4.86 (2H, d, J = 5.1 Hz), 1.23 (9H, s); ^{13}C NMR (101 MHz, $CDCl_3$) δ 178.8, 143.2, 141.6, 127.1, 121.3, 120.9, 120.2, 110.2, 38.9, 36.8, 27.7; HRMS (EI): Found M^+ 231.1367, $C_{13}H_{17}N_3O$ requires 231.1366.

***tert*-Butyl ((1*H*-indazol-3-yl)methyl)carbamate 31**

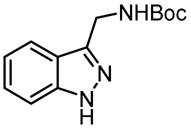

Triethylamine (0.11 mL, 0.75 mmol) and boc anhydride (47 μL, 0.50 mmol) were added to a solution of 3-(aminomethyl)-1*H*-indazole (74 mg, 0.50 mmol) in CH_2Cl_2 (5 mL) and stirred at room temperature for 4 h. The reaction mixture was concentrated and gave, after purification by column chromatography on silica gel, eluting with pentane–EtOAc (3:2 to 1:4), **31** (49%, 61 mg) as an oil. R_f 0.50 [cyclohexane:EtOAc (1:1)]; ^{1}H NMR ($CDCl_3$, 400 MHz) δ 7.77 (1H, d, J = 7.8 Hz), 7.41 (1H, d, J = 7.8 Hz), 7.34 (1H, t, J = 7.8 Hz), 7.12 (1H, t, J = 7.8 Hz), 5.44 (1H, br s), 4.71 (2H, d, J = 5.2 Hz), 1.47 (9H, s); ^{13}C NMR (101 MHz, $CDCl_3$) δ 156.2, 143.9, 141.4, 127.1, 121.3, 120.8, 120.4, 110.0, 79.8, 37.7, 28.5; HRMS (EI): Found M^+ 247.1315, $C_{13}H_{17}N_3O_2$ requires 247.1315.

Methyl 2-(1*H*-indazol-3-yl)acetate 32

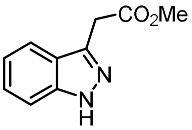

2-(1*H*-Indazol-3-yl)-acetic acid (88 mg, 0.50 mmol) was dissolved in MeOH, 5 drops of conc. H_2SO_4 added and refluxed overnight. Once cooled to room temperature, sat. aq. $NaHCO_3$ was

added until basic and extracted with CH_2Cl_2 (2×). The organic layers were combined, dried ($MgSO_4$), filtered and evaporated to afford title compound **32** (quant., 95 mg) as an oil. ^{1}H NMR ($CDCl_3$, 600 MHz) δ 10.92 (1H, br s), 7.72 (1H, d, *J* = 7.8 Hz), 7.42 (1H, d, *J* = 7.8 Hz), 7.35 (1H, t, *J* = 7.8 Hz), 7.16 (1H, t, *J* = 7.8 Hz), 4.08 (2H, s), 3.71 (3H, s); ^{13}CNMR (151 MHz, $CDCl_3$) δ 170.9, 141.3, 139.7, 127.0, 122.3, 120.9, 120.2, 110.2, 52.4, 33.5; HRMS (ESI): Found MNa^+ 213.0630, $C_{10}H_{10}N_2O_2Na$ requires 213.0645. Data in accordance with literature.[14]

2-(1*H*-Indazol-3-yl)-N-(tetrahydro-2*H*-pyran-4-yl)acetamide 33

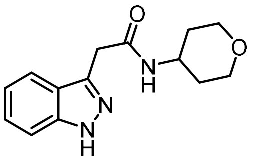

Following **GP6**, 2-(1*H*-indazol-3-yl)acetic acid (88 mg, 0.50 mmol) gave, after purification by column chromatography on silica gel, eluting with EtOAc–MeOH (1:0 to 19:1), **33** (76%, 99 mg) as a solid. R_f 0.14 [EtOAc]; ^{1}H NMR (MeOD, 400 MHz) δ 7.75 (1H, d, *J* = 8.2 Hz), 7.48 (1H, d, *J* = 8.3 Hz), 7.37 (1H, t, *J* = 7.8 Hz), 7.12 (1H, t, *J* = 7.5 Hz), 3.94 – 3.87 (3H, m), 3.45 (2H, td, *J* = 11.6, 2.3 Hz), 1.82 (1H, d, *J* = 12.7 Hz), 1.53 (1H, qd, *J* = 11.6, 4.4 Hz); ^{13}C NMR (101 MHz, MeOD) δ 171.5, 142.7, 141.4, 127.9, 123.2, 121.5, 121.3, 111.1, 67.7, 47.3, 35.6, 33.6; HRMS (EI): Found M^+ 259.1314, $C_{14}H_{17}N_3O_2$ requires 259.1315.

1-(Azetidin-1-yl)-2-(1*H*-indazol-3-yl)ethan-1-one 34

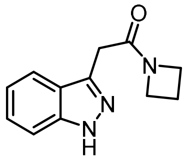

Following **GP6**, 2-(1*H*-indazol-3-yl)acetic acid (88 mg, 0.50 mmol) gave, after purification by column chromatography on silica gel, eluting with EtOAc–MeOH (49:1 to 9:1), **34** (47%, 51 mg) as a solid. R_f 0.30 [EtOAc:MeOH (9:1)]; ^{1}H NMR ($CDCl_3$, 400 MHz) δ 7.82 (1H, d, *J* = 8.1 Hz), 7.40 – 7.29 (2H, m), 7.13 (1H, t, *J* = 7.5 Hz), 4.16 (2H, t, *J* = 7.7 Hz), 4.04 (2H, t, *J* = 7.8 Hz), 3.85 (2H, s), 2.20 (2H, p, *J* = 7.7 Hz); ^{13}C NMR (101 MHz, $CDCl_3$) δ 169.5, 141.4, 140.5, 127.0, 122.4, 120.9, 120.8, 109.9, 50.9, 48.4, 32.0, 15.2; HRMS (EI): Found M^+ 215.1053, $C_{12}H_{13}N_3O$ requires 215.1053

1-(3,4-Dihydroisoquinolin-2(1*H*)-yl)-2-(1*H*-indazol-3-yl)ethan-1-one 35

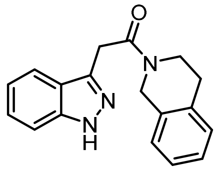

Following **GP6**, 2-(1*H*-indazol-3-yl)acetic acid (88 mg, 0.50 mmol) gave, after purification by column chromatography on silica gel, eluting with pentane–EtOAc (1:1 to 0:1), **35** (67%, 98 mg) as a solid. R_f 0.10 [cyclohexane:EtOAc (1:1)]; ^{1}H NMR ($CDCl_3$, 400 MHz) δ 7.86 (1H, dd, *J* = 8.3, 4.3 Hz), 7.45 – 7.29 (2H, m), 7.21 – 7.10 (4H, m), 7.06 (1H, d, *J* = 7.1 Hz), 4.76 (2H, s), 4.20 (2H, s), 3.84 (2H, dq, *J* = 424.9, 5.8 Hz), 2.77 (2H, app. dt, *J* = 41.3, 5.7 Hz); ^{13}C NMR (101 MHz, $CDCl_3$) δ 168.6, 168.6, 141.3, 141.0, 140.9, 135.1, 134.2, 133.4, 132.8, 128.9, 128.4, 127.2, 127.1, 126.9, 126.8, 126.6, 126.6, 126.4, 126.2, 122.5, 122.4, 121.1, 121.0, 120.9, 120.9, 120.9, 109.8, 48.0, 44.7, 44.0, 40.3, 34.7, 34.6, 29.5, 28.6; *Mixture of rotamers*. HRMS (ESI): Found MNa^+ 314.1258, $C_{18}H_{17}N_3ONa$ requires 314.1264.

***tert*-Butyl 4-(2-(1*H*-indazol-3-yl)acetyl)-1,4-diazepane-1-carboxylate 36**

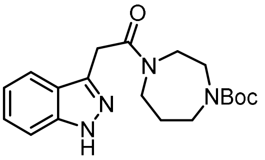

Following **GP6**, 2-(1*H*-indazol-3-yl)acetic acid (88 mg, 0.50 mmol) gave, after purification by column chromatography on silica gel, eluting with petroleum ether–EtOAc (1:1 to 0:1), **36** (quant., 179 mg) as an oil. R_f 0.30 [EtOAc]; ^{1}H NMR ($CDCl_3$, 400 MHz) δ 7.82 (1H, d, *J* = 7.7 Hz), 7.44 – 7.30 (2H, m), 7.13 (2H, t, *J* = 7.4 Hz), 4.10 (2H, app d, *J* = 6.9 Hz), 3.63 (3H, p, *J* = 6.8 Hz), 3.50 (1H, t, *J* = 5.8 Hz), 3.46 – 3.11 (4H, m), 1.96 – 1.70 (2H, m), 1.42 (9H, s); ^{13}CNMR (101 MHz, $CDCl_3$) δ 169.4, 155.5, 155.2, 141.3, 140.8, 127.1, 122.3, 121.0, 120.7, 110.0, 80.0, 49.7, 48.4, 47.8, 47.5, 46.8, 46.0, 45.7, 45.3, 38.8, 34.5, 28.5, 28.5, 25.8; *Mixture of rotamers.* HRMS (ESI): Found MNa^+ 381.1894, $C_{19}H_{26}N_4O_2Na$ requires 381.1897

Methyl 2-((*tert*-butoxycarbonyl)amino)-3-(1*H*-indazol-3-yl)propanoate 37

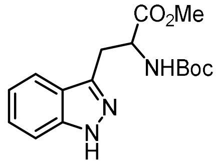

A mixture of methyl 2-amino-4-(2-aminophenyl)-4-oxobutanoate (103 mg, 0.46 mmol, 1.0 equiv.), Boc_2O (122 mg, 0.56 mmol, 1.2 equiv.), Et_3N (0.1 mL, 0.7 mmol, 1.5 equiv.) in dioxane/H_2O 1:1 (0.4 M) was stirred at room temperature for 3 hours under air. The mixture was diluted with water (5 mL), and then extracted with EtOAc (3×10 mL). The combined organic layers were dried ($MgSO_4$), filtered and evaporated. Purification by column chromatography on silica gel, eluting with pentane–EtOAc (7:3), gave methyl 4-(2-aminophenyl)-2-((*tert*-butoxycarbonyl)amino)-4-oxobutanoate (74%, 110 mg) as an oil. R_f 0.38 [pentane:EtOAc (7:3)]; ^1H NMR (400 MHz, $CDCl_3$) δ 7.65 (1H, d, J = 8.2 Hz), 7.29 – 7.22 (1H, m), 6.68 – 6.58 (2H, m), 6.23 (2H, br s), 5.61 (1H, d, J = 8.6 Hz), 4.68 – 4.59 (1H, m), 3.73 – 3.68 (1H, m), 3.72 (3H, s), 3.48 (1H, dd, J = 17.8, 3.5 Hz), 1.43 (9H, s); ^{13}C NMR (101 MHz, $CDCl_3$) δ 199.5, 172.6, 155.8, 150.7, 135.1, 131.2, 117.5, 117.3, 116.1, 80.2, 52.7, 49.7, 41.6, 28.4 (3C); HRMS (ESI): Found MNa^+ 667.2949, $C_{16}H_{22}N_2NaO_5$ requires 667.2950. Data in accordance with literature.[15] Following **GP3**, methyl 4-(2-aminophenyl)-2-((*tert*-butoxycarbonyl)amino)-4-oxobutanoate (97 mg, 0.30 mmol) gave, after purification by column chromatography on silica, eluting with pentane–EtOAc (1:1), **37** (17%, 16 mg) as a solid. R_f 0.30 [pentane: EtOAc (1:1)]; ^1H NMR (400 MHz, $CDCl_3$) δ 10.24 (1H, s), 7.66 (1H, d, J = 8.2 Hz), 7.46 (1H, d, J = 8.4 Hz), 7.37 (1H, t, J = 7.6 Hz), 7.15 (1H, t, J = 7.6 Hz), 5.69 (1H, d, J = 8.6 Hz), 4.80 (1H, dt, J = 8.6, 4.4 Hz), 3.66 (3H, s), 3.58 (1H, dd, J = 15.1, 5.2 Hz), 3.47 (1H, dd, J = 14.8, 5.0 Hz), 1.40 (9H, s); ^{13}C NMR (101 MHz, $CDCl_3$) δ 172.6, 155.6, 142.3, 141.1, 127.1, 122.7, 120.9, 120.0, 110.1, 80.1, 52.9, 52.6, 29.3, 28.4 (3C); HRMS (ESI): Found MNa^+ 342.1420, $C_{16}H_{21}N_3NaO_4$ requires 342.1424.

4-Isobutyl-3-methyl-1*H*-indazole 38

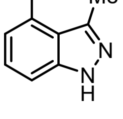

Following **GP7** for 24h, 4-bromo-3-methyl-1*H*-indazole (212 mg, 1.0 mmol) and *i*BuMgBr (1.0 M in THF, 2 mL, 2.0 mmol) gave, after purification by column chromatography on silica gel eluting with pentane–EtOAc–DCM (8:2:1 to 6:3:1), **38** (57%, 107 mg) as a solid. R_f 0.25

[pentane:EtOAc:DCM (8:2:1)]; ^{1}H NMR (600 MHz, $CDCl_3$) δ 9.89 (1H, s), 7.26 (2H, d, J = 4.2 Hz), 6.85 (1H, t, J = 4.0 Hz), 2.87 (2H, d, J = 7.1 Hz), 2.73 (3H, s), 1.94 (1H, hept, J = 6.7 Hz), 0.98 (d, J = 6.5 Hz, 6H); ^{13}C NMR (151 MHz, $CDCl_3$) δ 143.2, 142.2, 136.4, 126.8, 121.7, 121.6, 107.5, 42.4, 30.8, 22.6, 15.3; HRMS (ESI): Found MH^+ 189.1386, $C_{14}H_{19}N_2$ requires 189.1383.

3-Methyl-5-phenethyl-1-indazole 39

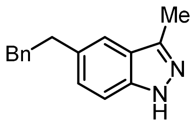

Following **GP4**, 5-bromo-3-methyl-1*H*-indazole (211 mg, 1.0 mmol) gave, after purification by column chromatography on silica, eluting with pentane–EtOAc (3:2), (*E*)-3-methyl-5-styryl-1*H*-indazole (94%, 220 mg) as a solid. R_f 0.44 [pentane: EtOAc (3:2)]; ^{1}H NMR (600 MHz, DMSO-d_6) δ 12.67 (1H, s), 7.87 (1H, s), 7.67 (1H, d, J = 8.6 Hz), 7.60 (2H, d, J = 7.4 Hz), 7.45 (1H, d, J = 8.6 Hz), 7.40 – 7.34 (3H, m), 7.25 (1H, t, J = 7.2 Hz), 7.22 (1H, d, J = 16.6 Hz); ^{13}C NMR (151 MHz, DMSO-d_6) δ 141.5, 140.5, 137.5, 129.2, 128.9, 128.7 (2C), 127.1, 126.1 (2C), 126.1, 124.5, 122.6, 118.5, 110.3, 11.7; HRMS (EI): Found M^+ 234.1146, $C_{16}H_{14}N_2$ requires 234.1157.

A round-bottom flask was charged with (*E*)-3-methyl-5-styryl-1*H*-indazole (47 mg, 0.20 mmol, 1.0 equiv.), Pd/C 10% (21 mg, 0.02 mmol, 0.1 equiv.), and MeOH (2 mL). The reaction mixture was stirred at room temperature for 12 hours under H_2 and then filtered through a pad of celite. Purification by column chromatography on silica gel, eluting with pentane–EtOAc (3:2), gave **39** (81%, 38 mg) as a solid. R_f 0.46 [pentane: EtOAc (3:2)]; ^{1}H NMR (600 MHz, $CDCl_3$) δ 9.80 (1H, s), 7.45 (1H, s), 7.34 (1H, d, J = 8.5 Hz), 7.29 (2H, t, J = 7.6 Hz), 7.23 – 7.19 (4H, m), 3.06 – 3.02 (2H, m), 3.00 – 2.96 (2H, m), 2.57 (3H, s); ^{13}C NMR (151 MHz, $CDCl_3$) δ 143.3, 141.9, 140.2, 133.9, 128.6, 128.5, 128.3, 126.1, 123.2, 119.1, 109.6, 38.7, 38.0, 12.1; HRMS (EI): Found M^+ 236.1309, $C_{16}H_{16}N_2$ requires 236.1313.

5-Isopropyl-3-methyl-1*H*-indazole 40

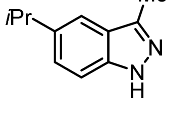

Following **GP2** for 16 h, 5-bromo-3-methyl-1*H*-indazole (212 mg, 1.0 mmol) and Zn(*i*Pr)$_2$ (1.0 M in toluene, 2×2 mL, 2×4 mmol) gave, after purification by column chromatography on silica

gel eluting with DCM–EtOAc (9:1 to 4:1), **40** (99%, 173 mg) as a solid. R_f 0.22 [DCM:EtOAc (4:1)]; ^{1}H NMR (600 MHz, $CDCl_3$) δ 9.77 (1H, br s), 7.48 (1H, s), 7.35 (1H, d, *J* = 8.6 Hz), 7.29 (1H, dd, *J* = 8.6, 1.6 Hz), 3.04 (1H, p, *J* = 6.9 Hz), 2.59 (3H, s), 1.32 (6H, d, *J* = 7.0 Hz); ^{13}C NMR (151 MHz, $CDCl_3$) δ 143.1, 141.0, 139.9, 126.5, 122.9, 116.4, 109.4, 34.0, 24.4, 11.9; HRMS (ESI): Found MH^+ 175.1230, $C_{11}H_{15}N_2$ requires 175.1229.

5-Methyl-1*H*-indazole-3-carbaldehyde 41'

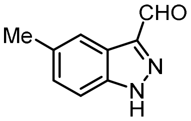

Following **GP1**, 5-methyl-1*H*-indole (394 mg, 3.0 mmol) gave, after purification by column chromatography on silica gel eluting with pentane–EtOAc (4:1), **41'** (79%, 380 mg) as a solid. R_f 0.32 [pentane:EtOAc (4:1)]; ^{1}H NMR (400 MHz, $CDCl_3$) δ 10.85 (1H, s), 10.29 (1H, s), 8.12 (1H, s), 7.47 (1H, d, *J* = 8.6 Hz), 7.32 (1H, dd, *J* = 8.6, 1.6 Hz), 2.50 (3H, s); ^{13}C NMR (101 MHz, $CDCl_3$) δ 187.6, 144.5, 140.0, 134.4, 130.3, 121.6, 121.2, 109.8, 21.6; HRMS (EI): Found M^+ 160.0629, $C_9H_8N_2O$ requires 160.0637.

3-(Cyclopentylmethyl)-5-methyl-1*H*-indazole 41

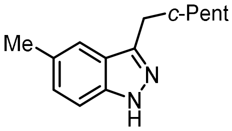

Following **GP1**, but only with *p*TSNHNH$_2$ (114 mg, 0.66 mmol, 1.1 equiv.) in MeOH (1 M) at room temperature for 12 h followed by the removal of the solvent and treatment with Cs_2CO_3 (293 mg, 0.9 mmol, 1.5 equiv.) and cyclopentylboronic acid (103 mg, 0.9 mmol, 1.5 equiv.) in 1,4-dioxane at 110 °C under argon for 12 h, 5-methyl-1*H*-indazole-3-carbaldehyde **41'** (96 mg, 0.6 mmol) gave, after purification by column chromatography on silica gel eluting with pentane–EtOAc (3:2), **41** (80%, 103 mg) as a solid. R_f 0.68 [pentane:EtOAc (4:1)]; ^{1}H NMR (600 MHz, $CDCl_3$) δ 7.49 (1H, s), 7.33 (1H, d, *J* = 8.4 Hz), 7.20 (1H, d, *J* = 8.4 Hz), 2.99 (2H, d, *J* = 7.5 Hz), 2.47 (3H, s), 2.45 – 2.36 (m, 1H), 1.81 – 1.74 (m, 2H), 1.71 – 1.63 (m, 2H), 1.58 – 1.50 (m, 2H), 1.32 (2H, dq, *J* = 14.9, 7.5 Hz); ^{13}C NMR (151 MHz, $CDCl_3$) δ 146.7, 139.9, 129.5, 128.7, 122.9, 119.6, 109.6, 40.3, 33.1, 32.9 (2C), 25.1 (2C), 21.5; HRMS (ESI): Found MH^+ 215.1541, $C_{14}H_{19}N_2$ requires 215.1543.

6-Cyclohexyl-3-methyl-1*H*-indazole 42

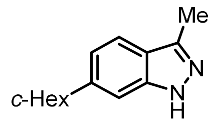

Following **GP7** for 16h, 6-bromo-3-methyl-1*H*-indazole (212 mg, 1.0 mmol) and CyhexMgBr (1.0 M in hexane, 2 mL, 2.0 mmol) gave, after purification by column chromatography on silica gel eluting with DCM–MeOH (99:1 to 97:3), **42** (18%, 39 mg) as a solid. R_f 0.40 [DCM:MeOH (49:1)]; ^{1}H NMR (600 MHz, $CDCl_3$) δ 10.40 (1H, s), 7.59 (1H, d, J = 8.4 Hz), 7.23 (1H, s), 7.04 (1H, d, J = 8.3 Hz), 2.70 – 2.57 (4H, m), 1.94 (2H, d, J = 11.9 Hz), 1.87 (2H, d, J = 11.1 Hz), 1.78 (1H, d, J = 13.4 Hz), 1.57 – 1.36 (4H, m), 1.34 – 1.19 (1H, m); ^{13}C NMR (151 MHz, $CDCl_3$) δ 147.5, 143.2, 141.9, 121.4, 120.7, 119.9, 106.9, 45.1, 34.8, 27.0, 26.3, 12.2; HRMS (ESI): Found MH^+ 215.1540, $C_{14}H_{19}N_2$ requires 215.1543.

7-Benzyl-3-methyl-1*H*-indazole 44

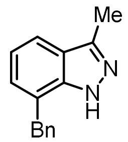

Following **GP7** for 16h, 7-bromo-3-methyl-1*H*-indazole (212 mg, 1.0 mmol) and BenzylMgCl (2.0 M in THF, 1.0 mL, 1 mmol) and gave, after purification by column chromatography on silica gel eluting with pentane–EtOAc–DCM (8:2:1 to 6:3:1), **44** (33%, 73 mg) as a solid. R_f 0.30 [pentane:EtOAc:DCM (8:2:1)]; ^{1}H NMR (600 MHz, $CDCl_3$) δ 9.50 (1H, s), 7.59 (1H, d, J = 8.0 Hz), 7.32 (2H, t, J = 7.0 Hz), 7.28 – 7.24 (3H, m), 7.22 (1H, d, J = 7.0 Hz), 7.13 (1H, t, J = 7.5 Hz), 4.26 (2H, s), 2.58 (3H, s); ^{13}C NMR (151 MHz, $CDCl_3$) δ 143.9, 140.8, 139.0, 129.0, 128.8, 126.9, 126.9, 123.2, 122.8, 120.7, 118.7, 38.4, 12.2; HRMS (ESI): Found MH^+ 223.1224, $C_{15}H_{15}N_2$ requires 223.1229.

6-((*tert*-Butyldimethylsilyl)oxy)-3-methyl-1*H*-indazole 46

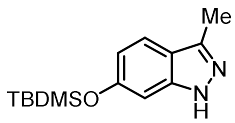

6-Hydroxy-3-methylindazole (104 mg, 0.70 mmol) was dissolved in DMF (5 mL), TBDMSCl (0.13 mL, 0.77 mmol) and imidazole (95 mg, 1.4 mmol) was added to the reaction mixture which was stirred at room temperature overnight. Water (20 mL) was added and the aqueous phase extracted with EtOAc (2×20 mL). The combined organic layers were dried ($MgSO_4$) and

concentrated in vacuo. The title compound was isolated by column chromatography on silica gel, eluting with pentane–EtOAc (4:1 to 7:3), to afford **46** (50%, 92 mg) as an oil. R*f* 0.16 [cyclohexane:EtOAc (4:1)]; ^{1}HNMR ($CDCl_3$, 600 MHz) δ 7.50 (1H, d, *J* = 8.6 Hz), 6.80 (1H, s), 6.71 (1H, d, *J* = 8.5 Hz), 2.55 (3H, s), 1.01 (9H, s), 0.22 (6H, s); ^{13}C NMR (151 MHz, $CDCl_3$) δ 155.6, 143.6, 142.6, 120.9, 118.4, 116.0, 99.1, 25.8, 18.4, 12.1, -4.2; HRMS (EI): Found M$^+$ 262.1495, $C_{14}H_{22}N_2OSi$ requires 262.1496.

6-(Benzyloxy)-3-methyl-1*H*-indazole 47

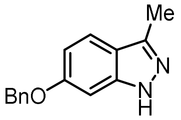

Following **GP8**, with K_2CO_3 (415 mg, 3.0 mmol) at 60 °C, 3-methyl-1*H*-indazol-6-ol (296 mg, 2.0 mmol) and (bromomethyl)benzene (0.31 mL, 2.6 mmol) gave, after purification by column chromatography on silica gel eluting with pentane–EtOAc (3:2), **47** (46%, 218 mg) as a solid. R*f* 0.25 [pentane:EtOAc (3:2)]; ^{1}H NMR (600 MHz, DMSO-*d*$_6$) δ 12.36 (1H, s), 7.55 (1H, d, *J* = 8.7 Hz), 7.47 (2H, d, *J* = 7.5 Hz), 7.40 (2H, t, *J* = 7.3 Hz), 7.33 (1H, t, *J* = 7.3 Hz), 6.91 (1H, s), 6.77 (1H, d, *J* = 8.7 Hz), 5.15 (2H, s), 2.42 (3H, s); ^{13}C NMR (151 MHz, DMSO-*d*$_6$) δ 157.6, 141.8, 140.9, 137.1, 128.4 (2C), 127.8, 127.6 (2C), 120.7, 117.1, 111.6, 92.2, 69.4, 11.7; HRMS (ESI): Found MH$^+$ 239.1171, $C_{15}H_{15}N_2O$ requires 239.1179.

6-Butoxy-3-methyl-1*H*-indazole 48

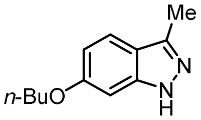

Following **GP8**, but with Cs_2CO_3 (977 mg, 3.0 mmol) at room temperature, 3-methyl-1*H*-indazol-6-ol (296 mg, 2.0 mmol) and 1-iodobutane (0.30 mL, 2.6 mmol) gave, after purification by column chromatography on silica gel eluting with pentane–EtOAc (3:2), **48** (36%, 147 mg) as a solid. R*f* 0.33 [pentane:EtOAc (3:2)]; ^{1}H NMR (400 MHz, $CDCl_3$) 9.67 (1H, s), 7.51 (1H, d, *J* = 8.2 Hz,), 6.79 (1H, dd, *J* = 8.2, 1.8 Hz), 6.78 (1H, s), 4.00 (2H, t, *J* = 6.5 Hz), 2.54 (3H, s), 1.81 (2H, p, *J* = 6.9 Hz), 1.52 (2H, h, *J* = 7.4 Hz), 0.99 (3H, t, *J* = 7.4 Hz); ^{13}C NMR (101 MHz, $CDCl_3$) δ 159.4, 143.6, 142.6, 121.0, 117.6, 112.6, 91.7, 68.1, 31.4, 19.4, 14.0, 12.1; HRMS (ESI): Found MH$^+$ 205.1334, $C_{12}H_{17}N_2O$ requires 205.1336.

6-(Cyclopentyloxy)-3-methyl-1*H*-indazole 49

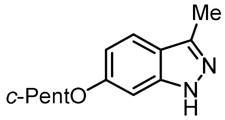

Following **GP8**, with Cs_2CO_3 (977 mg, 3.0 mmol) at room temperature, 3-methyl-1*H*-indazol-6-ol (296 mg, 2.0 mmol) and iodocyclopentane (0.30 mL, 2.6 mmol) gave, after purification by column chromatography on silica gel eluting with pentane–EtOAc (3:2), **49** (64%, 277 mg) as a solid. R_f 0.31 [pentane:EtOAc (3:2)]; ^{1}H NMR (400 MHz, $CDCl_3$) δ 9.62 (1H, s), 7.50 (1H, d, J = 9.5 Hz), 6.79 – 6.71 (2H, m), 4.82 – 4.76 (1H, m), 2.53 (3H, s), 1.96 – 1.86 (4H, m), 1.86 – 1.77 (2H, m), 1.68 – 1.58 (2H, m); ^{13}C NMR (101 MHz, $CDCl_3$) δ 158.3, 143.6, 142.6, 121.0 (2C), 117.5, 113.4 (2C), 93.0, 79.7, 33.0 (2C), 24.2 (2C), 12.1; HRMS (ESI): Found MH^+ 217.1333, $C_{13}H_{17}N_2O$ requires 217.1336.

1,3-Dimethyl-1*H*-indazole 52

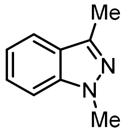

Following **GP3**, 1-(2-(methylamino)phenyl)ethan-1-one (14.9 mg, 1.0 mmol) gave, after purification by column chromatography on silica gel eluting with pentane–EtOAc (3:2), **52** (90%, 132 mg) as a solid. R_f 0.46 [pentane: EtOAc (3:2)]; ^{1}H NMR (600 MHz, $CDCl_3$) δ 7.65 (1H, d, J = 8.1 Hz), 7.40 – 7.35 (1H, m), 7.32 (1H, d, J = 8.4 Hz), 7.11 (1H, t, J = 7.4 Hz), 4.00 (3H, s), 2.58 (3H, s); ^{13}C NMR (151 MHz, $CDCl_3$) δ 141.4, 140.9, 126.3, 123.4, 120.5, 119.7, 108.9, 35.2, 12.0; HRMS (ESI): Found MH^+ 147.0920, $C_9H_{10}N_2$ requires 147.0917. Data in accordance with the literature.[16]

4. 2*H*-indazole synthesis

2-Methyl-2*H*-indazole 53

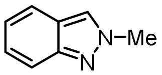

Following **GP9**, 1*H*-indazole (591 mg, 5.0 mmol) gave, after purification by column chromatography on silica gel eluting with pentane–EtOAc (3:2), **53** (84%, 555 mg) as an oil. R_f 0.42 [pentane:EtOAc (3:2)]; ^{1}H NMR (600 MHz, $CDCl_3$) δ 7.87 (1H, s), 7.69 (1H, d, *J* = 8.7 Hz), 7.63 (1H, d, *J* = 8.4 Hz), 7.30 – 7.25 (1H, m), 7.10 – 7.05 (1H, m), 4.21 (3H, s); ^{13}C NMR (151 MHz, $CDCl_3$) δ 149.2, 126.0, 123.6, 122.2, 121.7, 120.0, 117.3, 40.4; HRMS (EI): Found M^+ 132.0682, $C_8H_8N_2$ requires 132.0687. Data in accordance with literature.[17]

2,3-Dimethyl-2*H*-indazole 54

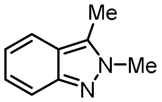

Following **GP9**, 3-methyl-1*H*-indazole (396 mg, 3.0 mmol) gave, after purification by column chromatography on silica gel eluting with pentane–EtOAc (3:2), **54** (51%, 224 mg) as a solid. R_f 0.27 [pentane:EtOAc (3:2)]; ^{1}H NMR (600 MHz, $CDCl_3$) δ 7.61 (1H, d, *J* = 8.6 Hz), 7.54 (1H, d, *J* = 8.4 Hz), 7.29 – 7.23 (1H, m), 7.02 (1H, t, *J* = 7.4 Hz), 4.09 (3H, s), 2.60 (3H, s); ^{13}C NMR (151 MHz, $CDCl_3$) δ 147.8, 131.6, 126.2, 121.2, 120.5, 119.7, 116.9, 37.4, 10.0; HRMS (EI): Found M^+ 146.0837, $C_9H_{10}N_2$ requires 146.0844. Data in accordance with literature.[18]

6-Methoxy-2-methyl-2*H*-indazole 55

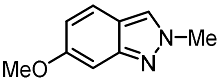

Following **GP9**, 6-methoxy-1*H*-indazole (178 mg, 1.2 mmol) gave, after purification by column chromatography on silica gel eluting with pentane–EtOAc (3:2), **55** (76%, 148 mg) as a solid. R_f 0.22 [pentane:EtOAc (3:2)]; ^{1}H NMR (400 MHz, $CDCl_3$) δ 7.76 (1H, s), 7.49 (1H, d, *J* = 9.1 Hz), 6.94 (1H, s), 6.77 (1H, dd, *J* = 9.1, 2.1 Hz), 4.14 (3H, s), 3.85 (3H, s); ^{13}C NMR (101 MHz, $CDCl_3$) δ 158.8, 150.2, 123.7, 120.9, 117.6, 116.6, 94.6, 55.3, 40.2; HRMS (EI): Found M^+ 162.0787, $C_9H_{10}N_2O$ requires 162.0793.

2,5-Dimethyl-2*H*-indazole 56

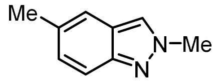

Following **GP9**, 5-methyl-1*H*-indazole (106 mg, 0.80 mmol) gave, after purification by column chromatography on silica gel eluting with pentane–EtOAc (3:2), **56** (74%, 87 mg) as a solid. R_f 0.17 [pentane:EtOAc (3:2)]; ^{1}H NMR (400 MHz, $CDCl_3$) δ 7.76 (1H, s), 7.59 (1H, d, *J* = 8.9 Hz), 7.38 (1H, s), 7.12 (1H, d, *J* = 8.9 Hz), 4.19 (3H, s), 2.41 (3H, s); ^{13}C NMR (101 MHz, $CDCl_3$) δ 148.1, 131.0, 128.8, 122.7, 122.5, 118.2, 117.0, 40.3, 21.8; HRMS (EI): Found M^+ 146.0837, $C_9H_{10}N_2$ requires 146.0844.

2,4-Dimethyl-2*H*-indazole 57

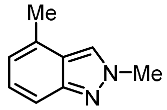

Following **GP9**, 4-methyl-1*H*-indazole (66 mg, 0.50 mmol) gave, after purification by column chromatography on silica gel, eluting with pentane–EtOAc (4:1 to 1:1), **57** (quant., 73 mg) as a solid. R_f 0.43 [cyclohexane:EtOAc (1:1)]; ^{1}H NMR ($CDCl_3$, 600 MHz) δ 7.88 (1H, s), 7.52 (1H, d, *J* = 8.7 Hz), 7.18 (1H, dd, *J* = 8.7, 6.7 Hz), 6.83 (1H, d, *J* = 6.7 Hz), 4.22 (3H, s), 2.51 (3H, s); ^{13}C NMR (151 MHz, $CDCl_3$) δ 149.3, 130.2, 126.3, 123.6, 122.8, 121.0, 114.7, 40.4, 19.3; HRMS (ESI): Found MH^+ 147.0916, $C_9H_{11}N_2$ requires 147.0922. Data in accordance with literature.[19]

2-(4-Methylbenzyl)-2*H*-indazole 58

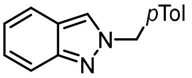

Following **GP10**, 2-nitrobenzaldehyde (151 mg, 1 mmol) and *p*-tolylmethanamine (133 mg, 1.1 mmol) gave, after purification by column chromatography on silica gel eluting with pentane–EtOAc (8:2), **58** (80%, 178 mg) as a solid. R_f 0.35 [pentane:EtOAc (4:1)]; ^{1}H NMR (600 MHz, $CDCl_3$) δ 7.86 (1H, s), 7.73 (1H, dd, *J* = 8.5, 1.1 Hz), 7.61 (1H, dt, *J* = 8.5, 1.1 Hz), 7.30 – 7.25 (1H, m), 7.20 (2H, d, *J* = 7.9 Hz), 7.16 (2H, d, *J* = 7.9 Hz), 7.06 (1H, ddd, *J* = 8.5, 6.6, 1.1 Hz), 5.56 (2H, s), 2.34 (3H, s); ^{13}C NMR (101 MHz, $CDCl_3$) δ 149.1, 138.4, 132.9, 129.7 (2C), 128.3 (2C), 126.0, 122.8, 122.2, 121.8, 120.3, 117.7, 57.5, 21.3; HRMS (EI): Found M^+ 222.1150, $C_{15}H_{14}N_2$ requires 222.1157.

2-Cyclohexyl-2*H*-indazole 59

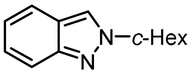

Following **GP10**, 2-nitrobenzaldehyde (453 mg, 3 mmol) and cyclohexanamine (0.38 mL, 3.3 mmol) gave, after purification by column chromatography on silica gel eluting with pentane–EtOAc (9:1), **59** (69%, 415 mg) as a solid. R_f 0.55 [pentane:EtOAc (9:1)]; ^{1}H NMR (600 MHz, $CDCl_3$) δ 7.94 (1H, s), 7.72 (1H, d, *J* = 8.7 Hz), 7.65 (1H, d, *J* = 8.4 Hz), 7.29 – 7.24 (1H, m), 7.06 (1H, dd, *J* = 8.4, 6.6 Hz), 4.40 (1H, tt, *J* = 11.8, 3.9 Hz), 2.31 – 2.24 (2H, m), 1.98 – 1.87 (4H, m), 1.81 – 1.76 (1H, m), 1.49 (2H, qt, *J* = 12.8, 3.6 Hz), 1.33 (1H, qt, *J* = 13.2, 3.7 Hz); ^{13}C NMR (151 MHz, $CDCl_3$) δ 148.4, 125.7, 121.5, 121.5, 120.3, 120.2, 117.6, 63.1, 34.1 (2C), 25.6 (2C), 25.5; HRMS (EI): Found M^+ 200.1310, $C_{13}H_{16}N_2$ requires 200.1313. Data in accordance with literature.[20]

2-(2*H*-Indazol-2-yl)-2-methylpropan-1-ol 60

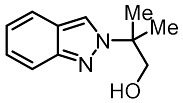

Following **GP10**, 2-nitrobenzaldehyde (151 mg, 1 mmol) and 2-amino-2-methylpropan-1-ol (0.11 mL, 1.1 mmol) gave, after purification by column chromatography on silica gel eluting with pentane–EtOAc (9:1), **60** (71%, 135 mg) as a solid. R_f 0.31 [pentane:EtOAc (9:1)]; ^{1}H NMR (400 MHz, $CDCl_3$) δ 8.05 (1H, s), 7.68 (2H, t, *J* = 7.8 Hz), 7.33 – 7.27 (1H, m), 7.12 – 7.05 (1H, m), 4.25 (1H, t, *J* = 6.7 Hz), 3.92 (2H, d, *J* = 6.7 Hz), 1.70 (6H, s); ^{13}C NMR (101 MHz, $CDCl_3$) δ 148.2, 126.3, 121.8, 121.2, 120.6, 120.4, 117.4, 71.0, 63.3, 25.5; HRMS (EI): Found M^+ 190.1101, $C_{11}H_{14}N_2O$ requires 190.1106.

(*R*)-2-(1-Phenylethyl)-2*H*-indazole 61

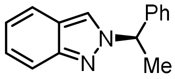

Following **GP10**, 2-nitrobenzaldehyde (453 mg, 3 mmol) and (*R*)-1-phenylethan-1-amine (0.42 mL, 3.3 mmol) gave, after purification by column chromatography on silica gel eluting with pentane–EtOAc (9:1), **61** (85%, 567 mg) as a solid. R_f 0.45 [pentane:EtOAc (9:1)]; ^{1}H NMR (600 MHz, $CDCl_3$) δ 7.90 (1H, s), 7.73 (1H, dd, *J* = 8.7, 1.1 Hz), 7.61 (1H, d, *J* = 8.4 Hz), 7.35 – 7.31 (2H, m), 7.30 – 7.24 (4H, m), 7.07 – 7.04 (1H, m), 5.83 (1H, q, *J* = 7.1 Hz), 2.04 (3H, d, *J* = 7.1 Hz); ^{13}C NMR (151 MHz, $CDCl_3$) δ 148.7, 141.2, 129.0 (2C), 128.3, 126.7

(2C), 126.0, 121.8, 121.8, 121.5, 120.3, 117.8, 62.8, 21.8; HRMS (EI): Found M^+ 222.1151, $C_{15}H_{14}N_2$ requires 222.1157. Data in accordance with literature.[21]

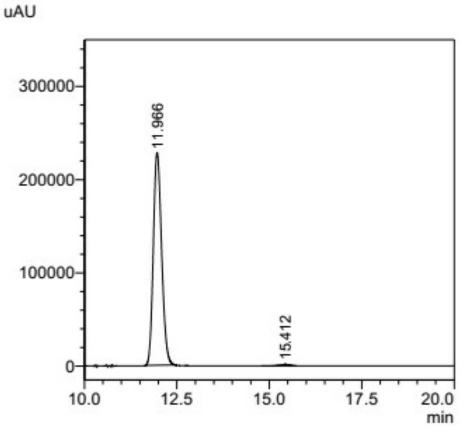

PDA Ch1 254nm

Peak#	Ret. Time	Area	Area%
1	11.966	3562739	99.282
2	15.412	25758	0.718
Total		3588496	100.000

Figure S1. Chromatogram of **61** - HPLC (CHIRALCEL® OD-H).

2-(1-Phenylethyl)-2*H*-indazole 61-rac

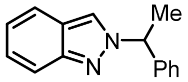

Following **GP10**, 2-nitrobenzaldehyde (302 mg, 2.0 mmol) and 1-phenylethan-1-amine (0.28 mL, 2.2 mmol) gave, after purification by column chromatography on silica gel eluting with pentane–EtOAc (9:1), **S67-rac** (80%, 356 mg) as a solid. R_f 0.45 [pentane:EtOAc (9:1)]; ^{1}H NMR (400 MHz, $CDCl_3$) δ 7.91 (1H, s), 7.74 (1H, dd, *J* = 8.8, 1.0 Hz), 7.62 (1H, dt, *J* = 8.4, 1.1 Hz), 7.37 – 7.31 (2H, m), 7.31 – 7.24 (4H, m), 7.07 (1H, ddd, *J* = 8.4, 6.6, 0.9 Hz), 5.84 (1H, q, *J* = 7.1 Hz), 2.05 (3H, d, *J* = 7.1 Hz); ^{13}C NMR (101 MHz, $CDCl_3$) δ 148.7, 141.2, 129.0 (2C), 128.3, 126.7 (2C), 126.0, 121.8, 121.8, 121.5, 120.3, 117.8, 62.8, 21.8; HRMS (EI): Found M^+ 222.1154, $C_{15}H_{14}N_2$ requires 222.1157.

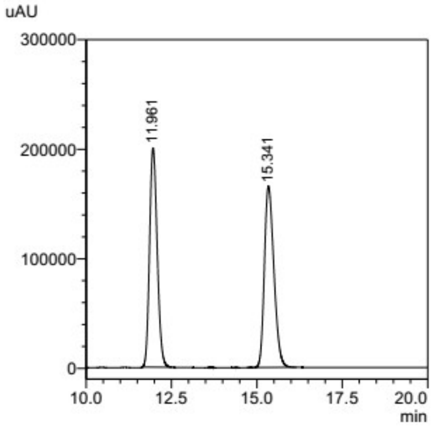

PDA Ch1 254nm

Peak#	Ret. Time	Area	Area%
1	11.961	3114986	49.269
2	15.341	3207383	50.731
Total		6322369	100.000

Figure S2. Chromatogram of **61-rac** - HPLC (CHIRALCEL® OD-H).

(*R*)-2-(2,3-dihydro-1*H*-inden-1-yl)-2*H*-indazole 62

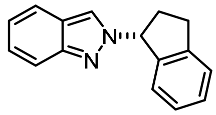

Following **GP10**, 2-nitrobenzaldehyde (151 mg, 1 mmol) and (*R*)-2,3-dihydro-1*H*-inden-1-amine (0.14 mL, 1.1 mmol) gave, after purification by column chromatography on silica gel eluting with pentane–EtOAc (9:1), **62** (50%, 117 mg) as a solid. R_f 0.35 [pentane:EtOAc (9:1)]; ^{1}H NMR (400 MHz, $CDCl_3$) δ 7.74 (1H, d, *J* = 8.8 Hz), 7.71 (1H, s), 7.59 (1H, d, *J* = 8.4 Hz), 7.39 (1H, d, *J* = 7.4 Hz), 7.35 (1H, t, *J* = 7.4 Hz), 7.30 – 7.22 (2H, m), 7.20 (1H, d, *J* = 7.3 Hz), 7.10 – 7.03 (1H, m), 6.18 (1H, dd, *J* = 8.1, 5.6 Hz), 3.22 (ddd, *J* = 15.6, 8.4, 5.8 Hz, 1H), 3.04 (1H, ddd, *J* = 15.6, 8.4, 6.0 Hz), 2.84 (1H, dtd, *J* = 14.0, 8.1, 5.8 Hz), 2.54 (1H, ddt, *J* = 14.0, 8.7, 5.6 Hz); ^{13}C NMR (101 MHz, $CDCl_3$) δ 148.9, 144.4, 140.7, 129.2, 127.3, 126.0, 125.4, 125.3, 121.9, 121.7, 121.3, 120.3, 117.7, 68.6, 34.6, 30.7; HRMS (EI): Found M^+ 234.1154, $C_{16}H_{14}N_2$ requires 234.1157.

***tert*-Butyl 2-((4*R*,6*R*)-6-(2-(2*H*-indazol-2-yl)ethyl)-2,2-dimethyl-1,3-dioxan-4-yl)acetate 63**

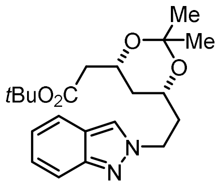

Following **GP10**, 2-nitrobenzaldehyde (151 mg, 1 mmol) and *tert*-butyl 2-((4*R*,6*R*)-6-(2-aminoethyl)-2,2-dimethyl-1,3-dioxan-4-yl)acetate (0.30 mL, 1.1 mmol) gave, after purification by column chromatography on silica gel eluting with pentane–EtOAc (9:1), **63** (83%, 311 mg) as a solid. R_f 0.31 [pentane:EtOAc (9:1)]; ^{1}H NMR (400 MHz; CDCl3) δ 7.89 (1H, s), 7.70 (1H, dd, *J* = 8.8, 1.1 Hz), 7.64 (1H, dt, *J* = 8.4, 1.1 Hz), 7.30 – 7.26 (1H, m), 7.10 – 7.05 (1H, m), 4.53 (2H, dd, *J* = 8.1, 5.5 Hz), 4.19 – 4.11 (1H, m), 3.72 – 3.64 (1H, m), 2.39 (1H, dd, *J* = 15.1, 7.1 Hz), 2.31 – 2.22 (2H, m), 2.06 – 1.96 (1H, m), 1.50 (1H, dt, *J* = 12.7, 2.5 Hz), 1.42 (9H, s), 1.40 (s, 3H), 1.38 (s, 3H), 1.28 – 1.18 (m, 1H); ^{13}C NMR (101 MHz, $CDCl_3$) δ 170.2, 149.2, 126.0, 123.4, 121.7, 121.7, 120.2, 117.5, 99.0, 80.8, 66.3, 65.6, 49.6, 42.8, 36.9, 36.4, 30.3, 28.2 (3C), 19.9; HRMS (ESI): Found MNa^+ 397.2103, $C_{21}H_{30}N_2NaO_4$ requires 397.2098.

5. Overview of indazole starting materials

Guideline substrates

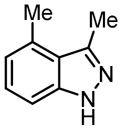
2

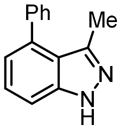
3

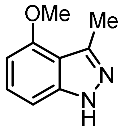
4

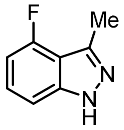
5

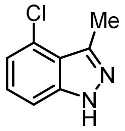
6

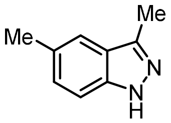
7

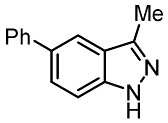
8

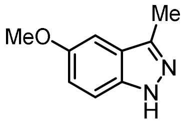
9

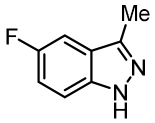
10

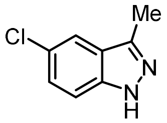
11

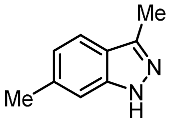
12

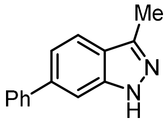
13

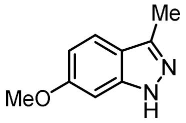
14

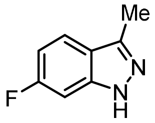
15

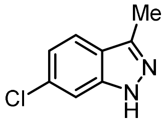
16

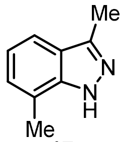
17

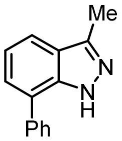
18

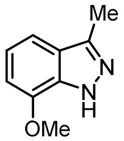
19

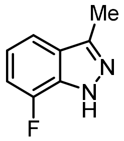
20

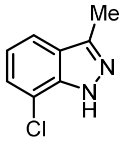
21

1*H*-indazole substrates

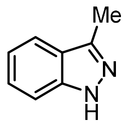
1

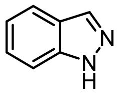
22

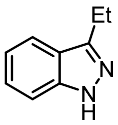
23

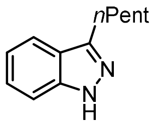
24

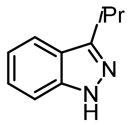
25

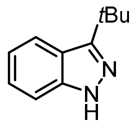
26

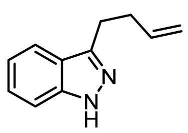
27

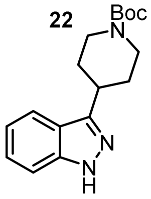
28

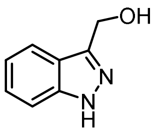
29

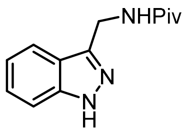
30

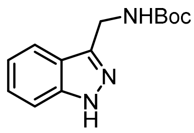
31

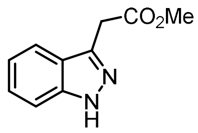
32

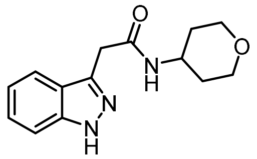
33

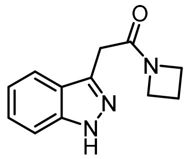
34

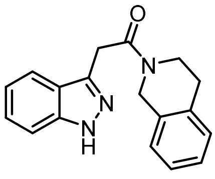
35

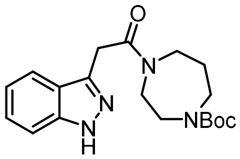
36

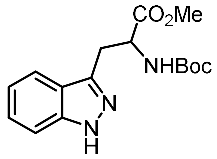
37

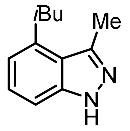
38

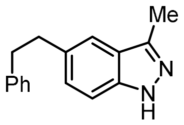
39

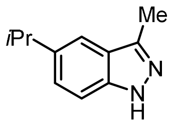
40

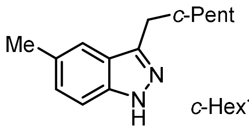
41

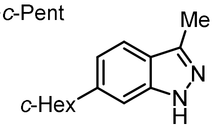
42

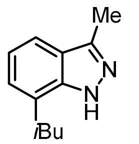
43

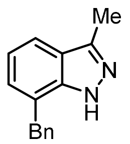
44

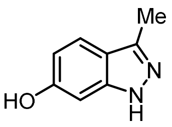
45

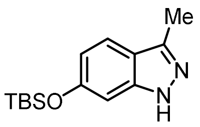
46

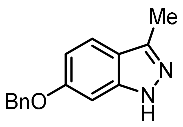
47

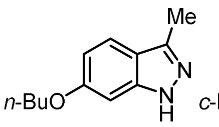
48

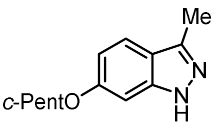
49

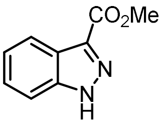
50*

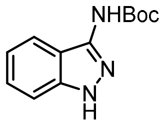
51*

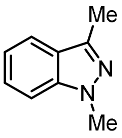
52*

**Scope limitations*

2*H*-indazole substrates

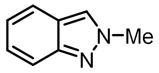

53

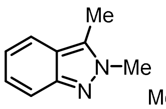

54

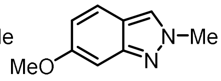

55

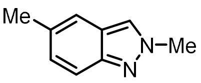

56

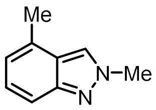

57

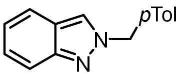

58

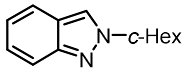

59

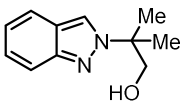

60

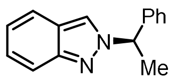

61

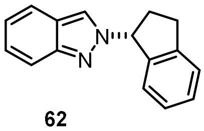

62

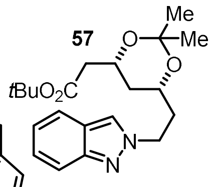

63

6. Reaction optimization for the photo-permutation of 1*H*-indazoles

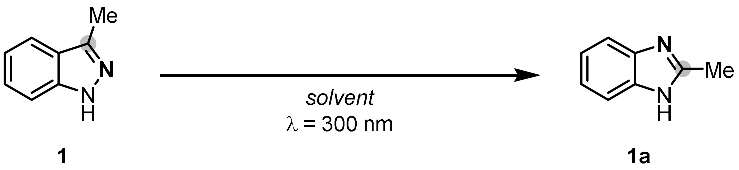

A microwave vial was charged with the 1*H*-indazole **1** (1.0 equiv.) and the tube was capped with a Supelco aluminium crimp seal with septum (PTFE/butyl), evacuated and refilled with N_2 (×3). Then degassed HFIP was added, the lid sealed with parafilm, and the reaction mixture placed under 300 nm light. After the specified time, the reaction mixture was removed, a solution of 1,3-dinitrobenzene in $CDCl_3$ (1.0 equiv, 0.2 M) was added as an internal standard and the reaction analysed by ^1H NMR.

Entry	Solvent	Concentration	Time	NMR Yield	RSM
		Solvent screen			
**1**	MeOH	0.05 M	16 h	**20%**	**78%**
**2**	^{i}PrOH	0.05 M	16 h	**21%**	**53%**
**3**	Isobutanol	0.05 M	16 h	**n.d.**	**80%**
**4**	TFE	0.05 M	16 h	**27%**	**71%**
**5**	HFIP	0.05 M	16h	**86%**[a]	**n.d.**
**6**	AcOEt	0.05 M	16 h	**7%**	**93%**
**7**	DCE	0.05 M	16 h	**n.d.**	**65%**
**8**	CH_3CN	0.05 M	16 h	**5%**	**91%**
**9**	Toluene	0.05 M	16 h	**n.d.**	**90%**
**10**	H_2O	0.05 M	16 h	**n.d.**	**95%**
		Concentration screen			
**11**	HFIP	0.025 M	16 h	**81%**	**8%**
**12**	HFIP	0.075 M	16 h	**84%**	**11%**
**13**	HFIP	0.1 M	16 h	**71%**	**25%**
**14**	HFIP	0.2 M	16 h	**69%**	**27%**
		Time screen			
**15**	HFIP	0.05 M	4 h	**46%**	**48%**
**16**	HFIP	0.05 M	8 h	**54%**	**45%**
**17**	HFIP	0.05 M	12 h	**67%**	**31%**

[a]Isolated Yield.

7. Reaction optimization for the photo-permutation of 2*H*-indazoles

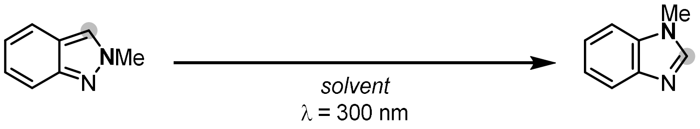

53 53a

A microwave vial was charged with the 2-methylindazole **55** (1.0 equiv.) and the tube was capped with a Supelco aluminium crimp seal with septum (PTFE/butyl), evacuated and refilled with N_2 (×3). Then degassed HFIP was added, the lid sealed with parafilm, and the reaction mixture placed under 300 nm light. After 16 hours, the reaction mixture was removed, a solution of 1,3-dinitrobenzene in $CDCl_3$ (1.0 equiv, 0.2 M) was added as an internal standard and the reaction analysed by ^{1}H NMR.

Entry	Solvent	Concentration	Additive	NMR Yield	RSM
**1**	HFIP	0.05 M	-	**64%**	**23%**
**2**	HFIP	0.025 M	-	**55%**	**25%**
			Additive screen		
**3**	HFIP	0.05 M	Et_3N (1.0 equiv.)	**56%**	**41%**
**4**	HFIP	0.05 M	AcOH (1.0 equiv.)	**30%**	**36%**
			Solvent screen		
**5**	MeOH	0.05 M	-	**76%**	**7%**
**6**	AcOEt	0.05 M	-	**80%**	**n.d.**
**7**	DCE	0.05 M	-	**84%**	**3%**
**8**	DME	0.025 M	-	**92%**	**3%**
**9**	Et_2O	0.075 M	-	**64%**	**28%**
**10**	DMF	0.1 M	-	**94%**	**n.d.**
**11**	1,4-dioxane	0.05 M	-	**95%**	**n.d**
**12**	CH_3CN	0.05 M	-	**95%**	**n.d.**
			Concentration screen		
**13**	CH_3CN	0.025 M	-	**96%**[c]	**n.d.**
**14**	CH_3CN	0.1 M	-	**73%**	**21%**
			Wavelength screen		
**15**[a]	CH_3CN	0.025 M	-	**95%**	**n.d.**
**16**[b]	CH_3CN	0.025 M	-	**n.d.**	**100%**

[a]310 nm; [b]350 nm; [c]Isolated Yield.

8. Pictures of reaction setup

Reactions were run on an RPR-200 Rayonet reactor using 300 nm light from 16 lamps. The carousel was placed in the photoreactor, and a fan placed above to keep the temperature stable (max. 35 °C). The reaction vials were placed on the outside of the carousel and no stirrer bars were added as the reaction did not require stirring for the photo-permutation to proceed.

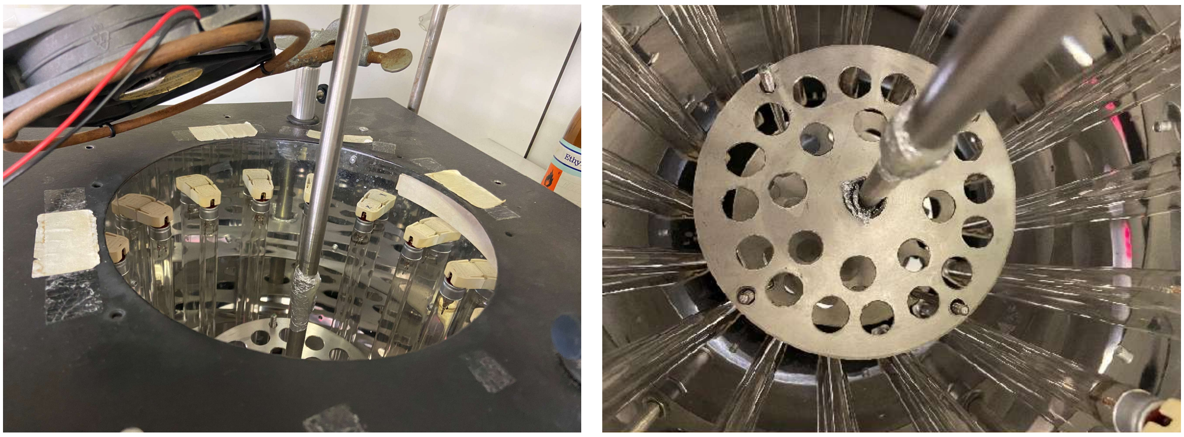

Picture S1 – Reaction setup for the photo-permutation at 300 nm.

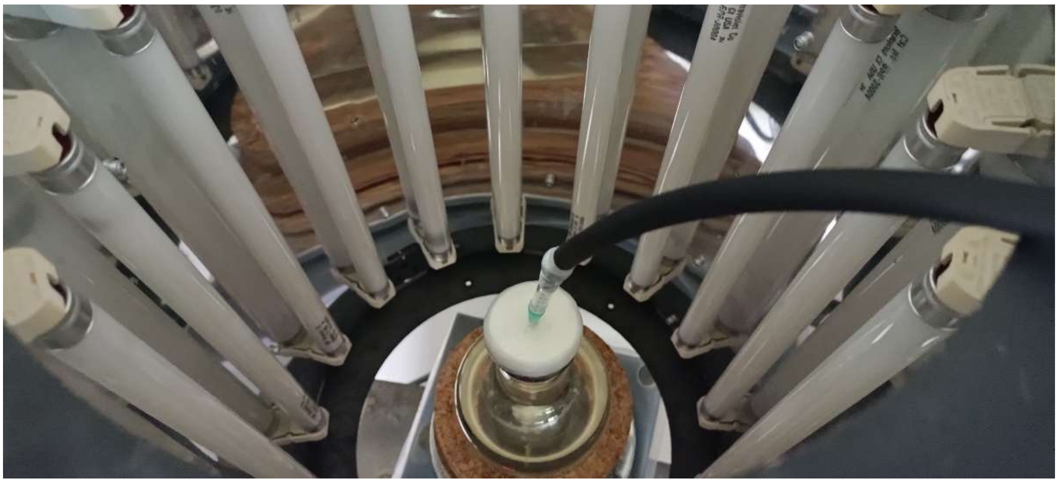

Picture S2. Set-Up for the gram-scale reaction of **1**.

9. Permutation products – 1*H*-indazoles

2-Methyl-*1H*-benzo[*d*]imidazole 1a

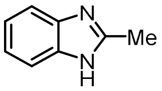

Following **GP11**, **1** (13.2 mg, 0.10 mmol) gave, after purification by column chromatography on silica gel eluting with EtOAc–MeOH (4:1), **1a** (86%, 11 mg) as a solid. R_f 0.38 [EtOAc:MeOH (4:1)]; ^{1}H NMR (400 MHz, $CDCl_3$) δ 7.55 (2H, dd, *J* = 6.0, 3.2 Hz), 7.25 – 7.19 (2H, m), 2.65 (3H, s); ^{13}C NMR (101 MHz, $CDCl_3$) δ 151.3, 138.8, 122.3, 114.7, 15.1; HRMS (ESI): Found MH^+ 133.0761, $C_8H_9N_2$ requires 133.0760. Data in accordance with literature.[22] Gram-Scale Reaction: Following **GP11**, but with a reaction time of 48 hours, **1** (1.3 g, 10 mmol) gave, after purification by column chromatography on silica gel eluting with EtOAc–MeOH (4:1), **1a** (62%, 814 mg) as a solid.

2,4/7-Dimethyl-1*H*-benzo[*d*]imidazole 2a and 17a

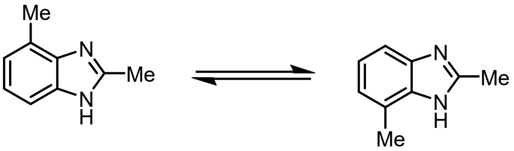

2a **17a**

Following **GP11** at 0.025 M, 3,4-dimethyl-1*H*-indazole **2** (14.6 mg, 0.10 mmol) gave, after purification by column chromatography on silica gel eluting with EtOAc–MeOH (9:1), **2a** (86%, 13 mg) as a solid.

Following **GP11** at 0.025 M, 3,7-dimethyl-1*H*-indazole **17** (14.6 mg, 0.10 mmol) gave, after purification by column chromatography on silica gel eluting with EtOAc–MeOH (9:1), **17a** (60%, 9 mg) as a solid.

R_f 0.47 [EtOAc:MeOH (9:1)]; ^{1}H NMR (600 MHz, $CDCl_3$) δ 7.35 (1H, d, *J* = 8.0 Hz), 7.12 (1H, t, *J* = 7.6 Hz), 7.02 (1H, d, *J* = 7.6 Hz), 2.61 (3H, s), 2.57 (3H, s); ^{13}C NMR (151 MHz, $CDCl_3$) δ 150.8, 138.7, 137.8, 125.1, 122.9, 122.3, 111.6, 17.2, 15.0; HRMS (EI): Found M^+ 146.0834, $C_9H_{10}N_2$ requires 146.0844. Data in accordance with literature.[23]

2-Methyl-4-phenyl-1*H*-benzo[d]imidazole 3a

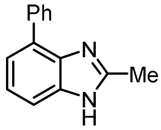

Following **GP11** at 0.025 M, **3** (21 mg, 0.10 mmol) gave, after purification by column chromatography on silica gel, eluting with pentane–EtOAc (1:1 to 0:1), **3a** (45%, 9 mg) as a solid. R_f 0.41 [EtOAc]; ^{1}HNMR (600 MHz, $CDCl_3$) δ 7.70 (2H, d, *J* = 7.0 Hz), 7.56 (1H, dd, *J* = 5.7, 3.4 Hz), 7.44 (2H, t, *J* = 7.7 Hz), 7.34 (1H, td, *J* = 7.2, 1.3 Hz), 7.31 – 7.28 (2H, m), 2.55 (3H, s); ^{13}CNMR (151 MHz, $CDCl_3$) δ 151.3, 138.8, 132.2, 129.0, 128.8, 128.7, 128.5, 127.6, 122.7, 122.2, 115.3, 15.1; HRMS (EI): Found M^+ 208.0992, $C_{14}H_{12}N_2$ requires 208.0995.

4/7-Fluoro-2-methyl-1*H*-benzo[d]imidazole 5a and 20a

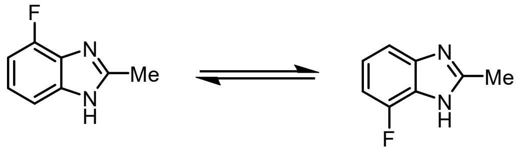

5a **20a**

Following **GP11** at 0.025 M, 4-fluoro-3-methyl-1*H*-indazole **5** (15 mg, 0.10 mmol) gave, after purification by column chromatography on silica gel eluting with pentane–EtOAc (4:1 to 0:1), **5a** (81%, 12 mg) as a solid.

Following **GP11** at 0.025 M, 7-fluoro-3-methyl-1*H*-indazole **20** (13.2 mg, 0.10 mmol) gave, after purification by column chromatography on silica gel eluting with EtOAc–MeOH (4:1), **20a** (70%, 11 mg) as a solid.

R_f 0.48 [EtOAc:MeOH (4:1)]; ^{1}H NMR (600 MHz, DMSO-d_6, major isomer) δ 12.47 (1H, s), 7.23 (1H, d, *J* = 7.1 Hz), 7.11 – 7.05 (1H, m), 6.92 – 6.85 (1H, m), 2.49 (3H, s); ^{1}H NMR (600 MHz, DMSO-d_6, minor isomer) δ 12.70 (1H, s), 7.34 (1H, d, *J* = 7.1 Hz), 7.11 – 7.05 (1H, m), 6.99 – 6.92 (1H, m), 2.49 (3H, s); ^{13}C NMR (DMSO-d_6, 151 MHz) δ 152.5 (*J* = 248.1 Hz), 151.9, 137.6 (*J* = 9.8 Hz), 131.6 (*J* = 16.7 Hz), 121.8 (*J* = 7.3 Hz), 107.1 (*J* = 3.5 Hz), 106.2 (*J* = 17.7 Hz), 14.5; ^{19}F NMR (565 MHz, DMSO-d_6) δ -129.69; HRMS (EI): Found M^+ 150.0584, $C_8H_7FN_2$ requires 150.0593. Data in accordance with literature.[24]

4/7-Chloro-2-methyl-1*H*-benzo[d]imidazole 6a and 21a

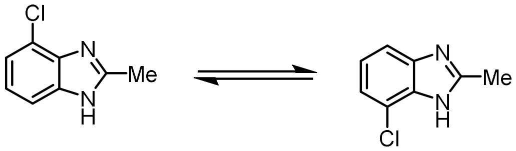

6a **21a**

Following **GP11** at 0.025 M, 4-chloro-3-methyl-1*H*-indazole **6** (17 mg, 0.10 mmol) gave, after purification by column chromatography on silica gel, eluting with EtOAc–MeOH (1:0 to 98:2), **6a** (91%, 15 mg) as a solid.

Following **GP11** at 0.025 M, 7-chloro-3-methyl-1*H*-indazole **21** (17 mg, 0.10 mmol) gave, after purification by column chromatography on silica gel, eluting with EtOAc–MeOH (1:0 to 9:1), **21a** (70%, 12 mg) as a solid.

R_f 0.29 [EtOAc]; ^{1}HNMR ($CDCl_3$, 400 MHz) δ 7.43 (1H, dd, *J* = 7.9, 1.1 Hz), 7.23 (1H, dd, *J* = 7.8, 1.1 Hz), 7.14 (1H, t, *J* = 7.9 Hz), 2.66 (3H, s); ^{13}C NMR (151 MHz, $CDCl_3$) δ 152.2, 137.6, 136.2, 123.6, 122.7, 120.2, 112.6, 14.6; HRMS (ESI): Found MH^+ 167.0366, $C_8H_8N_2Cl$ requires 167.0371. Data in accordance with literature.[25]

2,5/6-Dimethyl-1*H*-benzo[d]imidazole 7a and 12a

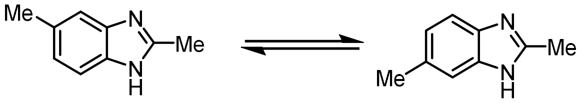

7a **12a**

Following **GP11** at 0.025 M, 3,5-dimethyl-1*H*-indazole **7** (15 mg, 0.10 mmol) gave, after purification by column chromatography on silica gel eluting with pentane–EtOAc (4:1 to 0:1), **7a** (38%, 6 mg) as a solid. Following **GP11** at 0.0075 M for 16 h gave **7a** (76%, 11 mg).

Following **GP11** at 0.025 M, 3,6-dimethyl-1*H*-indazole **12** (15 mg, 0.10 mmol) gave, after purification by column chromatography on silica gel, eluting with EtOAc–MeOH (1:0 to 19:1), **12a** (93%, 14 mg) as a solid.

R_f 0.18 [EtOAc]; ^{1}H NMR (600 MHz, $CDCl_3$) δ 7.42 (d, *J* = 8.2 Hz, 1H), 7.31 (s, 1H), 7.04 (dd, *J* = 8.2, 1.6 Hz, 1H), 2.60 (s, 3H), 2.45 (s, 3H); ^{13}C NMR (151 MHz, $CDCl_3$) δ 150.7, 138.6, 137.3, 132.1, 123.8, 114.6, 114.1, 21.7, 15.2; HRMS (ESI): Found MH^+ 147.0913, $C_9H_{11}N_2$ requires 147.0917. Data in accordance with literature.[26]

5/6-Fluoro-2-methyl-1*H*-benzo[d]imidazole 10a and 15a

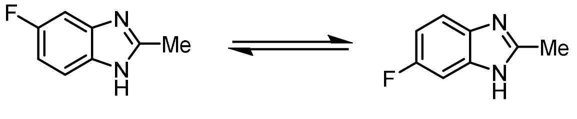

10a **15a**

Following **GP11** at 0.025 M, 5-fluoro-3-methyl-1*H*-indazole **10** (15 mg, 0.10 mmol) gave, after purification by column chromatography on silica gel eluting with pentane–EtOAc (4:1 to 0:1), **10a** (45%, 7 mg) as a solid. Following **GP11** at 0.0075 M for 24 h, gave **10a** (83%, 12 mg).

Following **GP11** at 0.025 M, 6-fluoro-3-methyl-1*H*-indazole **15** (15 mg, 0.10 mmol) gave, after purification by column chromatography on silica gel eluting with EtOAc–MeOH (4:1), **15a** (42%, 6 mg) as an oil. Following **GP11** at 0.0075 M for 24 h, gave **15a** (77%, 12 mg).

R_f 0.12 [pentane:EtOAc (1:9)]; ^{1}H NMR (600 MHz, $CDCl_3$) δ 7.44 (1H, dd, J = 8.8, 4.7 Hz), 7.21 (1H, d, J = 9.0 Hz), 6.97 (1H, td, J = 9.1, 2.4 Hz), 2.63 (3H, s); ^{13}C NMR (151 MHz, $CDCl_3$) δ 159.5 (d, J = 237.8 Hz), 152.4, 139.0, 135.2, 115.0, 110.5 (d, J = 25.4 Hz), 101.1, 15.2; ^{19}F NMR (565 MHz, $CDCl_3$) δ -120.66; HRMS (ESI): Found MH^+ 151.0662, $C_8H_8N_2F$ requires 151.0666; Data in accordance with literature.[27]

5/6-chloro-2-methyl-1*H*-benzo[d]imidazole 11a and 16a

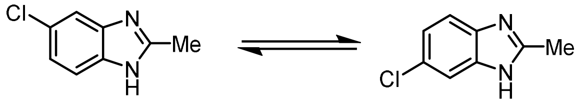

11a **16a**

Following **GP11** at 0.025 M, 5-chloro-3-methyl-1*H*-indazole **11** (17 mg, 0.10 mmol) gave, after purification by column chromatography on silica gel, eluting with EtOAc–MeOH (1:0 to 95:5), **11a** (34%, 6 mg) as a solid.

Following **GP11** at 0.025 M, 6-chloro-3-methyl-1*H*-indazole **16** (16.7 mg, 0.10 mmol) gave, after purification by column chromatography on silica gel eluting with EtOAc–MeOH (4:1), **16a** (82%, 14 mg) as a solid.

R_f 0.34 [EtOAc:MeOH (4:1)]; ^{1}H NMR (600 MHz, $CDCl_3$) δ 7.51 (1H, s), 7.43 (1H, d, J = 8.5 Hz), 7.19 (1H, dd, J = 8.5, 1.9 Hz), 2.62 (3H, s); ^{13}C NMR (151 MHz, $CDCl_3$) δ 152.2, 137.2, 128.1, 123.0, 115.4, 114.7, 15.2; HRMS (EI): Found M^+ 166.0291, $C_8H_7ClN_2$ requires 166.0298. Data in accordance with literature.[28]

2-Methyl-6-phenyl-1*H*-benzo[*d*]imidazole 13a

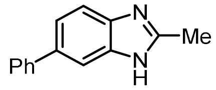

Following **GP11** at 0.025 M, **13** (20.8 mg, 0.10 mmol) gave, after purification by column chromatography on silica gel eluting with EtOAc– MeOH (4:1), **13a** (13%, 3 mg) as an oil. Following **GP11** at 0.0075 M for 24 h, gave **13a** (74%, 15 mg). R_f 0.47 [EtOAc:MeOH (4:1)]; ^{1}H NMR (600 MHz, $CDCl_3$) δ 7.74 (1H, s), 7.61 (3H, d, *J* = 8.0 Hz), 7.48 (1H, dd, *J* = 8.3, 1.7 Hz), 7.42 (2H, t, *J* = 7.6 Hz), 7.32 (1H, t, *J* = 7.4 Hz), 2.66 (3H, s); ^{13}C NMR (151 MHz, $CDCl_3$) δ 152.0, 141.9, 139.0, 138.5, 136.2, 128.9 (2C), 127.5 (2C), 126.9, 122.2, 115.0, 112.8, 15.1; HRMS (EI): Found M^+ 208.0990, $C_{14}H_{12}N_2$ requires 208.1000.

6-Methoxy-2-methyl-1*H*-benzo[d]imidazole 14a

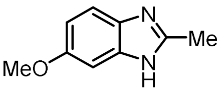

Following **GP11** at 0.025 M, **14** (16 mg, 0.10 mmol) gave, after purification by column chromatography on silica gel, eluting with EtOAc–MeOH (1:0 to 19:1), **14a** (61%, 10 mg) as a solid. R_f 0.10 [EtOAc]; ^{1}HNMR (600 MHz, $CDCl_3$) δ 7.42 (d, *J* = 8.7 Hz, 1H), 7.01 (d, *J* = 2.5 Hz, 1H), 6.85 (dd, *J* = 8.7, 2.5 Hz, 1H), 3.81 (s, 3H), 2.60 (s, 3H); ^{13}CNMR (151 MHz, $CDCl_3$) δ 156.3, 151.0, 139.0, 133.6, 115.3, 111.5, 97.7, 56.0, 15.0; HRMS (EI): Found M^+ 162.0788, $C_9H_{10}N_2O$ requires 162.0788. Data in accordance with literature.[29]

1*H*-Benzo[d]imidazole 22a

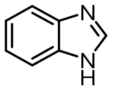

Following **GP11**, **22** (11.8 mg, 0.10 mmol) gave, after purification by column chromatography on silica gel eluting with EtOAc–MeOH (4:1), **22a** (28%, 3 mg) as a solid. R_f 0.45 [EtOAc:MeOH (4:1)]; ^{1}H NMR (400 MHz, DMSO-d_6) δ 12.45 (1H, s), 8.21 (1H, s), 7.59 (2H, dd, *J* = 6.0, 3.3 Hz), 7.21–7.16 (2H, m); ^{13}C NMR (101 MHz, DMSO-d_6) δ 141.9, 138.0, 121.7, 115.3; GC-MS m/z (EI): 118.10 (M^+), 63.15. Data in accordance with literature.[30]

2-Ethyl-1*H*-benzo[d]imidazole 23a

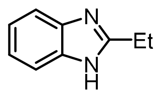

Following **GP11**, **23** (15 mg, 0.10 mmol) gave, after purification by column chromatography on silica gel, eluting with pentane–EtOAc (1:1 to 0:1), **23a** (89%, 13 mg) as a solid. R_f 0.41 [EtOAc]; ^{1}H NMR (600 MHz, $CDCl_3$) δ 7.55 (2H, dd, *J* = 6.0, 3.2 Hz), 7.22 (2H, dd, *J* = 6.0, 3.2 Hz), 2.98 (2H, q, *J* = 7.7 Hz), 1.45 (3H, t, *J* = 7.7 Hz); ^{13}C NMR (151 MHz, $CDCl_3$) δ 156.2, 138.8, 122.3, 114.7, 22.8, 12.4; HRMS (ESI): Found MH^+ 147.0916, $C_9H_{11}N_2$ requires 147.0917. Data in accordance with literature.[26]

2-Pentyl-1*H*-benzo[d]imidazole 24a

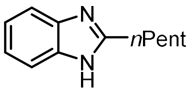

Following **GP11**, **24** (19 mg, 0.10 mmol) gave, after purification by column chromatography on silica gel, eluting with pentane–EtOAc (4:1 to 1:1), **24a** (quant., 19 mg) as a solid. R_f 0.37 [pentane:EtOAc (1:1)]; ^{1}H NMR ($CDCl_3$, 600 MHz) δ 7.55 (2H, dd, *J* = 5.8, 3.1 Hz), 7.21 (2H, dd, *J* = 5.8, 3.1 Hz), 2.94 (2H, t, *J* = 7.7 Hz), 1.86 (2H, p, *J* = 7.7 Hz), 1.38 – 1.22 (4H, m), 0.83 (3H, t, *J* = 7.2 Hz); ^{13}C NMR (151 MHz, $CDCl_3$) δ 155.6, 138.6, 122.2, 114.7, 31.6, 29.5, 28.2, 22.5, 14.0; HRMS (ESI): Found MH^+ 189.1384, $C_{12}H_{17}N_2$ requires 189.1386. Data in accordance with literature.[26]

2-Isopropyl-1*H*-benzo[d]imidazole 25a

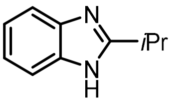

Following **GP11**, **25** (16, 0.10 mmol) gave, after purification by column chromatography on silica gel, eluting with pentane–EtOAc (4:2 to 1:1), **25a** (94%, 15 mg) as a solid. R_f 0.30 [cyclohexane:EtOAc (1:1)]; ^{1}H NMR ($CDCl_3$, 600 MHz) δ 7.49 (2H, dd, *J* = 6.0, 3.1 Hz), 7.15 (2H, dd, *J* = 6.0, 3.1 Hz), 3.19 (1H, hept, *J* = 7.0 Hz), 1.40 (6H, d, *J* = 7.0 Hz); ^{13}C NMR (101 MHz, $CDCl_3$) δ 158.1, 130.9, 126.0, 114.2, 27.8, 21.0; HRMS (EI): Found M^+ 160.0999, $C_{10}H_{12}N_2$ requires 160.0995. Data in accordance with literature.[26]

2-(tert-butyl)-1*H*-benzo[d]imidazole 26a

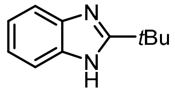

Following **GP11**, **26** (24 mg, 0.14 mmol) gave, after purification by column chromatography on silica gel, eluting with pentane–EtOAc (7:3 to 2:3), **26a** (78%, 19 mg) as a solid. R_f 0.38 [cyclohexane:EtOAc (1:1)]; ^{1}H NMR (DMSO, 600 MHz) δ 7.74 – 7.70 (2H, m), 7.44 – 7.39 (2H, m), 1.51 (9H, s); ^{13}C NMR (151 MHz, DMSO) δ 161.0, 132.8, 124.5, 114.0, 33.4, 28.4; HRMS (EI): Found M^+ 174.1152, $C_{11}H_{14}N_2$ requires 174.1152. Data in accordance with literature.[31]

2-(But-3-en-1-yl)-1*H*-benzo[d]imidazole 27a

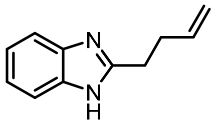

Following **GP11**, **27** (17 mg, 0.10 mmol) gave, after purification by column chromatography on silica gel, eluting with pentane–EtOAc (4:1 to 1:1), **27a** (76%, 13 mg) as a solid. R_f 0.34 [pentane:EtOAc (1:1)]; ^{1}H NMR (600 MHz, $CDCl_3$) δ 7.55 (2H, s), 7.22 (2H, dd, *J* = 6.0, 3.2 Hz), 5.92 (1H, ddt, *J* = 17.1, 10.3, 6.6 Hz), 5.13 (1H, dd, *J* = 17.1, 1.4 Hz), 5.07 (1H, dd, *J* = 10.3, 1.4 Hz), 3.04 (2H, t, *J* = 7.5 Hz), 2.63 (2H, q, *J* = 7.4 Hz); ^{13}C NMR (101 MHz, $CDCl_3$) δ 153.8, 135.6, 134.3, 124.4, 117.1, 114.4, 31.8, 27.3; (ESI): Found MH^+ 173.1070, $C_{11}H_{13}N_2$ requires 173.1073. Data in accordance with literature.[32]

***tert*-Butyl 4-(1*H*-benzo[d]imidazole-2-yl)piperidine-1-carboxylate 28a**

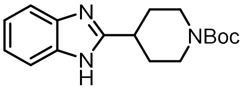

Following **GP11**, **28** (30 mg, 0.10 mmol) gave, after purification by column chromatography on silica gel, eluting with pentane–EtOAc (1:1 to 3:7), **28a** (86%, 26 mg) as a solid. R_f 0.24 [cyclohexane:EtOAc (1:1)]; ^{1}H NMR ($CDCl_3$, 600 MHz) δ 7.53 (2H, app. s), 7.19 (2H, dd, *J* = 6.1, 3.2 Hz), 4.19 (2H, br s), 3.11 (1H, tt, *J* = 12.1, 3.6 Hz), 2.82 (2H, br s), 2.07 (1H, d, *J* = 12.1 Hz), 1.88 (1H, qt, *J* = 12.1, 3.6 Hz), 1.45 (9H, s); ^{13}C NMR (151 MHz, $CDCl_3$) δ 157.4, 154.9, 138.3, 122.3, 114.9, 80.1, 44.2, 37.1, 30.9, 28.5; HRMS (ESI): Found MH^+ 302.1857, $C_{17}H_{24}N_3O_2$ requires 302.1863. Data in accordance with literature.[33]

(1*H*-Benzo[*d*]imidazol-2-yl)methanol 29a

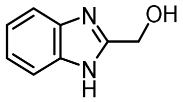

Following **GP11**, **29** (14.8 mg, 0.10 mmol) gave, after purification by column chromatography on silica gel eluting with EtOAc–MeOH (4:1), **29a** (74%, 11 mg) as a solid. R*f* 0.31 [EtOAc:MeOH (4:1)]; ^{1}H NMR (600 MHz, CD_3OD) δ 7.54 – 7.50 (2H, m), 7.21 – 7.18 (2H, m), 4.83 (2H, s); ^{13}C NMR (151 MHz, CD_3OD) δ 156.2, 139.3, 123.3 (2C), 115.6 (2C), 58.9; HRMS (EI): Found M^+ 148.0631, $C_8H_8N_2O$ requires 148.0637. Data in accordance with literature.[34]

***N*-((1*H*-Benzo[d]imidazol-2-yl)methyl)pivalamide 30a**

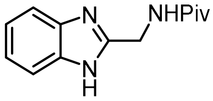

Following **GP11**, but stirring for 24 h **30** (20 mg, 0.09 mmol) gave, after purification by column chromatography on silica gel, eluting with EtOAc–MeOH (1:0 to 9:1), **30a** (80%, 17 mg) as a solid. R*f* 0.35 [EtOAc]; ^{1}HNMR ($CDCl_3$, 400 MHz) δ 7.58 (2H, dd, *J* = 5.9, 3.2 Hz), 7.39 (1H, s), 7.29 – 7.22 (2H, m), 4.62 (2H, d, *J* = 5.8 Hz), 1.25 (9H, s); ^{13}C NMR (101 MHz, $CDCl_3$) δ 182.2, 150.4, 130.5, 126.8, 114.4, 39.3, 35.2, 27.7; HRMS (ESI): Found MNa^+ 254.1268, $C_{13}H_{17}N_3ONa$ requires 254.1264.

***tert*-Butyl ((1*H*-benzo[d]imidazole-2-yl)methyl)carbamate 31a**

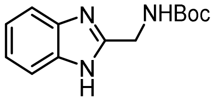

Following **GP11,** but stirring for 24 h **31** (25 mg, 0.10 mmol) gave, after purification by column chromatography on silica gel, eluting with pentane–EtOAc (7:3 to 1:3), **31a** (61%, 15 mg) as a solid. R*f* 0.18 [pentane:EtOAc (1:1)]; ^{1}H NMR (600 MHz, $CDCl_3$) δ 7.58 (s, 2H), 7.25 (s, 2H), 5.55 (s, 1H), 4.51 (app. d, *J* = 6.4 Hz, 2H), 1.47 (s, 9H); ^{13}C NMR (151 MHz, $CDCl_3$) δ 157.4, 152.2, 122.7, 80.7, 39.1, 28.4; HRMS (ESI): Found MH^+ 248.1390, $C_{13}H_{18}O_2N_3$ requires 248.1394. Data in accordance with literature.[35]

Methyl 2-(1*H*-benzo[d]imidazol-2-yl)acetate 32a

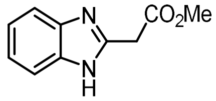

Following **GP11**, **32** (19 mg, 0.10 mmol) gave, after purification by column chromatography on silica gel, eluting with pentane–EtOAc (1:1 to 0:1), **32a** (84%, 16 mg) as an oil. R_f 0.38 [EtOAc]; ^{1}H NMR ($CDCl_3$, 400 MHz) δ 7.58 (2H, br s), 7.26 – 7.23 (2H, m), 4.09 (2H, s), 3.79 (3H, s); ^{13}CNMR (101 MHz, $CDCl_3$) δ 170.4, 146.9, 122.8, 52.8, 34.5; HRMS (ESI): Found MH^+ 191.0810, $C_{11}H_{13}O_2N_2$ requires 191.0815. Data in accordance with literature.[36]

2-(1*H*-Benzo[d]imidazol-2-yl)-N-(tetrahydro-2*H*-pyran-4-yl)acetamide 33a

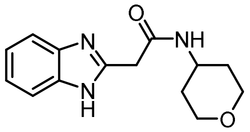

Following **GP11**, but stirring for 20 h **33** (26 mg, 0.10 mmol) gave, after purification by column chromatography on silica gel, eluting with EtOAc–MeOH (19:1 to 9:1), **33a** (52%, 13 mg) as a solid. R_f 0.16 [EtOAc:MeOH (19:1)]; ^{1}H NMR (DMSO, 400 MHz) δ 12.21 (1H, s), 8.29 (1H, d, J = 7.3 Hz), 7.48 (2H, br s), 7.12 (2H, dd, J = 6.4, 3.0 Hz), 3.90 – 3.66 (5H, m), 3.37 (2H, dd, J = 11.2, 2.6 Hz), 1.71 (2H, dd, J = 12.8, 2.6 Hz), 1.42 (2H, qd, J = 11.2, 4.3 Hz); ^{13}C NMR (151 MHz, DMSO) δ 166.0, 149.3, 137.0, 122.2, 114.5, 65.8, 45.3, 35.7, 32.4; HRMS (ESI): Found MH^+ 260.1396, $C_{14}H_{18}N_2O_2$ requires 260.1394.

1-(Azetidin-1-yl)-2-(1*H*-benzo[d]imidazole-2-yl)ethan-1-one 34

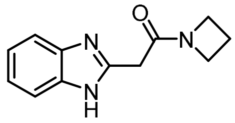

Following **GP11**, but stirring for 22 h **34** (22 mg, 0.10 mmol) gave, after purification by column chromatography on silica gel, eluting with EtOAc–MeOH (19:1 to 4:1), **34a** (47%, 10 mg) as an oil. R_f 0.21 [EtOAc:MeOH (9:1)]; ^{1}H NMR ($CDCl_3$, 400 MHz) δ 7.55 (2H, br s), 7.22 (2H, dd, J = 6.0, 3.3 Hz), 4.32 (2H, t, J = 7.7 Hz), 4.09 (2H, t, J = 7.7 Hz), 3.81 (2H, s), 2.32 (2H, p, J = 7.7 Hz); ^{13}C NMR (101 MHz, $CDCl_3$) δ 168.3, 148.2, 122.5, 50.9, 48.4, 31.4, 15.1; HRMS (ESI): Found MNa^+ 238.0955, $C_{12}H_{13}N_3ONa$ requires 238.0951.

2-(1*H*-Benzo[d]imidazol-2-yl)-1-(3,4-dihydroisoquinolin-2(1*H*)-yl)ethan-1-one 35a

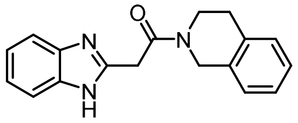

Following **GP11**, **35** (29 mg, 0.10 mmol) gave, after purification by column chromatography on silica gel, eluting with pentane–EtOAc (1:3 to 0:1), **35a** (55%, 16 mg) as an oil. R_f 0.35 [EtOAc]; ^{1}HNMR ($CDCl_3$, 600 MHz) δ 7.55 (2H, dd, *J* = 6.0, 3.2 Hz), 7.22 – 7.17 (4H, m), 7.16 – 7.07 (2H, m), 4.78 (2H, d, *J* = 26.0 Hz), 4.21 (2H, d, *J* = 16.7 Hz), 3.87 (2H, dt, *J* = 9.9, 6.0 Hz), 2.88 (2H, dt, *J* = 9.2, 5.9 Hz); ^{13}CNMR (151 MHz, $CDCl_3$) δ 167.8, 167.8, 148.5, 134.7, 134.1, 132.8, 132.0, 128.8, 128.5, 127.3, 127.0, 126.9, 126.8, 126.7, 126.4, 122.5, 122.5, 47.9, 44.7, 44.2, 40.5, 34.3, 34.2, 29.4, 28.5; *Mixture of rotamers*. HRMS (ESI): Found MH^+ 292.1447, $C_{18}H_{18}N_3O$ requires 292.1444.

***tert*-Butyl 4-(2-(1*H*-benzo[d]imidazole-2-yl)acetyl)-1,4-diazepane-1-carboxylate 36a**

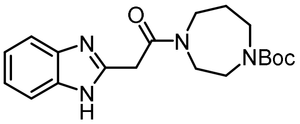

Following **GP11**, but stirring for 22 h **36** (36 mg, 0.10 mmol) gave, after purification by column chromatography on silica gel, eluting with EtOAc–MeOH (99:1 to 19:1), **36a** (64%, 23 mg) as a solid. R_f 0.12 [EtOAc]; ^{1}H NMR ($CDCl_3$, 400 MHz) δ 7.55 (2H, dd, *J* = 6.0, 3.2 Hz), 7.23 (2H, dd, *J* = 6.0, 3.2 Hz), 4.12 (2H, d, *J* = 6.5 Hz), 3.77 – 3.29 (6H, m), 1.95 – 1.82 (1H, m), 1.41 (9H, s). ^{13}CNMR^{13}C NMR (101 MHz, $CDCl_3$) δ 168.5, 148.4, 138.1, 122.7, 115.1, 80.3, 49.2, 48.1, 47.7, 47.5, 47.2, 46.6, 46.2, 45.8, 38.8, 33.8, 33.3, 28.5, 28.5, 27.5, 25.8; *Mixture of rotamers*. HRMS (ESI): Found MNa^+ 381.1903, $C_{19}H_{26}N_4O_3Na$ requires 381.1897.

Methyl 3-(1*H*-benzo[*d*]imidazol-2-yl)-2-((tert-butoxycarbonyl)amino)propanoate 37a

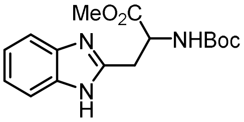

Following **GP11**, **37** (32 mg, 0.10 mmol) gave, after purification by column chromatography on silica gel eluting with EtOAc–MeOH (8:2), **37a** (43%, 14 mg) as a solid. R_f 0.27 [EtOAc:MeOH (8:2)]; ^{1}H NMR (400 MHz, $CDCl_3$) δ 7.54 (2H, s), 7.22 (2H, dq, *J* = 7.0, 3.9 Hz), 5.87 (1H, d, *J* = 7.4 Hz), 4.79 (1H, s), 3.73 (3H, s), 3.51 – 3.38 (2H, m), 1.41 (9H, s); ^{13}C

NMR (101 MHz, $CDCl_3$) δ 172.1, 156.1, 150.5, 131.1, 129.0, 122.6, 80.9, 53.0, 52.1, 32.6, 28.4; HRMS (EI): Found M^+ 319.1527, $C_{16}H_{21}N_3O_4$ requires 319.1532.

4/7-Isobutyl-2-methyl-1*H*-benzo[*d*]imidazole 38a and 43a

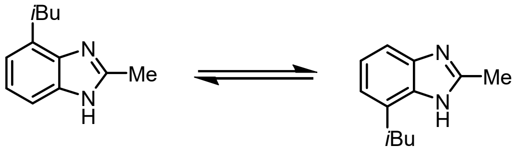

38a **43a**

Following **GP11**, **38** (17 mg, 0.10 mmol) gave, after purification by column chromatography on silica gel eluting with pentane–EtOAc (8:2 to 0:1), **38a** (58%, 11 mg) as a solid.
Following **GP11**, **43** (17 mg, 0.10 mmol) gave, after purification by column chromatography on silica gel eluting with pentane–EtOAc (8:2 to 0:1), **43a** (30%, 6 mg) as a solid.
R_f 0.12 [pentane:EtOAc (1:9)]; ^{1}H NMR (600 MHz, $CDCl_3$) δ 7.39 (1H, d, J = 8.0 Hz), 7.14 (1H, t, J = 7.6 Hz), 7.00 (1H, d, J = 7.3 Hz), 2.77 (2H, d, J = 7.3 Hz), 2.61 (3H, s), 2.08 (1H, hept, J = 6.8 Hz), 0.87 (6H, d, J = 6.2 Hz); ^{13}C NMR (151 MHz, $CDCl_3$) δ 150.7, 138.9, 137.8, 128.3, 122.9, 122.1, 112.4, 41.1, 29.3, 22.7, 15.0; HRMS (ESI): Found MH^+ 189.1387, $C_{12}H_{17}N_2$ requires 189.1386.

2-Methyl-5-phenethyl-1*H*-benzo[*d*]imidazole 39a

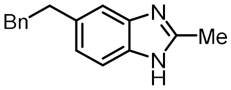

Following **GP11**, **39** (23.6 mg, 0.10 mmol) gave, after purification by column chromatography on silica gel eluting with EtOAc–MeOH (8:2), **39a** (87%, 21 mg) as an oil. R_f 0.40 [EtOAc:MeOH (8:2)]; ^{1}H NMR (400 MHz, $CDCl_3$) δ 7.47 (1H, d, J = 8.2 Hz), 7.34 (1H, s), 7.27 – 7.21 (2H, m), 7.19 – 7.13 (3H, m), 7.06 (1H, d, J = 8.2 Hz), 3.05 – 2.98 (2H, m), 2.98 – 2.91 (2H, m), 2.61 (3H, s); ^{13}C NMR (101 MHz, $CDCl_3$) δ 151.6, 141.9, 138.7, 137.5, 136.1, 128.6 (2C), 128.4 (2C), 125.9, 123.1, 114.5, 113.7, 38.7, 38.2, 14.9; HRMS (EI): Found M^+ 236.1308, $C_{16}H_{16}N_2$ requires 236.1313.

5-Isopropyl-2-methyl-1*H*-benzo[*d*]imidazole 40a

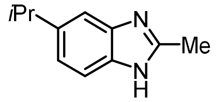

Following **GP11**, **40** (15 mg, 0.10 mmol) gave, after purification by column chromatography on silica gel eluting with pentane–EtOAc (8:2 to 0:1), **40a** (56%, 10 mg) as a solid. R_f 0.16 [pentane:EtOAc (1:9)]; ^{1}H NMR (600 MHz, $CDCl_3$) δ 7.45 (1H, d, *J* = 8.2 Hz), 7.37 (1H, s), 7.11 (1H, dd, *J* = 8.3, 1.7 Hz), 3.02 (1H, hept, *J* = 6.7 Hz), 2.61 (3H, s), 1.29 (6H, d, *J* = 6.9 Hz); ^{13}C NMR (151 MHz, $CDCl_3$) δ 150.7, 143.7, 131.0, 129.6, 129.0, 121.5, 114.6, 34.4, 24.7, 15.2; HRMS (ESI): Found MH^+ 175.1233, $C_{11}H_{15}N_2$ requires 175.1240.

2-(Cyclopentylmethyl)-5-methyl-1*H*-benzo[*d*]imidazole 41a

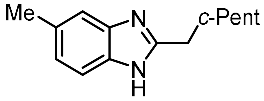

Following **GP11**, **41** (21.4 mg, 0.10 mmol) gave, after purification by column chromatography on silica gel eluting with EtOAc–MeOH (8:2), **41a** (77%, 17 mg) as a solid. R_f 0.44 [EtOAc:MeOH (8:2)]; ^{1}H NMR (600 MHz, $CDCl_3$) δ 7.44 (1H, d, *J* = 8.2 Hz), 7.34 (1H, s), 7.04 (1H, d, *J* = 8.2, 1.6 Hz), 2.94 (2H, d, *J* = 7.7 Hz), 2.45 (3H, s), 2.44 – 2.39 (1H, m), 1.80 – 1.73 (2H, m), 1.63 – 1.56 (2H, m), 1.53 – 1.45 (2H, m), 1.29 – 1.22 (2H, m); ^{13}C NMR (151 MHz, $CDCl_3$) δ 155.2, 138.6, 137.1, 131.8, 123.5, 114.6, 114.3, 39.6, 35.4, 32.7 (2C), 25.0 (2C), 21.7; HRMS (EI): Found M^+ 214.1466, $C_{14}H_{18}N_2$ requires 214.1470.

6-Cyclohexyl-2-methyl-1*H*-benzo[d]imidazole 42a

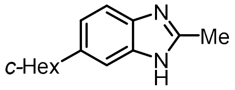

Following **GP11**, **42** (21 mg, 0.10 mmol) gave, after purification by column chromatography on silica gel eluting with pentane–EtOAc (4:1 to 0:1), **42a** (75%, 16 mg) as a solid. R_f 0.12 [pentane:EtOAc (1:9)]; ^{1}H NMR (600 MHz, $CDCl_3$) δ 7.45 (1H, d, *J* = 8.2 Hz), 7.36 (1H, s), 7.09 (1H, d, *J* = 8.2 Hz), 2.64 – 2.56 (4H, m), 1.91 (2H, d, *J* = 12.0 Hz), 1.84 (2H, d, *J* = 12.1 Hz), 1.75 (1H, d, *J* = 13.3 Hz), 1.51 – 1.35 (4H, m), 1.28 – 1.23 (1H, m); ^{13}C NMR (151 MHz, $CDCl_3$) δ 151.0, 143.0, 142.9, 122.0 121.8, 114.4, 111.9, 44.9, 35.2, 27.2, 26.3, 15.0; HRMS (ESI): Found MH^+ 215.1547, $C_{14}H_{19}N_2$ requires 215.1557.

7-Benzyl-2-methyl-1*H*-benzo[d]imidazole 44a

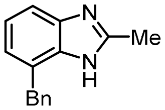

Following **GP11**, **44** (22 mg, 0.10 mmol) gave, after purification by column chromatography on silica gel eluting with pentane–EtOAc (4:1 to 0:1), **44a** (49%, 11 mg) as a solid. R_f 0.12 [pentane:EtOAc (1:9)]; ^{1}H NMR (600 MHz, $CDCl_3$) δ 7.41 (1H, d, *J* = 8.0 Hz), 7.24 – 7.22 (2H, m), 7.19 – 7.14 (4H, m), 6.99 (1H, d, *J* = 7.3 Hz), 4.29 (2H, s), 2.46 (3H, s); ^{13}C NMR (151 MHz, $CDCl_3$) δ 151.0, 140.1, 139.0, 137.5, 128.8, 128.5, 127.1, 126.2, 122.8, 122.3, 113.0, 37.5, 14.8; HRMS (ESI): Found MH^+ 223.1224, $C_{15}H_{15}N_2$ requires 223.1230.

2-Methyl-1*H*-benzo[*d*]imidazol-6-ol 45a

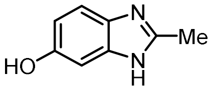

Following **GP11**, **45** (14.8 mg, 0.10 mmol) gave, after purification by column chromatography on silica gel eluting with EtOAc–MeOH (4:1), **45a** (48%, 7 mg) as a solid. R_f 0.47 [EtOAc:MeOH (4:1)]; ^{1}H NMR (600 MHz, DMSO-d_6) δ 9.00 (1H, s), 7.22 (1H, d, *J* = 8.5 Hz), 6.77 (1H, s), 6.59 (1H, dd, *J* = 8.5, 1.6 Hz), 2.41 (3H, s); ^{13}C NMR (151 MHz, DMSO-d_6) 152.8, 150.1, 138.4, 133.2, 115.1, 110.6, 98.6, 14.5; HRMS (EI): Found M^+ 148.0628, $C_8H_8N_2O$ requires 148.0637.

6-((*tert*-Butyldimethylsilyl)oxy)-2-methyl-1*H*-benzo[d]imidazole 46a

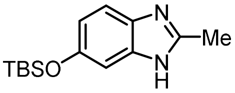

Following **GP11** at 0.025 M, **46** (26 mg, 0.10 mmol) gave, after purification by column chromatography on silica gel, eluting with pentane–EtOAc (4:1 to 0:1), **46a** (71%, 19 mg) as a solid. R_f 0.43 [EtOAc]; ^{1}H NMR ($CDCl_3$, 600 MHz) δ 7.36 (2H, d, *J* = 8.6 Hz), 6.97 (2H, s), 6.76 (1H, d, *J* = 8.6 Hz), 2.58 (3H, s), 0.99 (9H, s), 0.18 (6H, s); ^{13}C NMR (151 MHz, $CDCl_3$) δ 151.5, 150.8, 138.8, 134.3, 115.9, 115.0, 104.6, 25.8, 18.2, 15.0, -4.4; HRMS (ESI): Found MH^+ 263.1570, $C_{14}H_{23}ON_2Si$ requires 263.1574.

6-(Benzyloxy)-2-methyl-1*H*-benzo[*d*]imidazole 47a

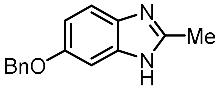

Following **GP11**, **47** (23.8 mg, 0.10 mmol) gave, after purification by column chromatography on silica gel eluting with EtOAc–MeOH (4:1), **47a** (44%, 10 mg) as a solid. R_f 0.33 [EtOAc:MeOH (4:1)]; ^{1}H NMR (600 MHz, $CDCl_3$) δ 7.44 (2H, d, *J* = 7.5 Hz), 7.41 (1H, d, *J* = 8.7 Hz), 7.37 (2H, t, *J* = 7.5 Hz), 7.31 (1H, t, *J* = 7.4 Hz), 7.06 (1H, d, *J* = 1.9 Hz), 6.93 (1H, dd, *J* = 8.7, 1.9 Hz), 5.08 (2H, s), 2.56 (3H, s); ^{13}C NMR (151 MHz, $CDCl_3$) δ 155.5, 150.8, 137.4, 128.7 (2C), 128.0, 127.7 (2C), 124.1, 120.5, 115.4, 112.4, 99.2, 71.0, 15.0; HRMS (EI): Found M^+ 238.1100, $C_{15}H_{14}N_2O$ requires 238.1106.

6-Butoxy-2-methyl-*1H*-benzo[*d*]imidazole 48a

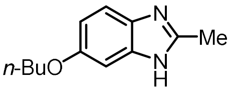

Following **GP11**, **48** (21.6 mg, 0.10 mmol) gave, after purification by column chromatography on silica gel eluting with EtOAc–MeOH (4:1), **48a** (64%, 13 mg) as a solid. R_f 0.33 [EtOAc:MeOH (4:1)]; ^{1}H NMR (400 MHz, $CDCl_3$) δ 7.40 (1H, d, *J* = 8.7 Hz), 7.00 (1H, d, *J* = 2.4 Hz), 6.85 (1H, dd, *J* = 8.7, 2.4 Hz), 3.95 (2H, t, *J* = 6.6 Hz), 2.58 (3H, s), 1.76 (2H, p, *J* = 6.6 Hz), 1.49 (2H, h, *J* = 7.4 Hz), 0.96 (3H, t, *J* = 7.4 Hz); ^{13}C NMR (101 MHz, $CDCl_3$) δ 155.7, 151.0, 139.0, 133.7, 115.3, 112.0, 98.6, 68.6, 31.6, 19.4, 15.0, 14.0; HRMS (ESI): Found MH^+ 205.1334, $C_{12}H_{17}N_2O$ requires 205.1336.

6-(Cyclopentyloxy)-2-methyl-1*H*-benzo[*d*]imidazole 49a

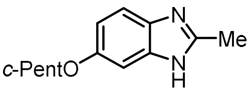

Following **GP11**, **49** (21.6 mg, 0.10 mmol) gave, after purification by column chromatography on silica gel eluting with EtOAc–MeOH (4:1), **49a** (65%, 14 mg) as a solid. R_f 0.33 [EtOAc:MeOH (4:1)]; ^{1}H NMR (400 MHz, $CDCl_3$) δ 7.39 (1H, d, *J* = 8.7 Hz), 6.99 (1H, d, *J* = 2.3 Hz), 6.81 (1H, dd, *J* = 8.7, 2.3 Hz), 4.74 (1H, p, *J* = 4.3 Hz), 2.57 (3H, s), 1.90 – 1.83 (4H, m), 1.83 – 1.74 (m, 2H), 1.65 – 1.56 (m, 2H); ^{13}C NMR (101 MHz, $CDCl_3$) δ 154.6, 150.8, 139.0, 133.5, 115.2, 113.0, 100.0, 80.2, 32.9 (2C), 24.2 (2C), 15.1; HRMS (ESI): Found MH^+ 217.1334, $C_{13}H_{17}N_2O$ requires 217.1336.

10.Permutation products – 2*H*-indazoles

1-Methyl-1*H*-benzo[*d*]imidazole 53a

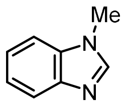

Following **GP11**, **53** (13.2 mg, 0.10 mmol) gave, after purification by column chromatography on silica gel eluting with EtOAc–MeOH (9:1), **53a** (96%, 13 mg) as an oil. R_f 0.47 [EtOAc:MeOH (9:1)]; ^{1}H NMR (600 MHz, $CDCl_3$) δ 7.86 (1H, s), 7.81 (1H, d, *J* = 7.2 Hz), 7.39 (1H, d, *J* = 8.0 Hz), 7.32 (1H, t, *J* = 7.5 Hz), 7.29 (1H, t, *J* = 7.5 Hz), 3.84 (3H, s); ^{13}C NMR (151 MHz, $CDCl_3$) δ 143.9, 143.6, 134.7, 123.1, 122.2, 120.4, 109.4, 31.1; HRMS (EI): Found M^+ 132.0679, $C_8H_8N_2$ requires 132.0687. Data in accordance with literature.[37]

1,2-Dimethyl-1*H*-benzo[*d*]imidazole 54a

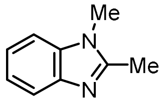

Following **GP11**, **54** (14.6 mg, 0.10 mmol) gave, after purification by column chromatography on silica gel eluting with EtOAc–MeOH (9:1), **54a** (75%, 11 mg) as a solid. R_f 0.26 [EtOAc:MeOH (9:1)]; ^{1}H NMR (600 MHz, $CDCl_3$) δ 7.72 – 7.69 (1H, m), 7.31 – 7.22 (3H, m), 3.72 (3H, s), 2.61 (3H, s); ^{13}C NMR (151 MHz, $CDCl_3$) δ 151.9, 142.6, 135.9, 122.1, 121.9, 119.1, 108.9, 29.9, 13.9; HRMS (EI): Found M^+ 146.0835, $C_9H_{10}N_2$ requires 146.0844. Data in accordance with literature.[38]

5-Methoxy-1-methyl-1*H*-benzo[*d*]imidazole 55a

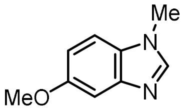

Following **GP11**, **55** (14.6 mg, 0.10 mmol) gave, after purification by column chromatography on silica gel eluting with EtOAc–MeOH (9:1), **55a** (87%, 14 mg) as a solid. R_f 0.35 [EtOAc:MeOH (9:1)]; ^{1}H NMR (600 MHz, $CDCl_3$) δ 7.78 (1H, s), 7.27 – 7.22 (2H, m), 6.95 (1H, dd, *J* = 8.8, 2.3 Hz), 3.85 (3H, s), 3.78 (3H, s); 13C NMR (151 MHz, $CDCl_3$) δ 156.3, 144.6, 143.8, 129.4, 113.3, 109.8, 102.3, 55.9, 31.2; HRMS (EI): Found M^+ 162.0788, $C_9H_{10}N_2O$ requires 162.0793. Data in accordance with literature.[39]

1,6-Dimethyl-1*H*-benzo[*d*]imidazole 56a

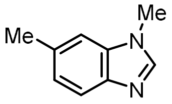

Following **GP11**, **56** (14.6 mg, 0.10 mmol) gave, after purification by column chromatography on silica gel eluting with EtOAc–MeOH (9:1), **56a** (60%, 9 mg) as a solid. R_f 0.43 [EtOAc:MeOH (9:1)]; ^{1}H NMR (400 MHz, $CDCl_3$) δ 7.79 (1H, s), 7.68 (1H, d, *J* = 8.1 Hz), 7.18 (1H, s), 7.11 (1H, d, *J* = 8.1 Hz), 3.80 (3H, s), 2.51 (3H, s); ^{13}C NMR (101 MHz, $CDCl_3$) δ 143.4, 142.0, 135.0, 133.1, 123.8, 119.9, 109.4, 31.1, 21.9; HRMS (EI): Found M^+ 146.0836, $C_9H_{10}N_2$ requires 146.0844. Data in accordance with literature.[40]

1,7-Dimethyl-1*H*-benzo[*d*]imidazole 57a

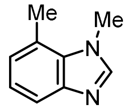

Following **GP11**, **57** (15 mg, 0.10 mmol) gave, after purification by column chromatography on silica gel, eluting with EtOAc–MeOH (1:0 to 9:1), **57a** (73%, 11 mg) as an oil. R_f 0.22 [EtOAc]; ^{1}H NMR ($CDCl_3$, 400 MHz) δ 7.75 (1H, s), 7.61 (1H, d, *J* = 8.1 Hz), 7.13 (1H, t, *J* = 7.9 Hz), 7.00 (1H, d, *J* = 7.3 Hz), 4.04 (3H, s), 2.73 (3H, s); ^{13}C NMR (101 MHz, $CDCl_3$) δ 144.6, 144.5, 133.3, 125.2, 122.4, 121.6, 118.4, 34.2, 18.5; HRMS (EI): Found M^+ 146.0839, $C_9H_{10}N_2$ requires 146.0844. Data in accordance with literature.[41]

1-(4-Methylbenzyl)-1*H*-benzo[*d*]imidazole 58a

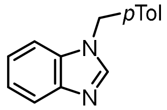

Following **GP11**, **58** (22.2 mg, 0.10 mmol) gave, after purification by column chromatography on silica gel eluting with EtOAc–MeOH (9:1), **58a** (93%, 21 mg) as a solid. R_f 0.41 [EtOAc:MeOH (9:1)]; ^{1}H NMR (400 MHz, $CDCl_3$) δ 7.92 (1H, s), 7.85 – 7.80 (1H, m), 7.33 – 7.22 (3H, m), 7.14 (2H, d, *J* = 7.9 Hz), 7.08 (2H, d, *J* = 7.9 Hz), 5.30 (2H, s), 2.33 (3H, s); ^{13}C NMR (101 MHz, $CDCl_3$) δ 144.1, 143.3, 138.2, 134.0, 132.5, 129.8 (2C), 127.3 (2C), 123.1, 122.3, 120.5, 110.2, 48.8, 21.2; HRMS (EI): Found MH^+ 222.1145, $C_{15}H_{14}N_2$ requires 222.1157. Data in accordance with literature.[42]

1-Cyclohexyl-1*H*-benzo[*d*]imidazole 59a

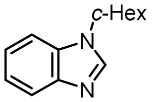

Following **GP11**, **59** (20 mg, 0.10 mmol) gave, after purification by column chromatography on silica gel eluting with EtOAc–MeOH (9:1), **59a** (95%, 19 mg) as a solid. R_f 0.44 [EtOAc:MeOH (9:1)]; ^{1}H NMR (400 MHz, $CDCl_3$) δ 7.99 (1H, s), 7.84 – 7.77 (1H, m), 7.42 (1H, d, *J* = 6.7 Hz), 7.31 – 7.23 (2H, m), 4.19 (1H, tt, *J* = 12.0, 3.8 Hz), 2.22 (2H, d, *J* = 13.3 Hz), 1.97 (2H, d, *J* = 13.8 Hz), 1.86 – 1.74 (3H, m), 1.58 – 1.43 (2H, m), 1.41 – 1.23 (2H, m); ^{13}C NMR (101 MHz, $CDCl_3$) δ 144.0, 140.5, 133.5, 122.6, 122.1, 120.5, 110.2, 55.5, 33.4 (2C), 25.8 (2C), 25.5; HRMS (EI): Found M^+ 200.1307, $C_{13}H_{16}N_2$ requires 200.1313. Data in accordance with literature.[43]

2-(1*H*-Benzo[*d*]imidazol-1-yl)-2-methylpropan-1-ol 60a

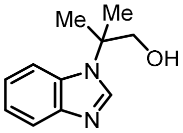

Following **GP11**, **60** (19 mg, 0.10 mmol) gave, after purification by column chromatography on silica gel eluting with EtOAc–MeOH (9:1), **60a** (48%, 9 mg) as an oil. R_f 0.42 [EtOAc:MeOH (9:1)]; ^{1}H NMR (400 MHz, $CDCl_3$) δ 7.76 (1H, s), 7.60 – 7.53 (2H, m), 7.19 (2H, td, *J* = 7.2, 1.5 Hz), 7.15 (2H, td, *J* = 7.2, 1.2 Hz), 3.98 (2H, s), 1.73 (6H, s); ^{13}C NMR (101 MHz, $CDCl_3$) δ 144.6, 141.9, 132.6, 122.5, 122.1, 120.4, 112.9, 68.1, 60.7, 24.7 (2C); HRMS (EI): Found M^+ 190.1100, $C_{11}H_{14}N_2O$ requires 190.1106.

(*R*)-1-(1-Phenylethyl)-1*H*-benzo[*d*]imidazole 61a

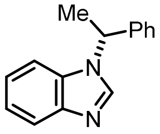

Following **GP11**, **61** (22.2 mg, 0.10 mmol) gave, after purification by column chromatography on silica gel eluting with pentane–EtOAc (1:9), **61a** (48%, 11 mg) as a solid. R_f 0.26 [pentane–EtOAc (1:9)]; ^{1}H NMR (400 MHz, $CDCl_3$) δ 8.08 (1H, s), 7.82 (1H, d, *J* = 7.9 Hz), 7.37 – 7.22 (4H, m), 7.21 – 7.16 (4H, m), 5.62 (1H, q, *J* = 7.1 Hz), 2.00 (3H, d, *J* = 7.1 Hz); ^{13}C NMR (101 MHz, $CDCl_3$) δ 144.2, 141.1, 140.8, 133.8, 129.1 (2C), 128.2, 126.1 (2C), 123.0, 122.4, 120.5,

110.7, 55.4, 21.7; HRMS (EI): Found M^+ 222.1152, $C_{15}H_{14}N_2$ requires 222.1157. Data in accordance with literature.[44]

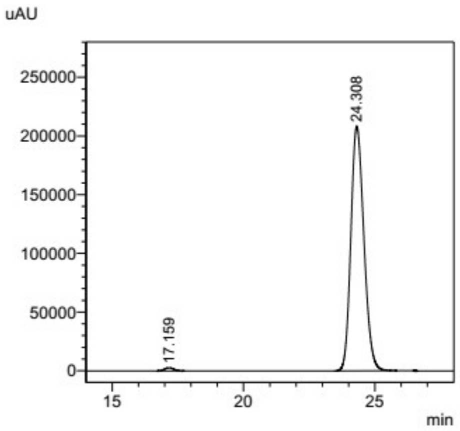

PDA Ch1 254nm

Peak#	Ret. Time	Area	Area%
1	17.159	63333	0.873
2	24.308	7189099	99.127
Total		7252432	100.000

Figure S3. Chromatogram of **61a** - HPLC (CHIRALCEL® OD-H).

1-(1-Phenylethyl)-1*H*-benzo[*d*]imidazole 61a-rac

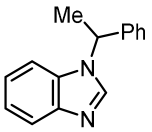

Following **GP11**, **61-rac** (22.2 mg, 0.10 mmol) gave, after purification by column chromatography on silica gel eluting with pentane–EtOAc (1:9), **61a-rac** (47%, 10 mg) as a solid. R_f 0.26 [pentane–EtOAc (1:9)]; ^{1}H NMR (400 MHz, $CDCl_3$) δ 8.08 (1H, s), 7.82 (1H, d, *J* = 8.0 Hz), 7.36 – 7.22 (4H, m), 7.22 – 7.16 (4H, m), 5.62 (1H, q, *J* = 7.2 Hz), 2.00 (3H, d, *J* = 7.2 Hz); ^{13}C NMR (101 MHz, $CDCl_3$) δ 144.3, 141.1, 140.8, 133.8, 129.1, 128.2, 126.1, 123.0, 122.4, 120.5, 110.8, 55.4, 21.7; HRMS (EI): Found M^+ 222.1154, $C_{15}H_{14}N_2$ requires 222.1157. Data in accordance with literature.[45]

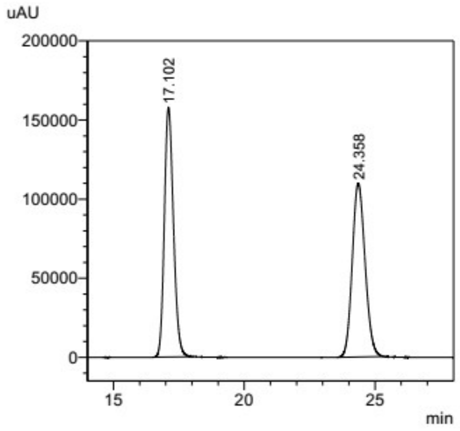

PDA Ch1 254nm

Peak#	Ret. Time	Area	Area%
1	17.102	3817251	50.496
2	24.358	3742197	49.504
Total		7559448	100.000

Figure S4. Chromatogram of **61a-rac** - HPLC (CHIRALCEL® OD-H).

(*R*)-1-(2,3-Dihydro-1*H*-inden-1-yl)-1*H*-benzo[*d*]imidazole 62a

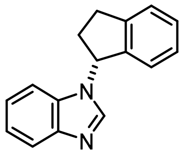

Following **GP11**, **62** (23.4 mg, 0.10 mmol) gave, after purification by column chromatography on silica gel eluting with EtOAc–MeOH (9:1), **62a** (79%, 19 mg) as an oil. R_f 0.36 [EtOAc:MeOH (9:1)]; ^{1}H NMR (400 MHz, $CDCl_3$) δ 7.82 (1H, d, J = 7.9 Hz), 7.76 (1H, s), 7.41 (1H, d, J = 7.5 Hz), 7.36 (1H, t, J = 7.5 Hz), 7.31 – 7.17 (4H, m), 7.13 (1H, d, J = 7.5 Hz), 5.98 (1H, t, J = 7.2 Hz), 3.25 – 3.14 (1H, m), 3.07 (1H, dt, J = 16.1, 7.8 Hz), 2.76 (1H, dtd, J = 13.1, 8.1, 4.7 Hz), 2.36 (1H, ddt, J = 13.6, 9.0, 6.9 Hz); ^{13}C NMR (101 MHz, $CDCl_3$) δ 144.5, 143.8, 142.2, 139.8, 133.5, 129.2, 127.4, 125.5, 125.0, 122.8, 122.3, 120.6, 110.6, 61.0, 33.5, 30.6; HRMS (EI): Found M^+ 234.1150, $C_{16}H_{14}N_2$ requires 234.1157.

***tert*-Butyl 2-((4R,6R)-6-(2-(1*H*-benzo[*d*]imidazol-1-yl)ethyl)-2,2-dimethyl-1,3-dioxan-4-yl)acetate 63a**

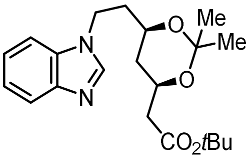

Following **GP11**, **63** (37.4 mg, 0.10 mmol) gave, after purification by column chromatography on silica gel eluting with EtOAc–MeOH (9:1), **63a** (91%, 34 mg) as an oil. R_f 0.38 [EtOAc:MeOH (9:1)]; ^{1}H NMR (600 MHz, $CDCl_3$) δ ^{1}H NMR ($CDCl_3$, 400 MHz) δ 7.83 (1H,

s), 7.80 – 7.76 (1H, m), 7.40 – 7.35 (1H, m), 7.30 – 7.22 (2H, m), 4.35 (1H, ddd, *J* = 14.4, 9.6, 6.1 Hz), 4.22 (1H, ddd, *J* = 14.4, 6.6, 4.1 Hz), 4.15 – 4.07 (m, 1H,), 3.68 – 3.58 (1H, m), 2.37 (1H, dd, *J* = 15.1, 6.9 Hz), 2.23 (1H, dd, *J* = 15.1, 6.2 Hz), 2.04 – 1.95 (1H, m), 1.93 – 1.83 (m, 1H), 1.39 (12H, s), 1.33 (3H, s), 1.26 – 1.14 (2H, m); ^{13}C NMR (101 MHz, $CDCl_3$) δ 170.1, 144.0, 143.4, 133.8, 122.9, 122.2, 120.5, 109.8, 99.1, 80.8, 66.1, 65.1, 42.7, 40.6, 36.4, 36.1, 30.2, 28.2 (3C), 19.8; HRMS (EI): Found M^+ 374.2200, $C_{21}H_{30}N_2O_4$ requires 374.2206.

11.Mechanistic studies

a. UV-vis titration with HFIP

To study the effect of HFIP on the tautomerization of indazole **1** we measured the UV-Vis spectra in dichloromethane (0.1 mM), and with addition of HFIP. Spectra were obtained using a Shimadzu (UV-2600) UV-vis spectrophotometer and a 1 cm quartz cuvette "QS" from Hellma Analytics. From previous studies indazoles such as **1** have been shown to sit predominantly in the *1H*-indazole form as determined by UV-vis experiments.[46] Our UV-Vis studies showed no difference in absorbance spectra when HFIP was added, which suggests that tautomerization does not occur significantly in the ground state. This is line with our calculations that show the HFIP-mediated tautomerization to occur at the excited state.

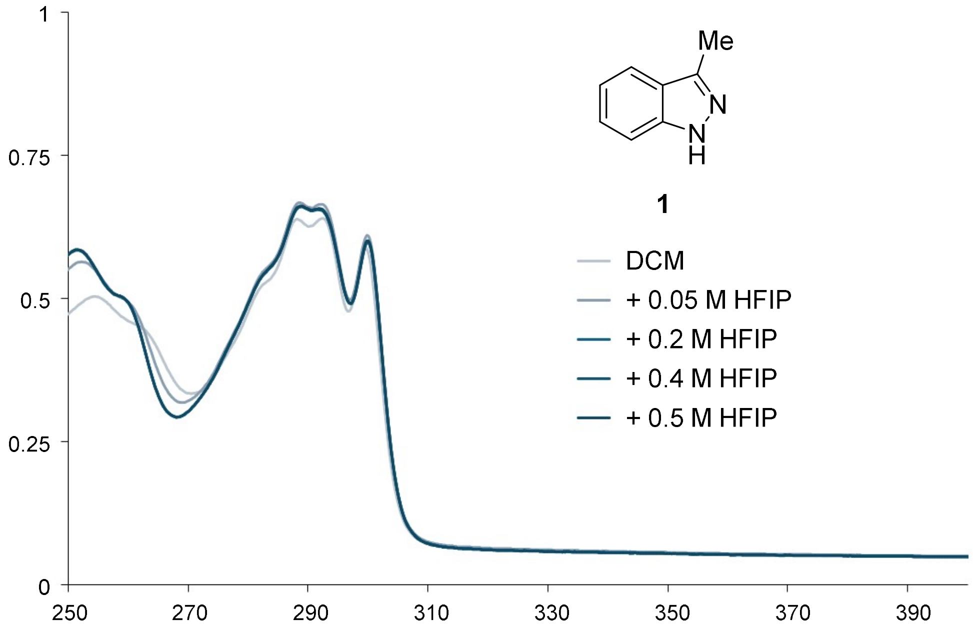

b. HFIP/CH_3CN mixtures

We also studied the effect of solvent mixtures on the photoisomerization of indazole **1**. We observed modest reaction to afford benzimidazole **1a** with HFIP/CH_3CN solvent mixtures, but noted a marked decrease in yield with decreasing amounts of HFIP. This suggests the non-innocent role of HFIP for the reaction.

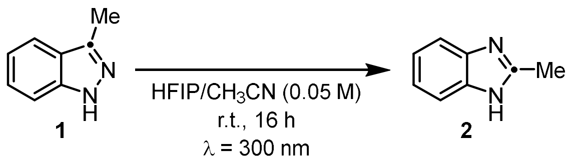

**Entry**	**HFIP/CH_3CN Ratio**	**Yield**
**1**	3:1	**50%**
**2**	1:1	**23%**
**3**	1:3	**17%**
**4**	1:9	**16%**

12.Computational studies

To accurately model excited states, the multiconfigurational CASSCF (complete active space self-consistent field) method was employed using OpenMolcas software.[47] The active spaces (electrons,orbitals) in the CASSCF calculations were selected from the highest-lying p molecular orbitals and the nitrogen's lone pair (n) orbitals. For the indazole molecules, an active space of 12 electrons distributed over 10 orbitals (12,10) was found necessary for an accurate representation. Throughout the CASSCF calculations, the cc-pVDZ[48] basis set was utilized. Ground state geometries and conical intersection (CX) points were optimized at the CAS(12,10)/cc-pVDZ level of theory using the OpenMolcas program. All CASSCF calculations were performed using the state-averaged formalism.

To compare the ground state and excited state energies of **1** + HFIP and **1b** + HFIP systems, geometry optimizations were performed in the gas phase for both states using CAM-B3LYP[49] functional and cc-pVDZ basis set with the ORCA 5.0.4 software. The same theoretical level was applied for vibrational frequency calculations to confirm that no imaginary frequencies were present in the Hessian matrix. Further single-point calculations were using the cc-pVTZ basis set.[48] Solvent effects were included using the implicit universal solvation model (SMD).[50] As ORCA 5.0.4 does not provide specific solvent parameters for HFIP, the parametres for 2-propanol were employed, with the dielectric constant adjusted to match that of HFIP ($\varepsilon = 16.7$). To compare the ground state and excited state basicity of **1** and **1b**, geometry optimizations and frequency calculations were performed for both neutral and protonated forms of these molecules at CAM-B3LYP/cc-pVDZ and TDCAM-B3LYP/cc-pVDZ level of theory. Additionally, single point energy calculations were carried out at the CAM-B3LYP/cc-pVTZ/SMD(HFIP) and TDCAM-B3LYP/cc-pVDZ level of theories.

To investigate the proton transfer pathway from HFIP to **1**, constrained optimization calculations were performed in the excited state at TDCAM-B3LYP/cc-pVDZ level of theory, with the distance between N^2 atom of 1*H*-indazole and the H atom of HFIP fixed at specific intervals. Single-point energy calculations were subsequently carried out at TDCAM-B3LYP/cc-pVTZ/SMD(HFIP) level of theory.

For the reaction path, Density Functional Theory (DFT) calculations were performed with the Gaussian16 C.01 software package.[51] Geometry optimizations and frequency calculations were carried out using the unrestricted formalism of the ωB97XD[52] functional in combination with the def2-TZVP[48d] basis set, as implemented in Gaussian16. The same level of theory was

employed for vibrational frequency calculations to confirm that no negative eigenvalues in the Hessian matrix, with only one negative eigenvalue for transition states. No symmetry restrictions were applied. Further single-point calculations utilized the cc-pVQZ basis set.[48] Solvent effects were incorporated into the geometry optimizations through the implicit universal solvation model (SMD). Since Gaussian16 lacks specific solvent parameters for HFIP, the parameters of 2-propanol were used, with the dielectric constant adjusted to match that of HFIP (ε = 16.7) for the reaction path of **1b**. The default parameters for acetonitrile (CH_3CN) were applied for the mechanism of **53**. Thermodynamic data were obtained via a quasi-harmonic correction to entropy using the rigid rotor/harmonic oscillator (RRHO) approximation,[53] where vibrational modes below 100 cm^{-1} were treated with a free rotor approximation interpolated with a damping function. These thermodynamic corrections were applied using *Goodvibes.py*,[54] assuming a solution-phase standard state (T = 298.15 K, c = 1 mol^{-1}). All energy values are reported in kcal mol^{-1}. Cartesian coordinated and thermodynamic data are provided in the electronic supplementary materials.

Excited State Calculations of Indazoles

For **1**, **1b**, and **53** the conical intersection points were systematically optimized at SA-CAS(12,10)/cc-pVDZ level of theory. Upon photoexcitation, **1** populates the $\pi\pi^*$ singlet excited state. The two conical intersection points (**CX1** and **CX1'**) identified, which have the potential to form the Dewar intermediate (Table S1), are significantly higher in energy compared to the Frank-Condon (FC) region (Figure S5). **1b** and **53** also populate the $\pi\pi^*$ singlet excited state after photoexcitation. The conical intersections (**CX2** and **CX4**, respectively) leading to the formation of Dewar intermediate (Table S1) are lower in energy than the FC region and therefore accessible (Figure S5).

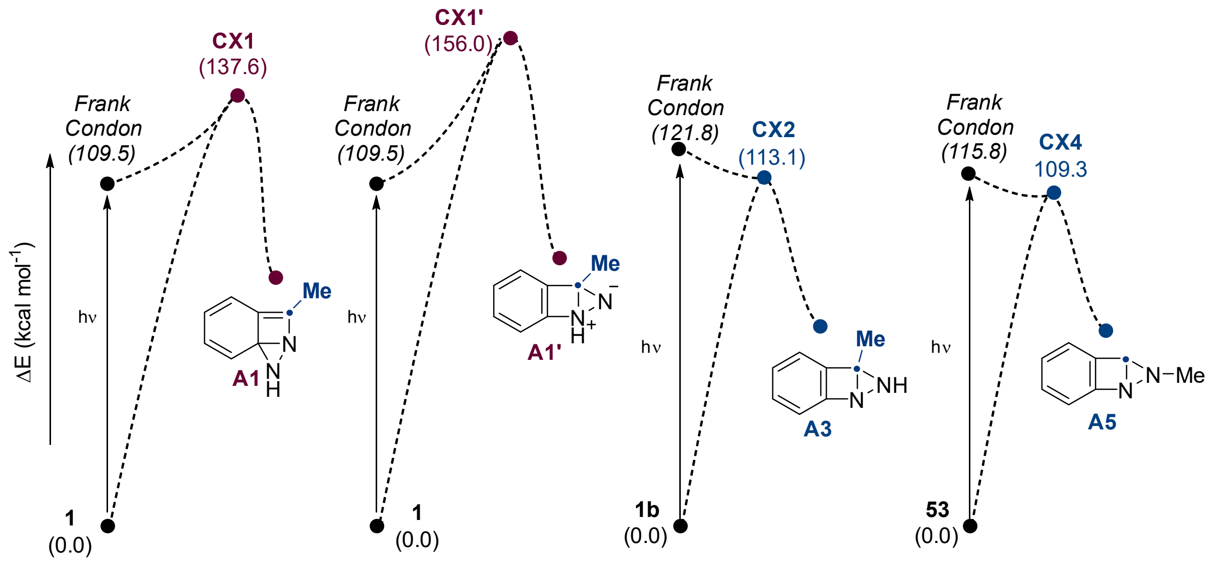

Figure S5. Schematic indicating the energies of **1**, **CX1**, **CX1'**, **1b**, **CX2**, **53** and **CX4** at SA-CAS(12,10)/cc-pVDZ level of theory (kcal mol^{-1}).

Table S1. Selected geometric parameters of **1@S$_0$**, **1@CX1**, **1@CX1'**, **1b@S$_0$**, **1b@CX2**, **53@S$_0$** and **53@CX4** at SA-CAS(12,10)/cc-pVDZ level of theory.

	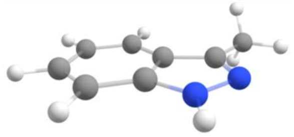 **1@S$_0$**	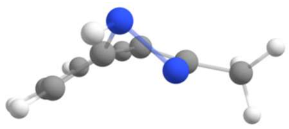 **1@CX1**
**R(N$_1$–C$_{7a}$)**	2.141 Å	1.875 Å
**α(N$_1$–N$_2$–C$_{7a}$)**	107.2°	80.3°
	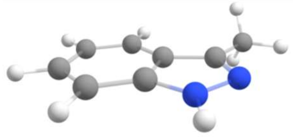 **1@S$_0$**	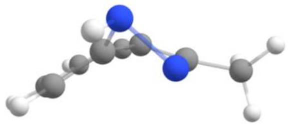 **1@CX1'**
**R(N$_1$–C$_3$)**	2.141 Å	1.866 Å
**α(N$_1$–N$_2$–C$_3$)**	107.2°	81.9°
	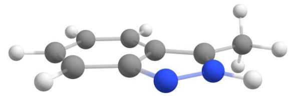 **1b@S$_0$**	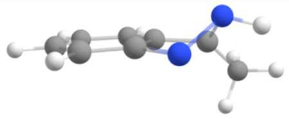 **1b@CX2**
**R(N$_1$–C$_3$)**	2.266 Å	2.084 Å
**α(N$_1$–N$_2$–C$_3$)**	115.6°	89.5°
	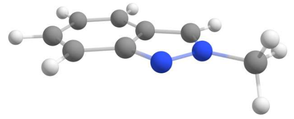 **53@S$_0$**	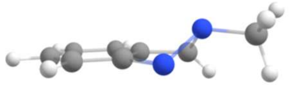 **53@CX4**
**R(N$_1$–C$_3$)**	2.249 Å	2.086 Å
**α(N$_1$–N$_2$–C$_3$)**	114.2°	88.9°

The ground state and excited state energies and basicity of **1 + HFIP** and **1b + HFIP** systems were compared. In the ground state, **1 + HFIP** is more stable, while in the excited state, **1b + HFIP** is more stable (Figure S6). Regarding basicity, **1b** exhibits higher basicity in the ground state, whereas **1** shows greater basicity in the excited state.

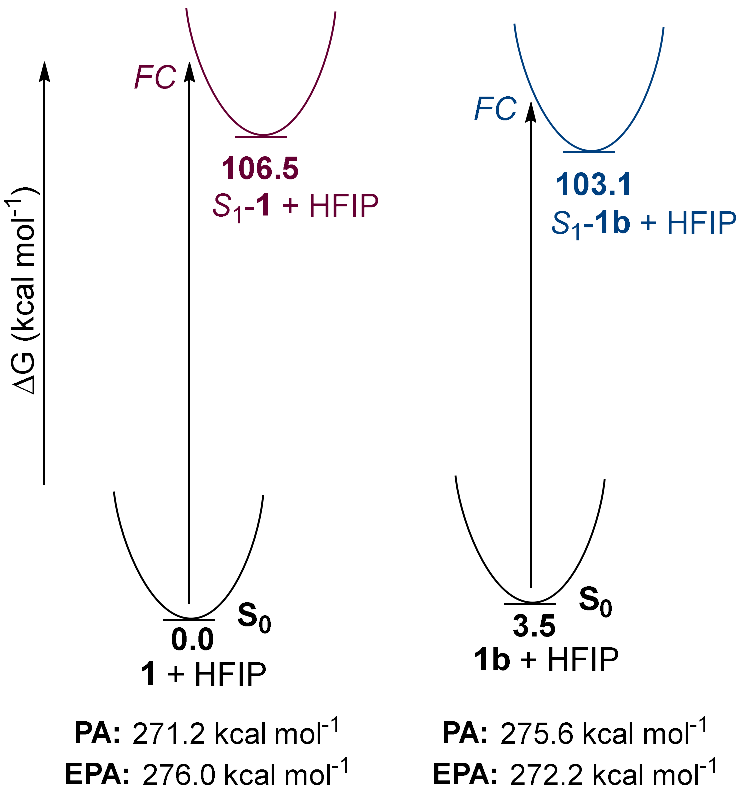

Figure S6. The ground state and excited state energies and basicity of **1** + HFIP and **1b** + HFIP systems at CAM-B3LYP/cc-pVTZ/SMD(HFIP)//cc-pVDZ(gas) and TDCAM-B3LYP/cc-pVTZ/SMD(HFIP)//cc-pVDZ(gas)

Proton Transfer Path

To explore the proton transfer pathway from HFIP to **1**, constrained optimizations were performed in the excited state by varying the distance (R_{N-H}) between the N^2 atom of **1** and the H atom of HFIP (Figure S7). Along this pathway, the maximum energy point was identified at R = 1.16 Å, with an energy barrier of 9.5 kcal mol^{-1}.

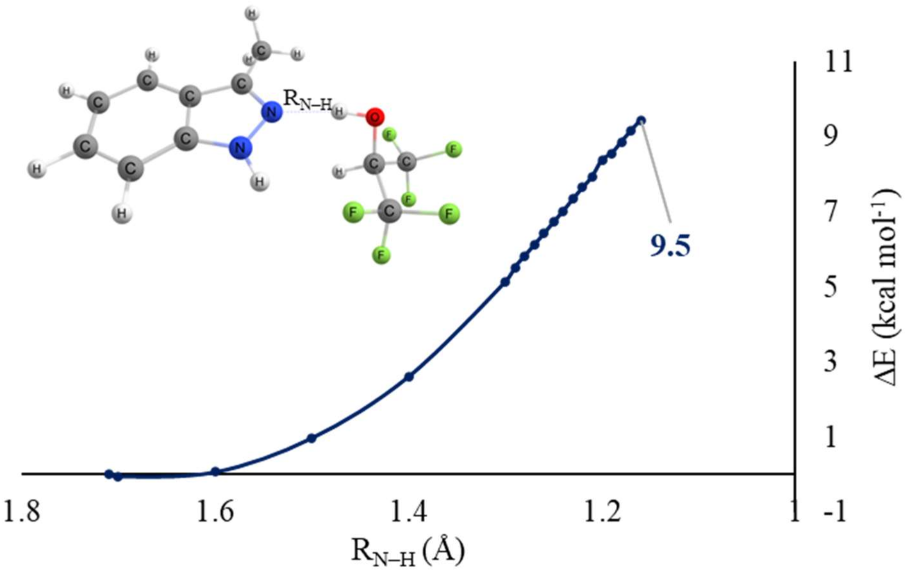

Figure S7. Excited state proton transfer pathway from HFIP to **1** at TDCAM-B3LYP/cc-pVTZ/SMD(HFIP)//cc-pVDZ(gas)

Reaction Mechanism

The Dewar intermediate (**A3**) undergoes homolytic cleavage of the N–N bond with a barrier of 19.1 kcal mol^{-1}, leading to the formation of a biradical intermediate (**A4**). This biradical intermediate then transforms into another Dewar intermediate, **A2**, with a barrier of 5 kcal mol^{-1}. The Dewar intermediate **A2** undergoes a barrierless 4π electrocyclic ring-opening, resulting in the formation of the final product (**1a**).

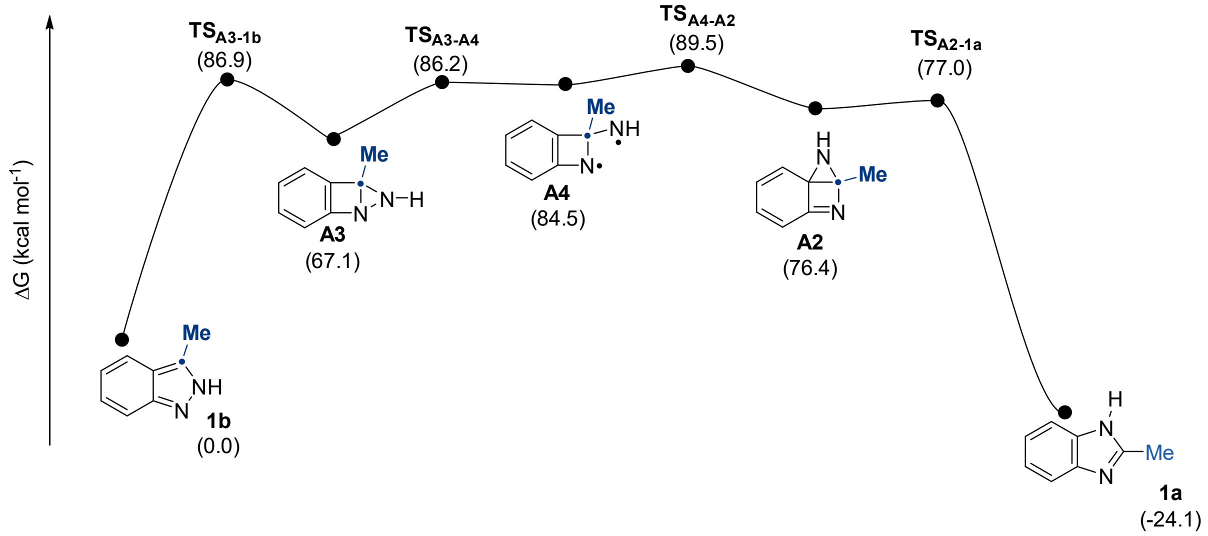

Figure S8. Computed reaction energy profile of **1b**. The numbers given brackets are the relative Gibbs free energies at (U)ωB97XD/cc-pVQZ/SMD(HFIP)//def2-TZVP/SMD(HFIP) level of theory (kcal mol^{-1})

The Dewar intermediate (**A5**) undergoes homolytic cleavage of the N–N bond, overcoming a barrier of 16.1 kcal mol^{-1}, which leads the formation of a biradical intermediate (**A6**). This biradical intermediate subsequently converts into another Dewar intermediate, **A7**, requiring a barrier of 11.4 kcal mol^{-1}. Finally, **A7** undergoes a barrierless 4π electrocyclic ring-opening, leading to the formation of the final product (**53a**).

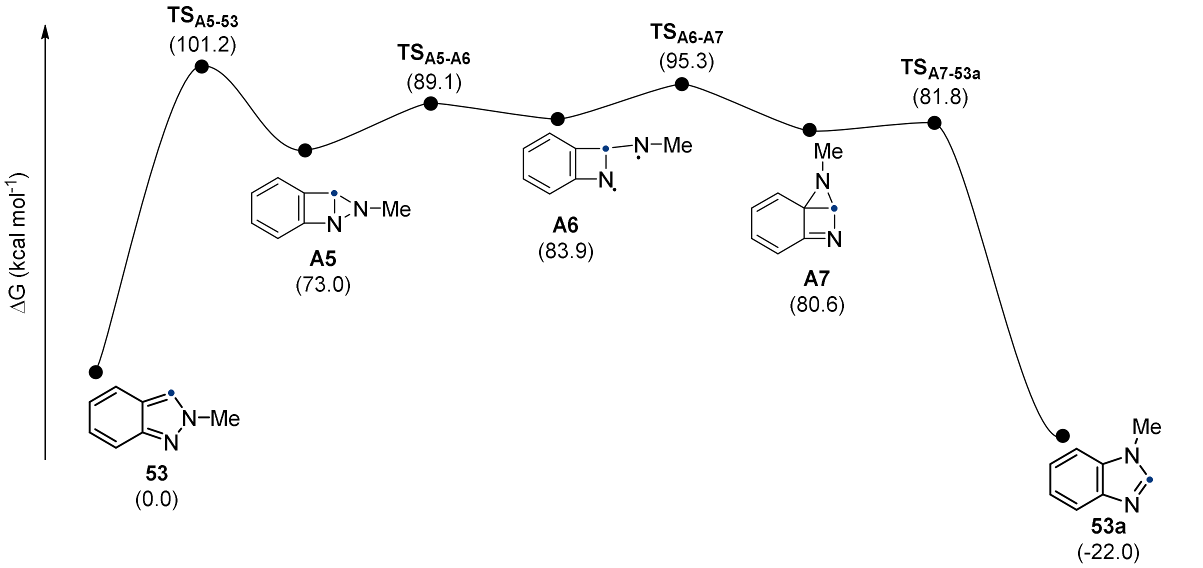

Figure S9. Computed reaction energy profile of **53**. The numbers given brackets are the relative Gibbs free energies at (U)ωB97XD/cc-pVQZ/SMD(CH_3CN)//def2-TZVP/SMD(CH_3CN) level of theory (kcal mol^{-1}).

Excited State Behaviour of Benzimidazoles

Conical intersection points for **1a** and **53a** were systematically optimized at SA-CAS(12,10/cc-pVDZ level of theory to investigate the possibility of the benzimidazoles after their formation.

Upon photoexcitation **1a** and **53a** populate the ππ* singlet excited state. Conical intersection points (**CX3** and **CX5**) with the potential to form the Dewar intermediate were identified (Figure S10). However, the energies of these points are higher than that of the Frank-Condon region, making them inaccessible.

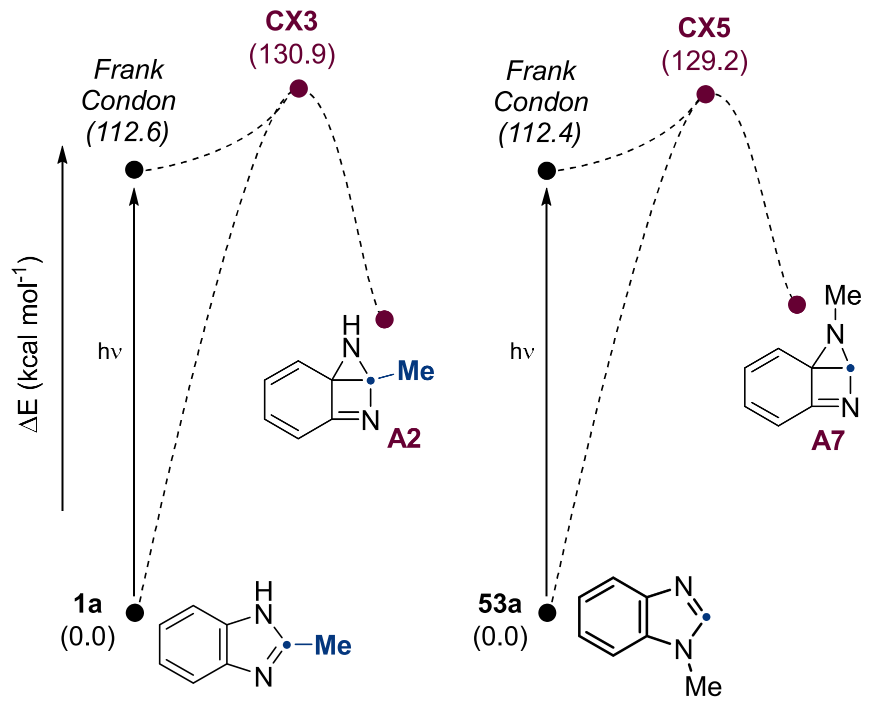

Figure S10. Schematic indicating the energies of **1a**, **CX3**, **53a** and **CX5** at SA-CAS(12,10)/cc-pVDZ level of theory (kcal mol^{-1}).

Table S2. Selected geometric parameters of **1a@S$_0$**, **1a@CX3**, **53a@S$_0$** and **53a@CX5** at SA-CAS(12,10)/cc-pVDZ level of theory.

	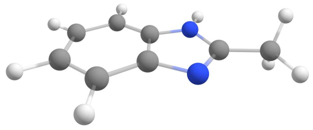 **1a@S$_0$**	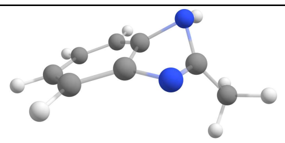 **1a@CX3**
**R(C$_2$–C$_9$)**	2.215 Å	1.845 Å
**α(C$_2$–N$_3$–C$_9$)**	107.0°	79.4°
	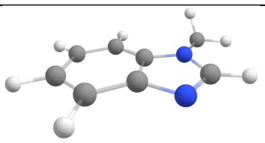 **53a@S$_0$**	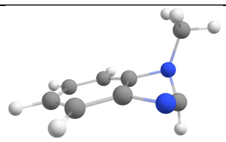 **53a@CX5**
**R(C$_2$–C$_9$)**	2.191 Å	1.911 Å
**α(C$_2$–N$_3$–C$_9$)**	105.7°	83.6°

13. NMR Spectra

2’ – ^{1}H NMR (400 MHz, $CDCl_3$)

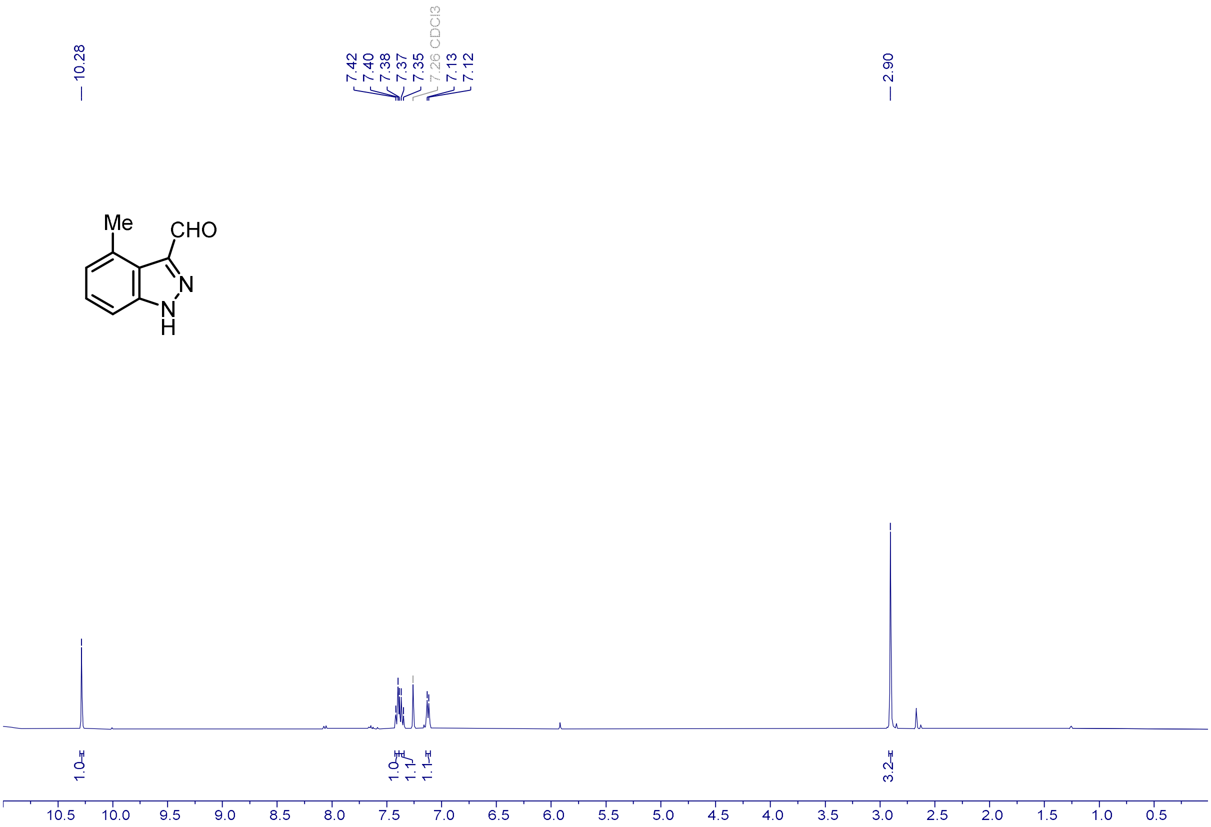

2’ – ^{13}C NMR (101 MHz, $CDCl_3$)

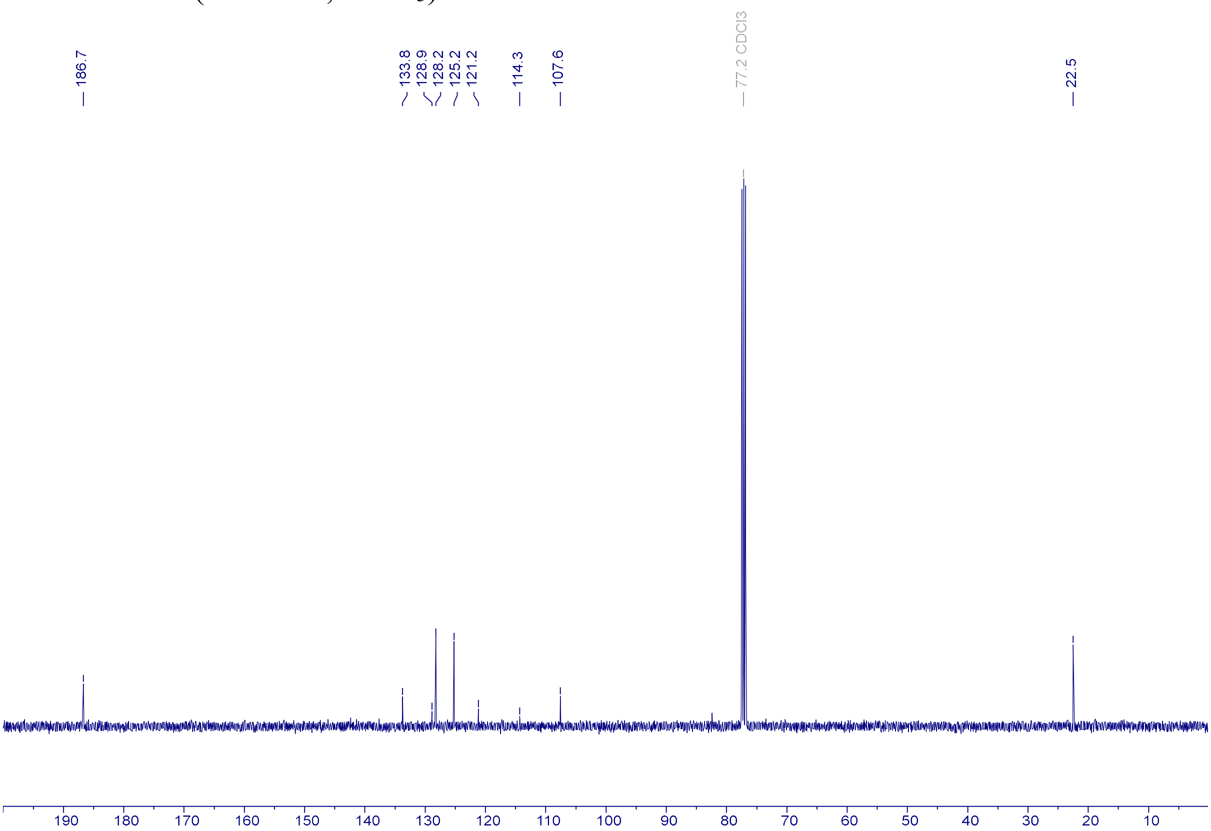

2 – ^{1}H NMR (600 MHz, $CDCl_3$)

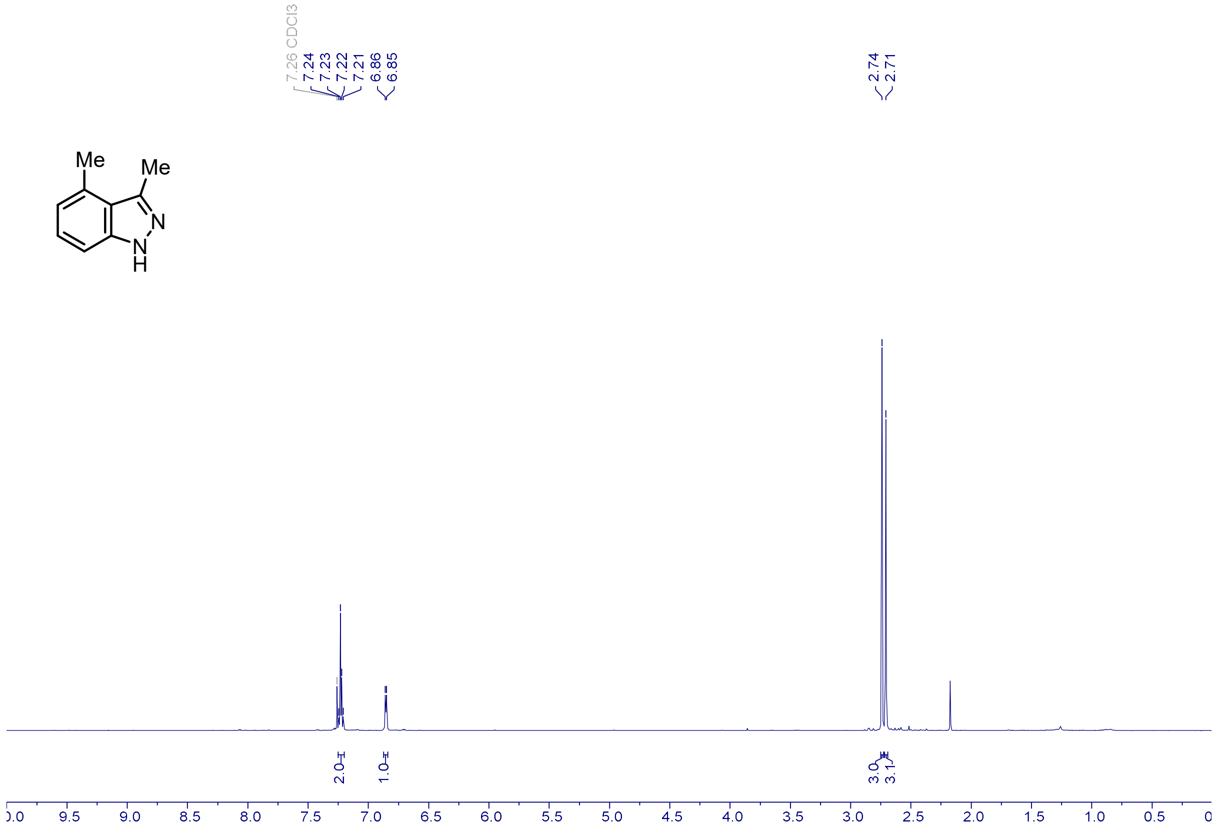

2 – ^{13}C NMR (151 MHz, $CDCl_3$)

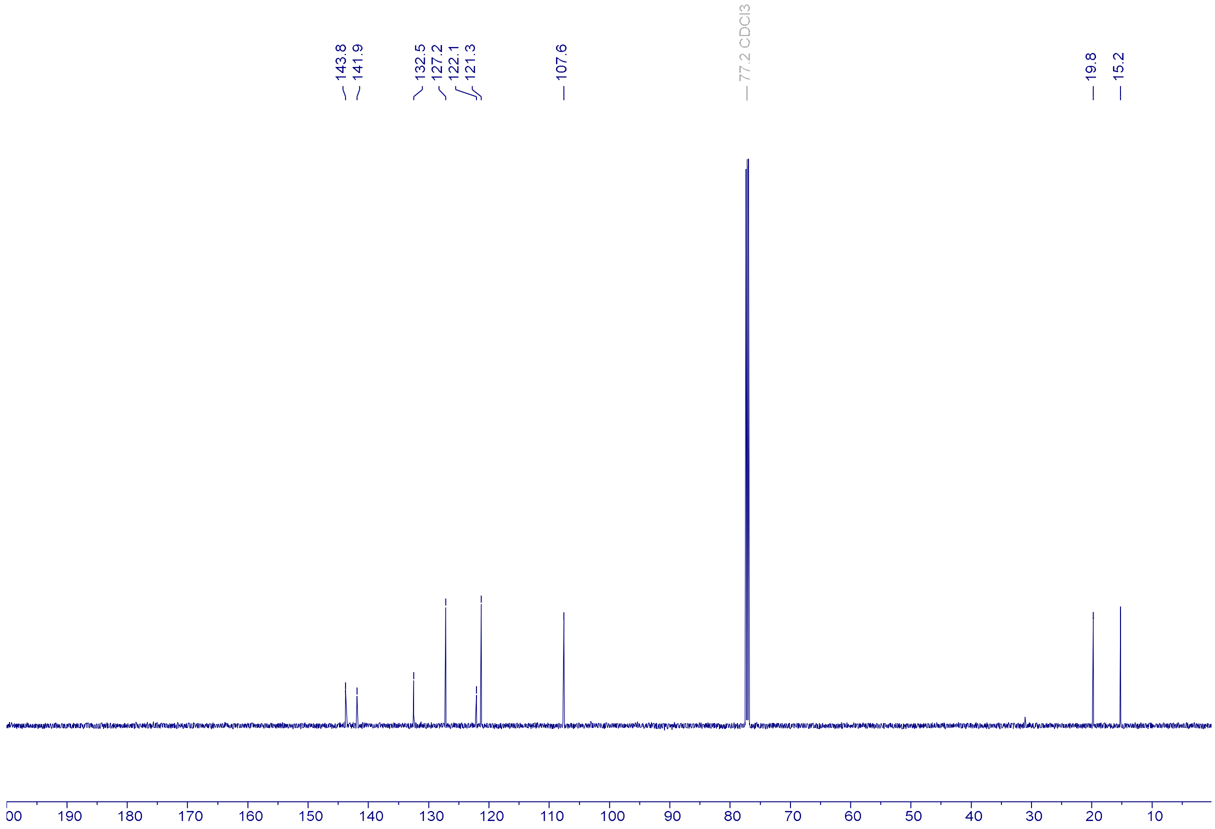

3 – ^{1}H NMR (600 MHz, $CDCl_3$)

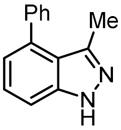

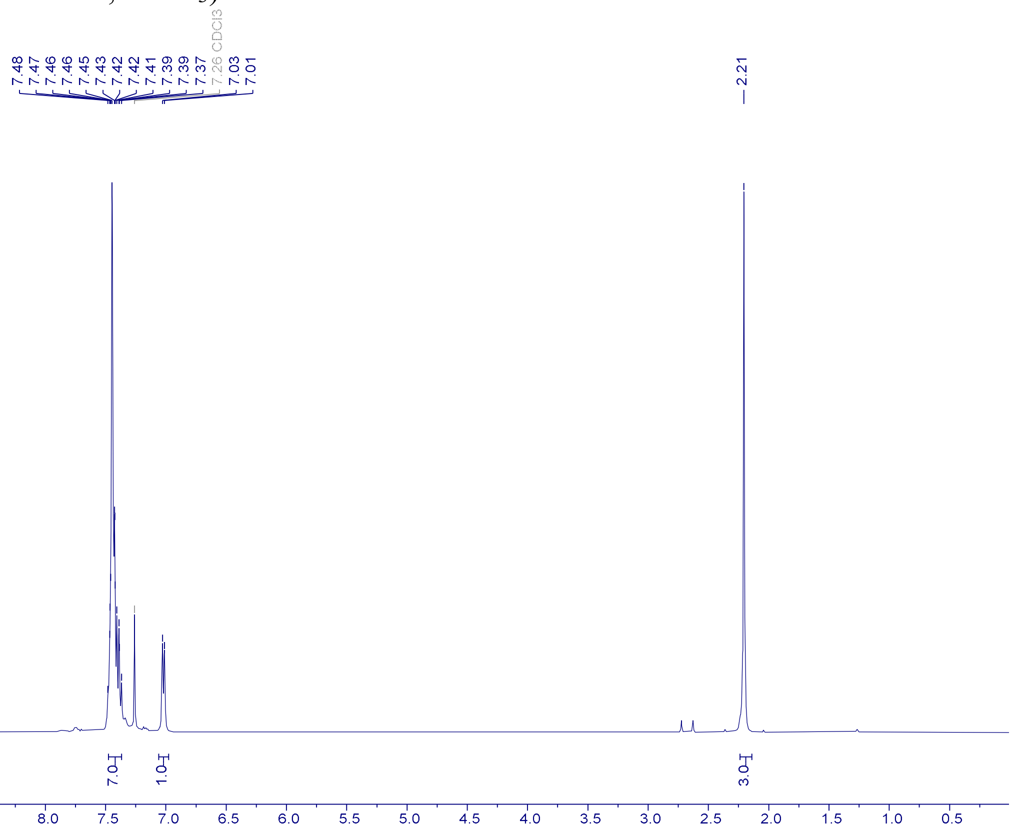

3 – ^{13}C NMR (151 MHz, $CDCl_3$)

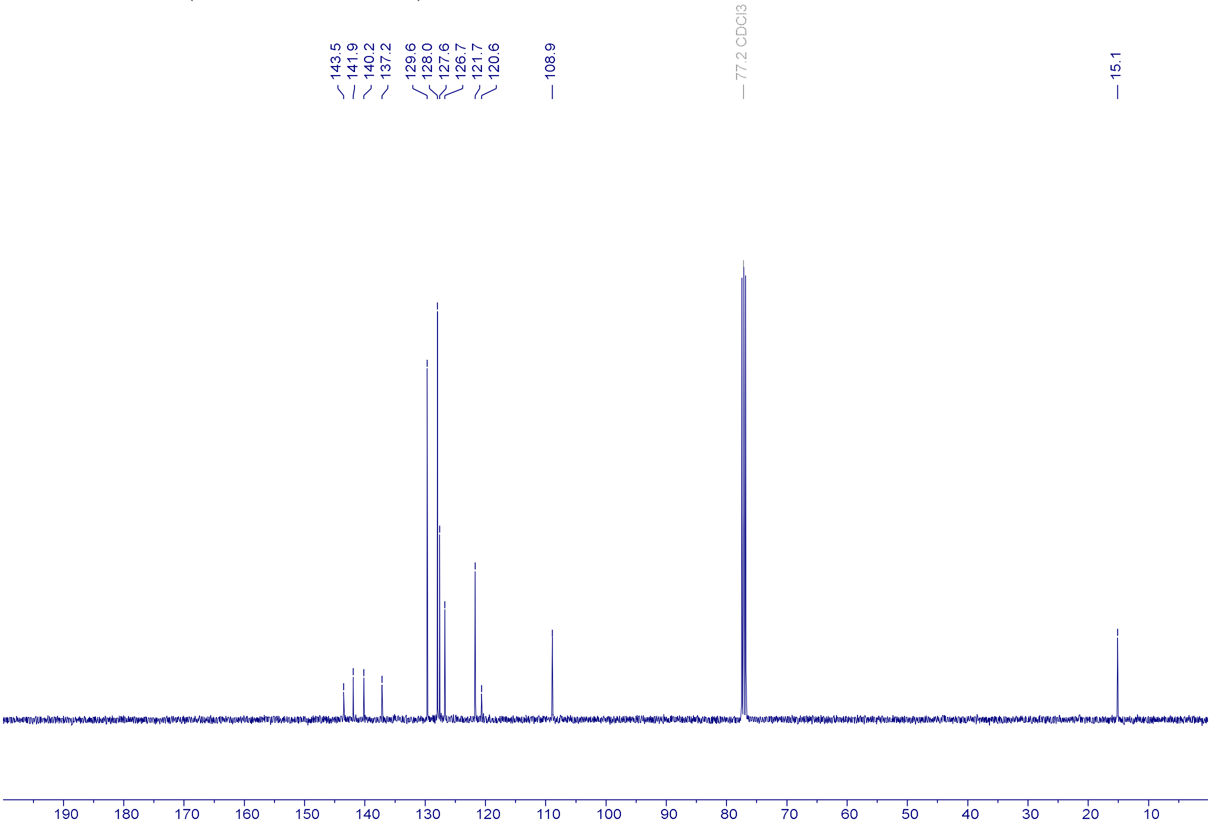

7 – ^{1}H NMR (600 MHz, $CDCl_3$)

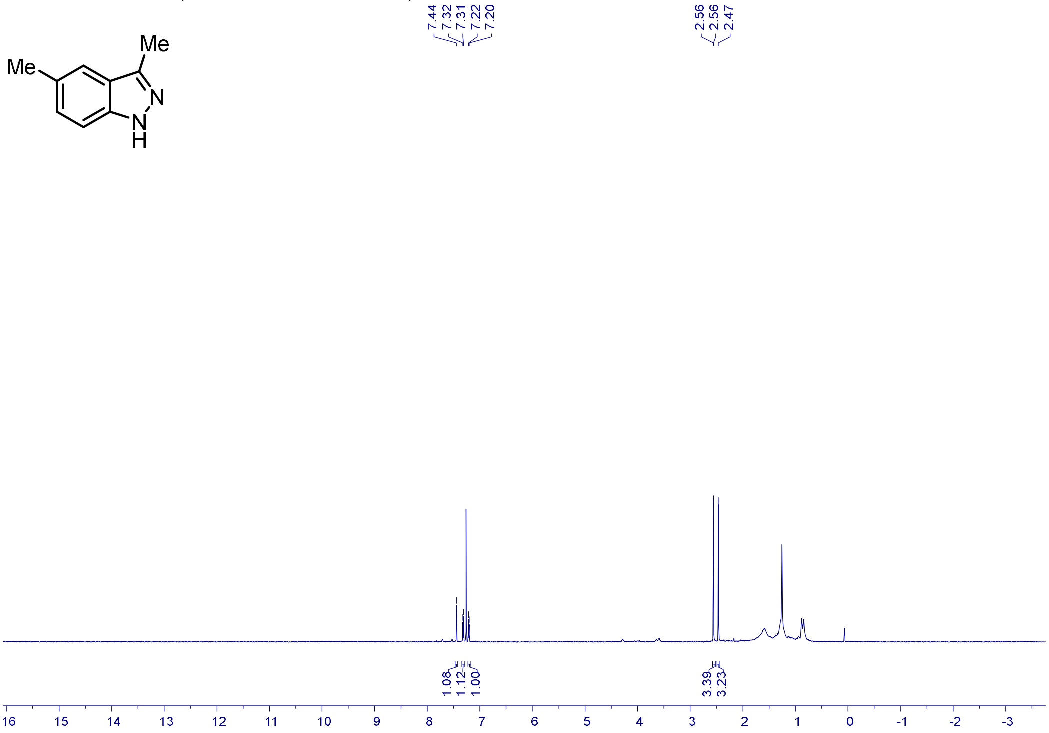

7 – ^{13}C NMR (151 MHz, $CDCl_3$)

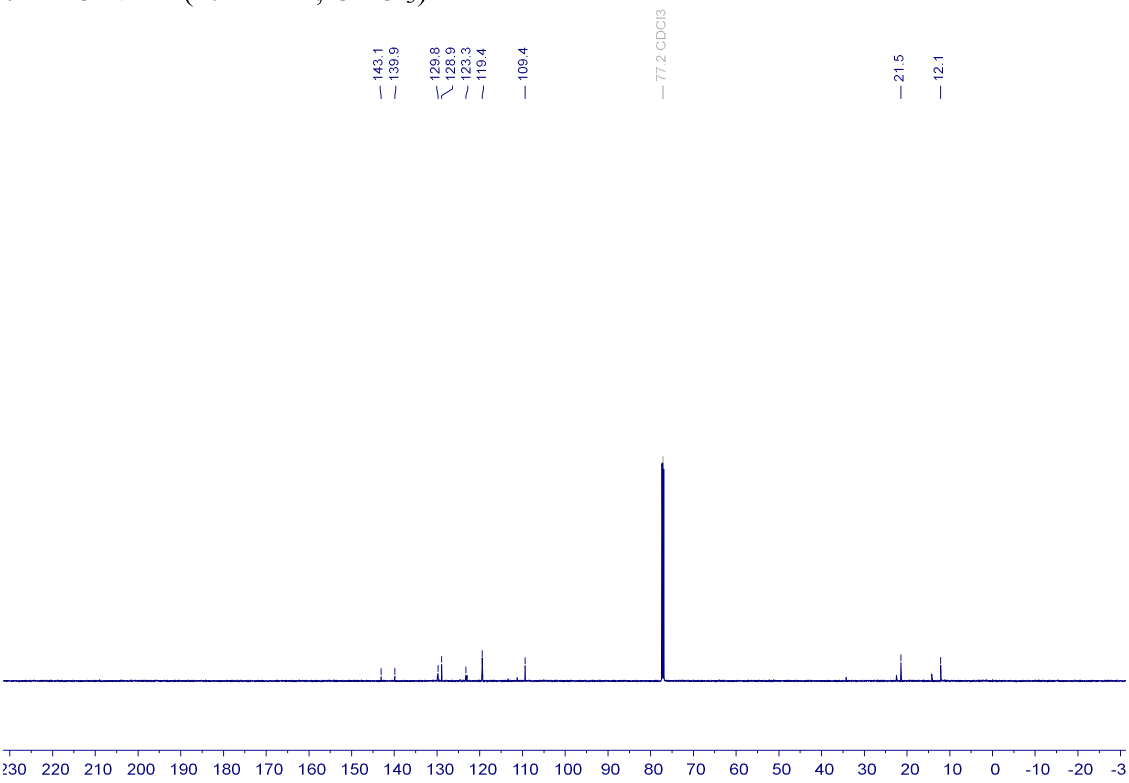

9 – ^{1}H NMR (400 MHz, $CDCl_3$)

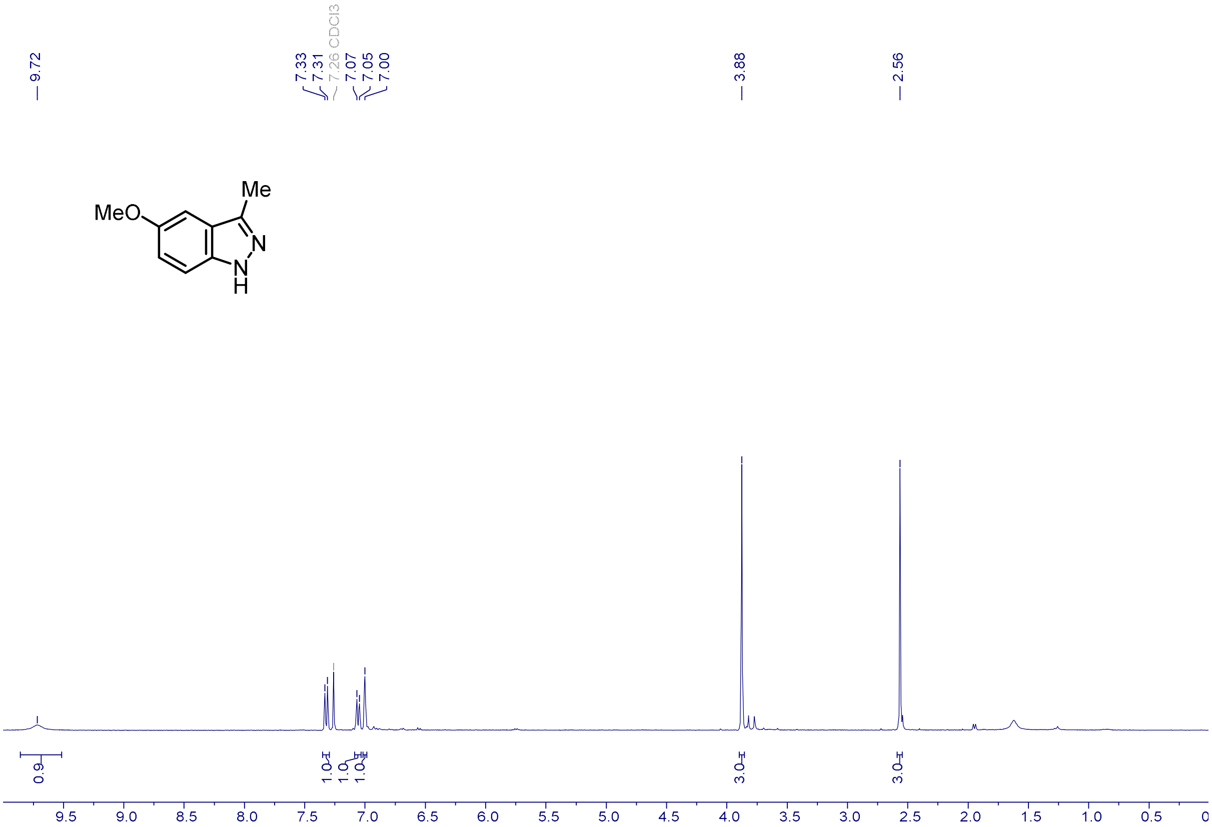

9 – ^{13}C NMR (101 MHz, $CDCl_3$)

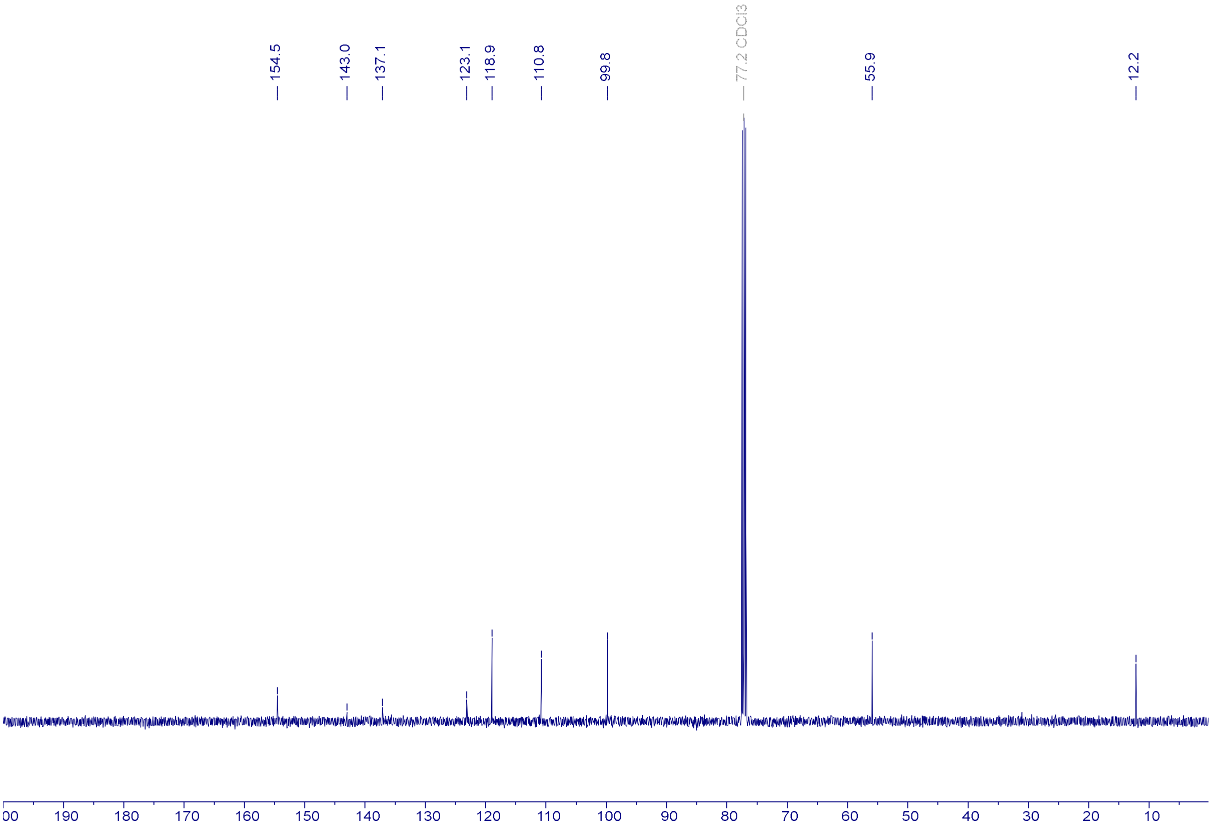

13 – ^{1}H NMR (600 MHz, DMSO-d_6)

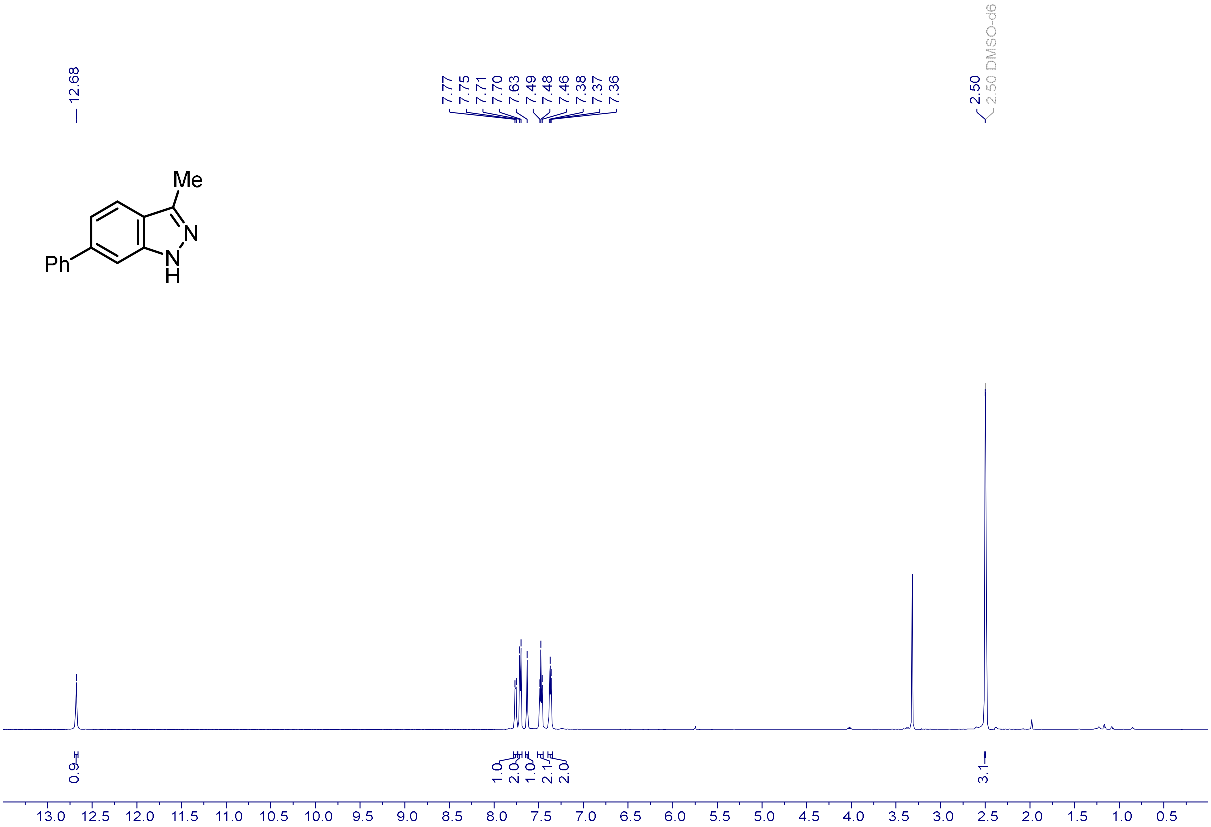

13 – ^{13}C NMR (151 MHz, DMSO-d_6)

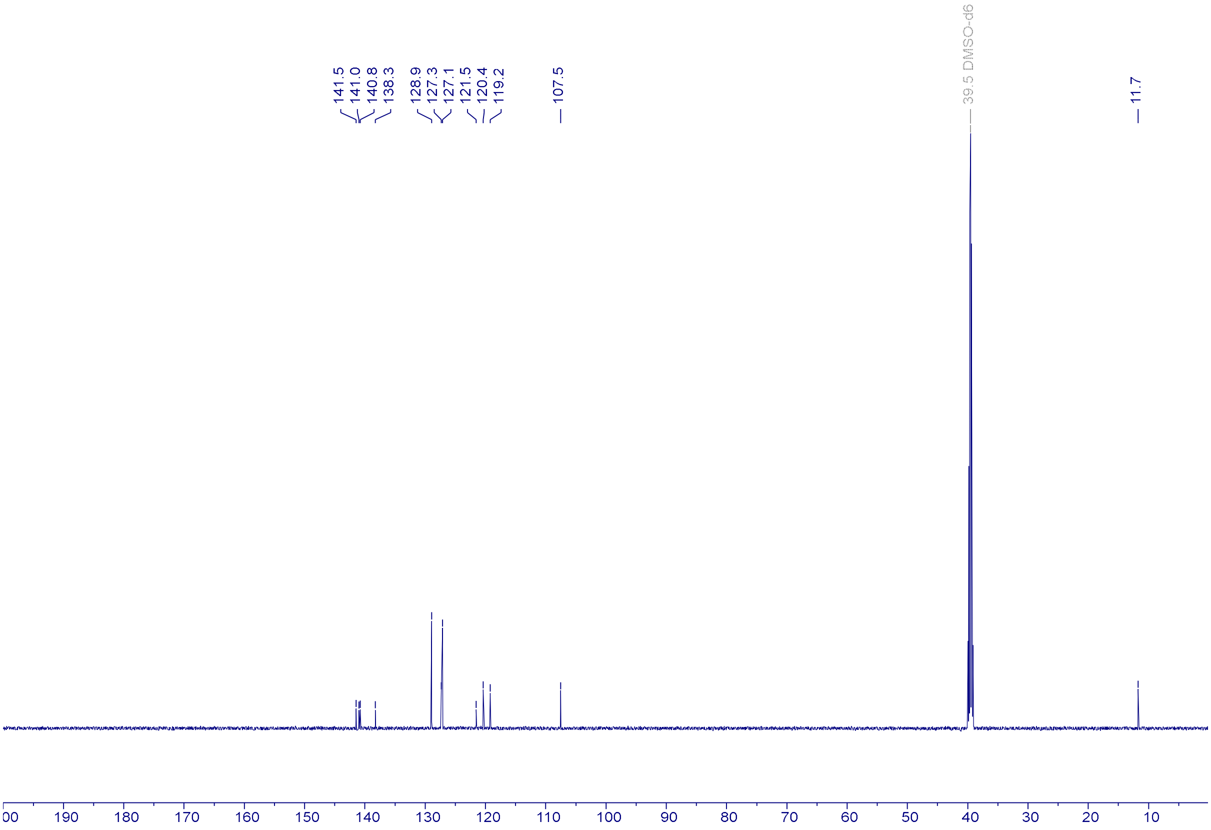

16 – ^{1}H NMR (600 MHz, $CDCl_3$)

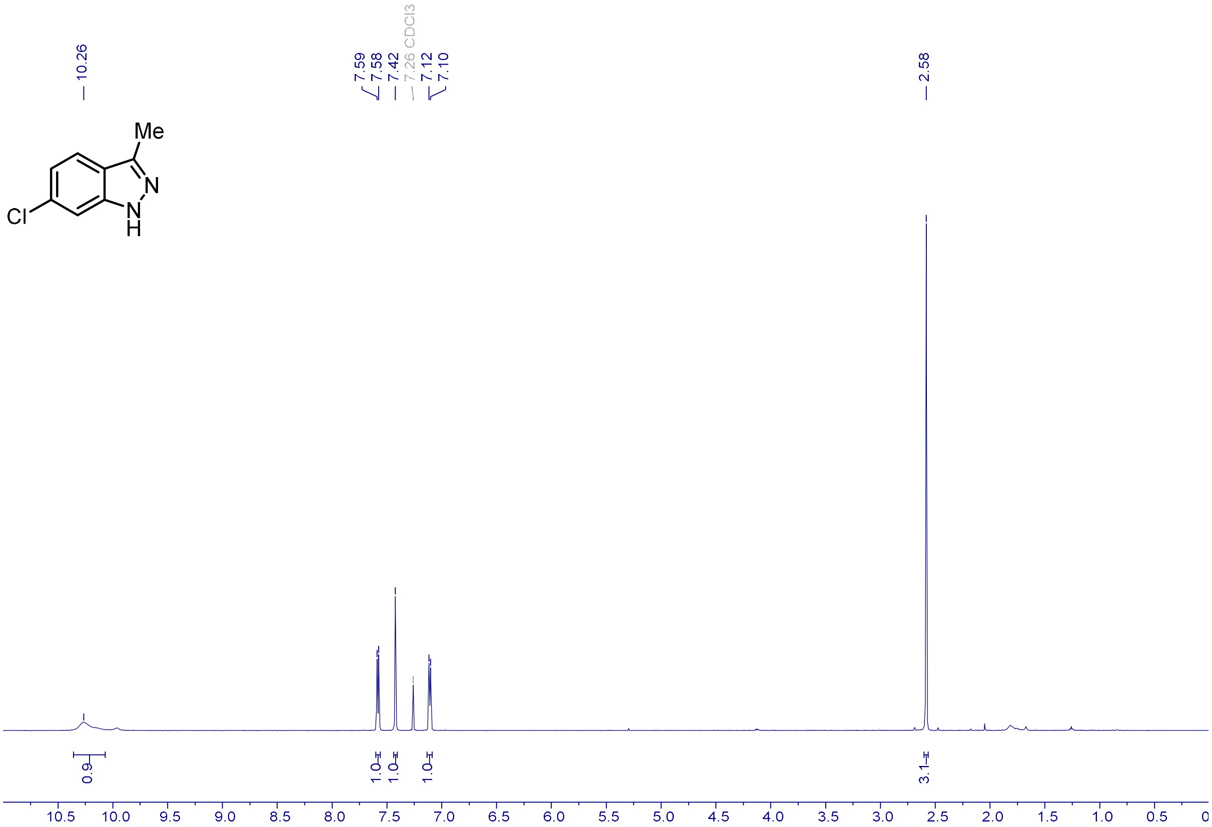

16 – ^{13}C NMR (151 MHz, $CDCl_3$)

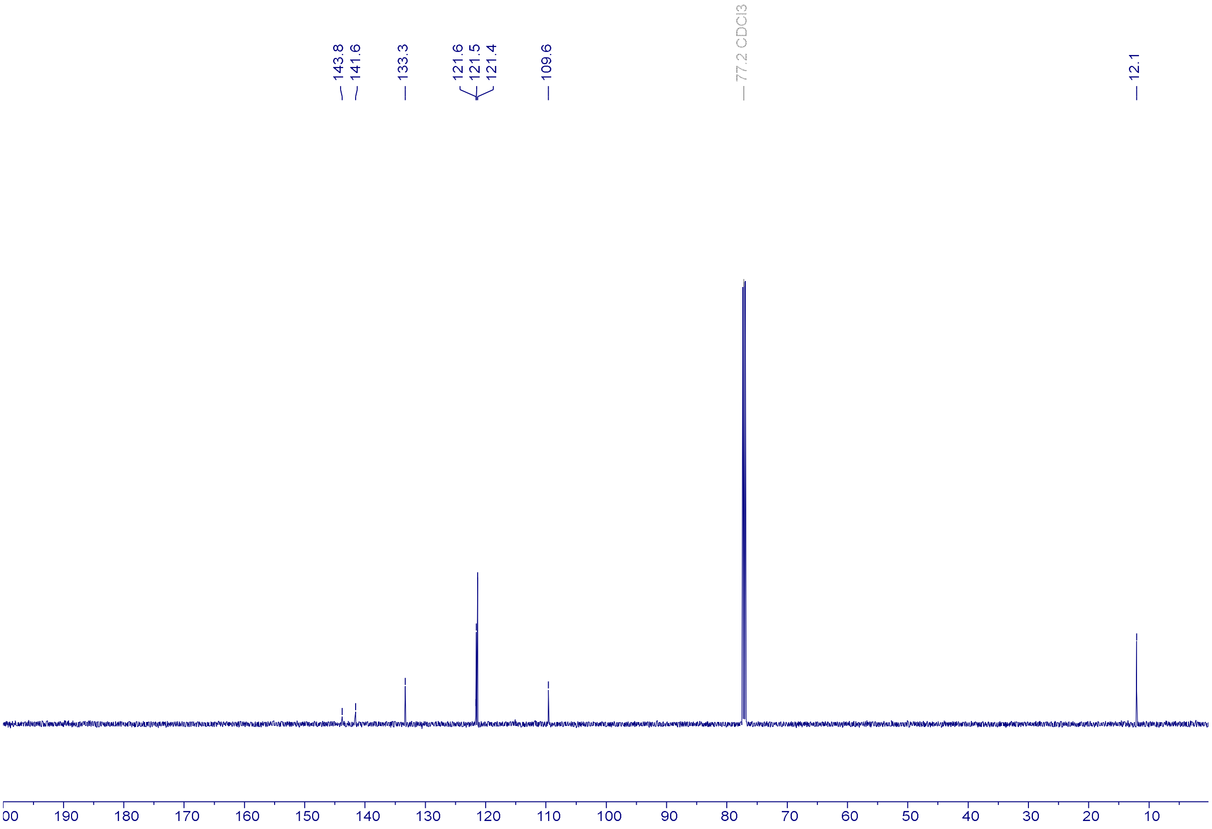

18 – ^{1}H NMR (600 MHz, $CDCl_3$)

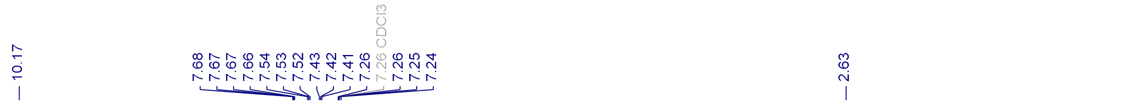

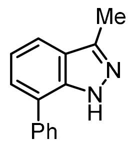

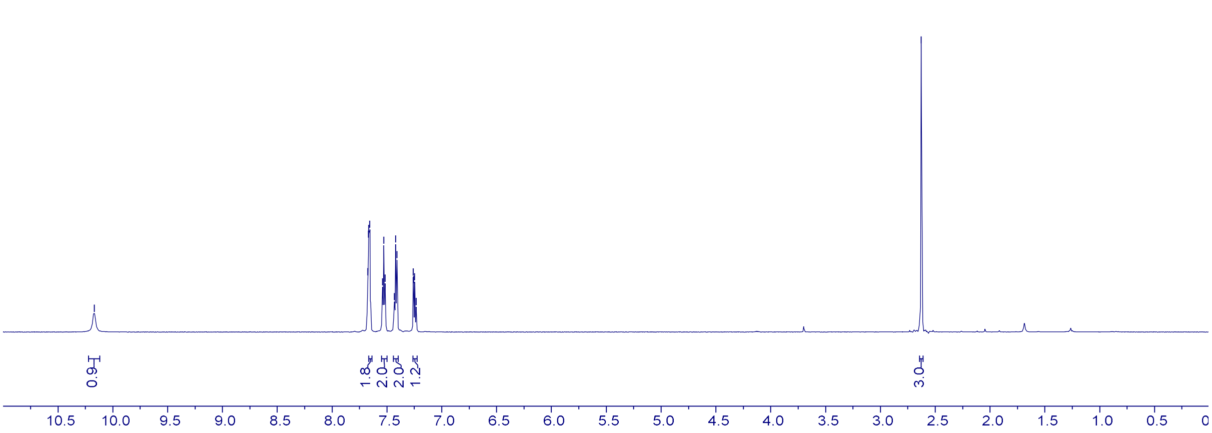

18 – ^{13}C NMR (151 MHz, $CDCl_3$)

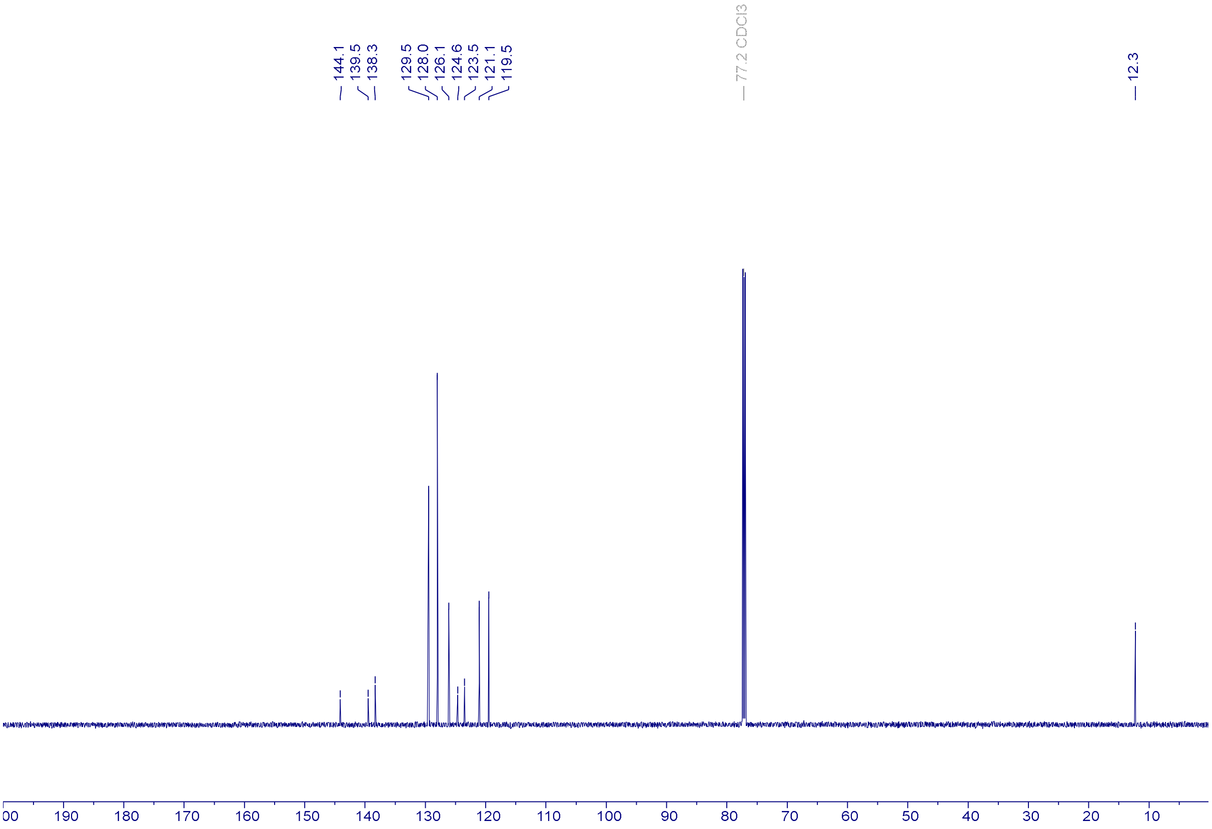

19 – ^{1}H NMR (600 MHz, $CDCl_3$)

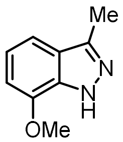

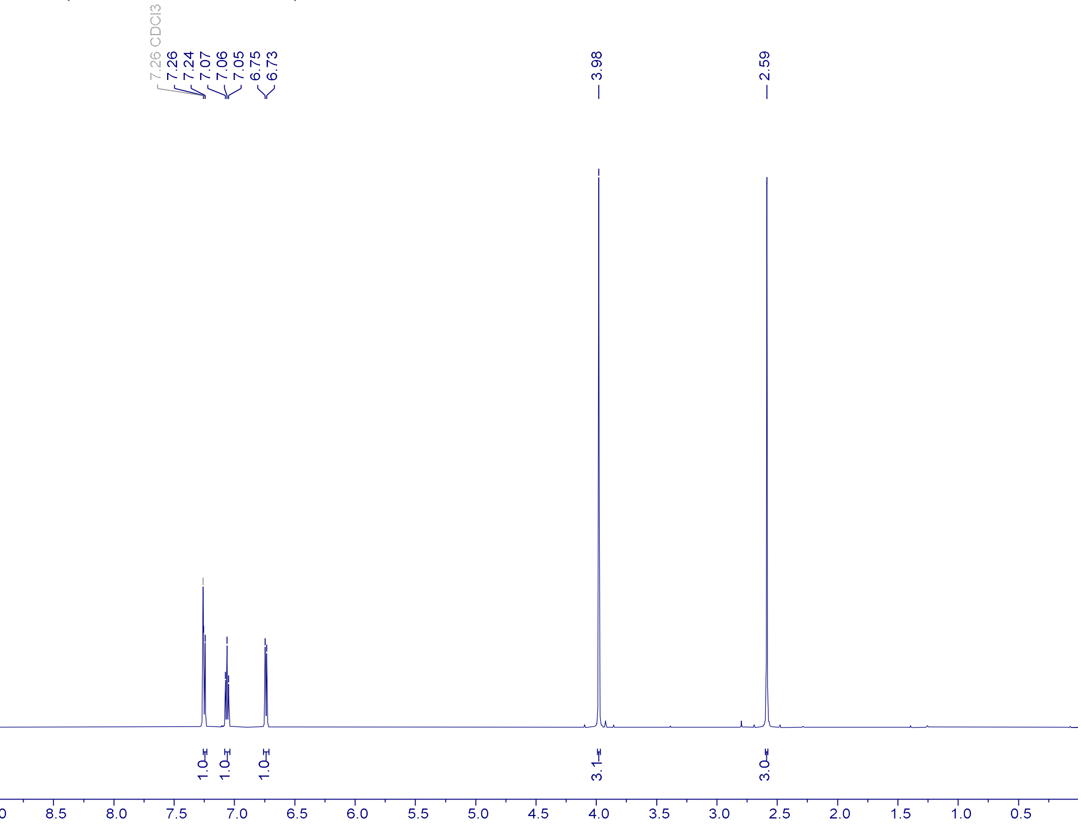

19 – ^{13}C NMR (151 MHz, $CDCl_3$)

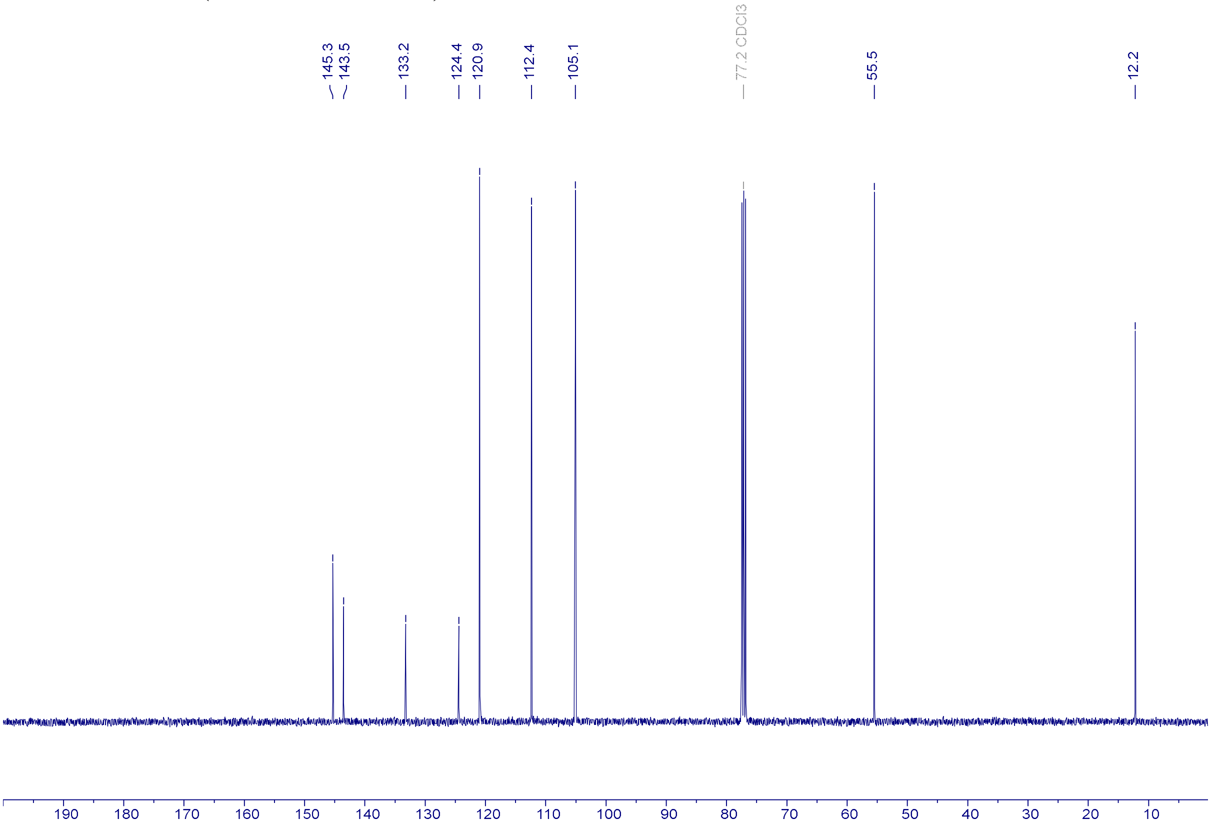

20 – ^{1}H NMR (600 MHz, $CDCl_3$)

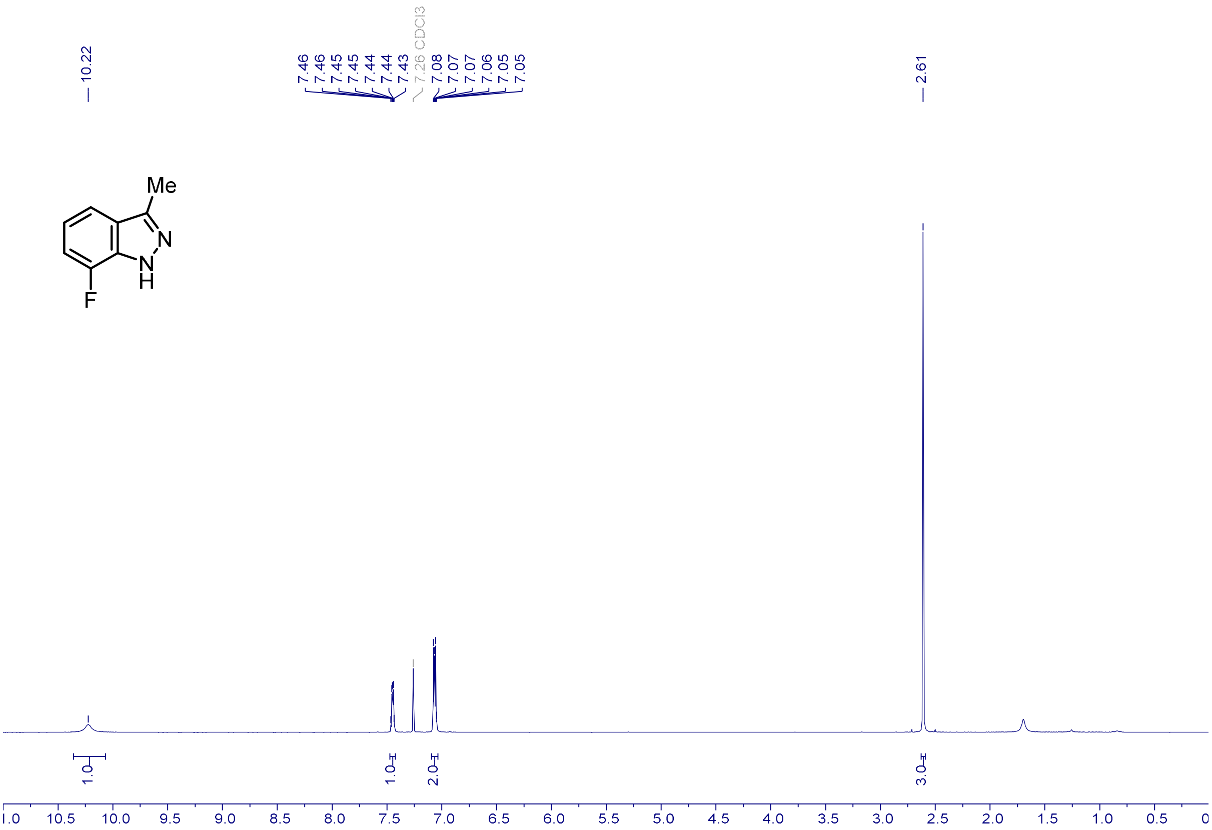

20 – ^{13}C NMR (151 MHz, $CDCl_3$)

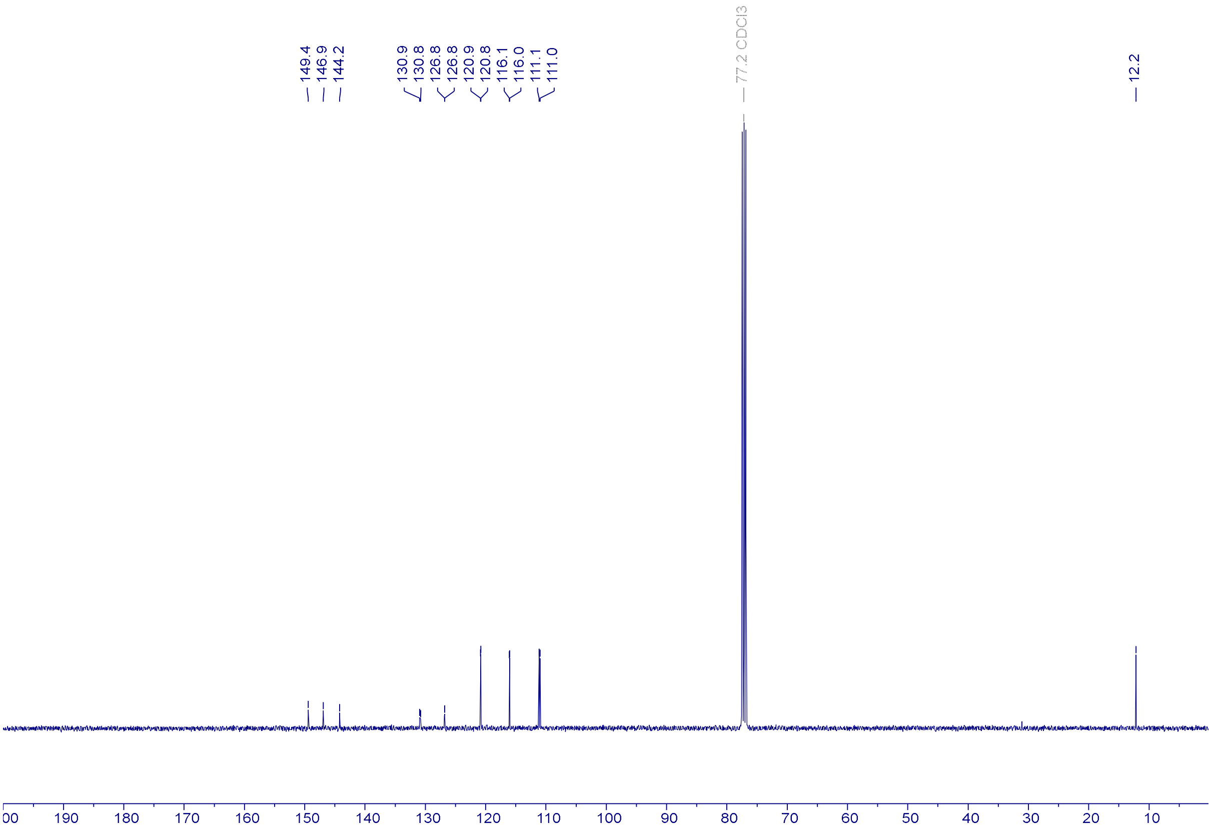

20 – ^{19}F NMR (565 MHz, $CDCl_3$)

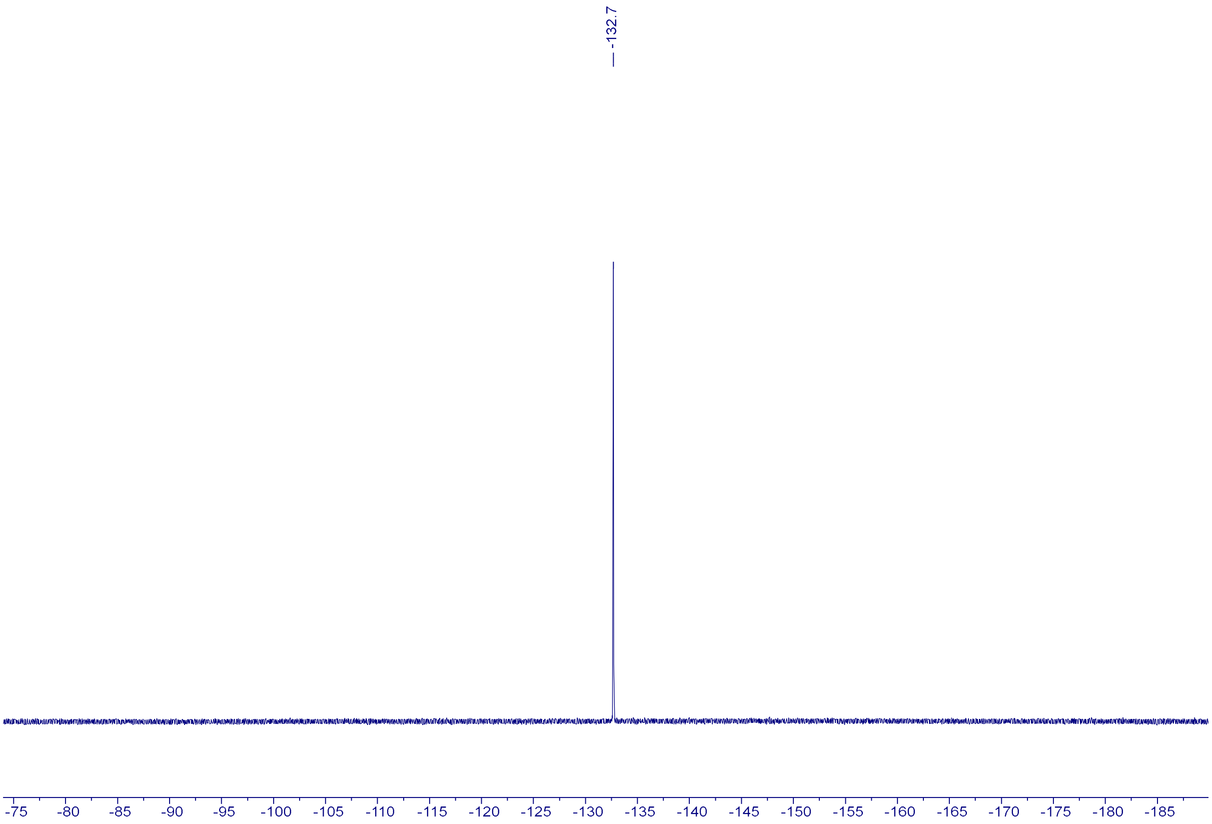

24 – ^{1}H NMR (600 MHz, $CDCl_3$)

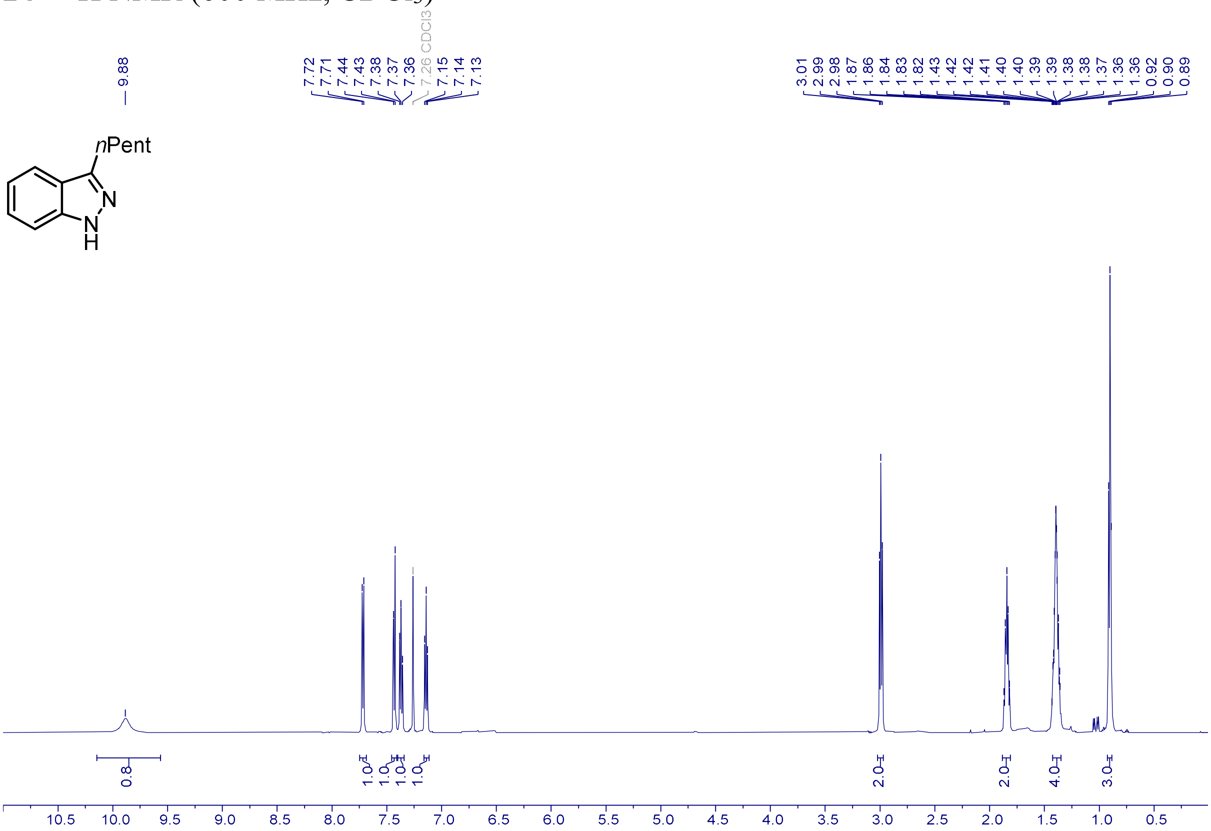

24 – ^{13}C NMR (151 MHz, $CDCl_3$)

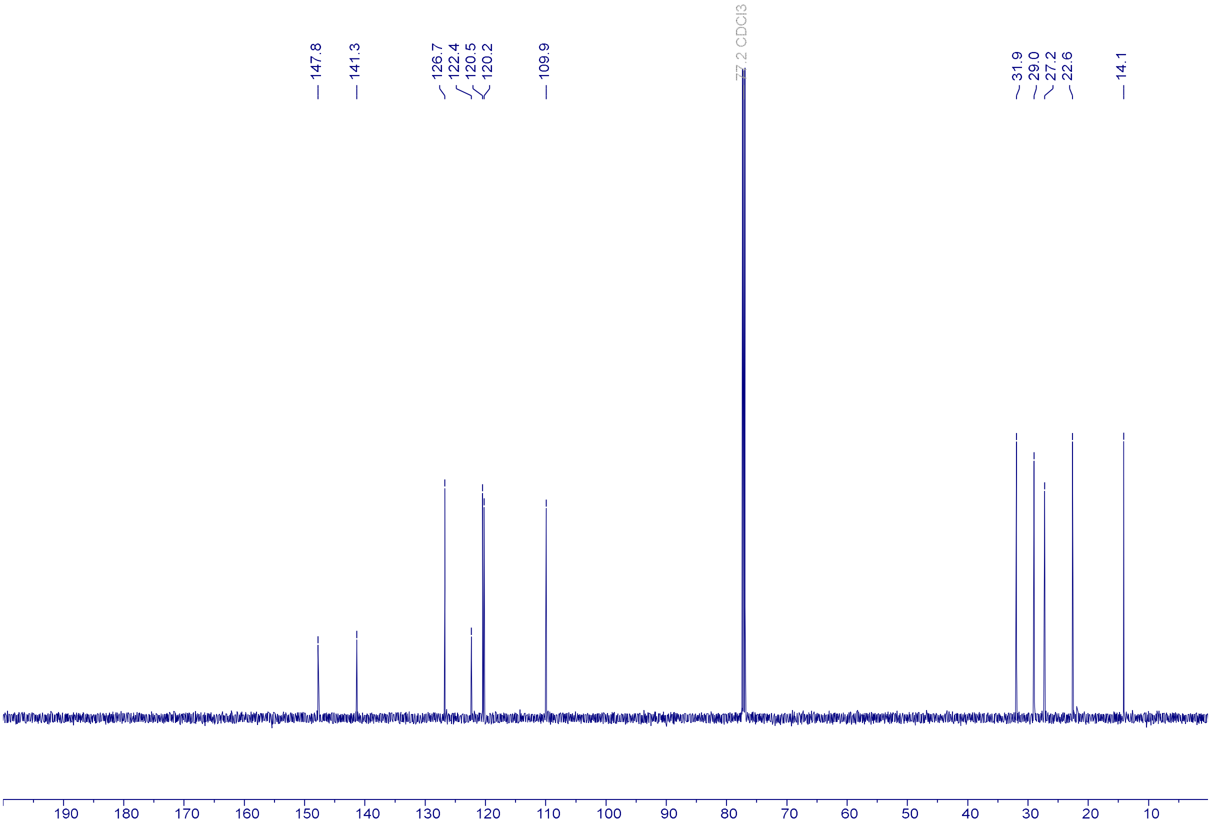

30 – ^{1}H NMR (400 MHz, $CDCl_3$)

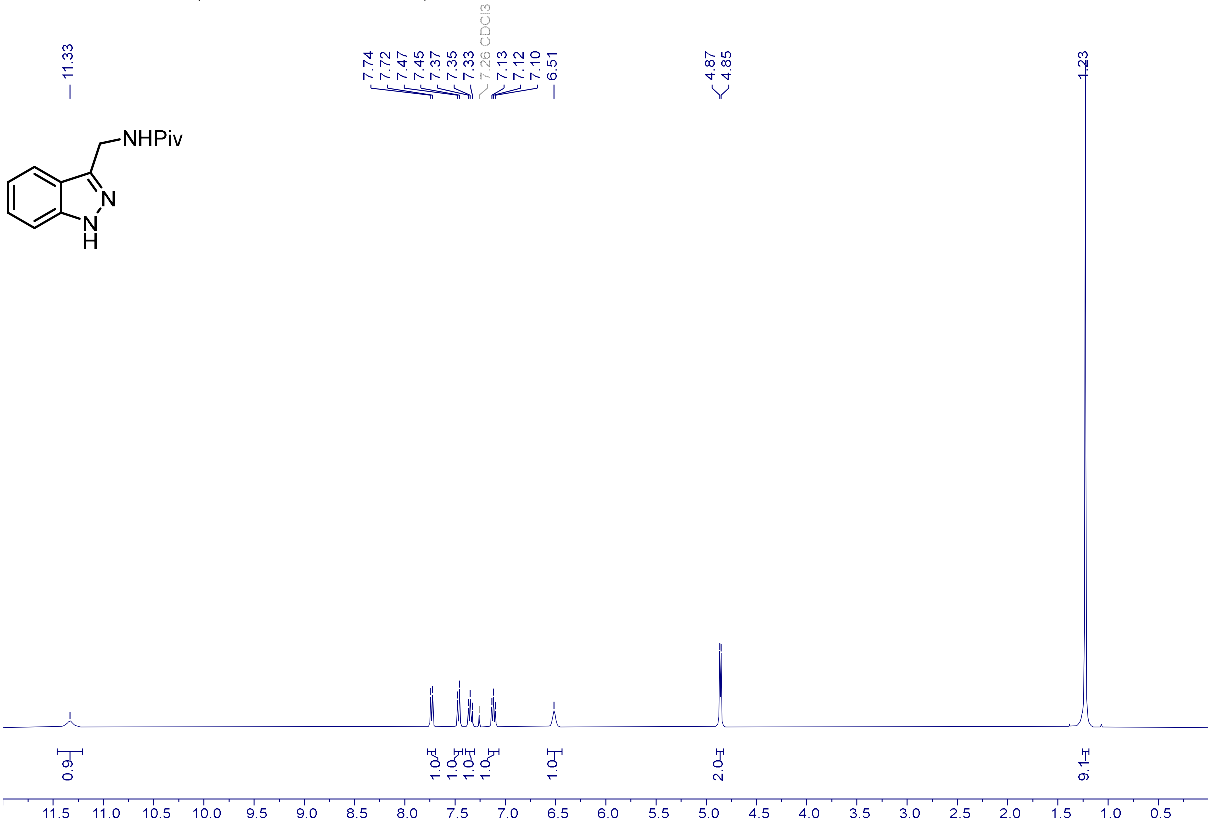

30 – ^{13}C NMR (101 MHz, $CDCl_3$)

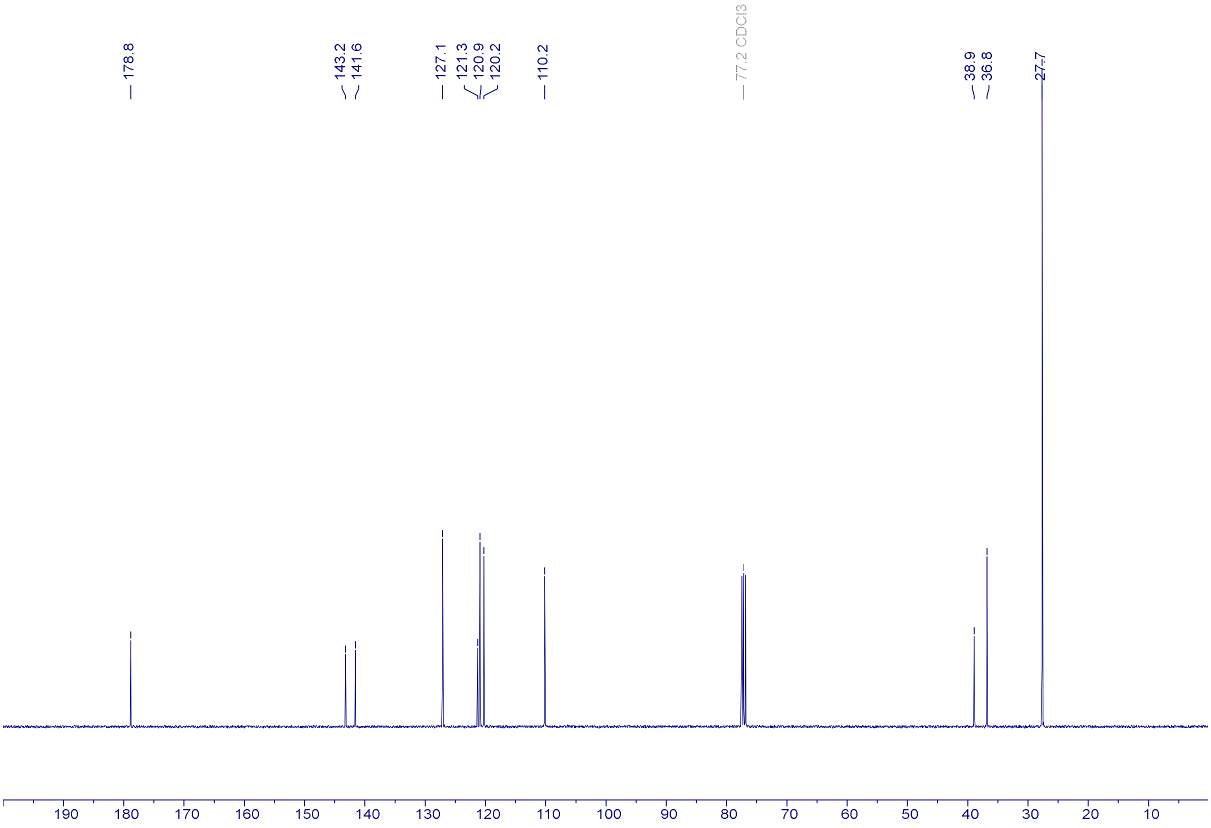

31 – ¹H NMR (400 MHz, $CDCl_3$)

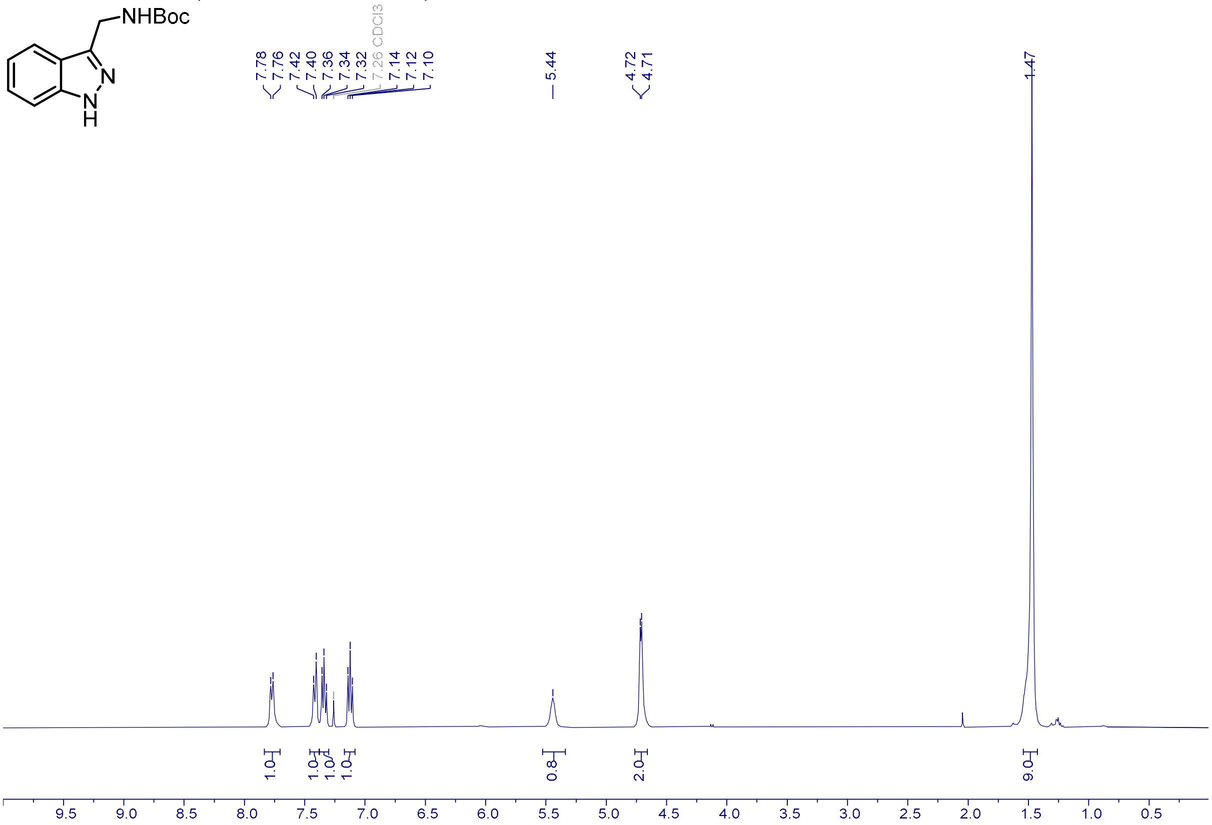

31 – ¹³C NMR (101 MHz, $CDCl_3$)

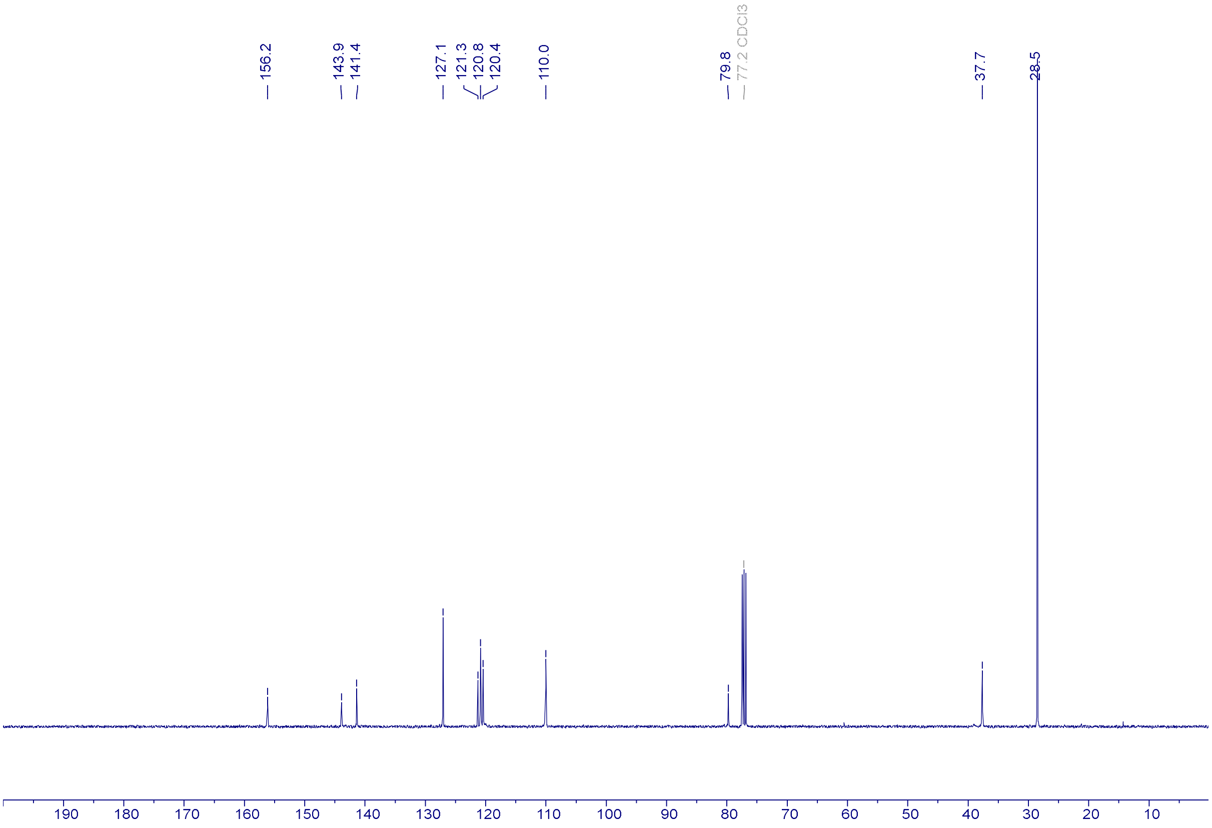

33 – ^{1}H NMR (400 MHz, MeOD)

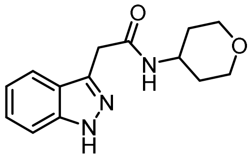

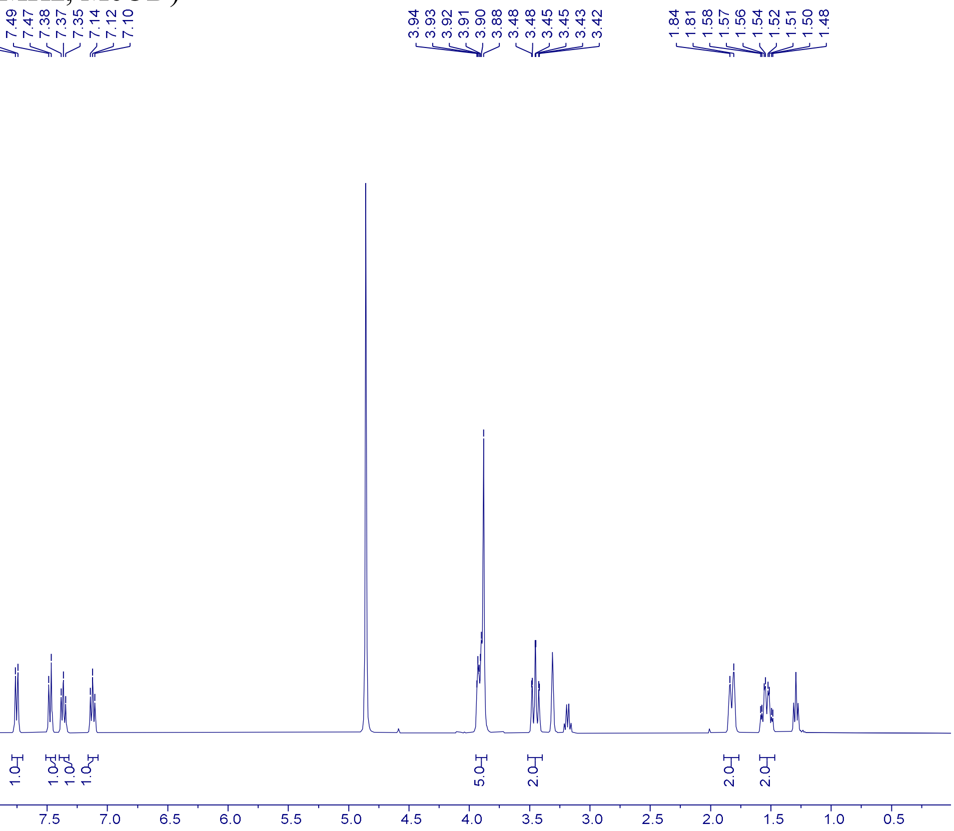

33 – ^{13}C NMR (101 MHz, MeOD)

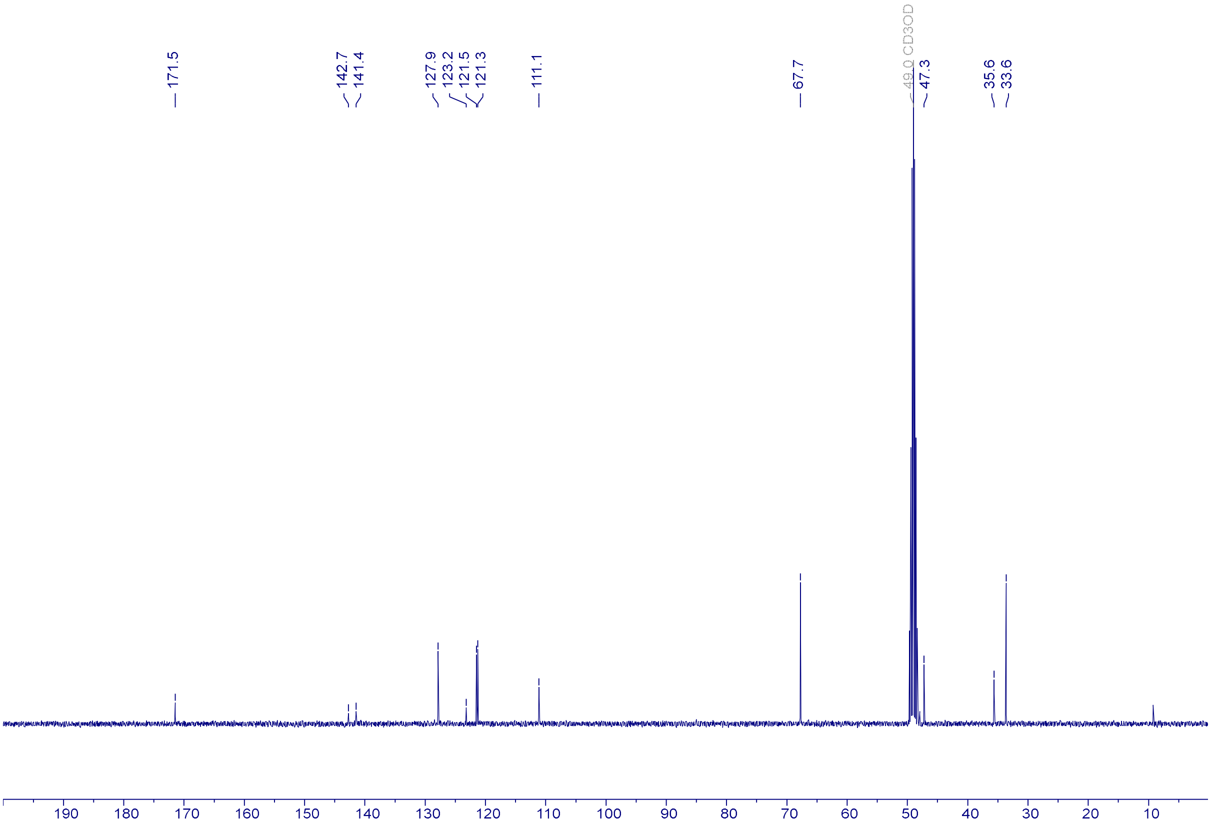

34 – ^{1}H NMR (400 MHz, $CDCl_3$)

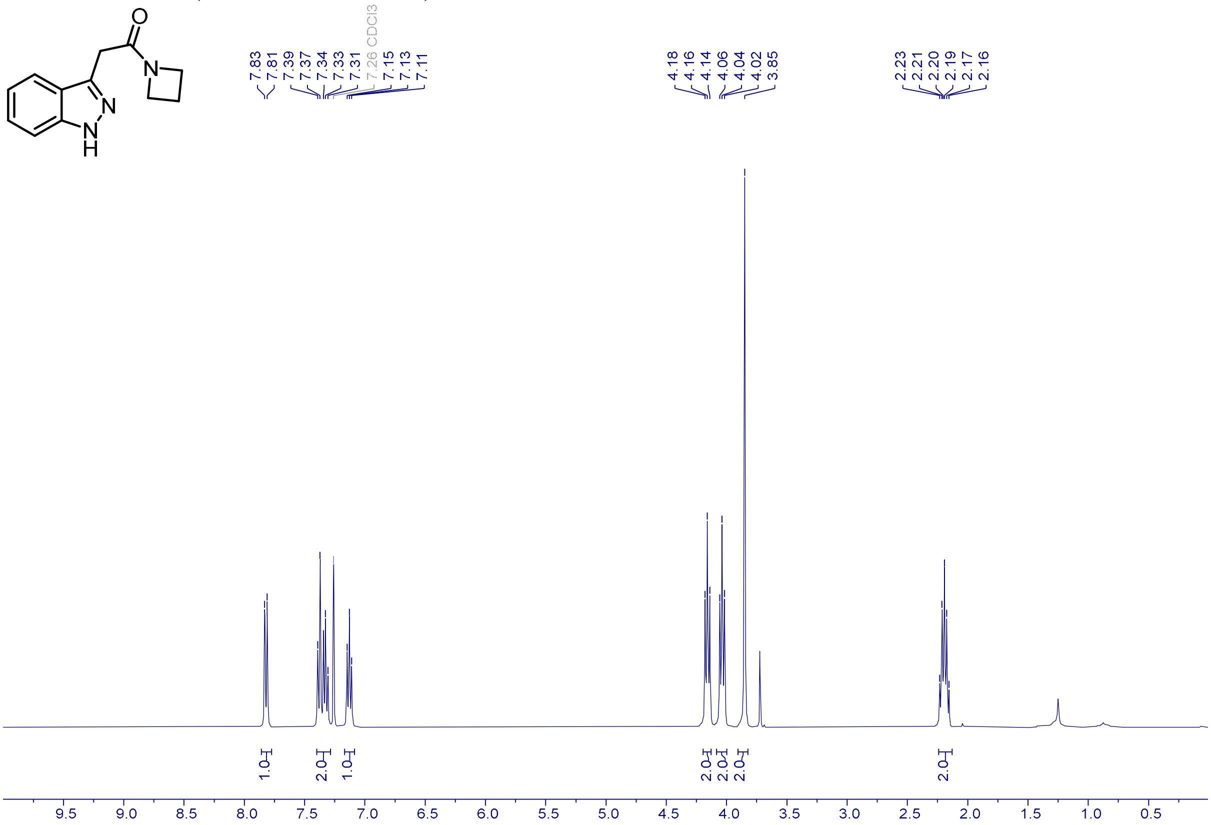

34 – ^{13}C NMR (101 MHz, $CDCl_3$)

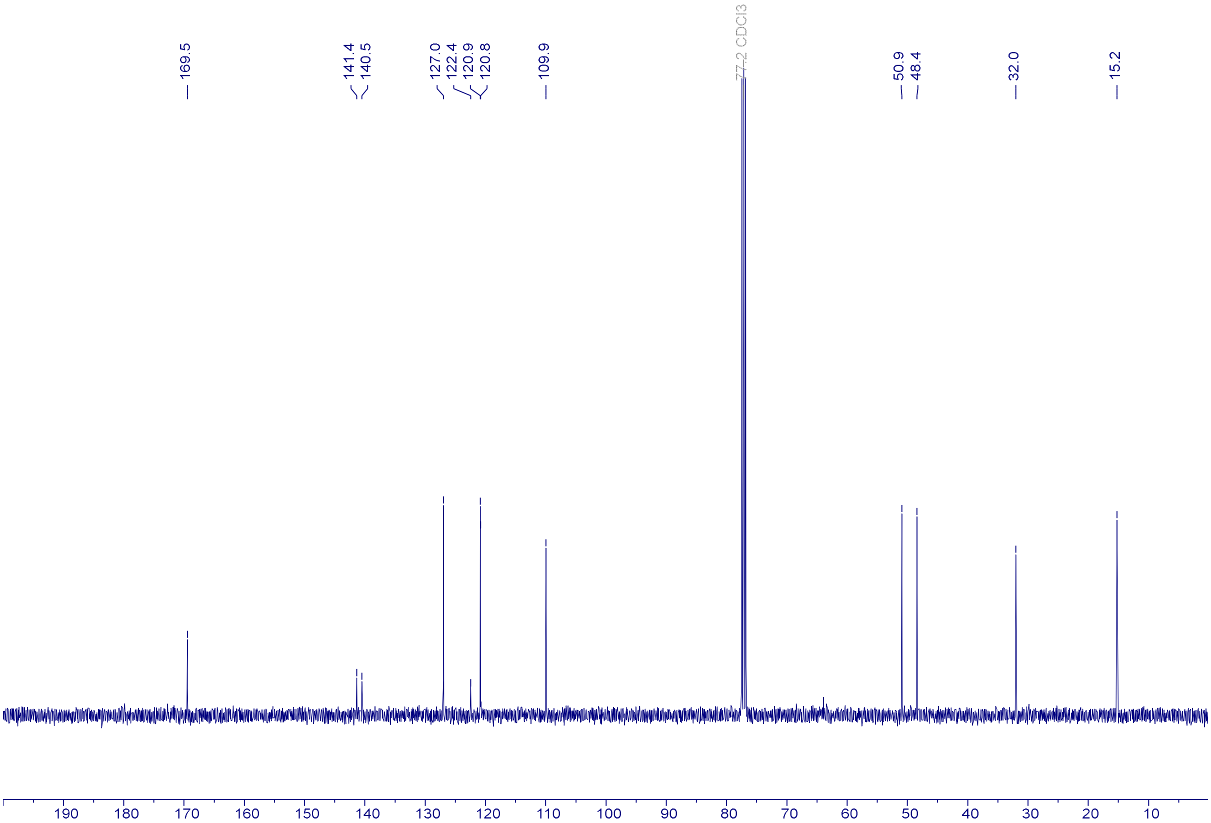

35 – ^{1}H NMR (400 MHz, $CDCl_3$)

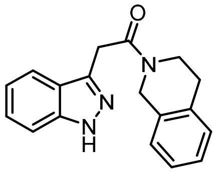

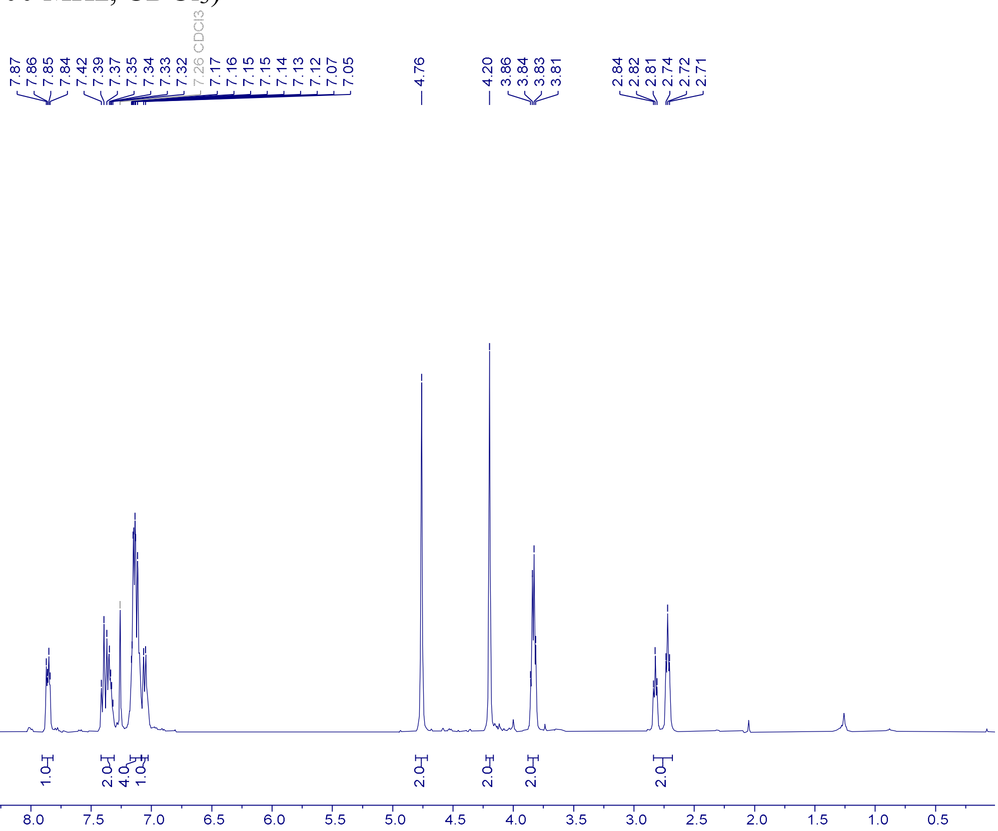

35 – ^{13}C NMR (101 MHz, $CDCl_3$)

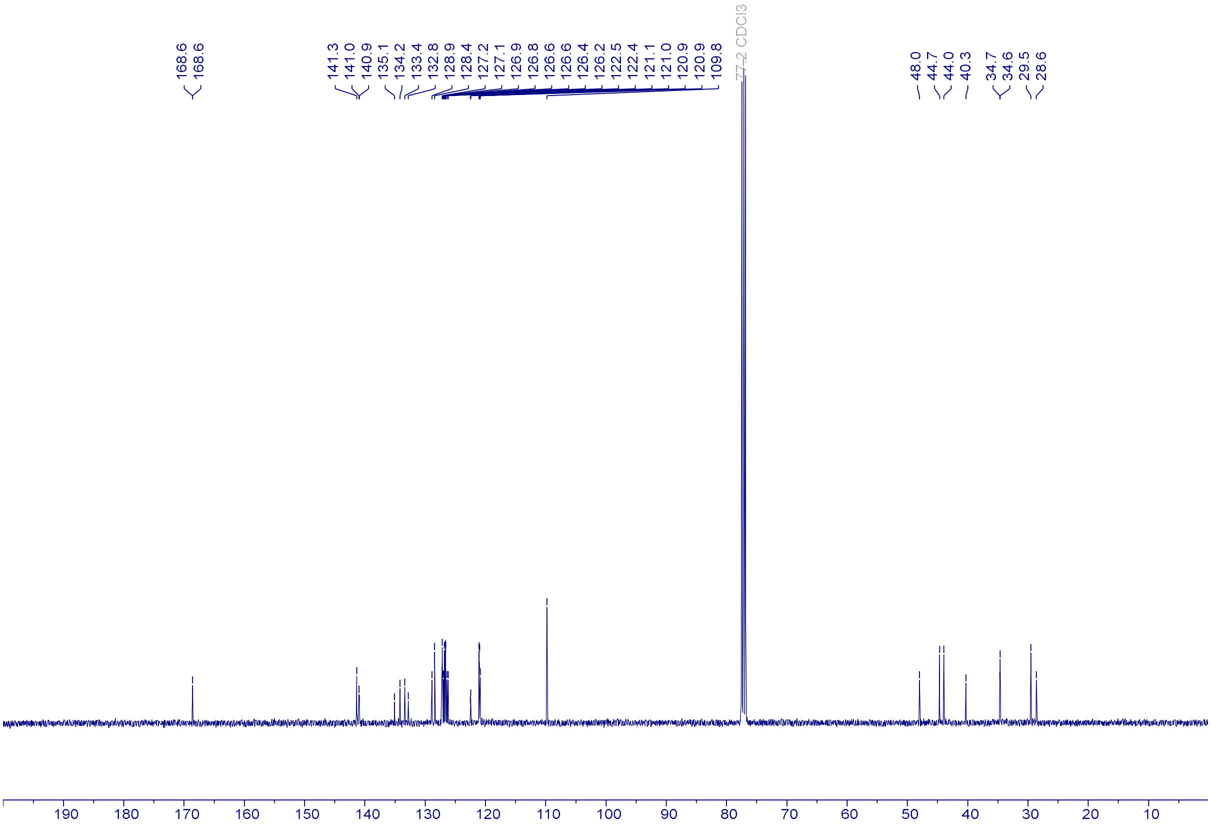

36 – ^{1}H NMR (400 MHz, $CDCl_3$)

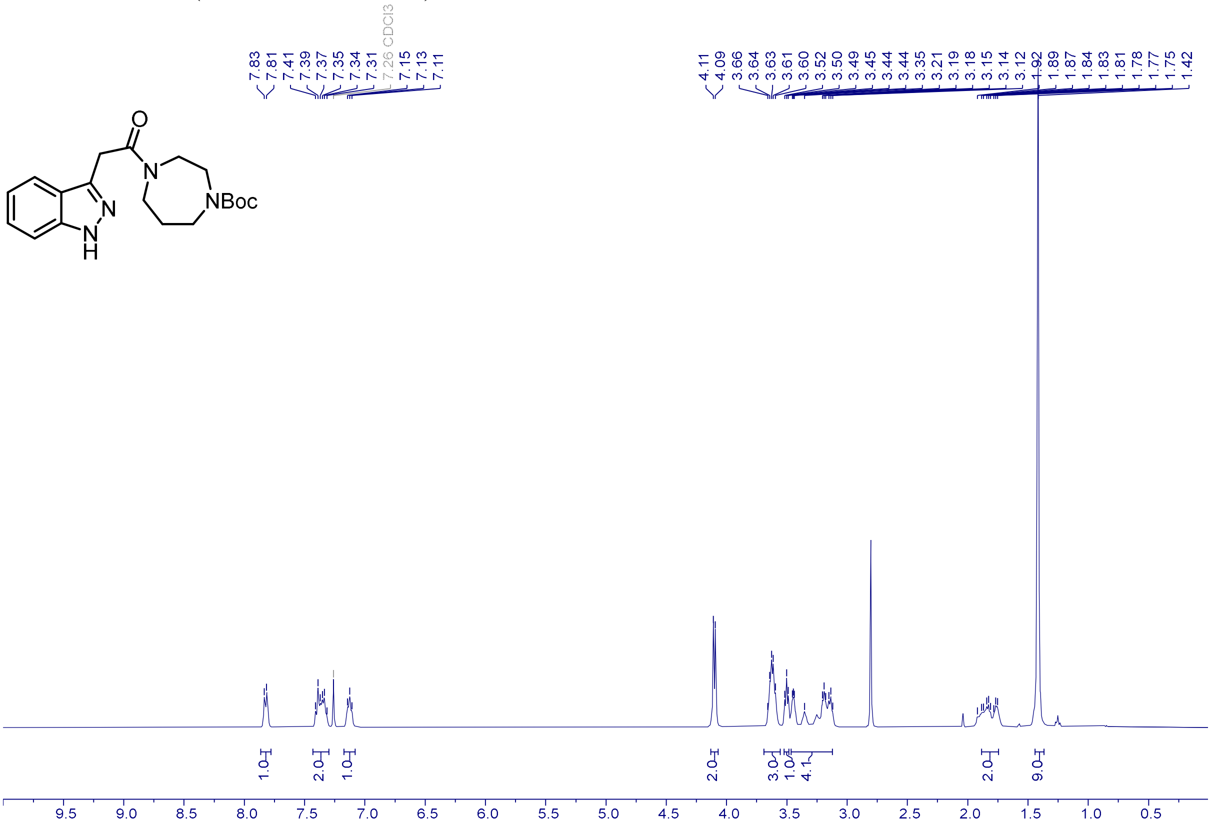

36 – ^{13}C NMR (101 MHz, $CDCl_3$)

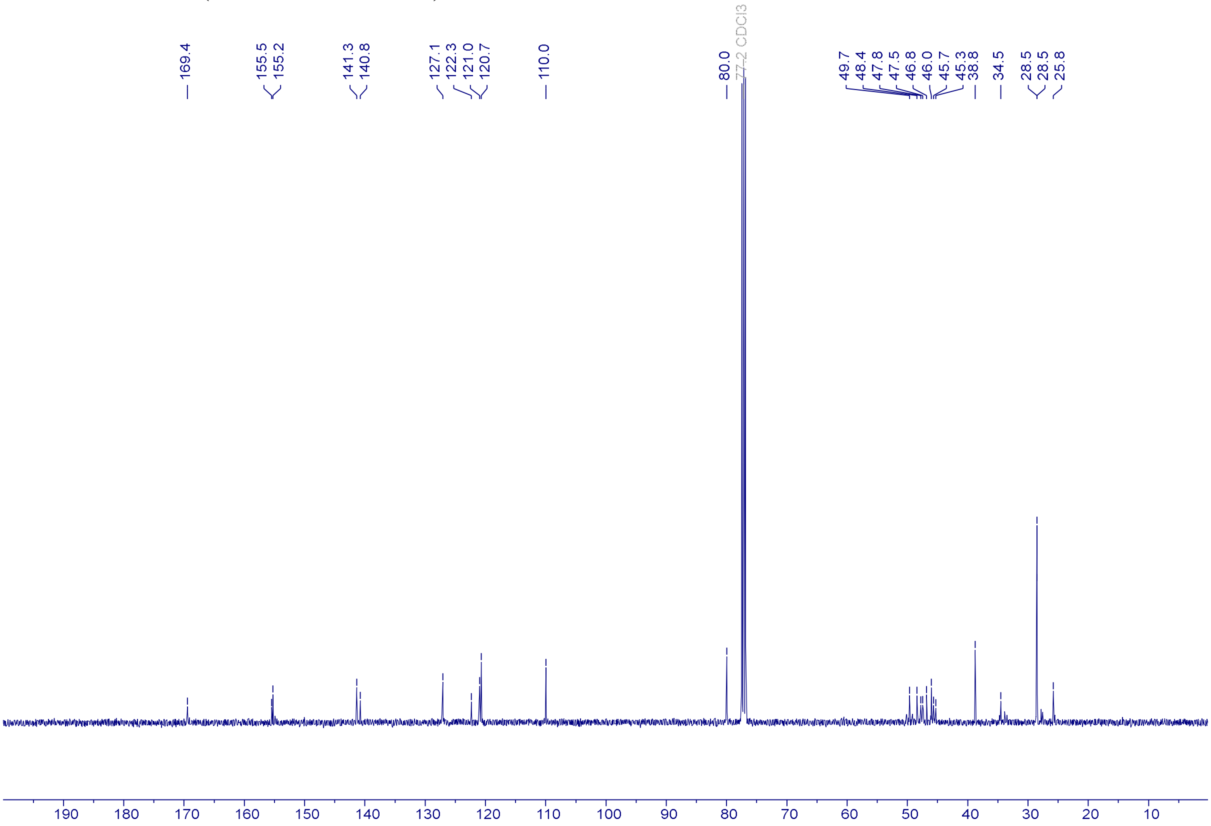

37 – ^{1}H NMR (400 MHz, $CDCl_3$)

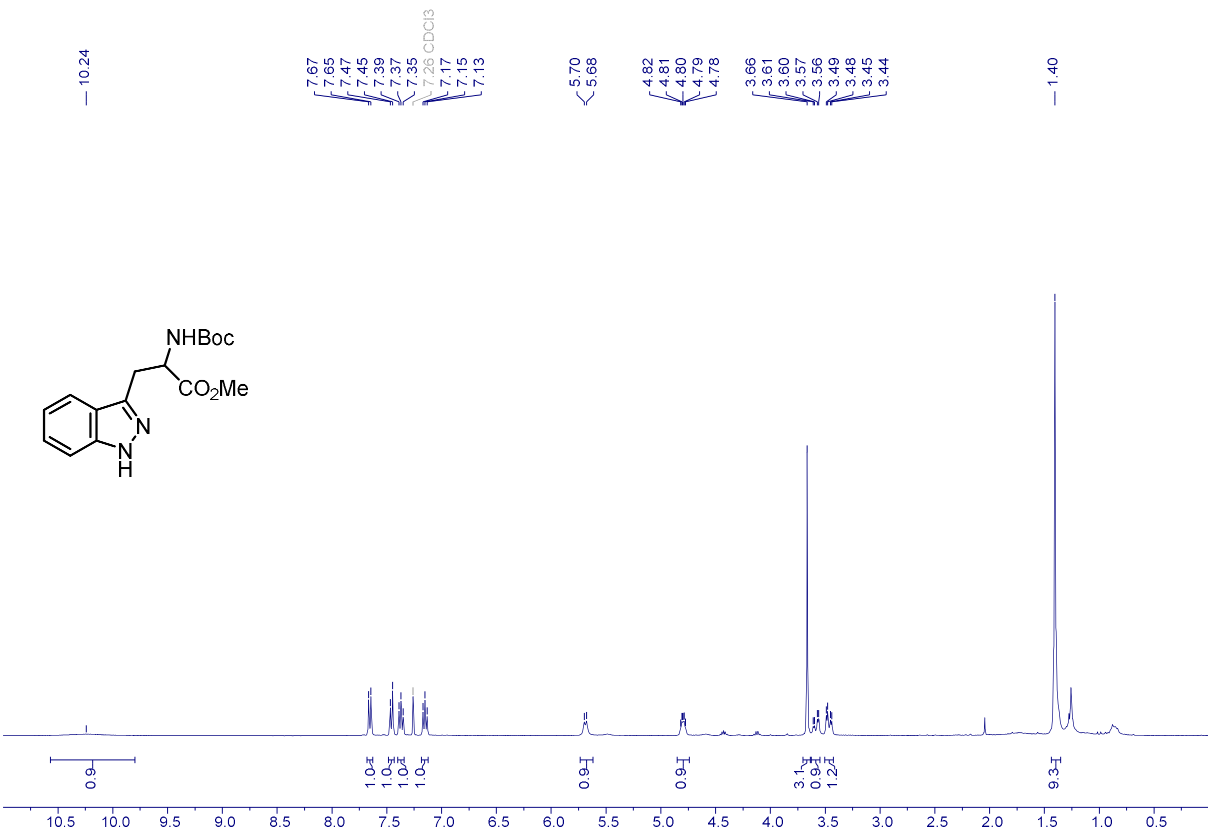

37 – ^{13}C NMR (101 MHz, $CDCl_3$)

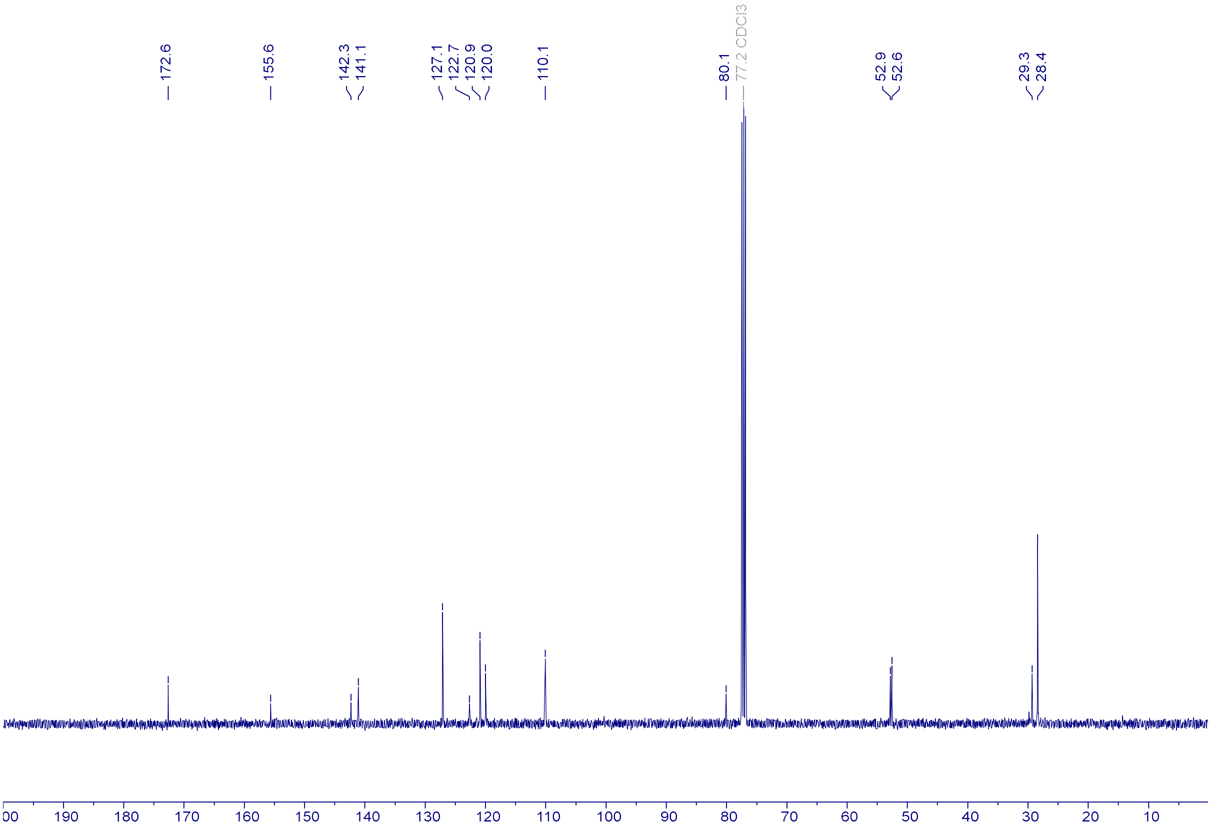

38 – ^{1}H NMR (600 MHz, $CDCl_3$)

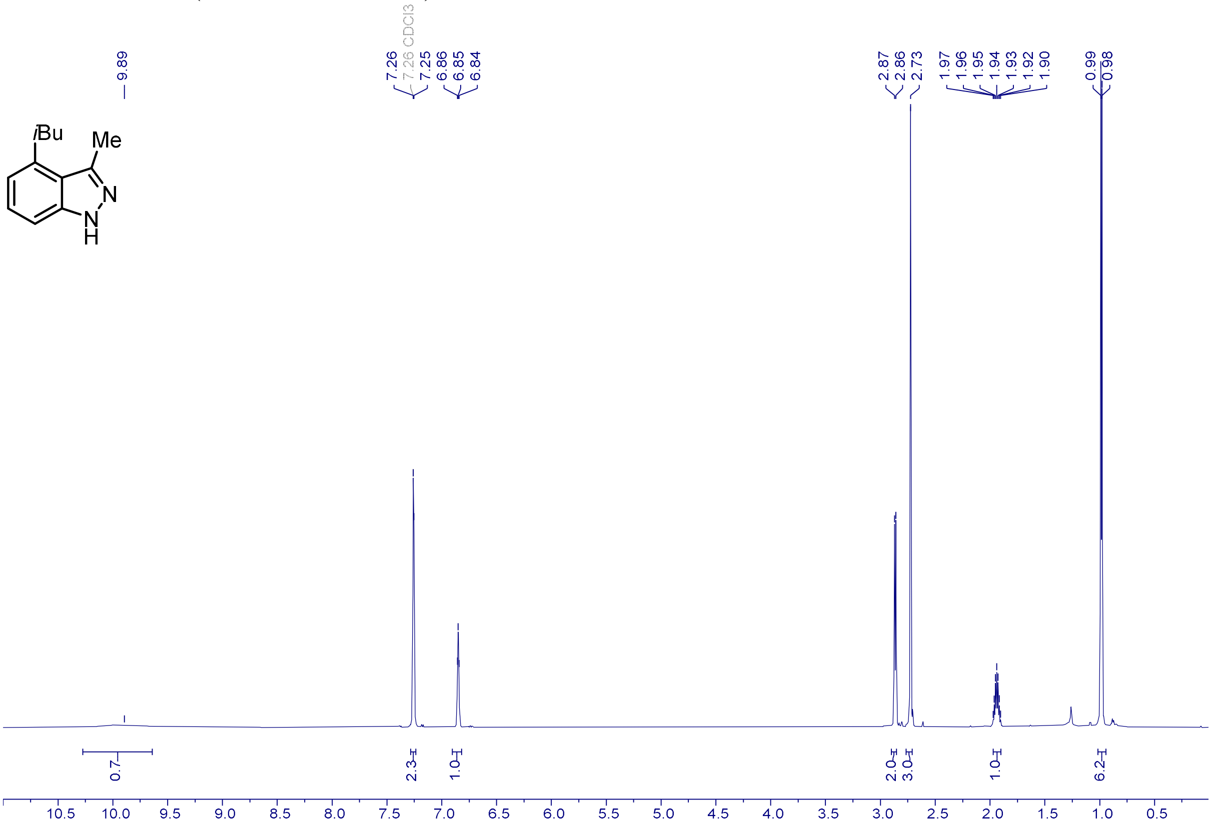

38 – ^{13}C NMR (151 MHz, $CDCl_3$)

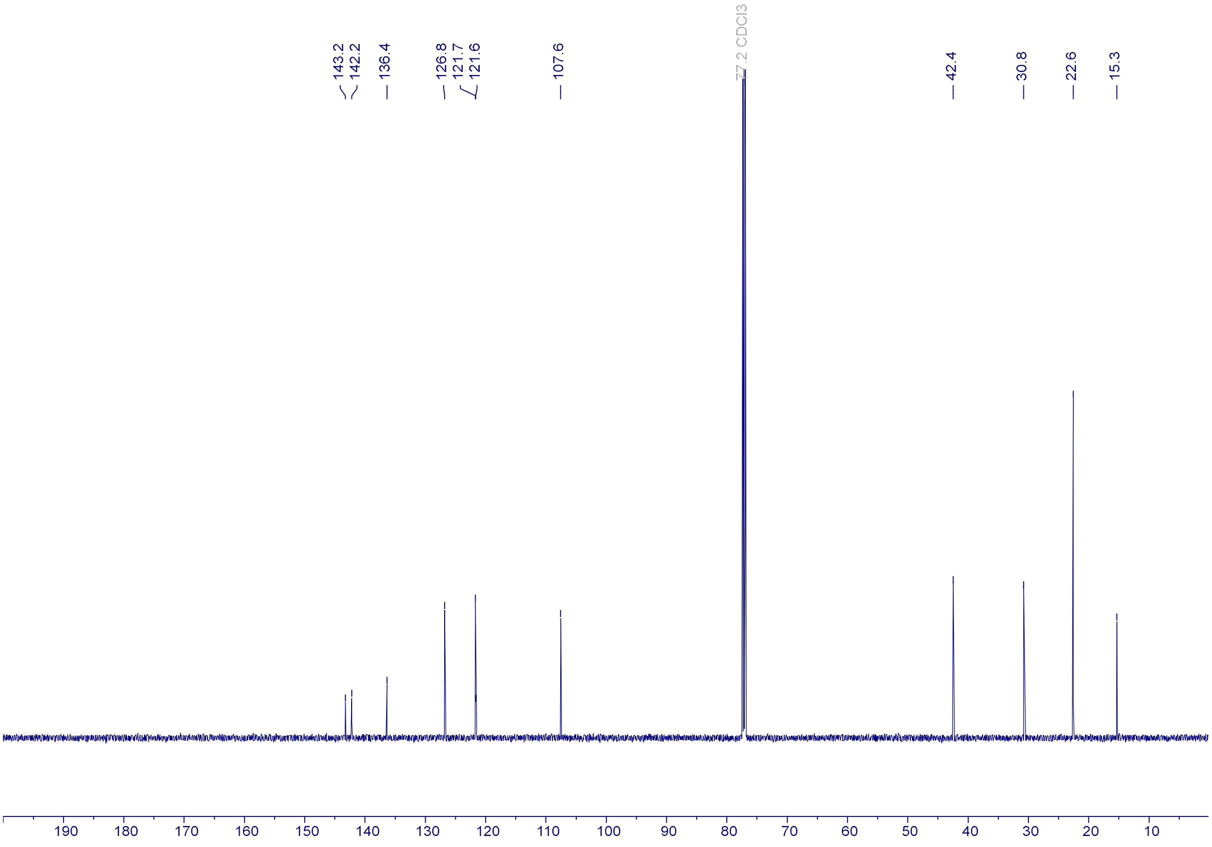

39’ - ^{1}H NMR (600 MHz, DMSO-d_6)

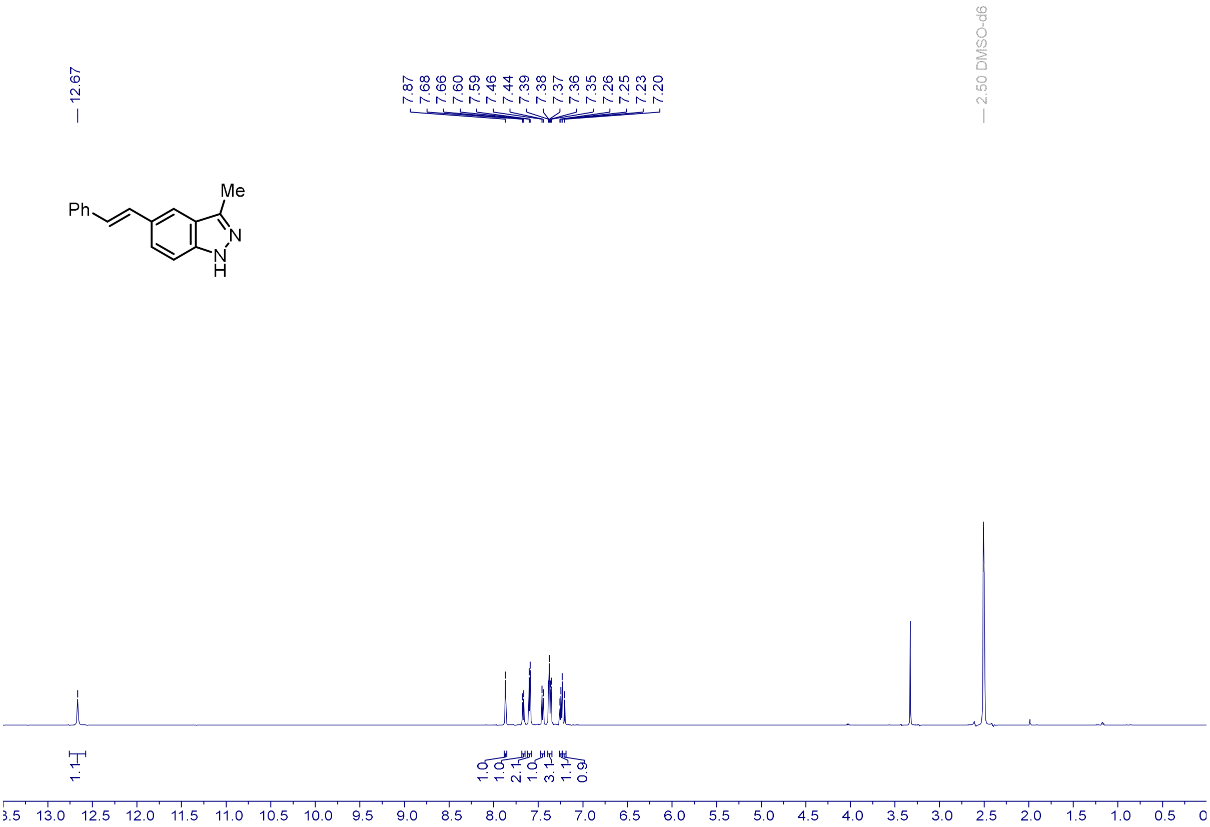

39’ – ^{13}C NMR (151 MHz, DMSO-d_6)

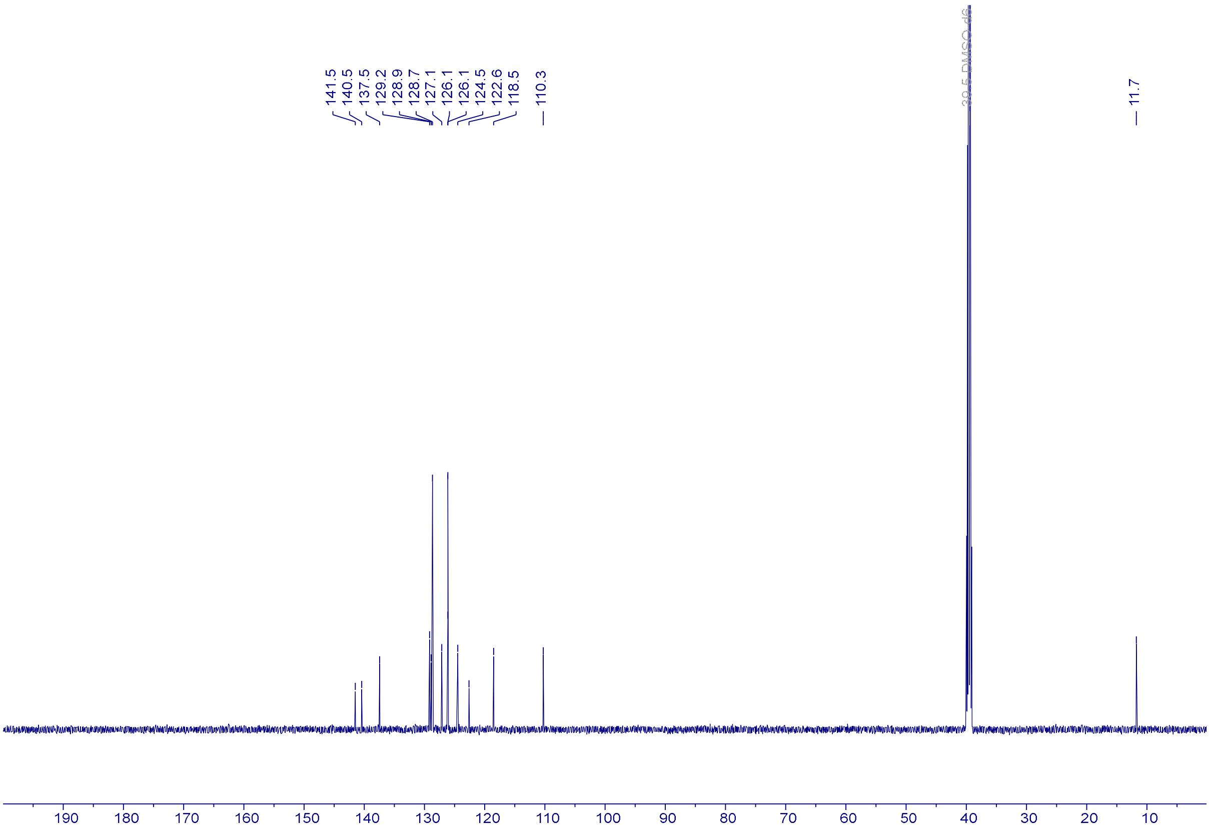

39 - ^{1}H NMR (600 MHz, $CDCl_3$)

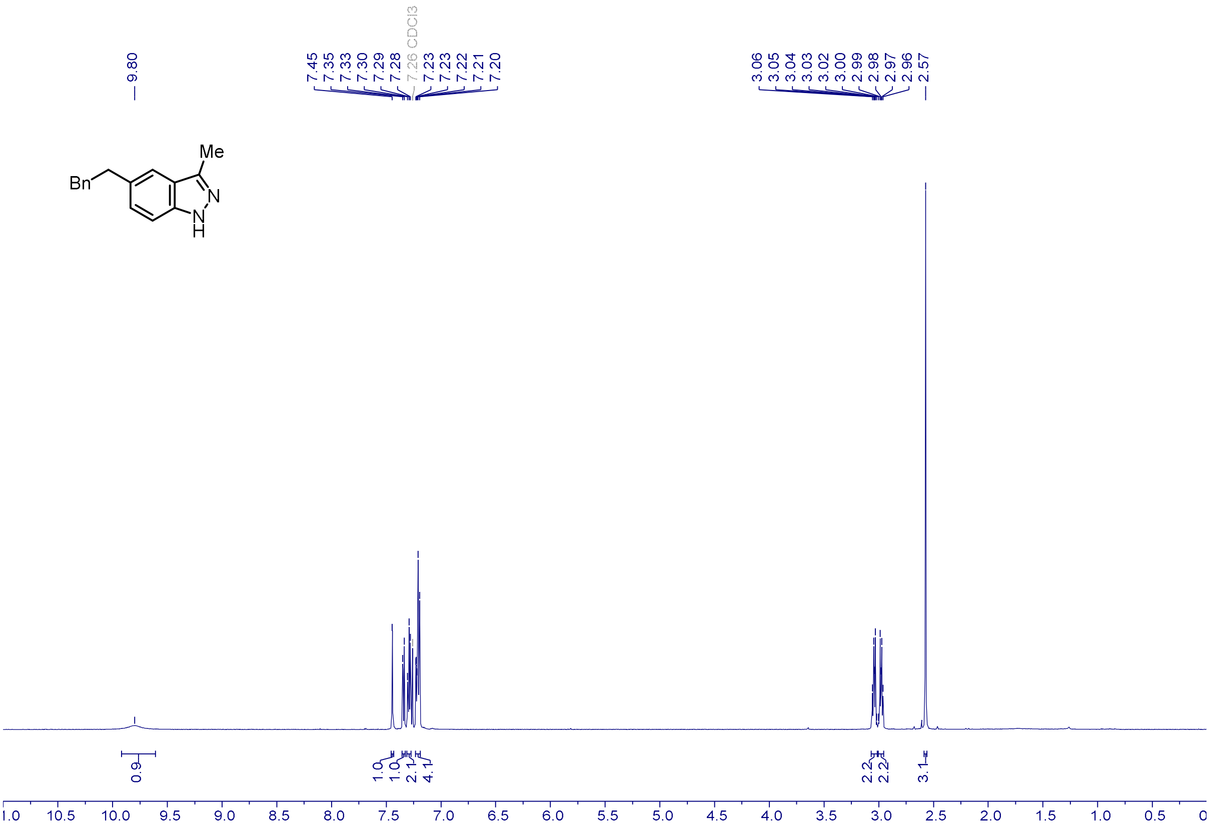

39 – ^{13}C NMR (151 MHz, $CDCl_3$)

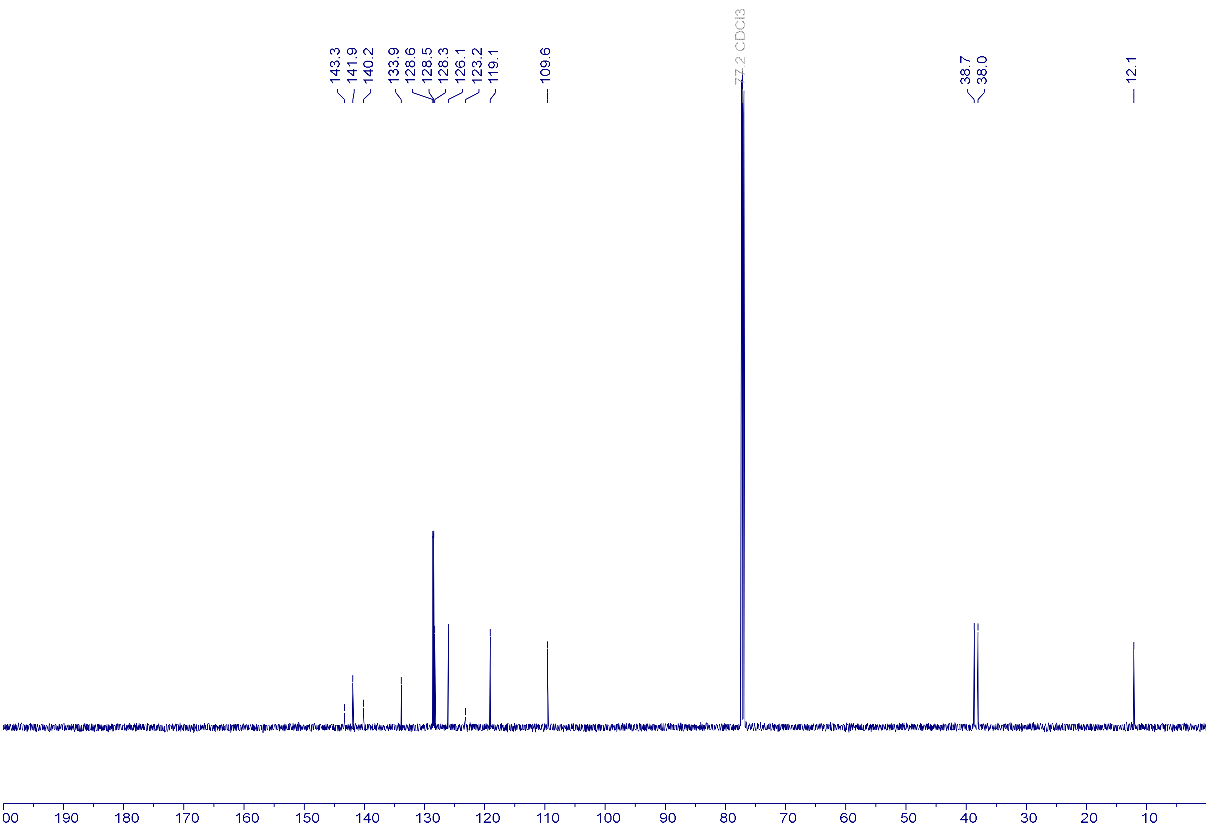

40 – ^{1}H NMR (600 MHz, $CDCl_3$)

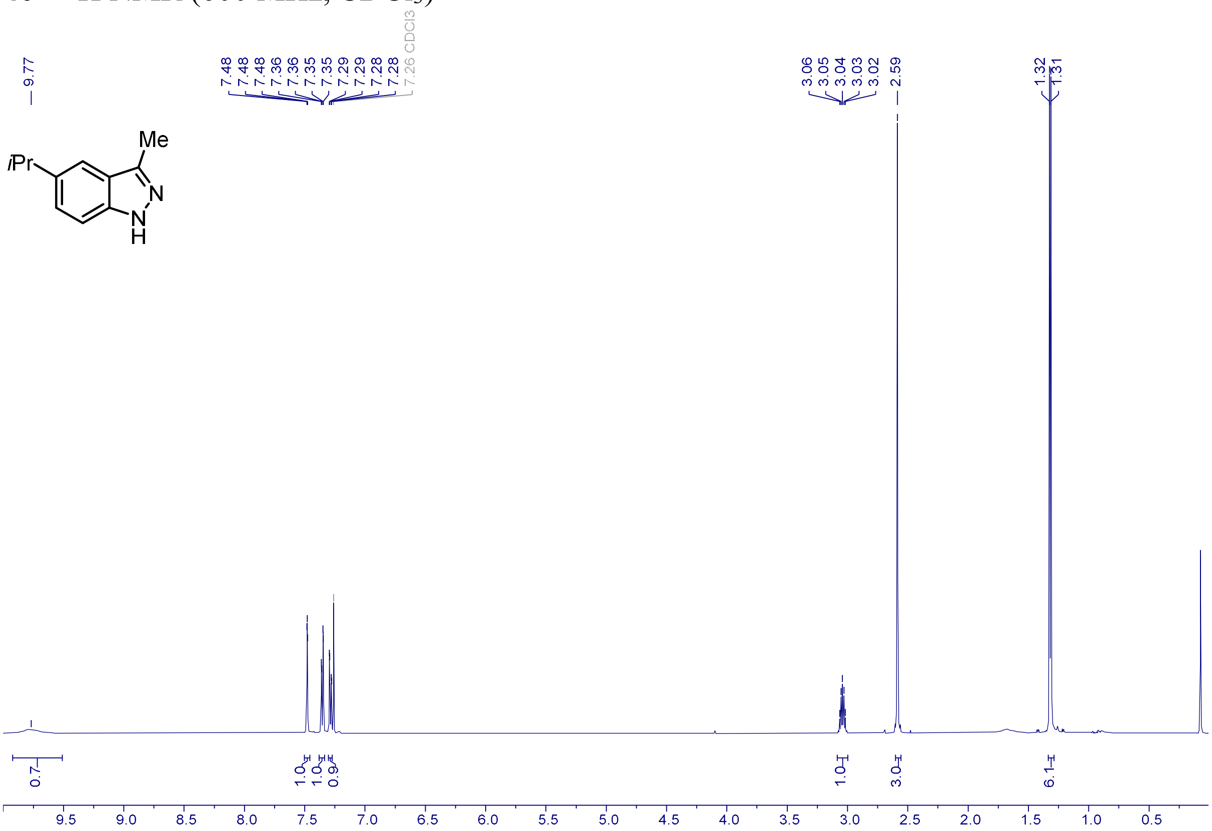

40 – ^{13}C NMR (151 MHz, $CDCl_3$)

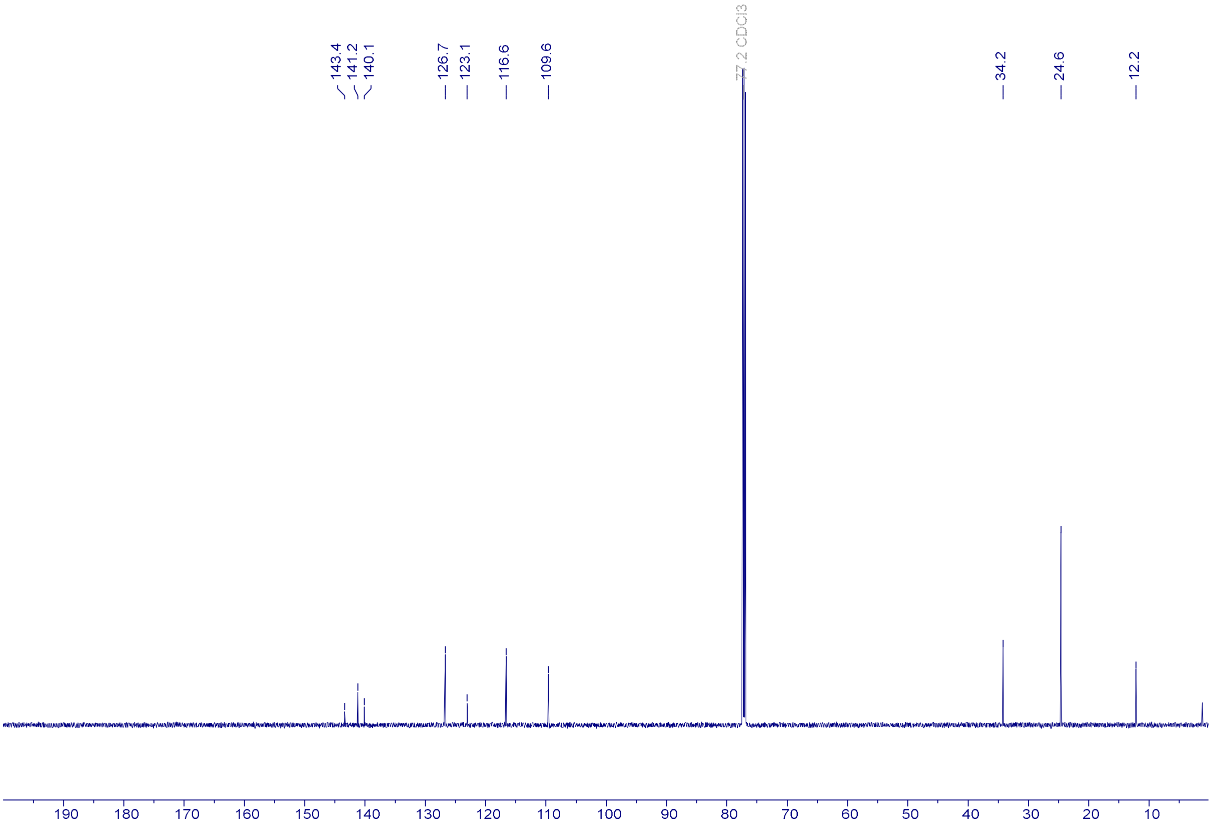

41’ - ^{1}H NMR (400 MHz, $CDCl_3$)

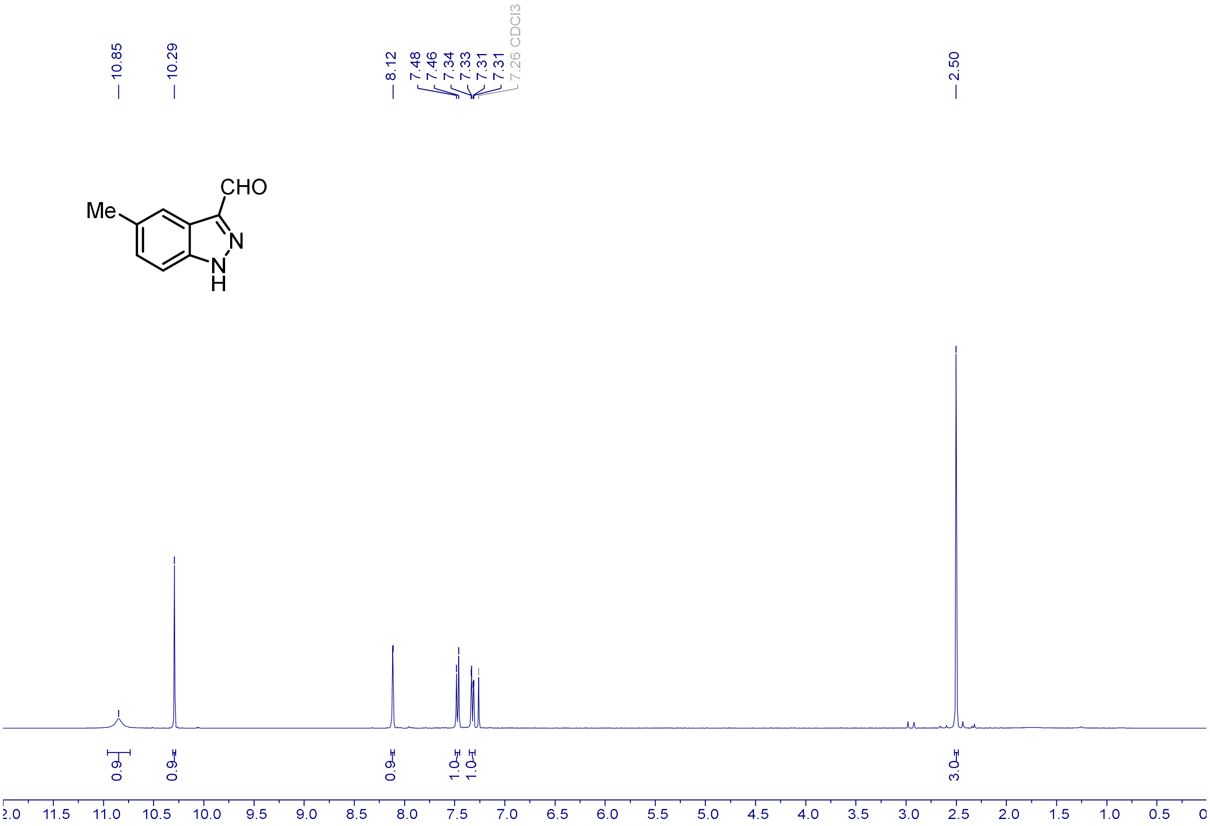

41’ - ^{13}C NMR (101 MHz, $CDCl_3$)

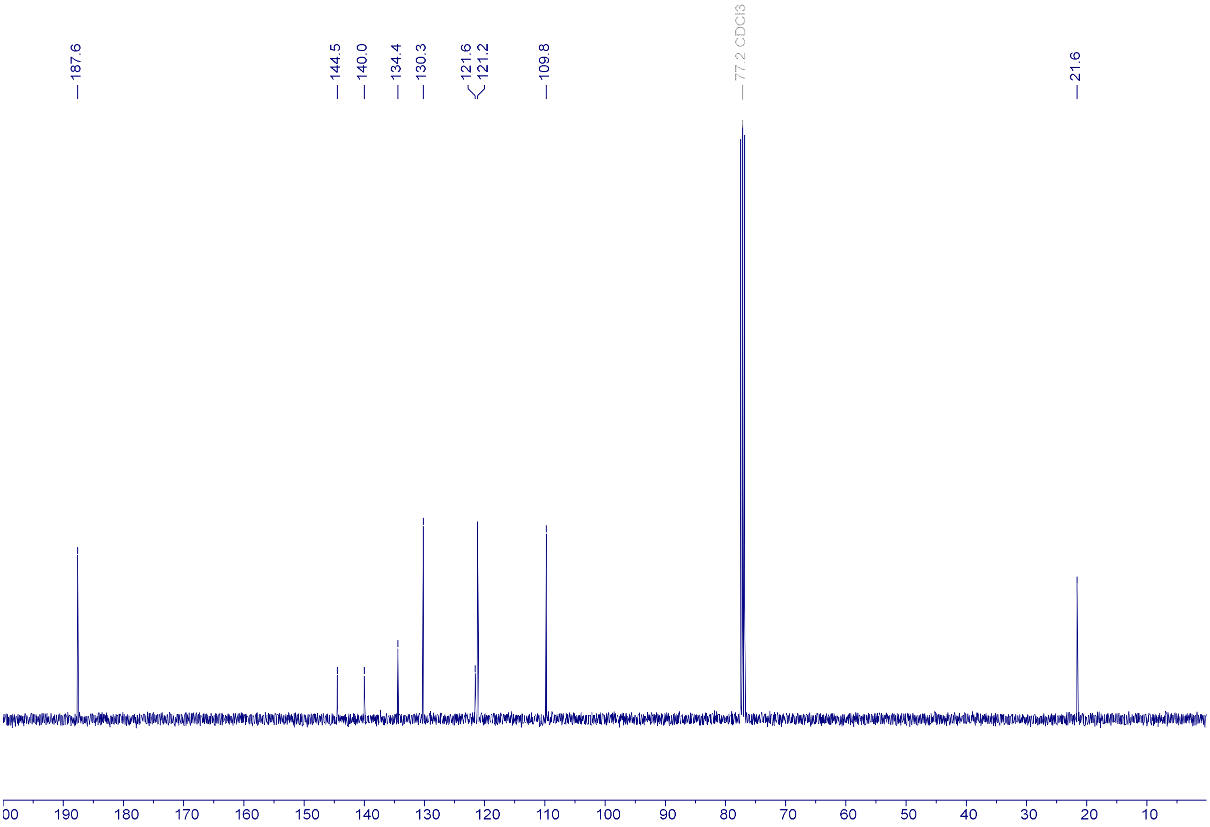

41 – ^{1}H NMR (600 MHz, $CDCl_3$)

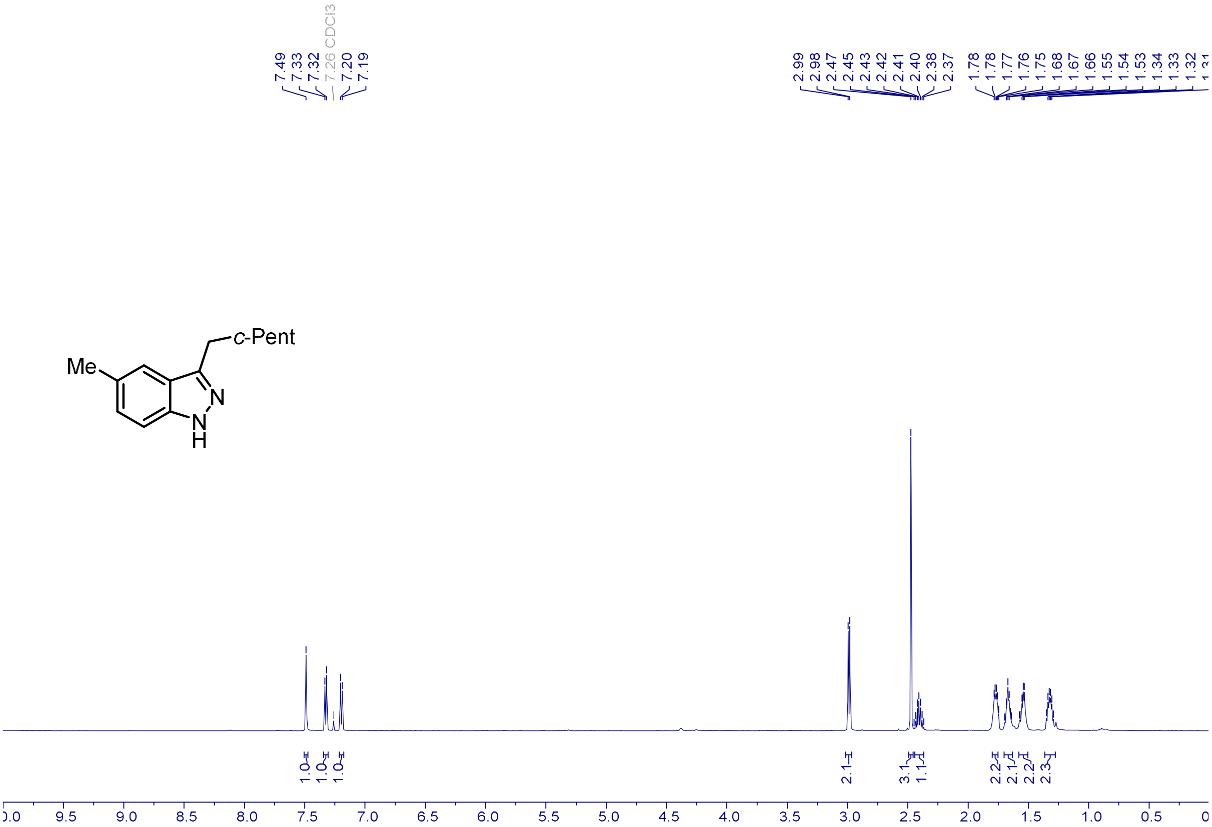

41 – ^{13}C NMR (151 MHz, $CDCl_3$)

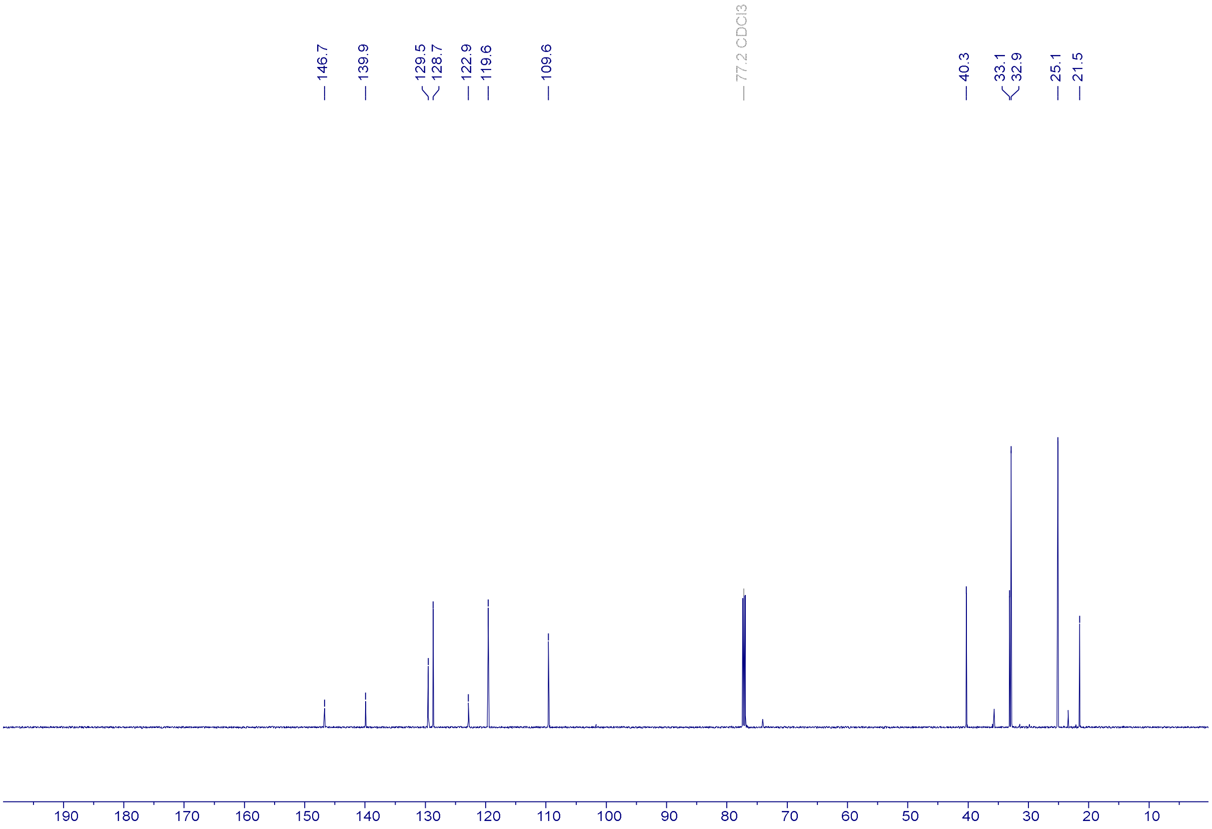

42 – ^{1}H NMR (600 MHz, $CDCl_3$)

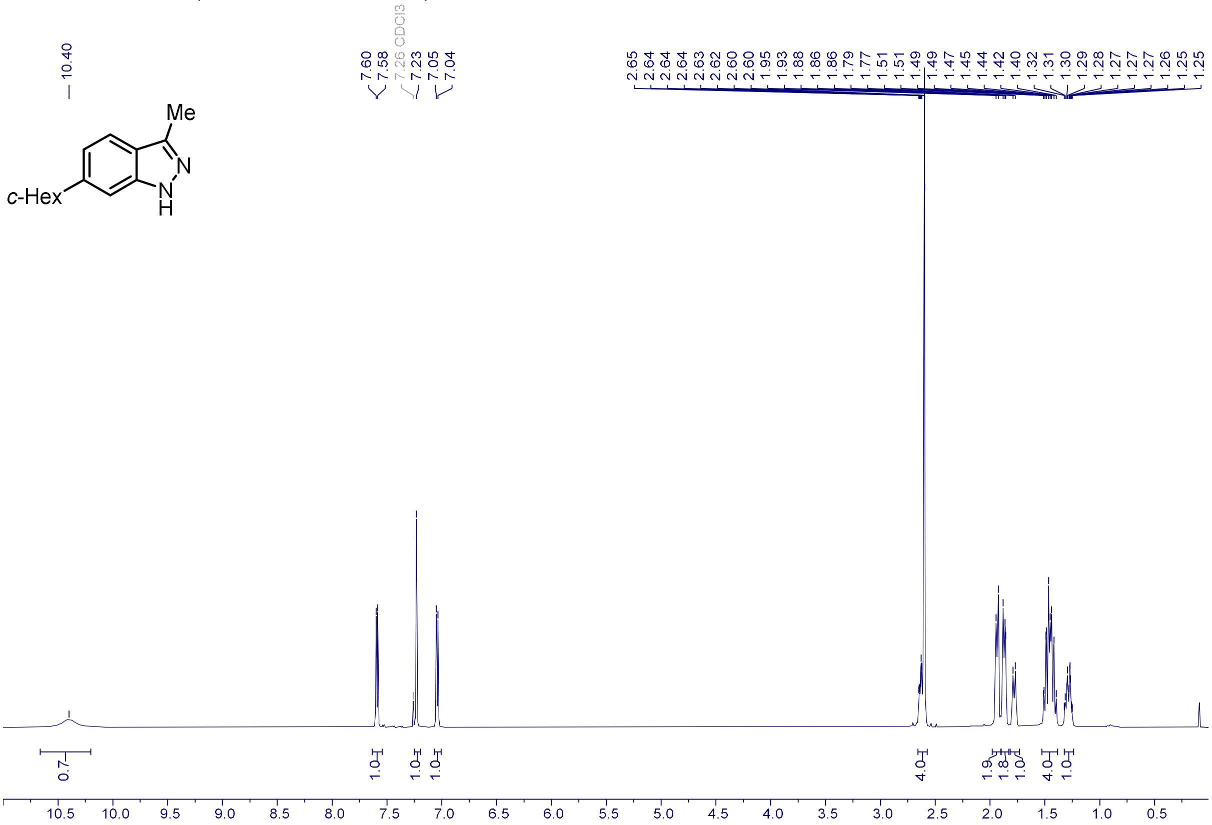

42 – ^{13}C NMR (151 MHz, $CDCl_3$)

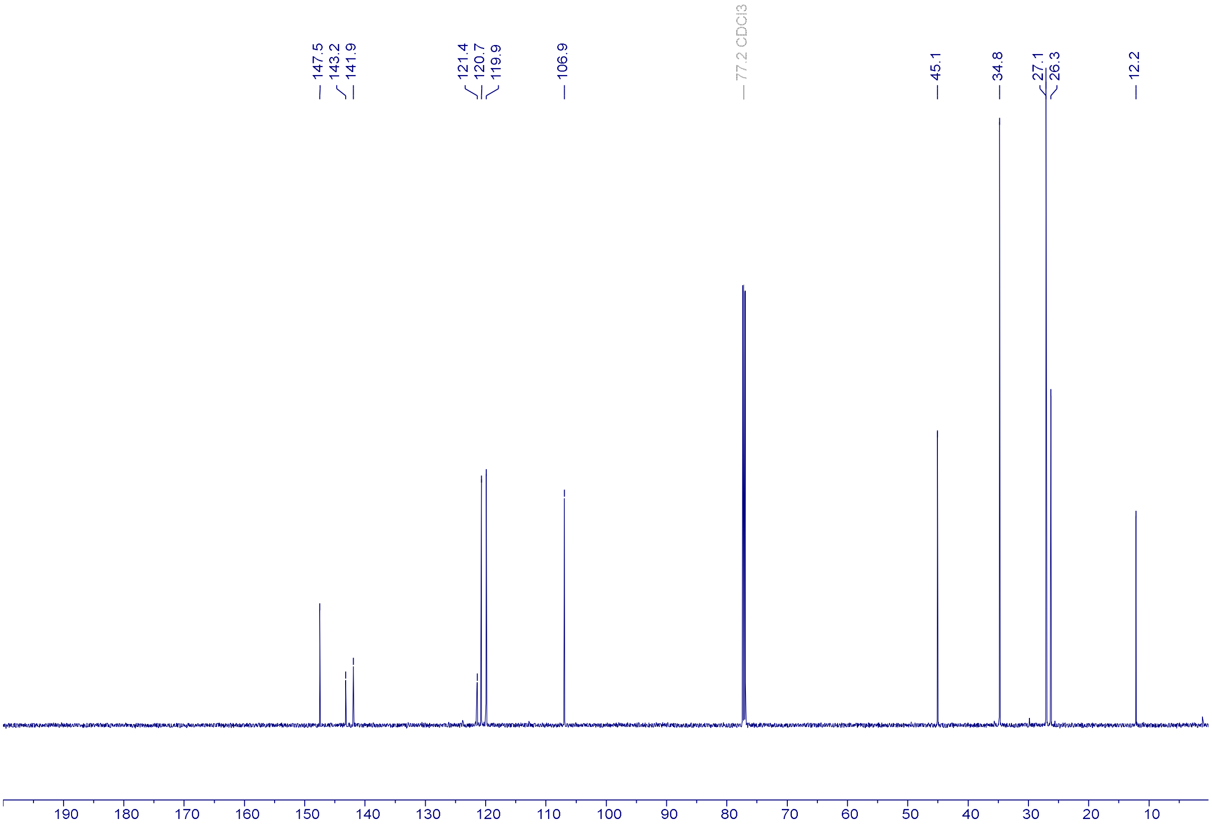

44 – ^{1}H NMR (600 MHz, $CDCl_3$)

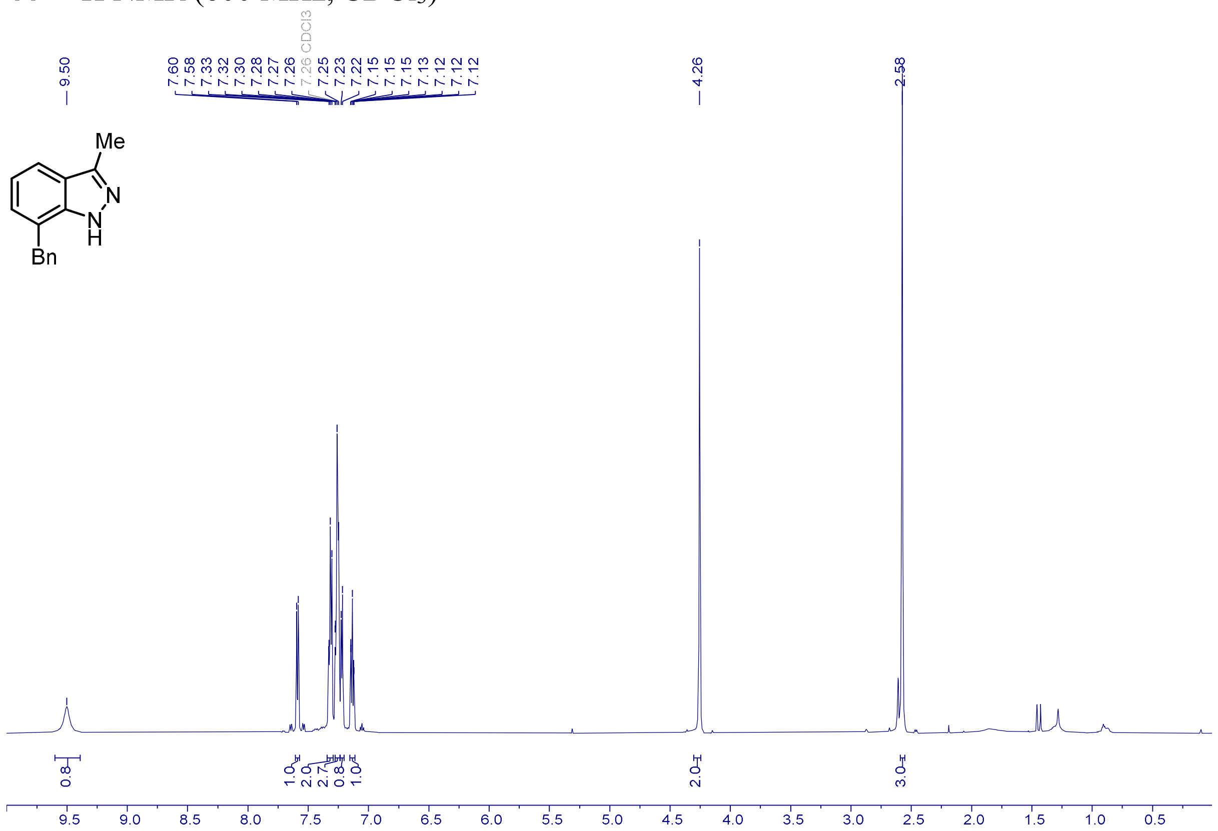

44 – ^{13}C NMR (151 MHz, $CDCl_3$)

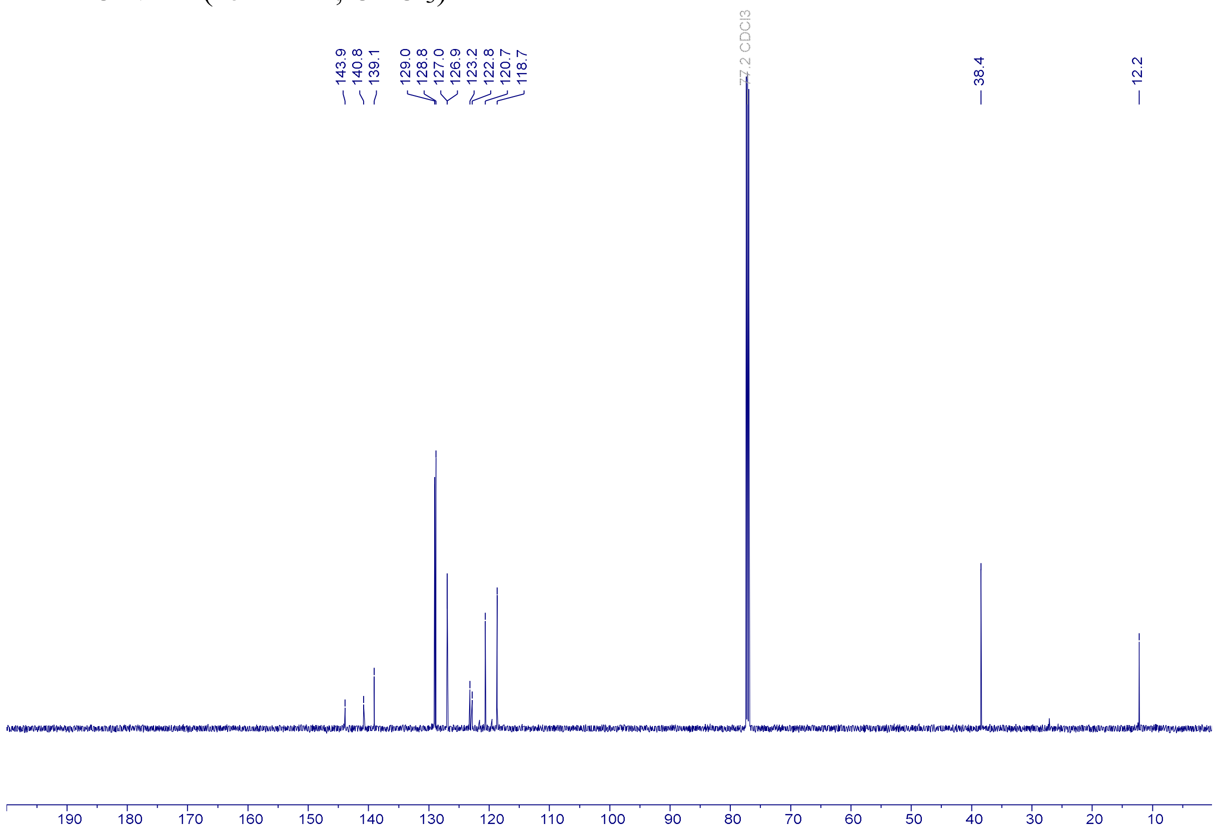

46 – ^{1}H NMR (600 MHz, $CDCl_3$)

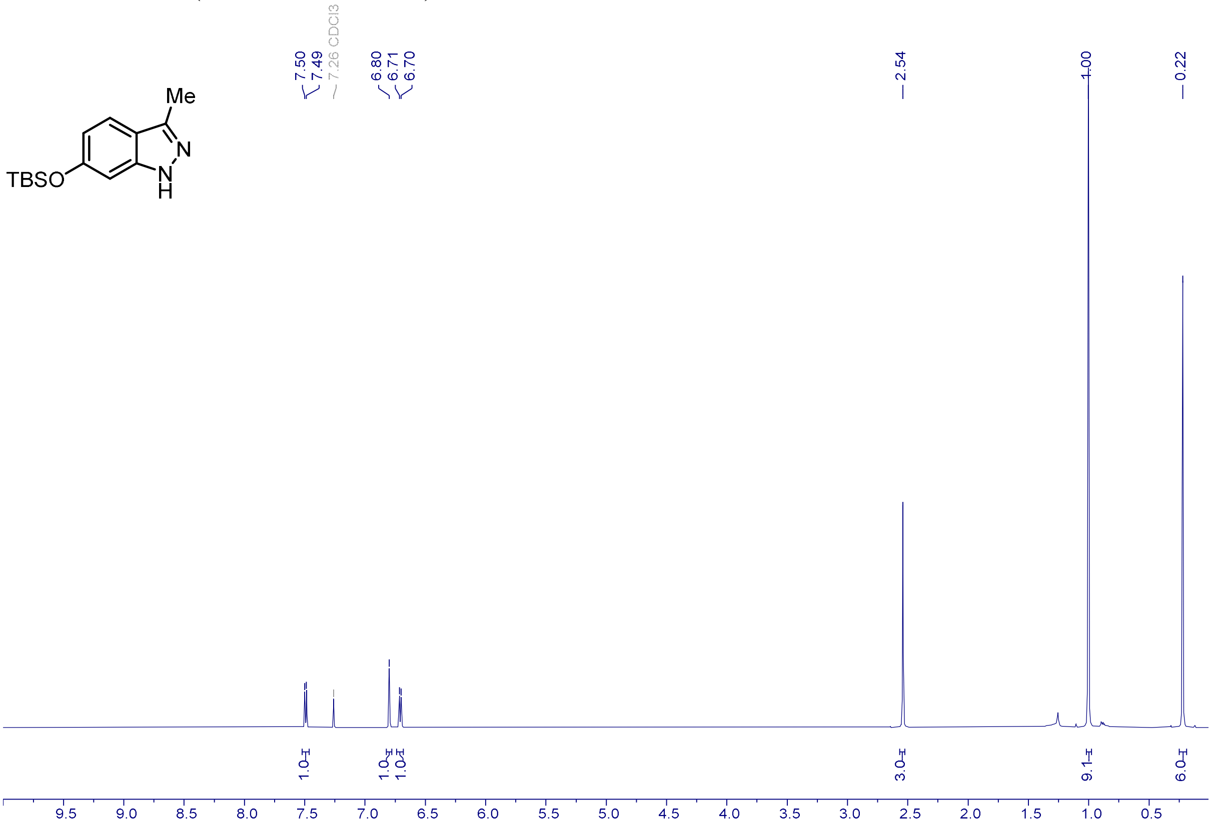

46 – ^{13}C NMR (151 MHz, $CDCl_3$)

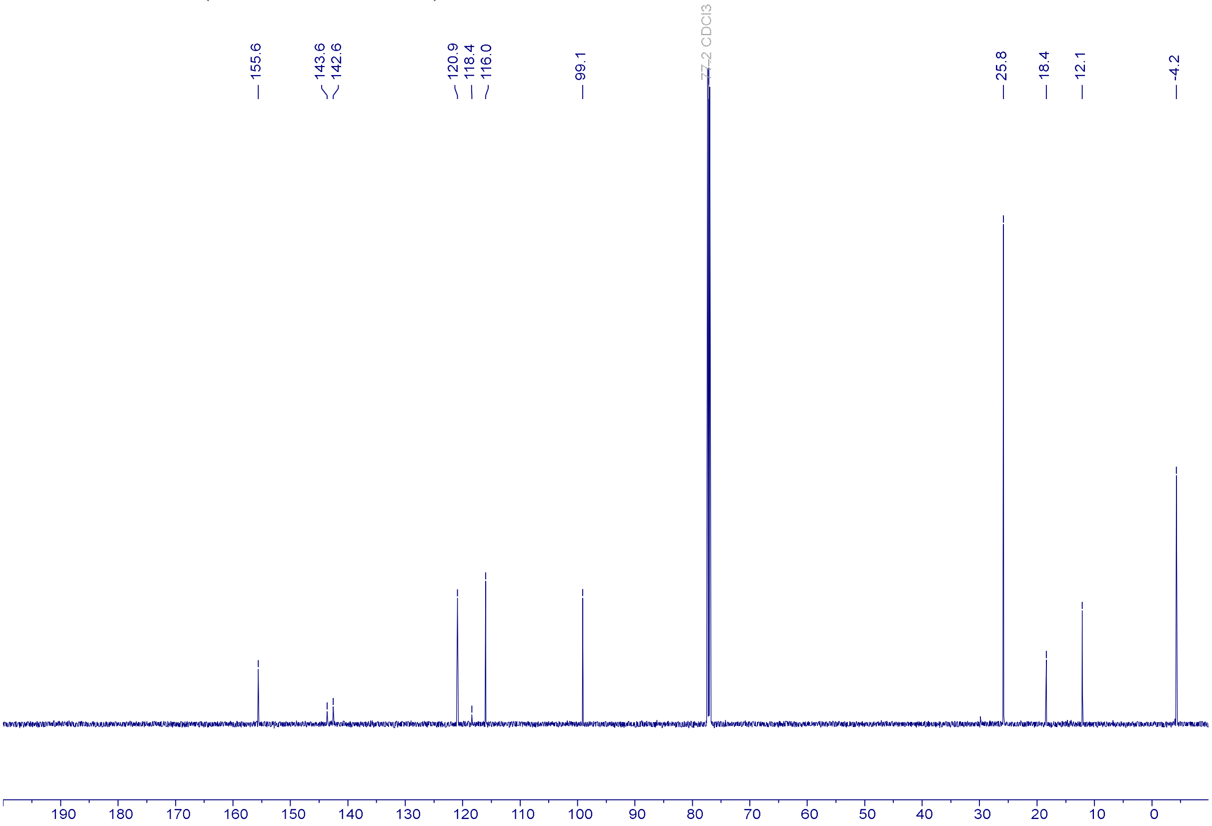

47 – ^{1}H NMR (600 MHz, DMSO-d_6)

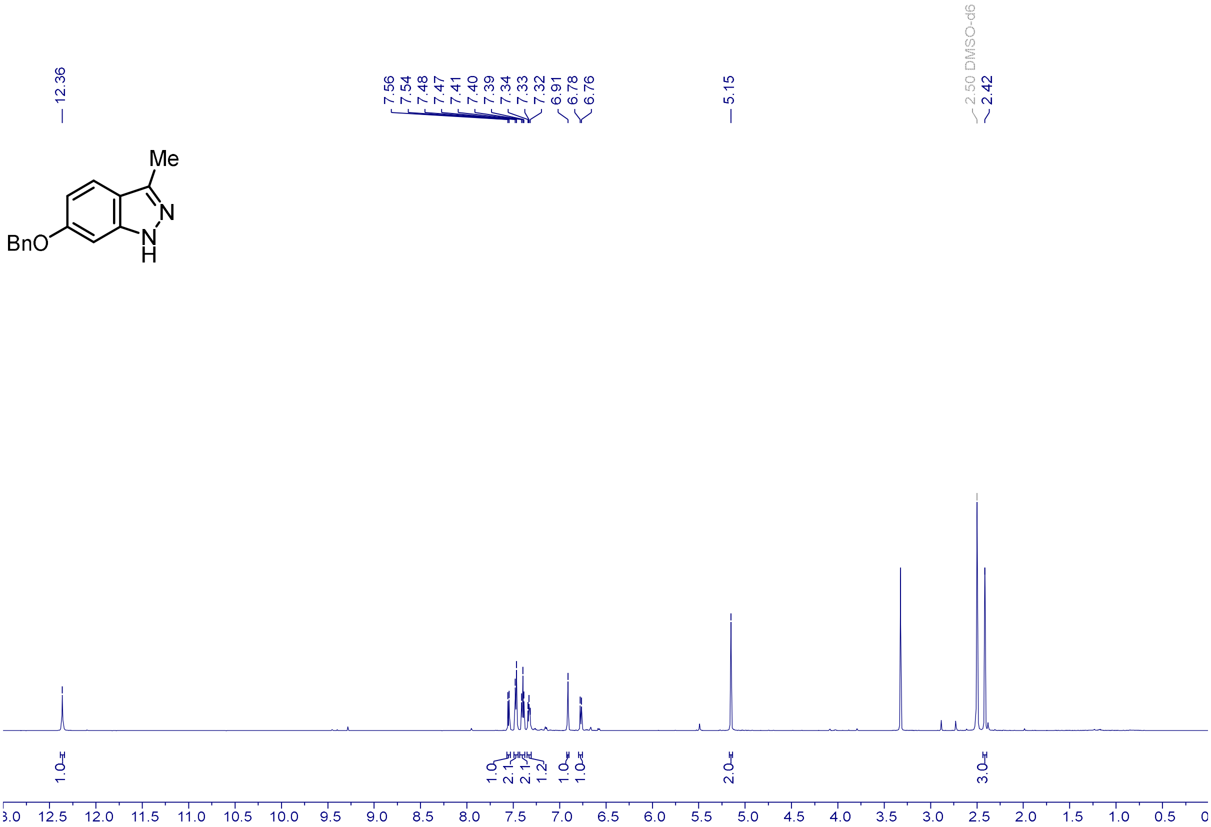

47 – ^{13}C NMR (151 MHz, DMSO-d_6)

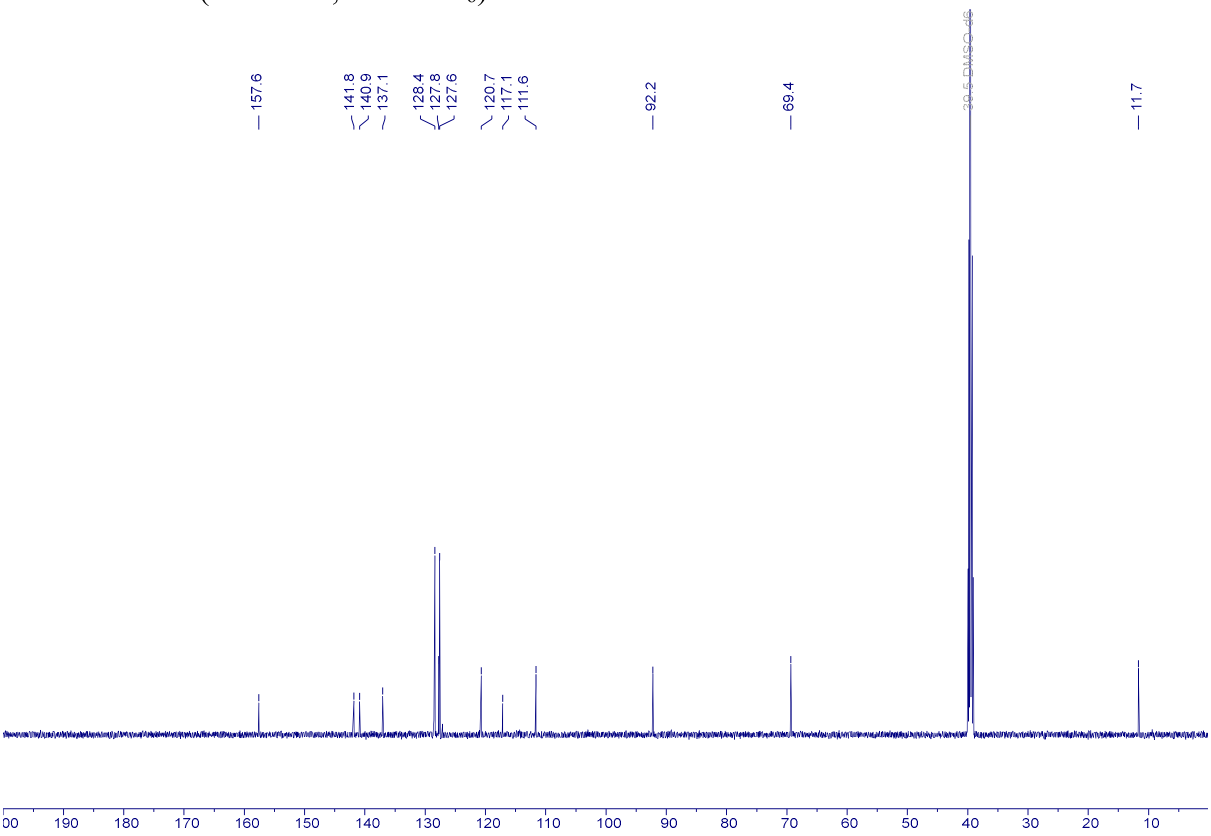

48 – ^{1}H NMR (400 MHz, $CDCl_3$)

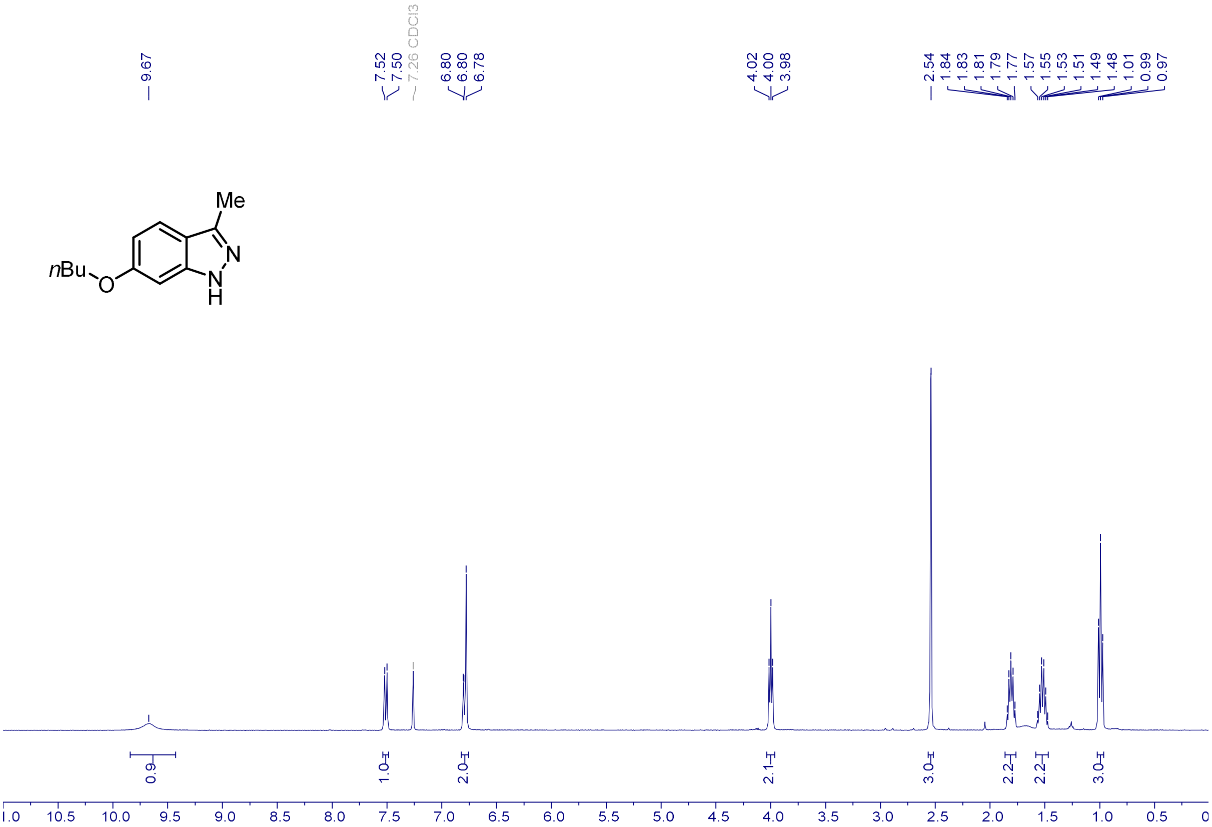

48 – ^{13}C NMR (101 MHz, $CDCl_3$)

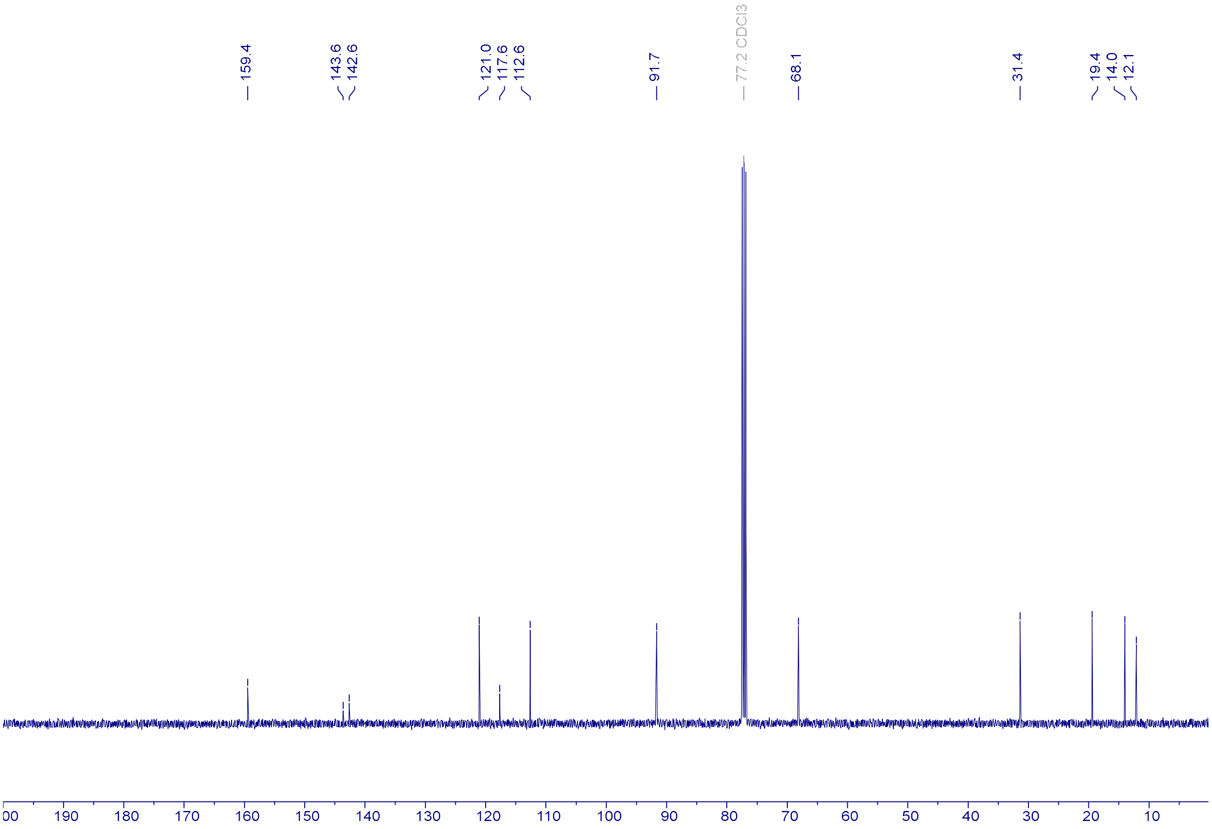

49 – ^{1}H NMR (400 MHz, $CDCl_3$)

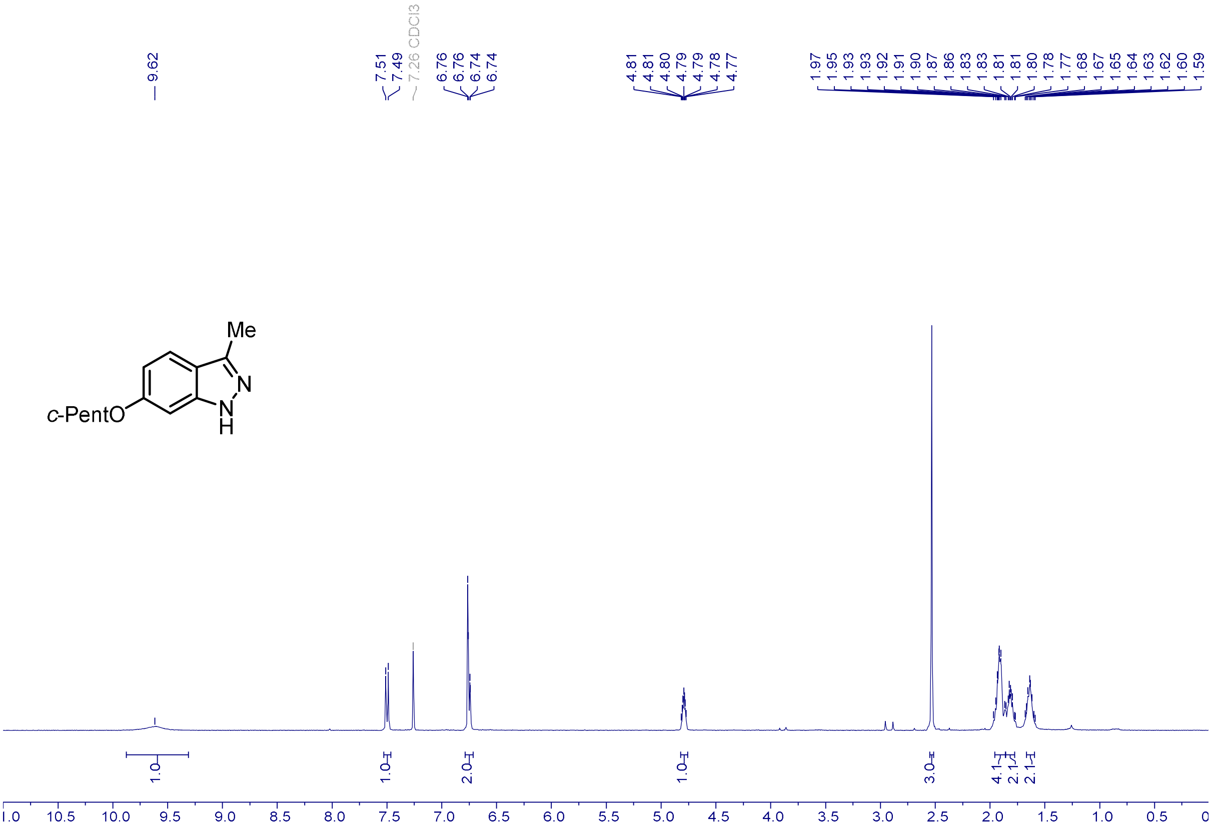

49 – ^{13}C NMR (101 MHz, $CDCl_3$)

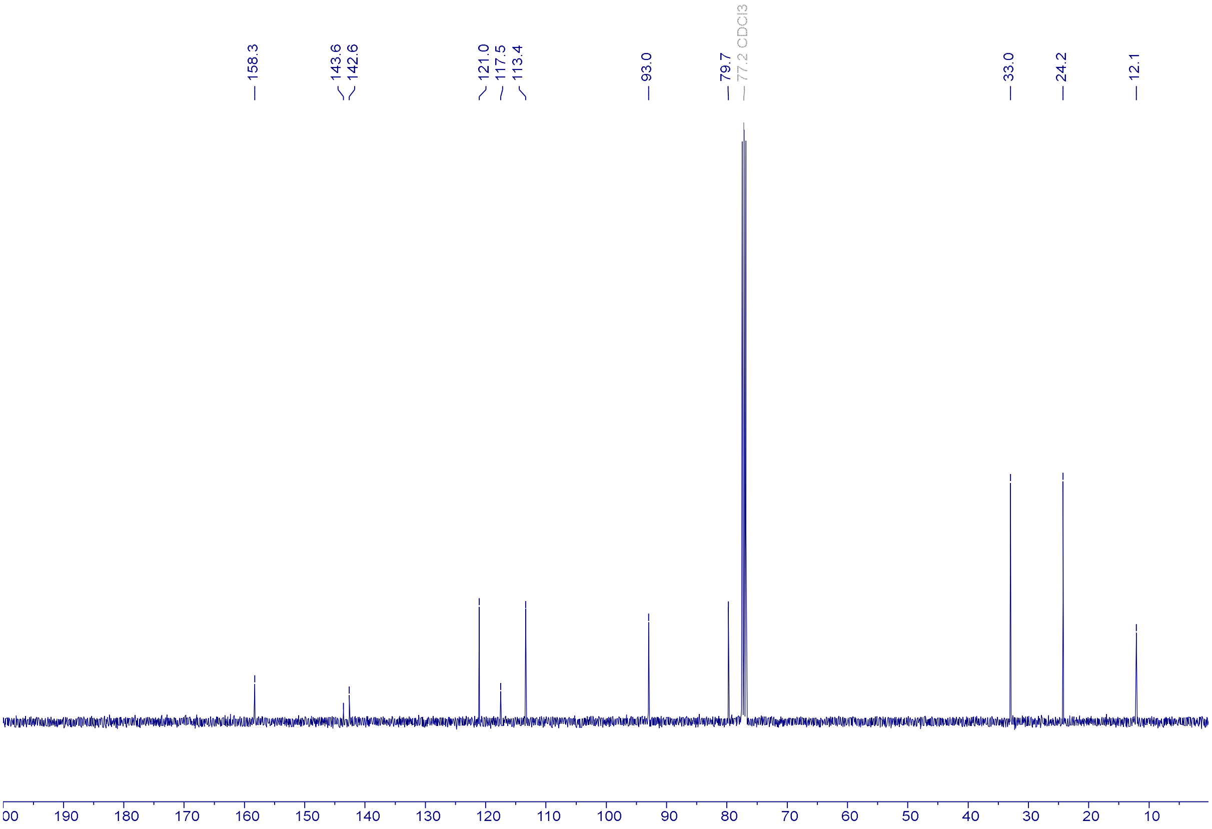

55 – ^{1}H NMR (400 MHz, $CDCl_3$)

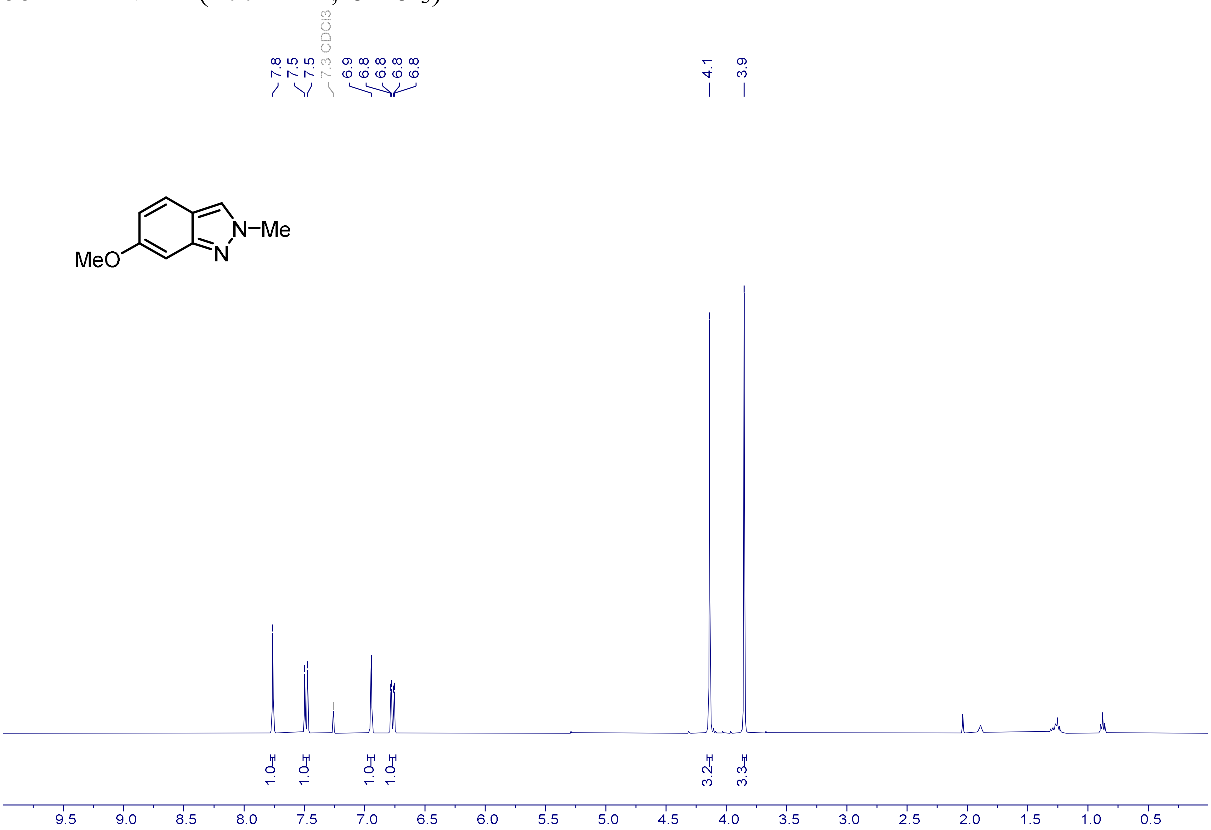

55 – ^{13}C NMR (101 MHz, $CDCl_3$)

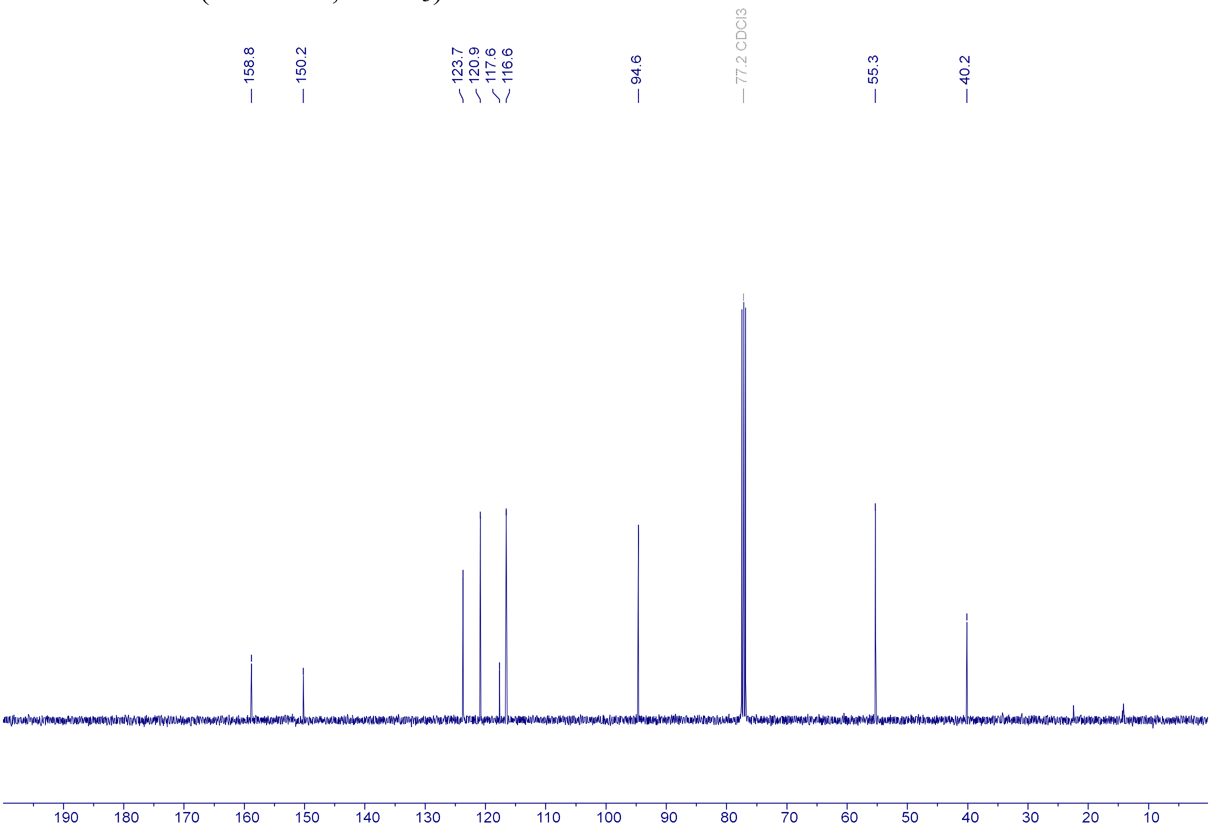

56 – ^{1}H NMR (400 MHz, $CDCl_3$)

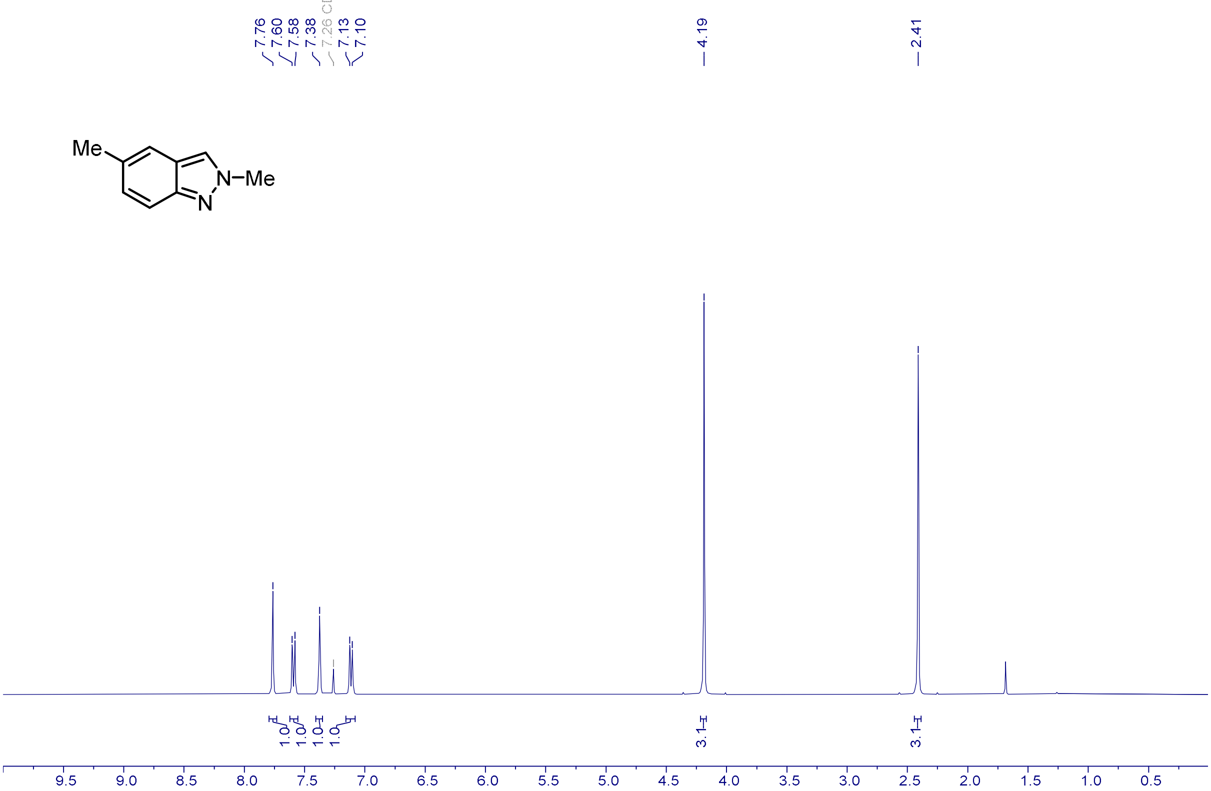

56 – ^{13}C NMR (101 MHz, $CDCl_3$)

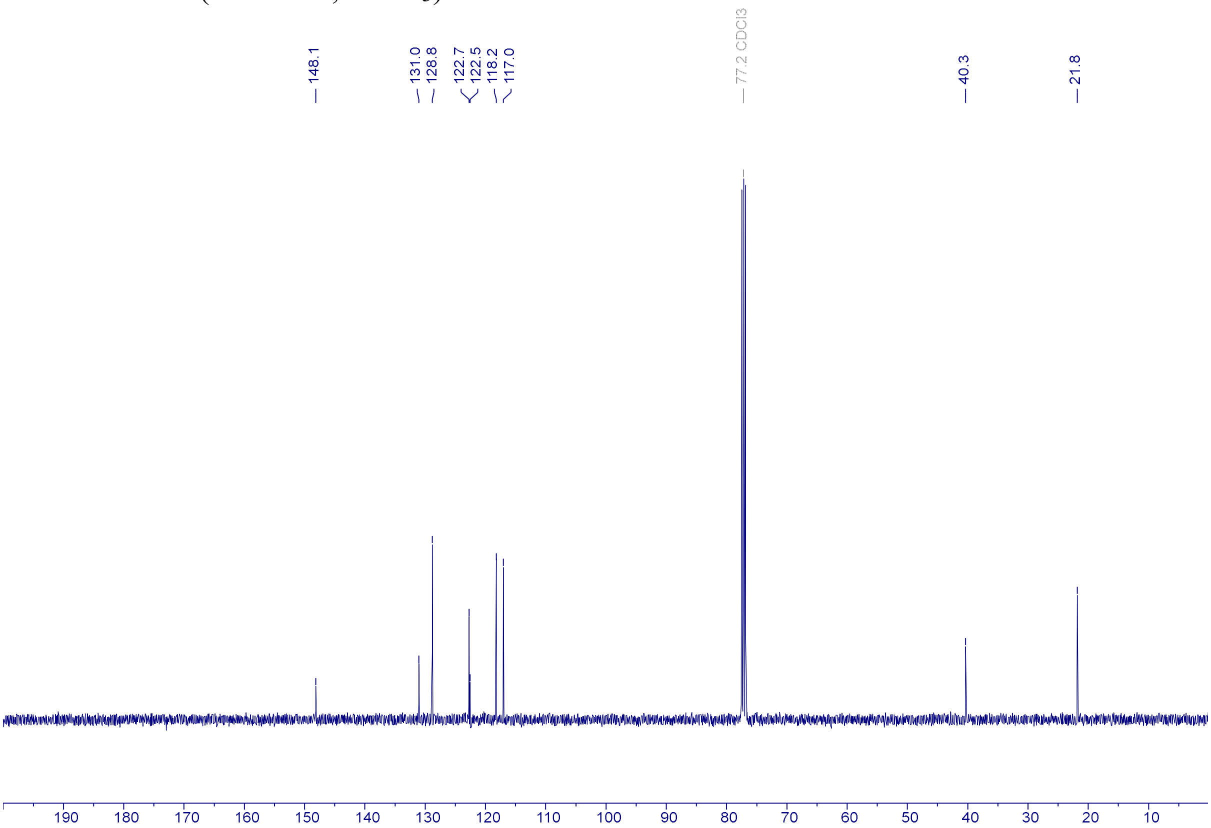

58 – ^{1}H NMR (600 MHz, $CDCl_3$)

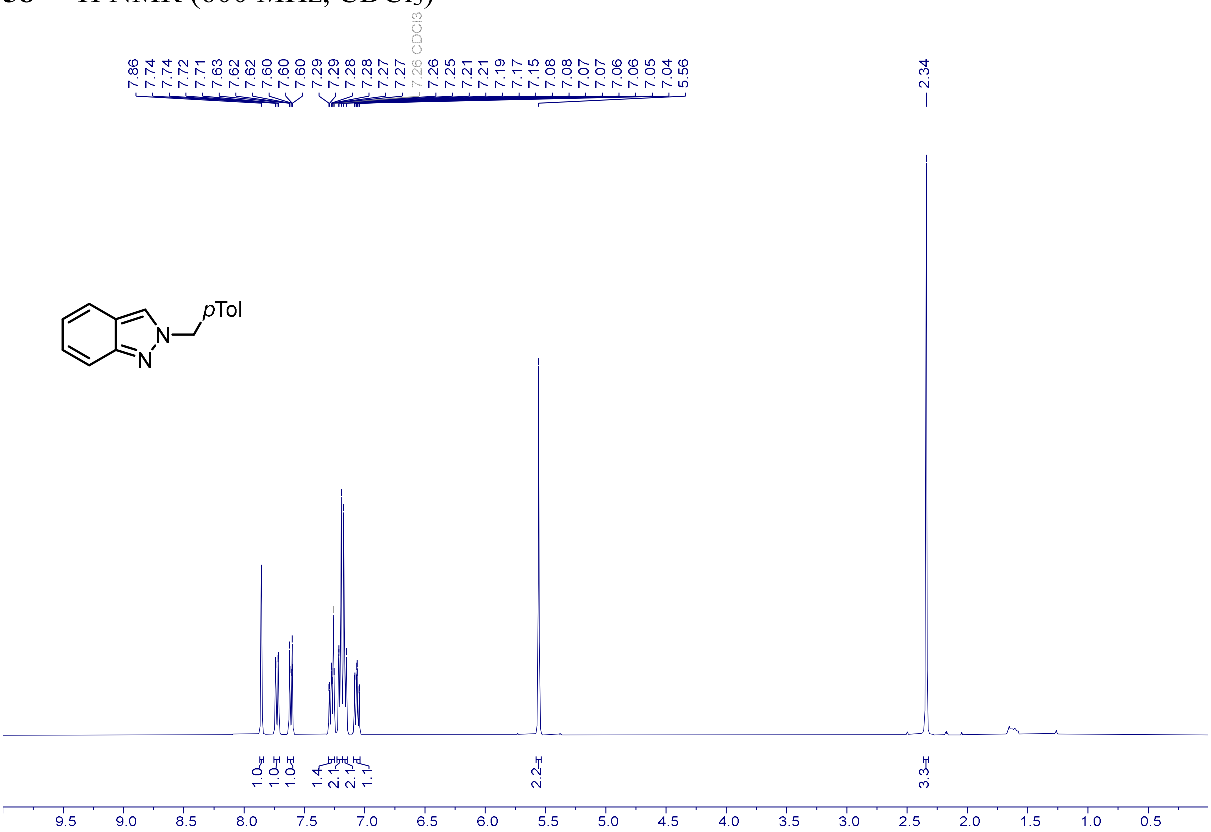

58 – ^{13}C NMR (151 MHz, $CDCl_3$)

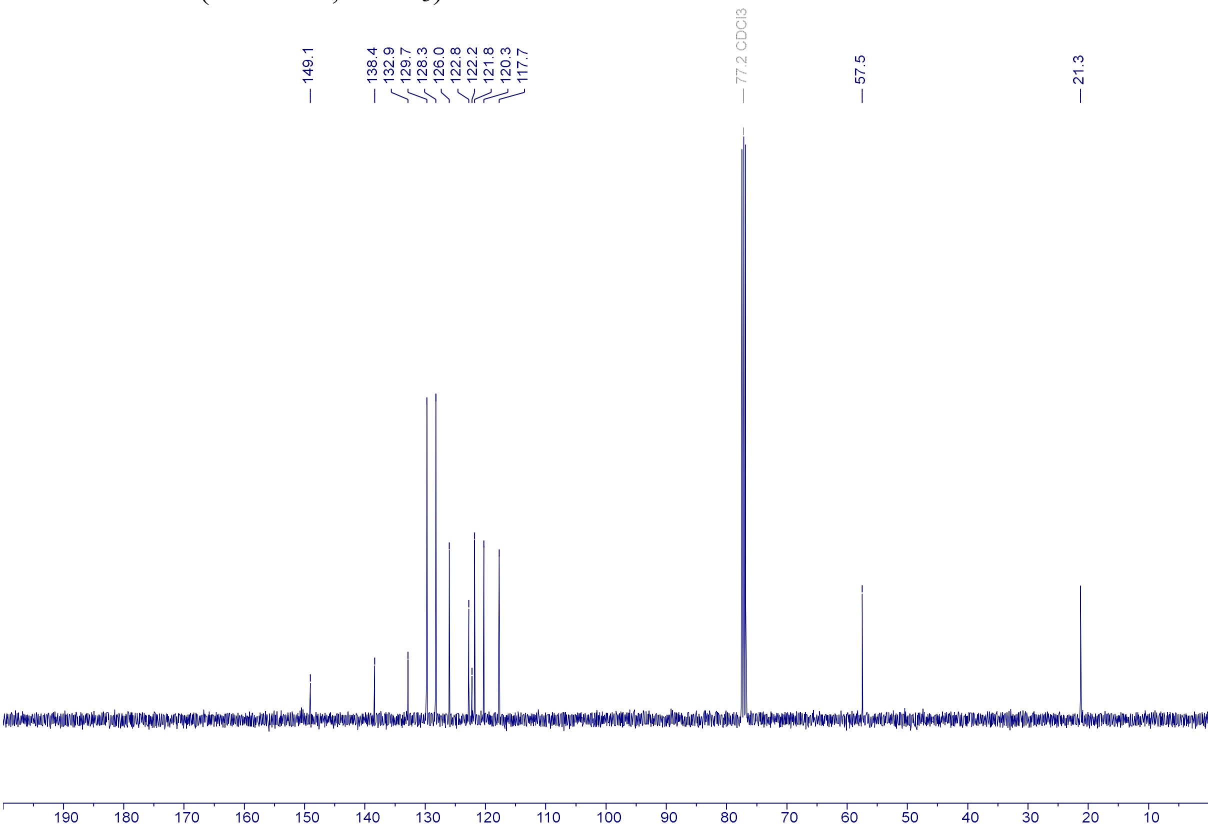

60 – ^{1}H NMR (400 MHz, $CDCl_3$)

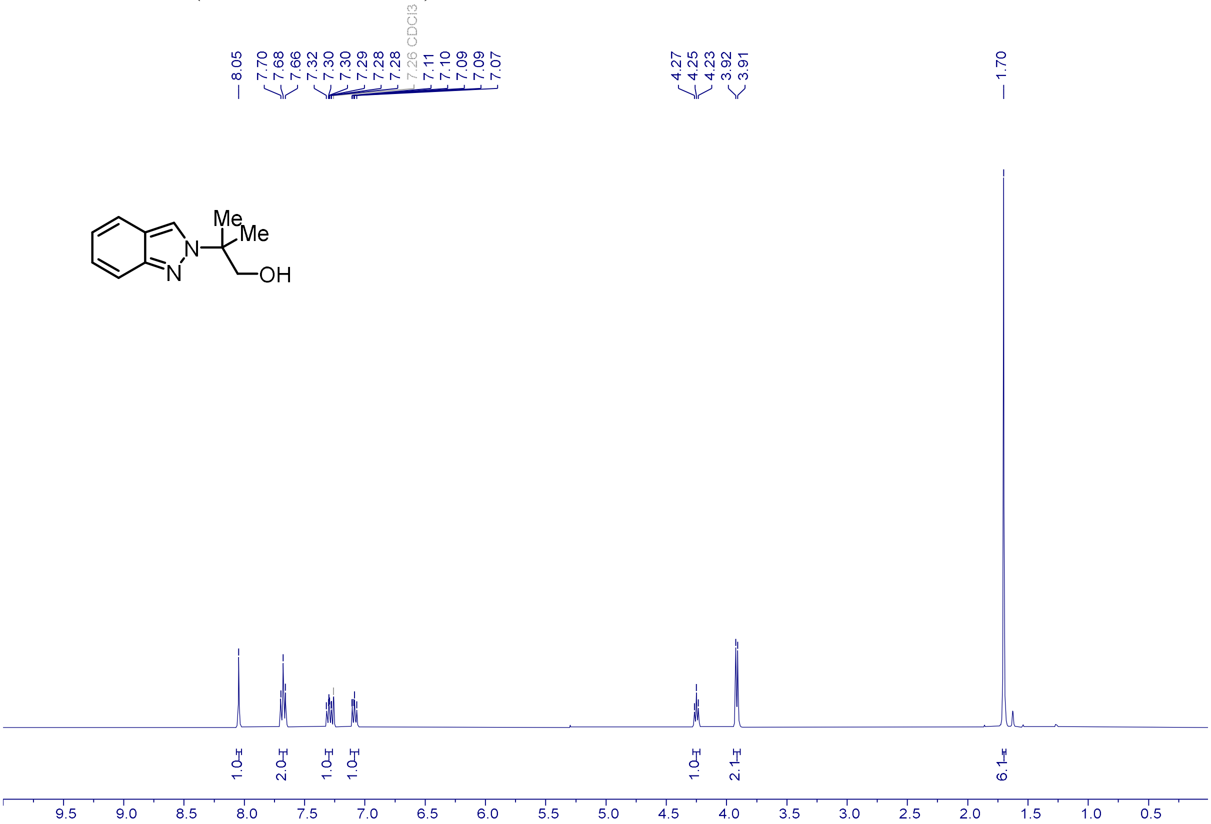

60 – ^{13}C NMR (101 MHz, $CDCl_3$)

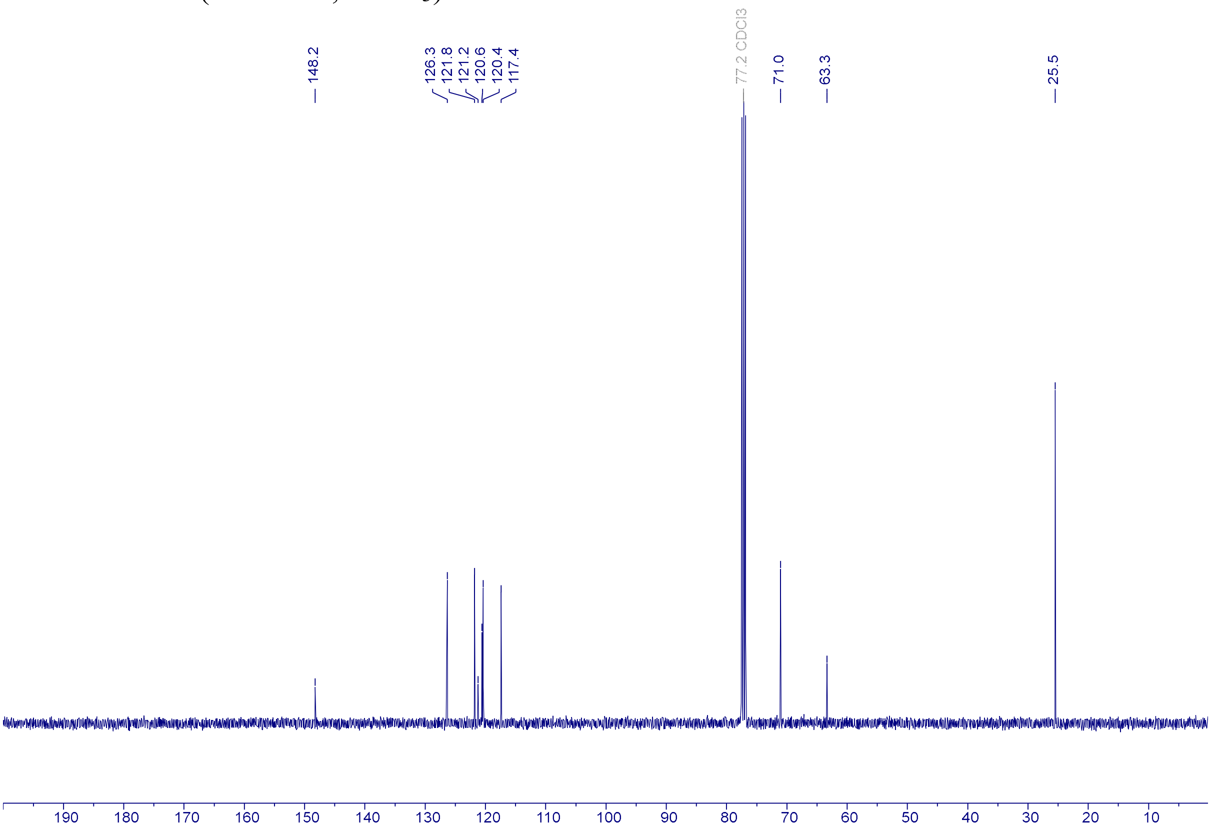

61 – ^{1}H NMR (400 MHz, $CDCl_3$)

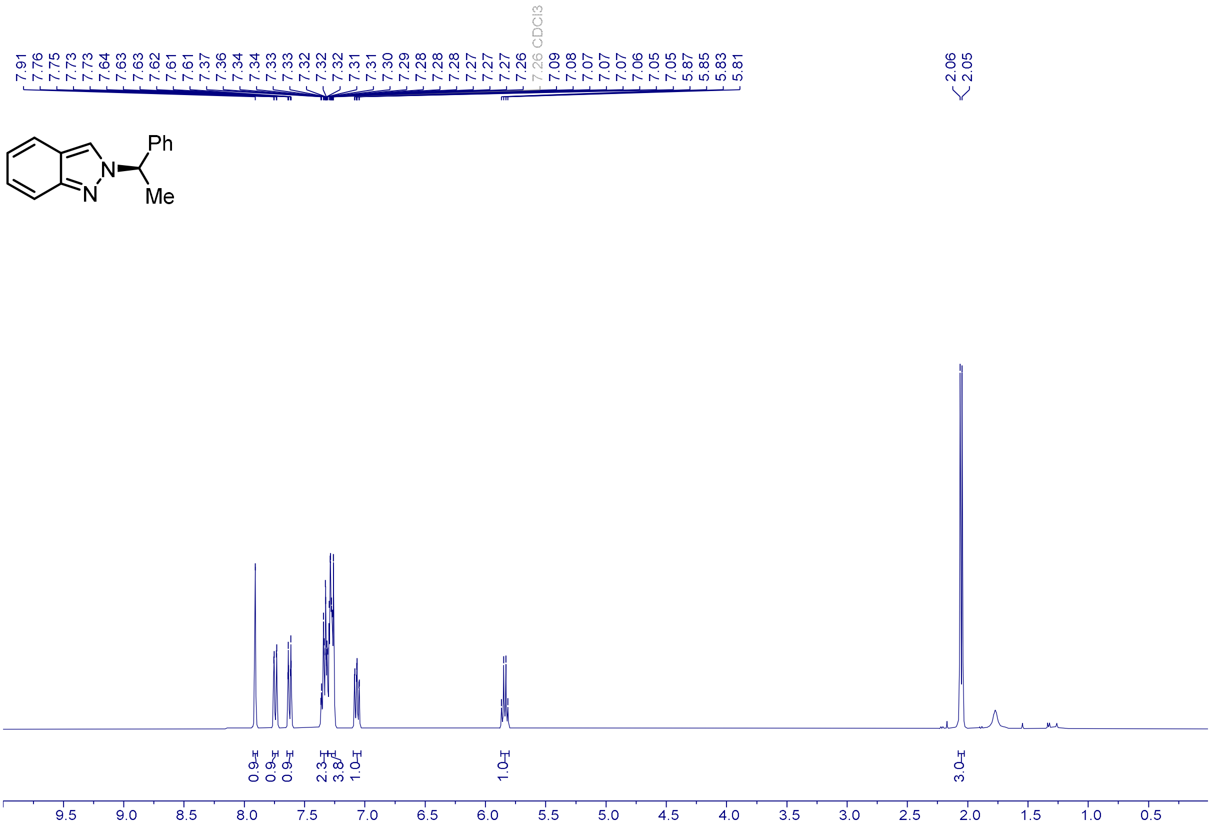

61 – ^{13}C NMR (101 MHz, $CDCl_3$)

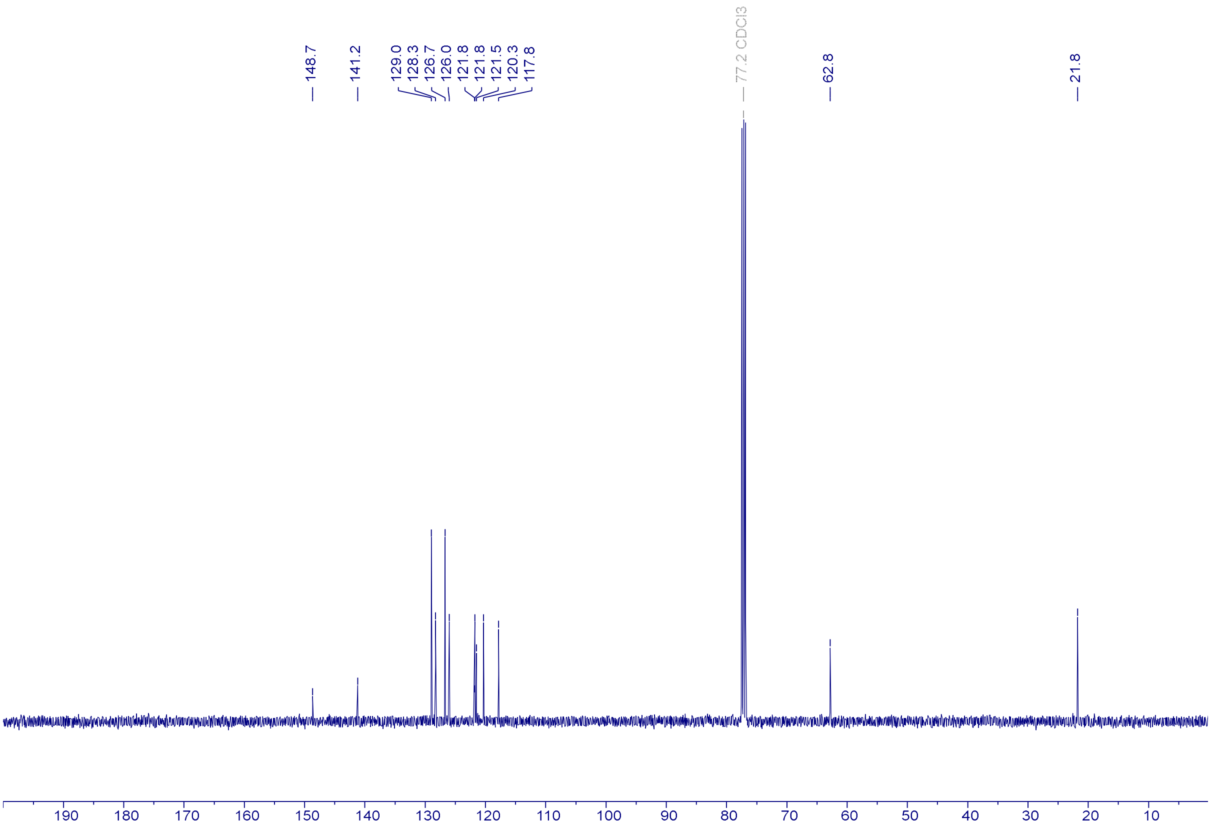

61-rac - ^{1}H NMR (400 MHz, $CDCl_3$)

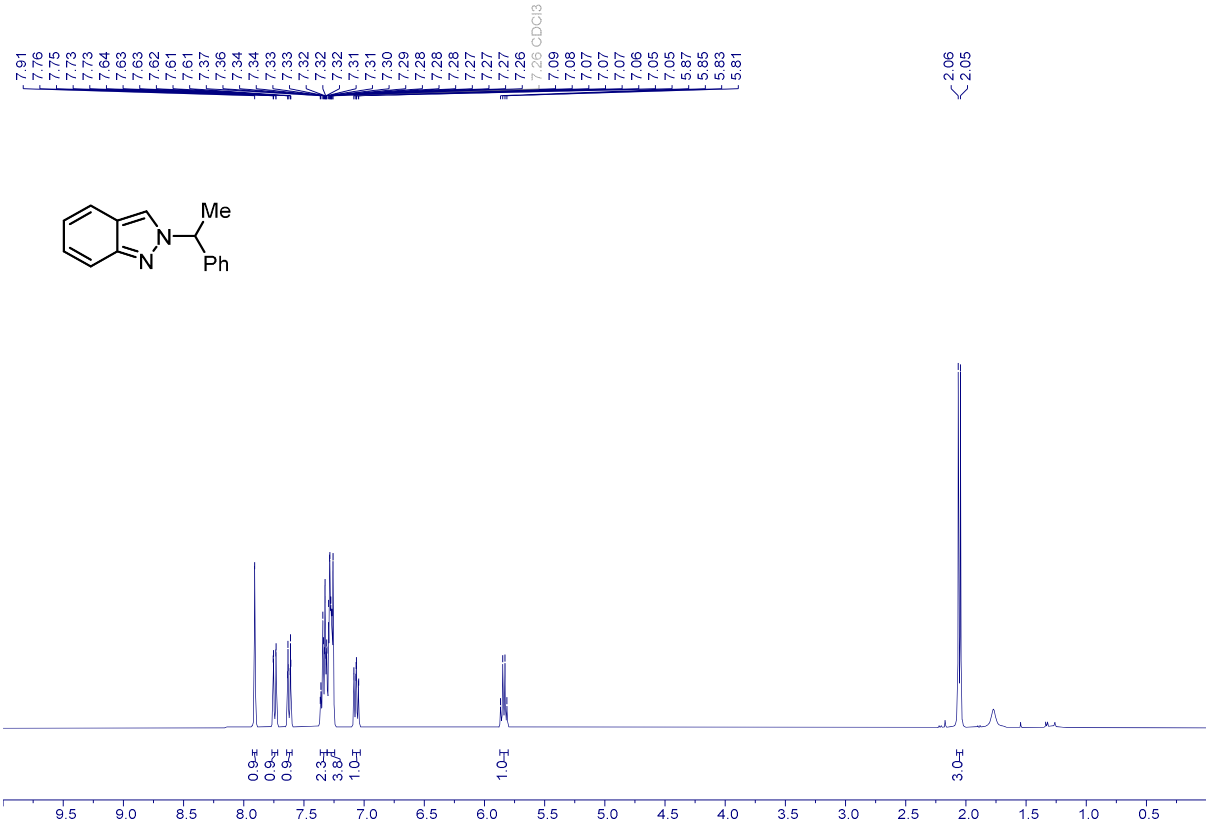

61-rac - ^{13}C NMR (101 MHz, $CDCl_3$)

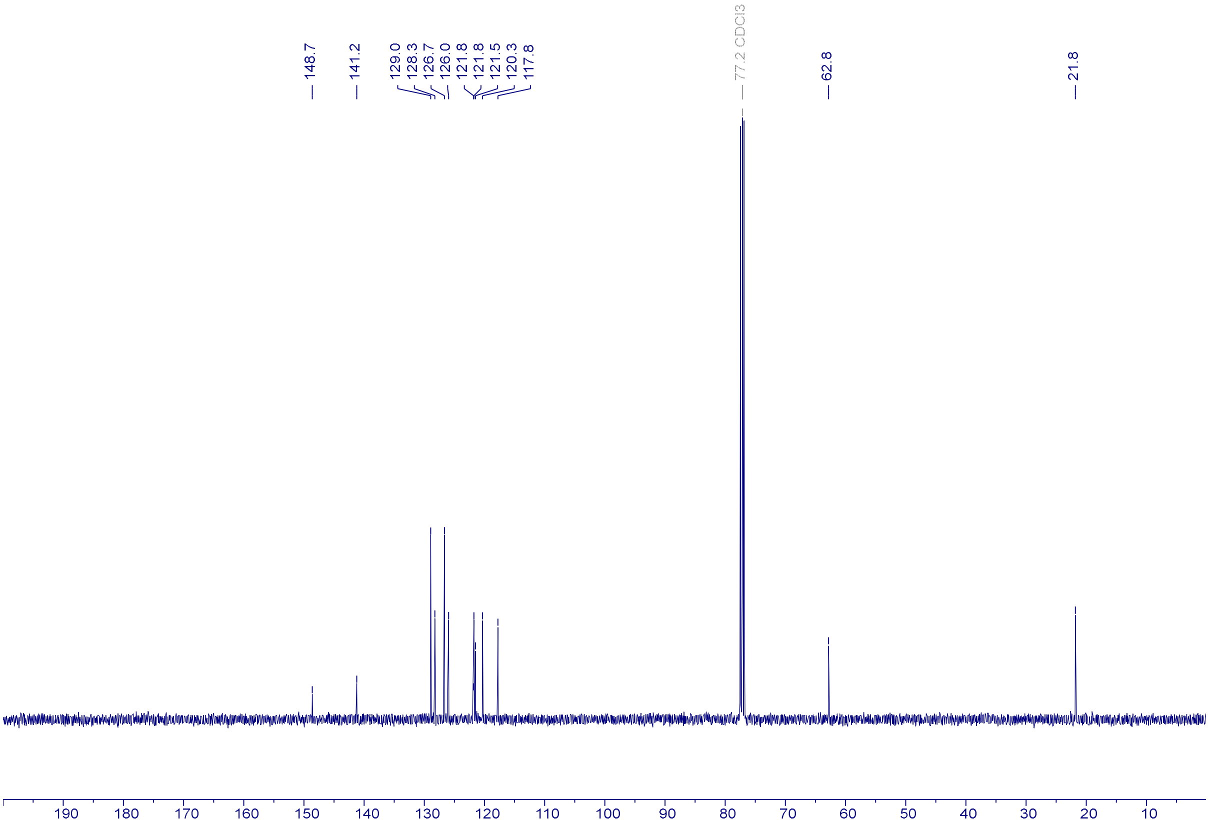

62 – ¹H NMR (400 MHz, $CDCl_3$)

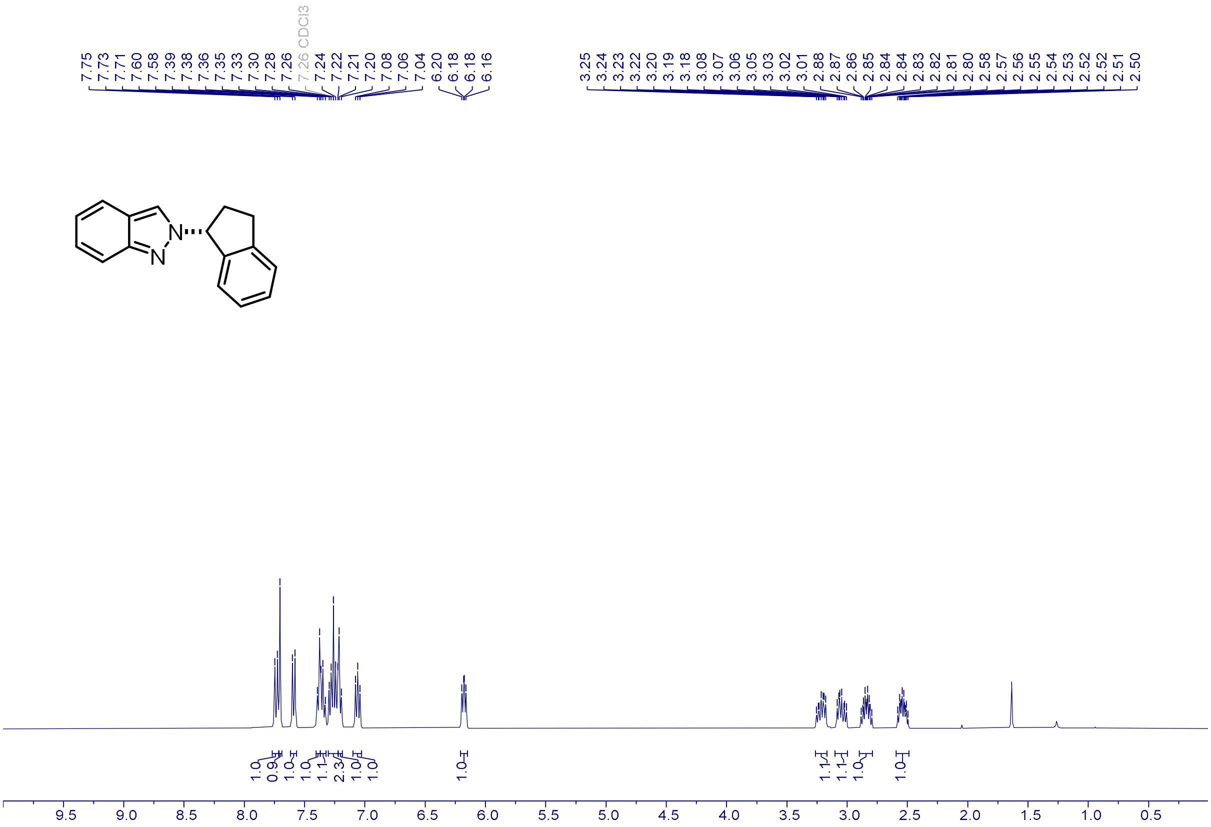

62 – ¹³C NMR (101 MHz, $CDCl_3$)

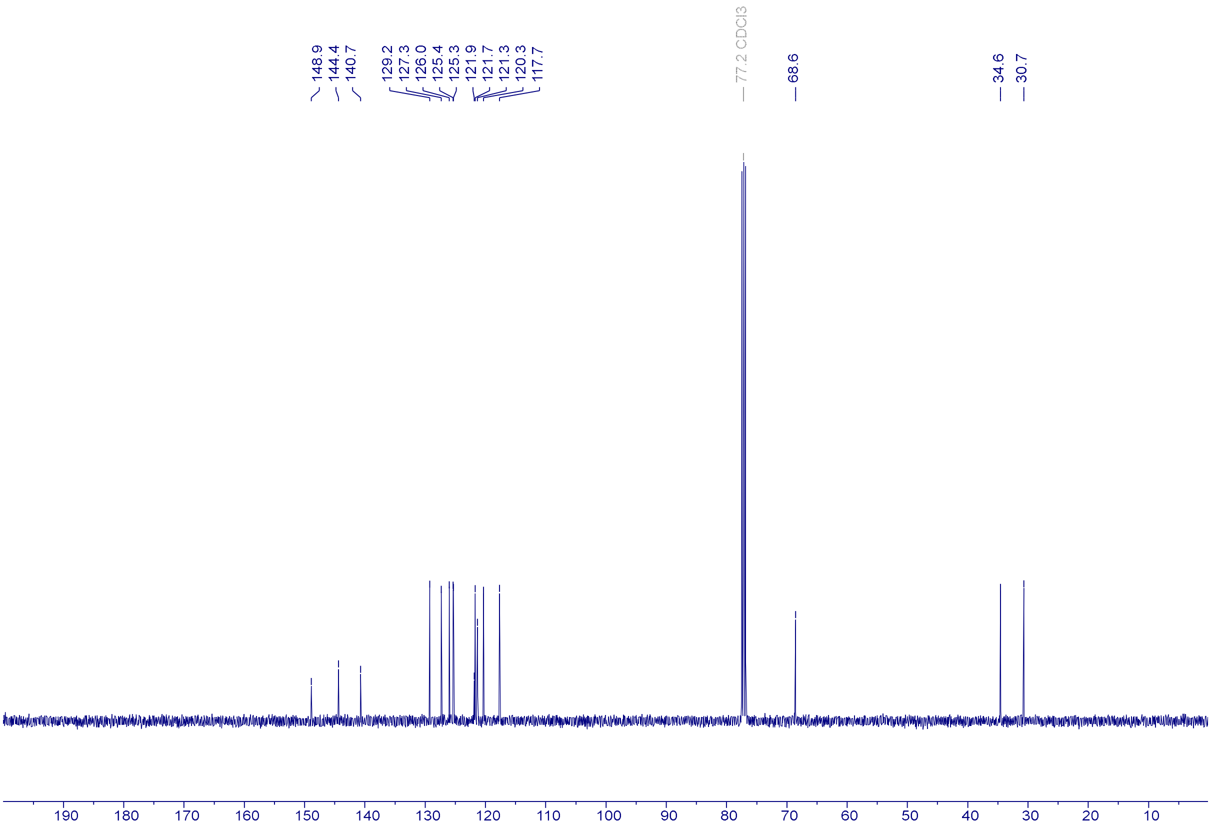

63 – ^{1}H NMR (400 MHz, $CDCl_3$)

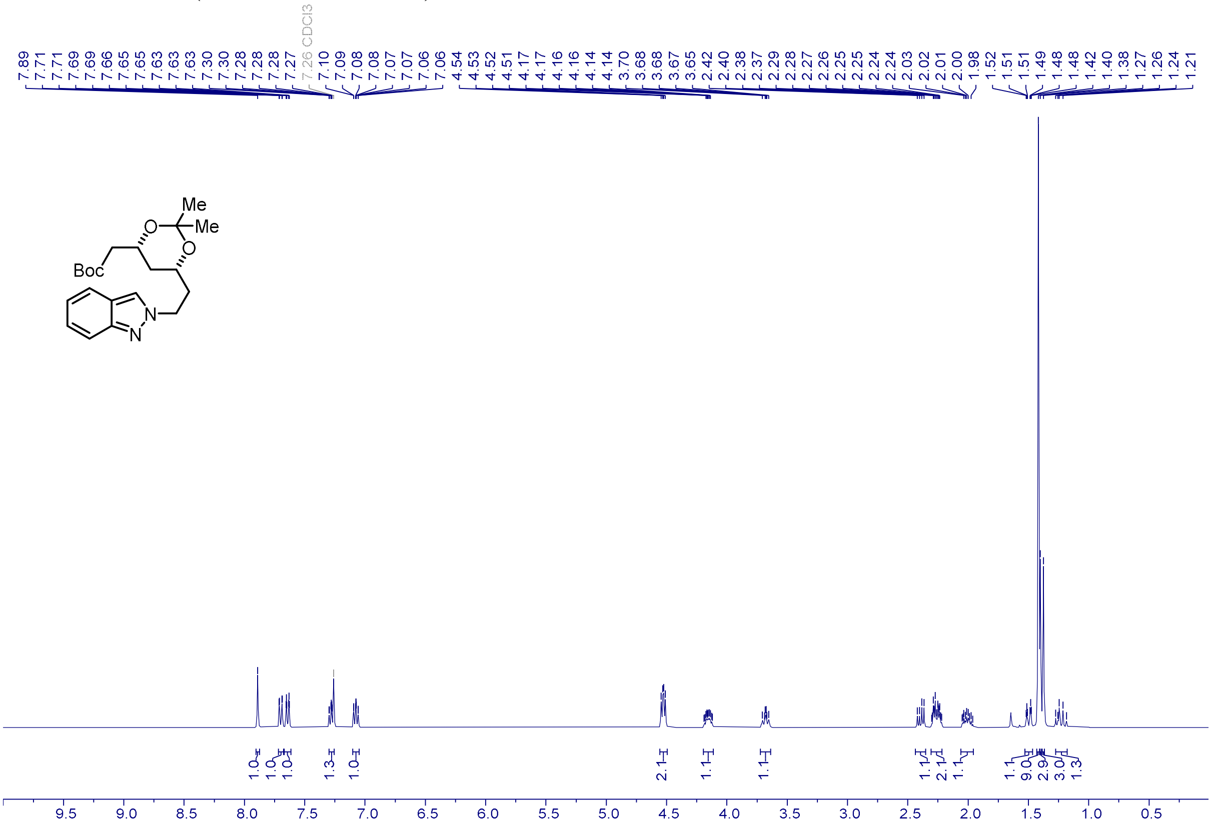

63 – ^{13}C NMR (101 MHz, $CDCl_3$)

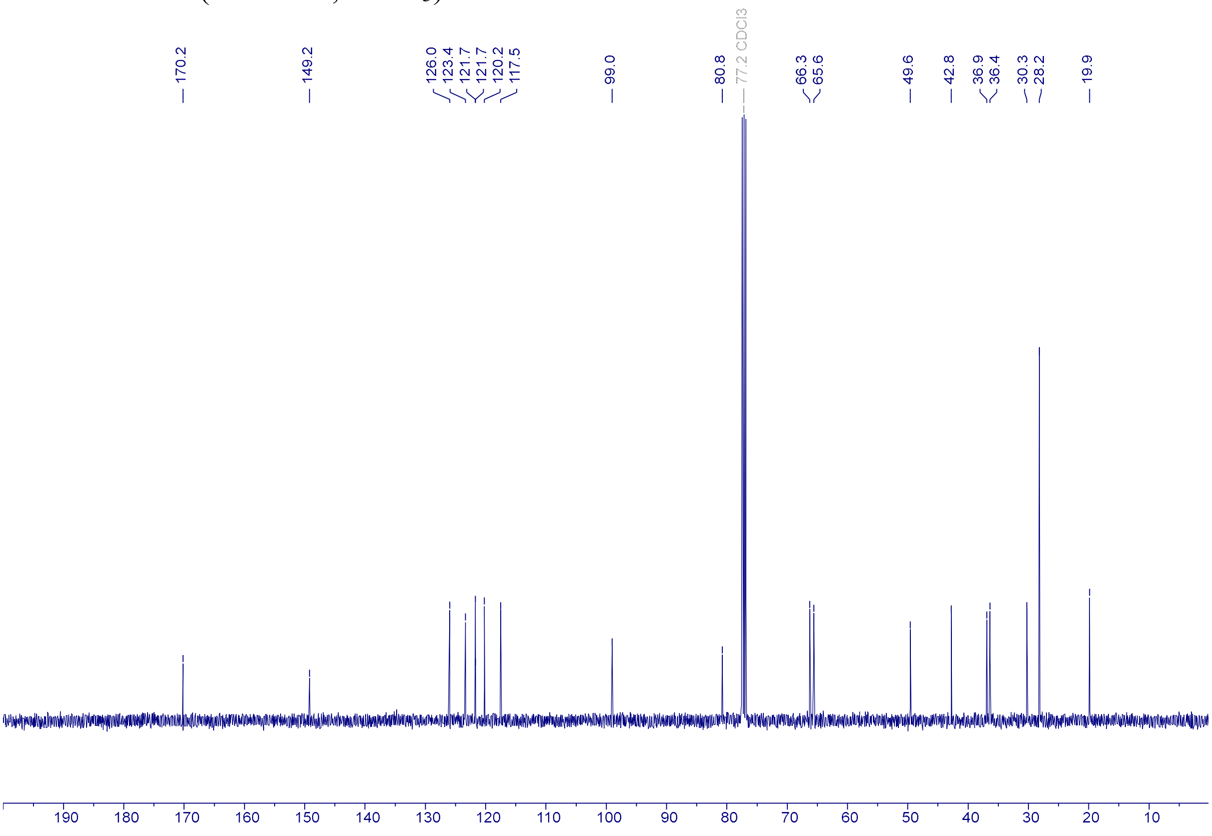

3a – ^{1}H NMR (600 MHz, $CDCl_3$)

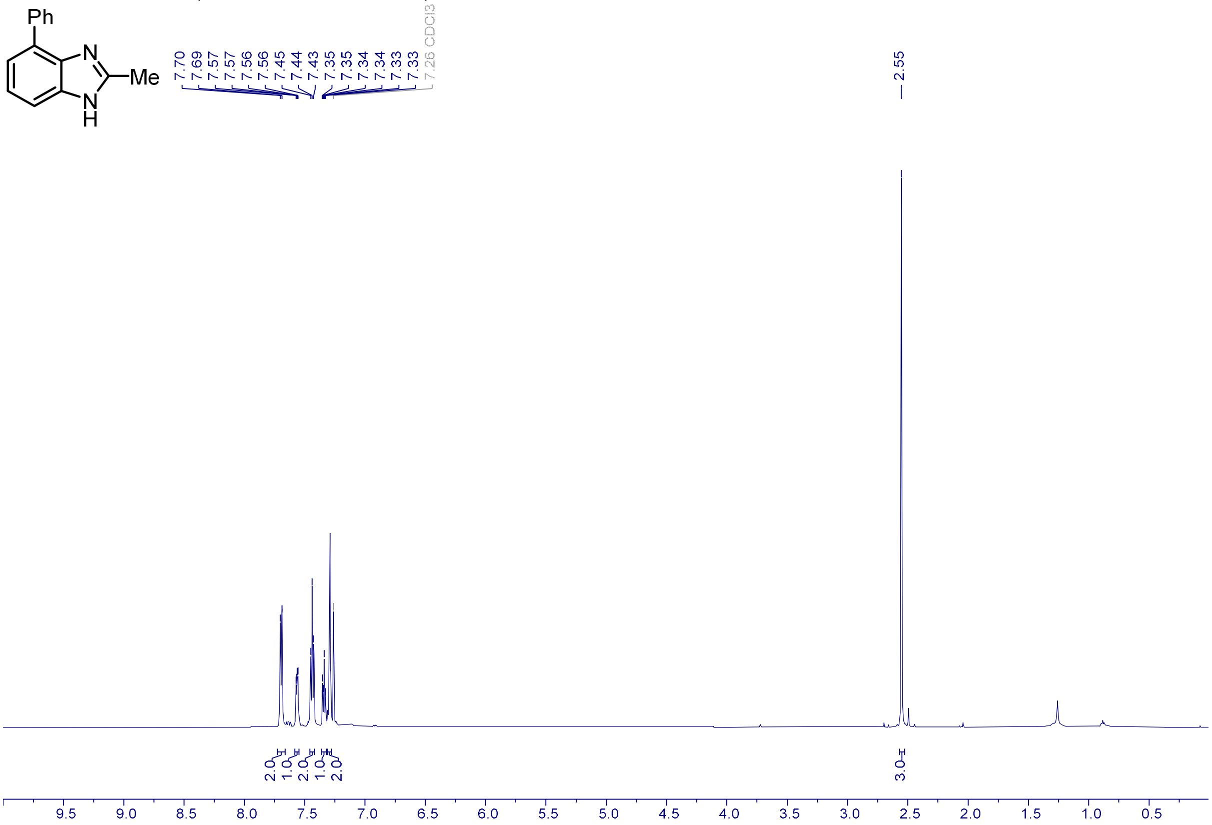

3a – ^{13}C NMR (151 MHz, $CDCl_3$)

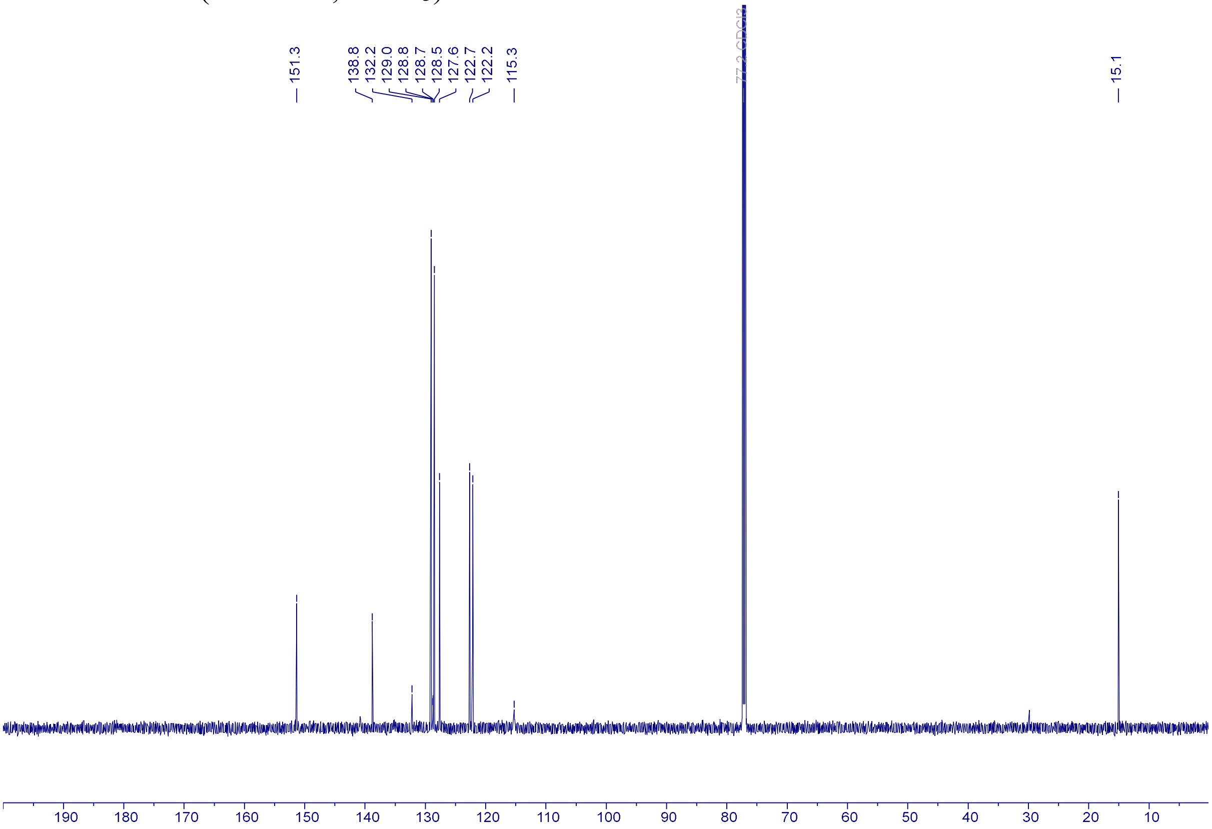

13a – ^{1}H NMR (600 MHz, $CDCl_3$)

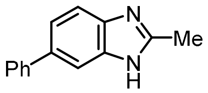

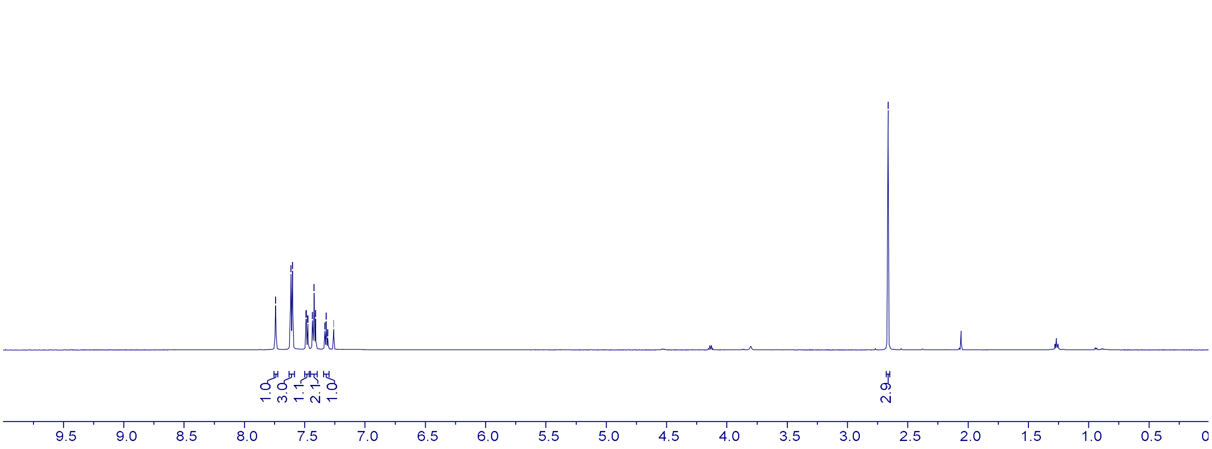

13a – ^{13}C NMR (151 MHz, $CDCl_3$)

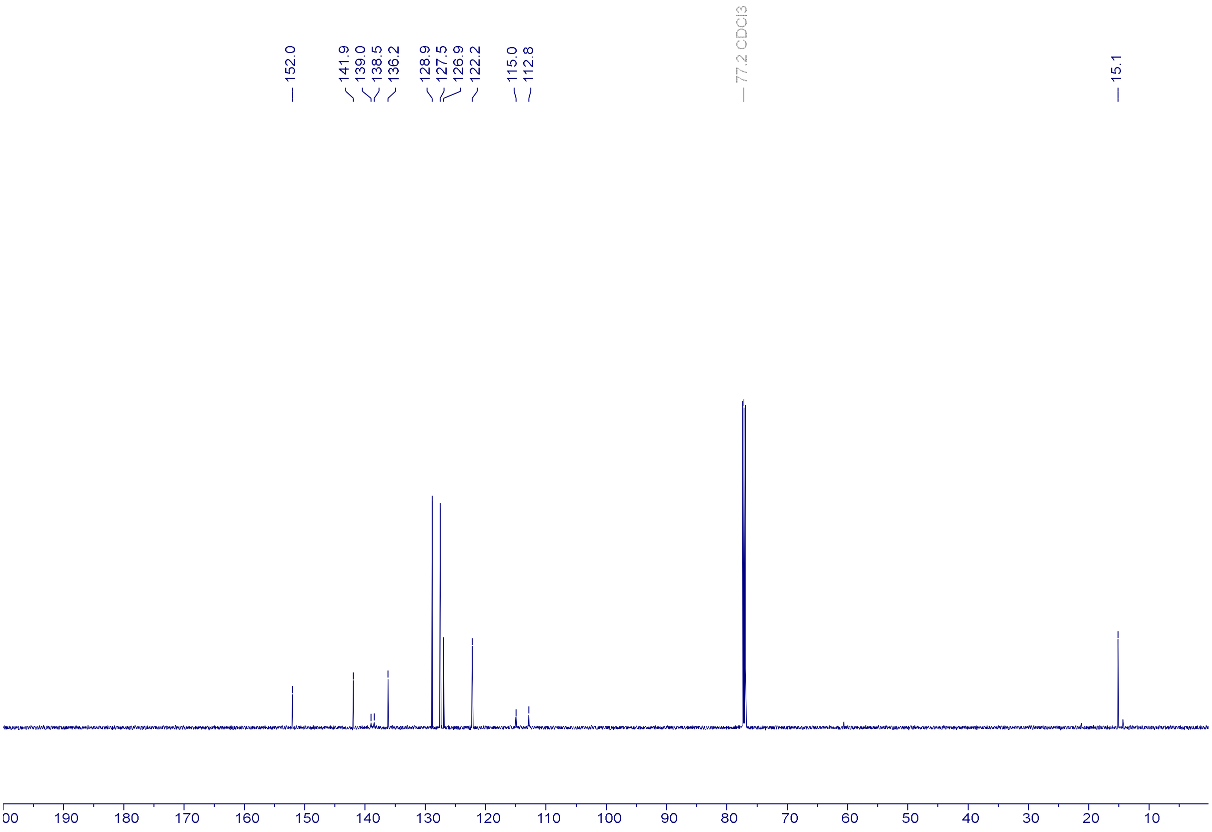

30a – ^{1}H NMR (400 MHz, $CDCl_3$)

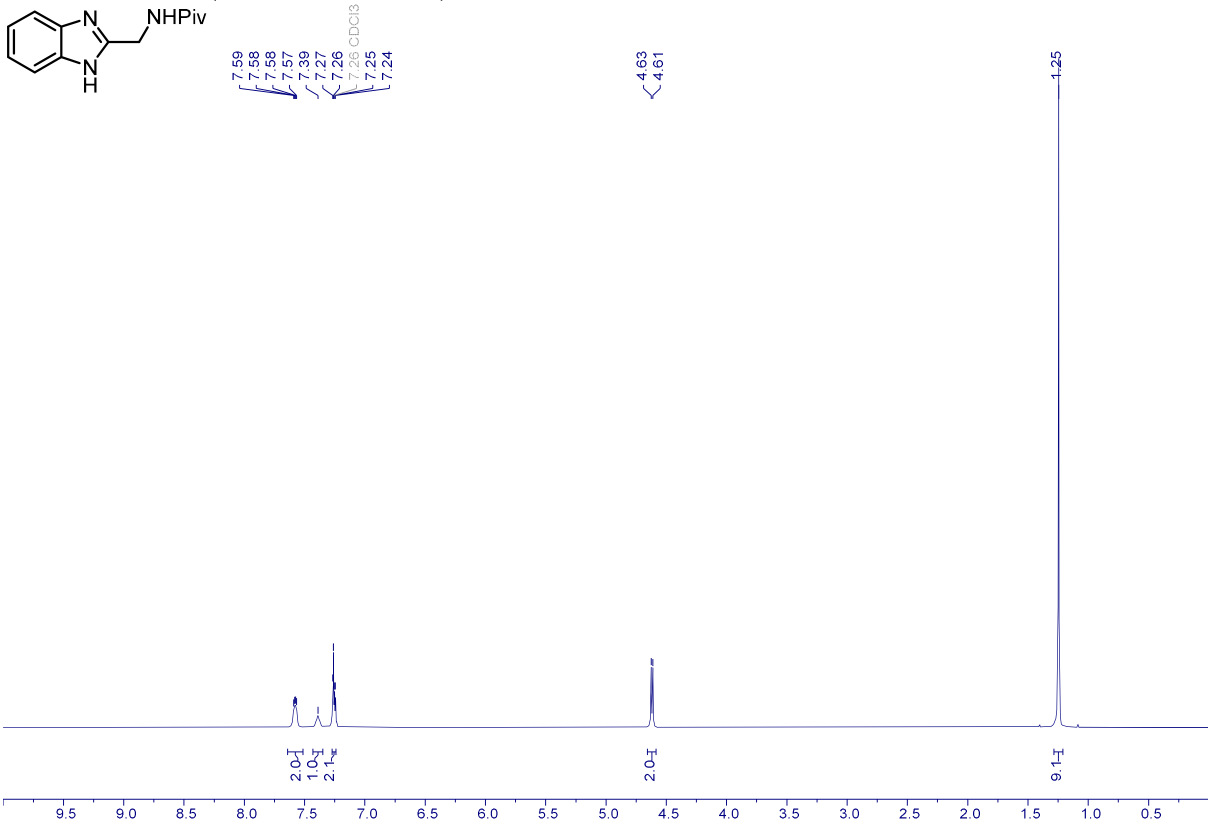

30a – ^{13}C NMR (101 MHz, $CDCl_3$)

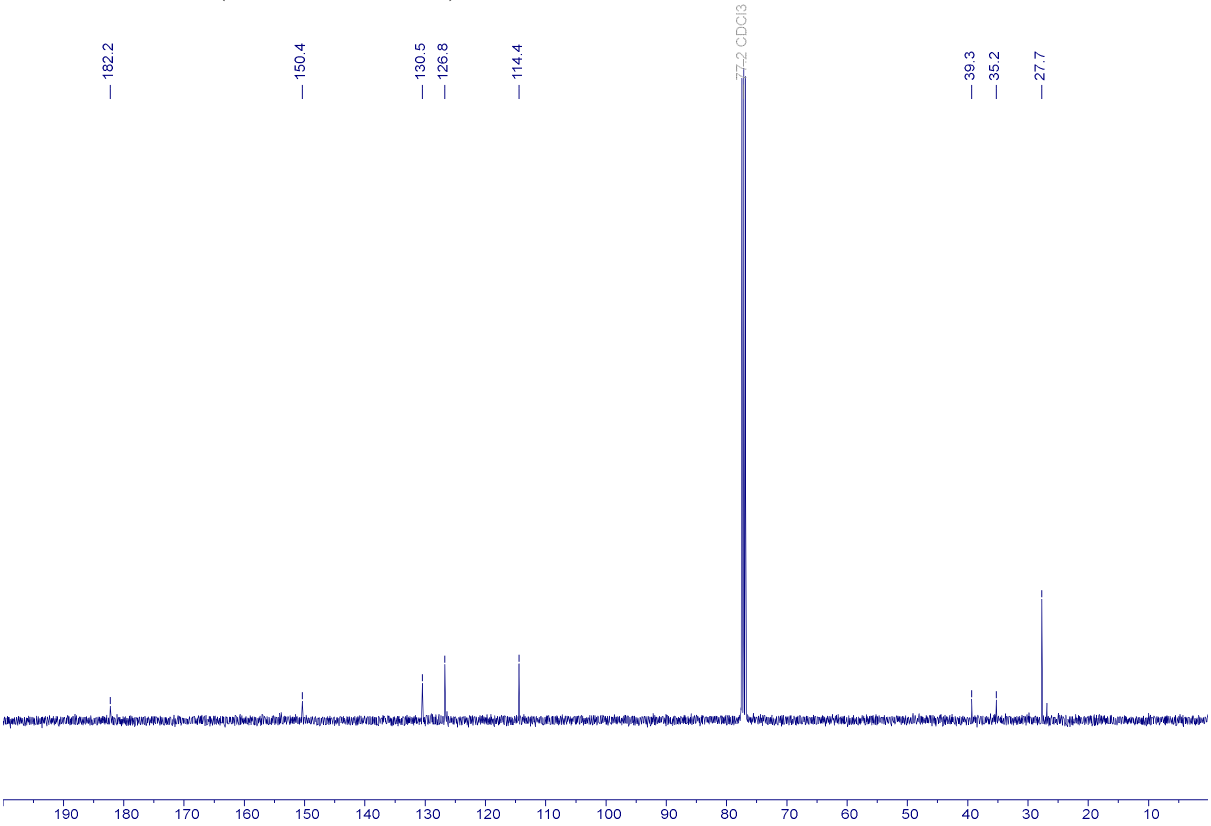

33a – ^{1}H NMR (400 MHz, DMSO)

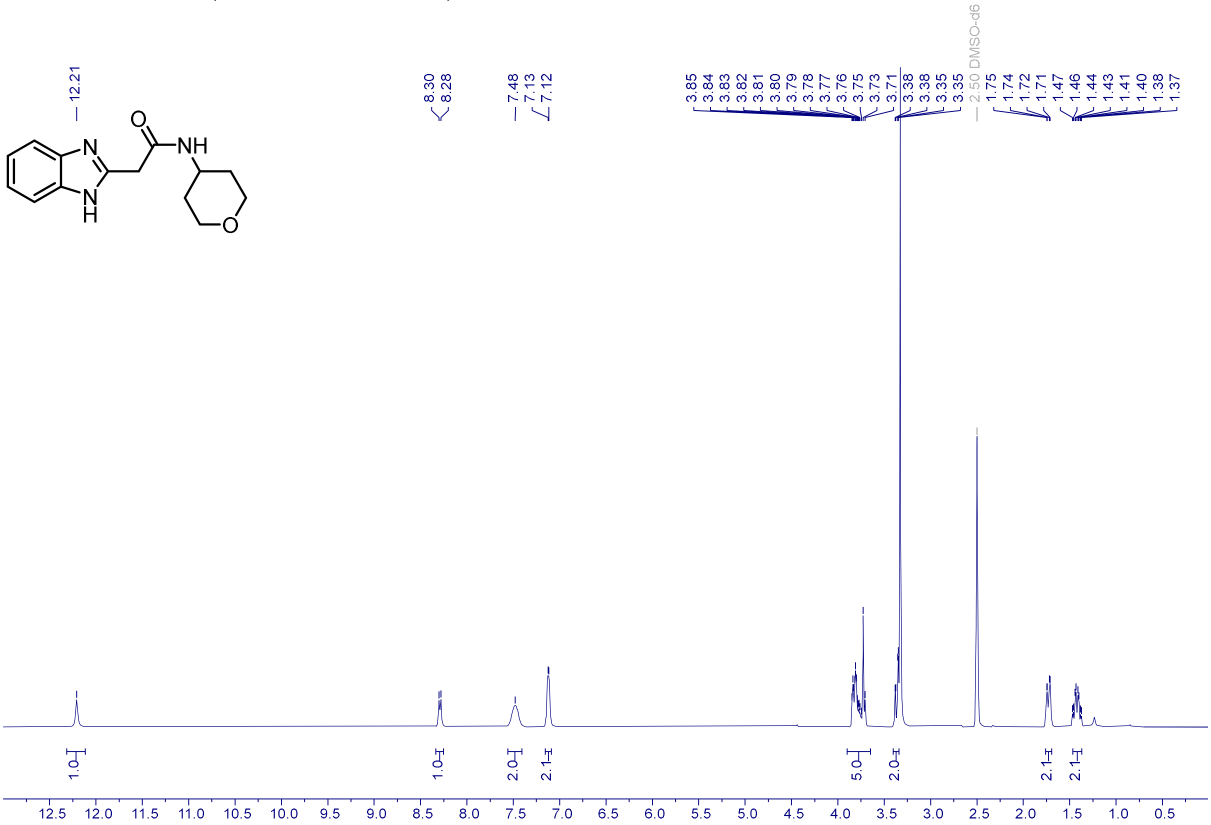

33a – ^{13}C NMR (151 MHz, DMSO)

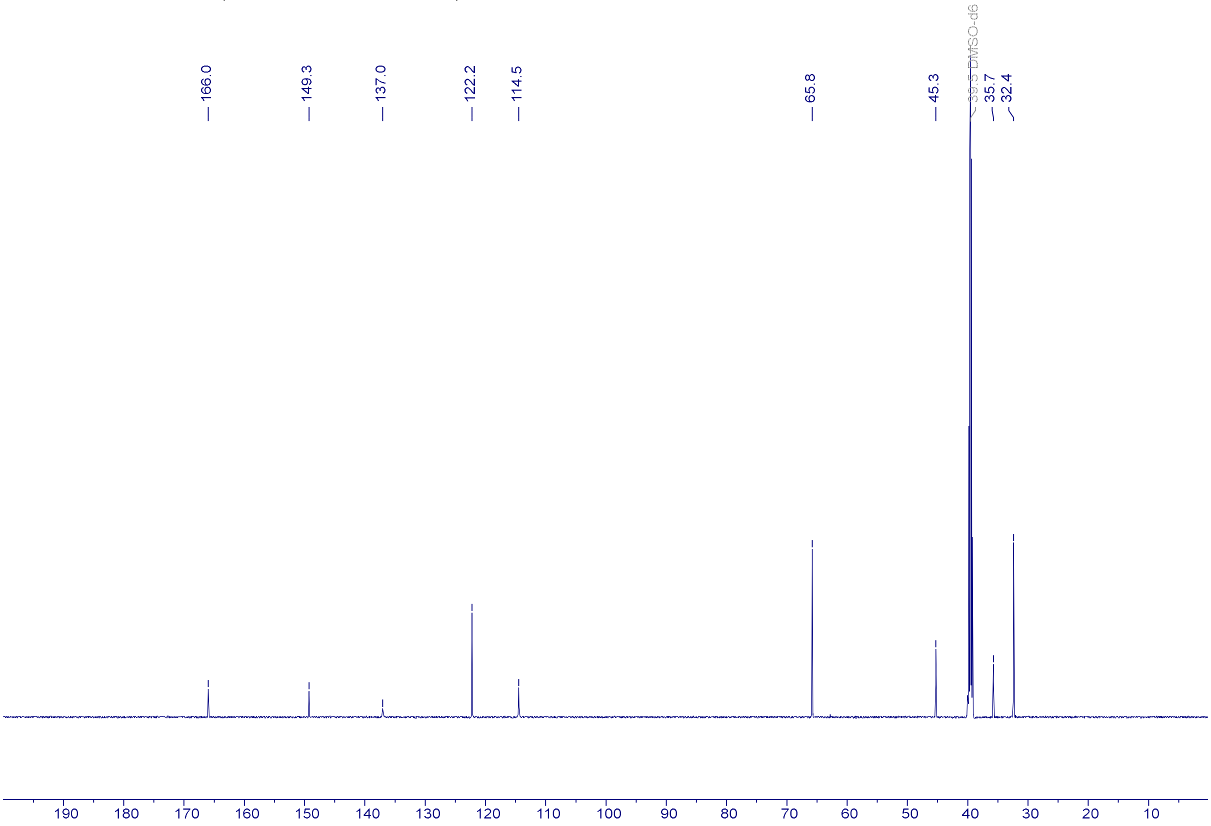

34a – ^{1}H NMR (400 MHz, CDCl$_3$)

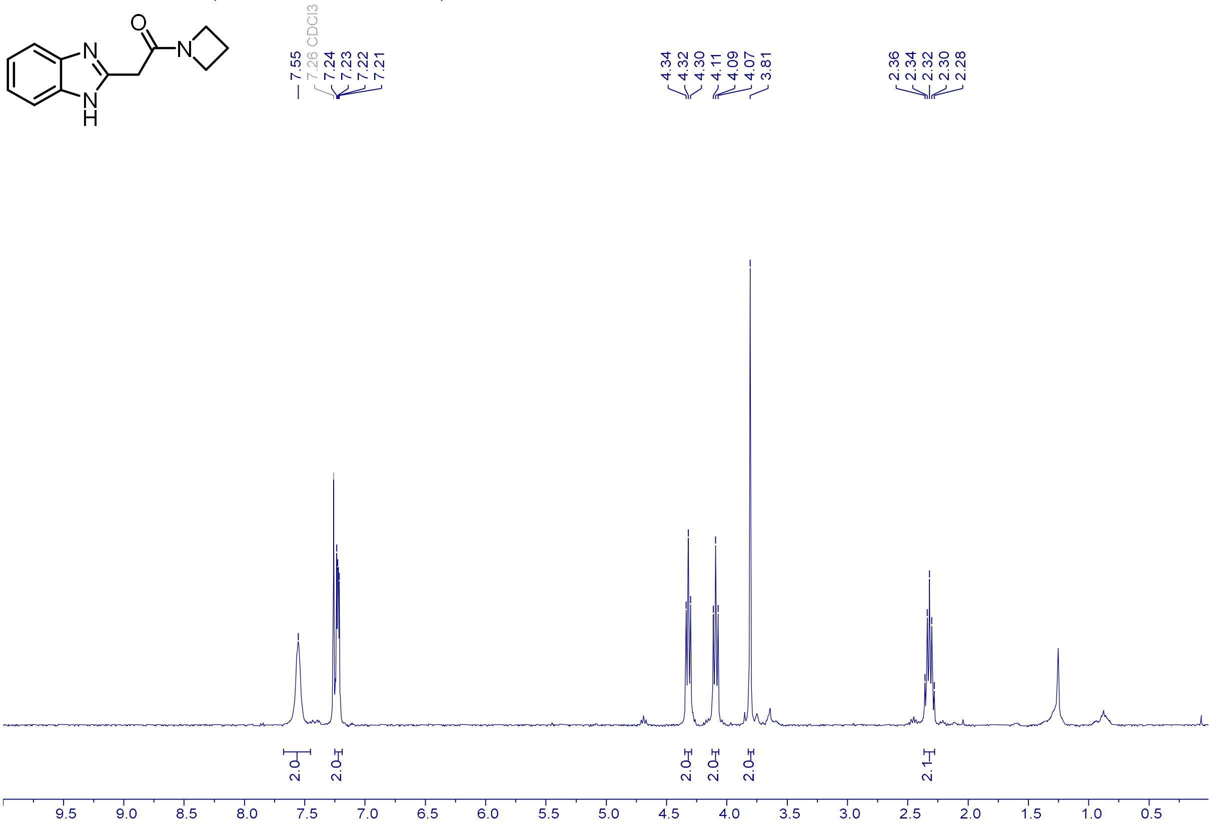

34a – ^{13}C NMR (101 MHz, CDCl$_3$)

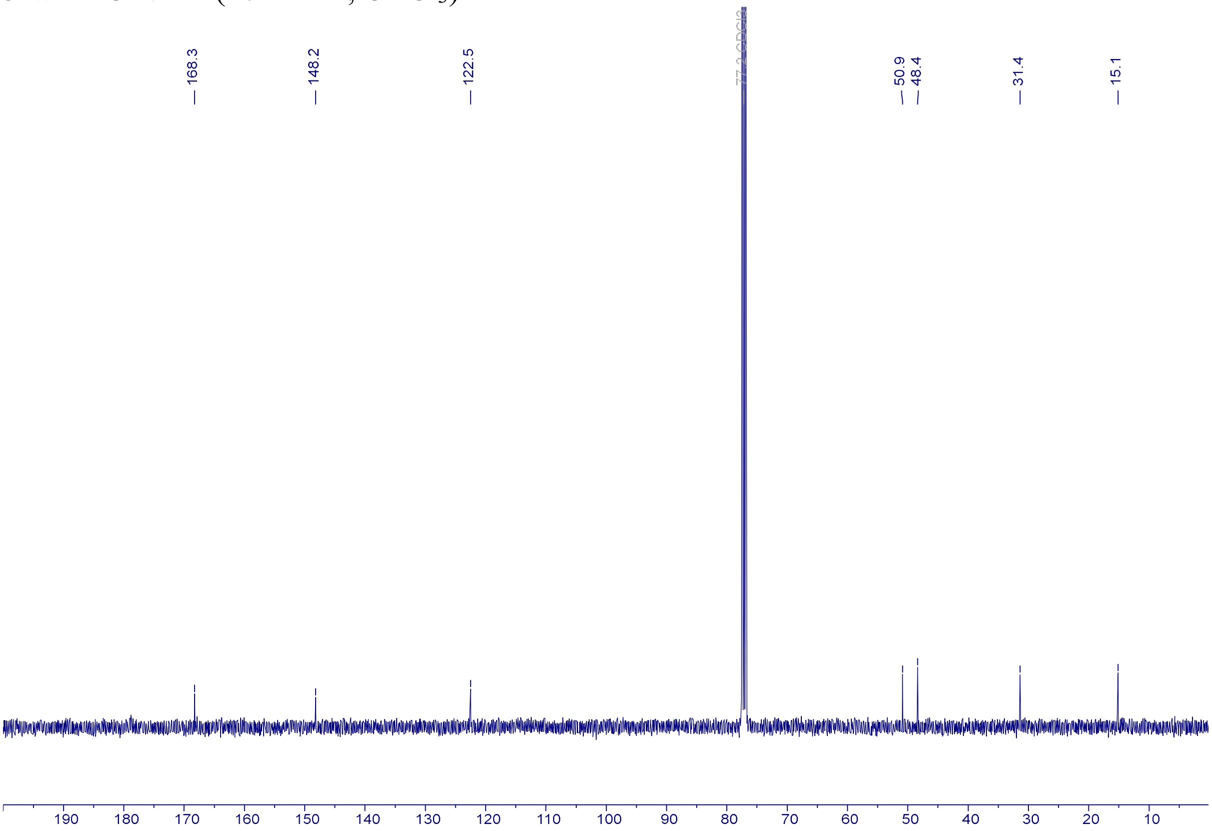

35a – ^{1}H NMR (600 MHz, $CDCl_3$)

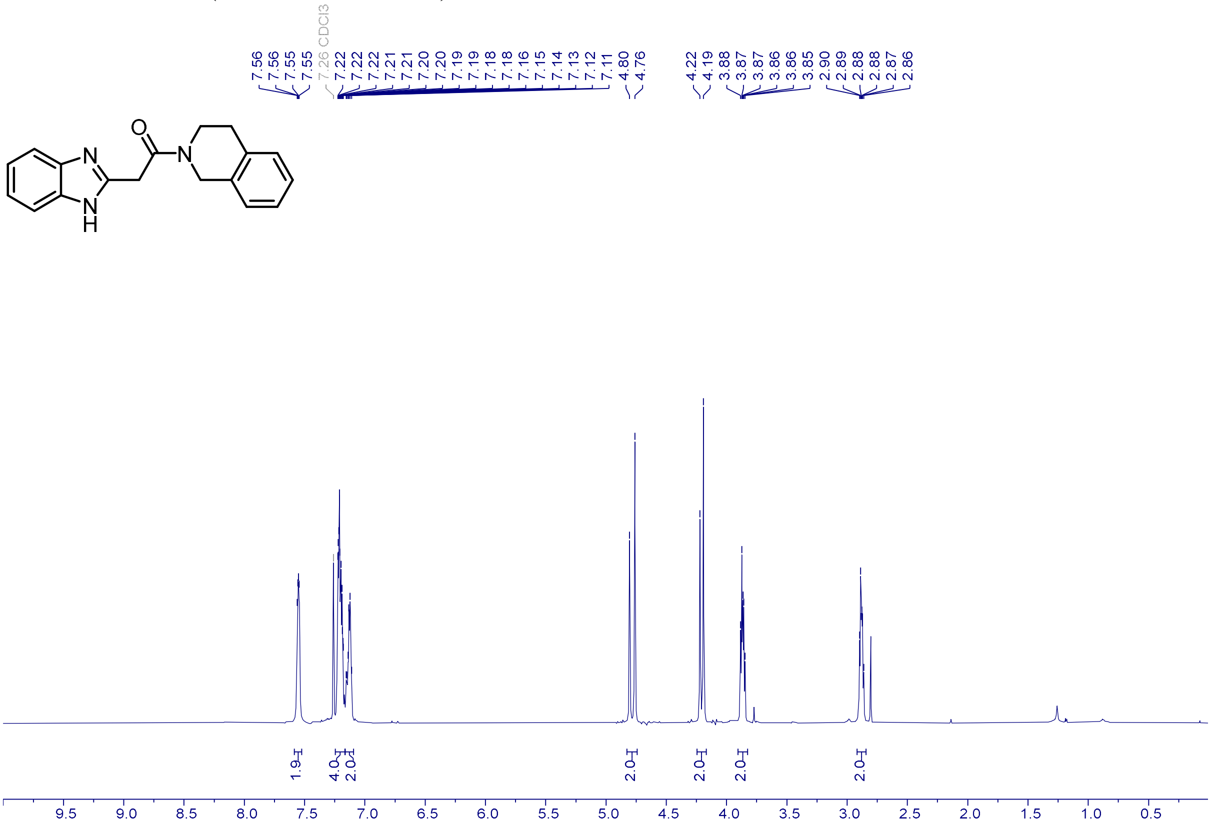

35a – ^{13}C NMR (151 MHz, $CDCl_3$)

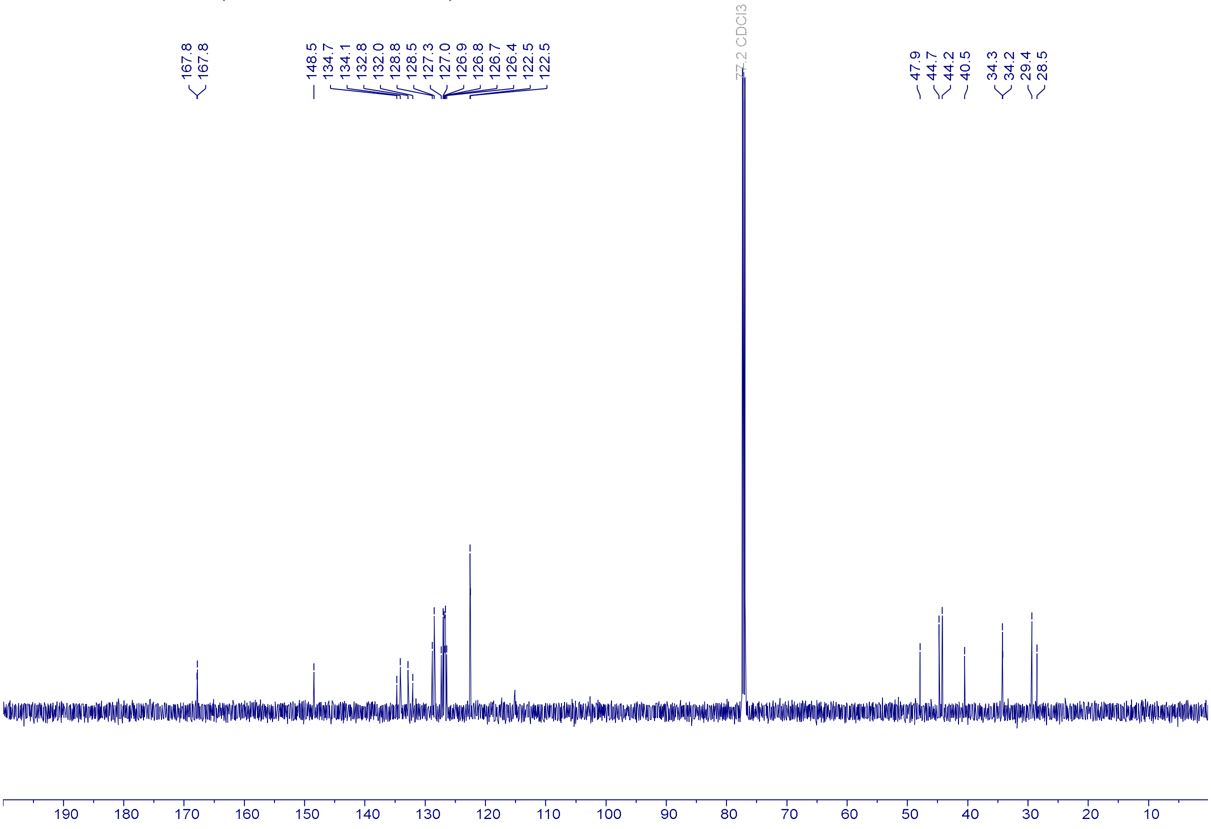

36a – ^{1}H NMR (400 MHz, $CDCl_3$)

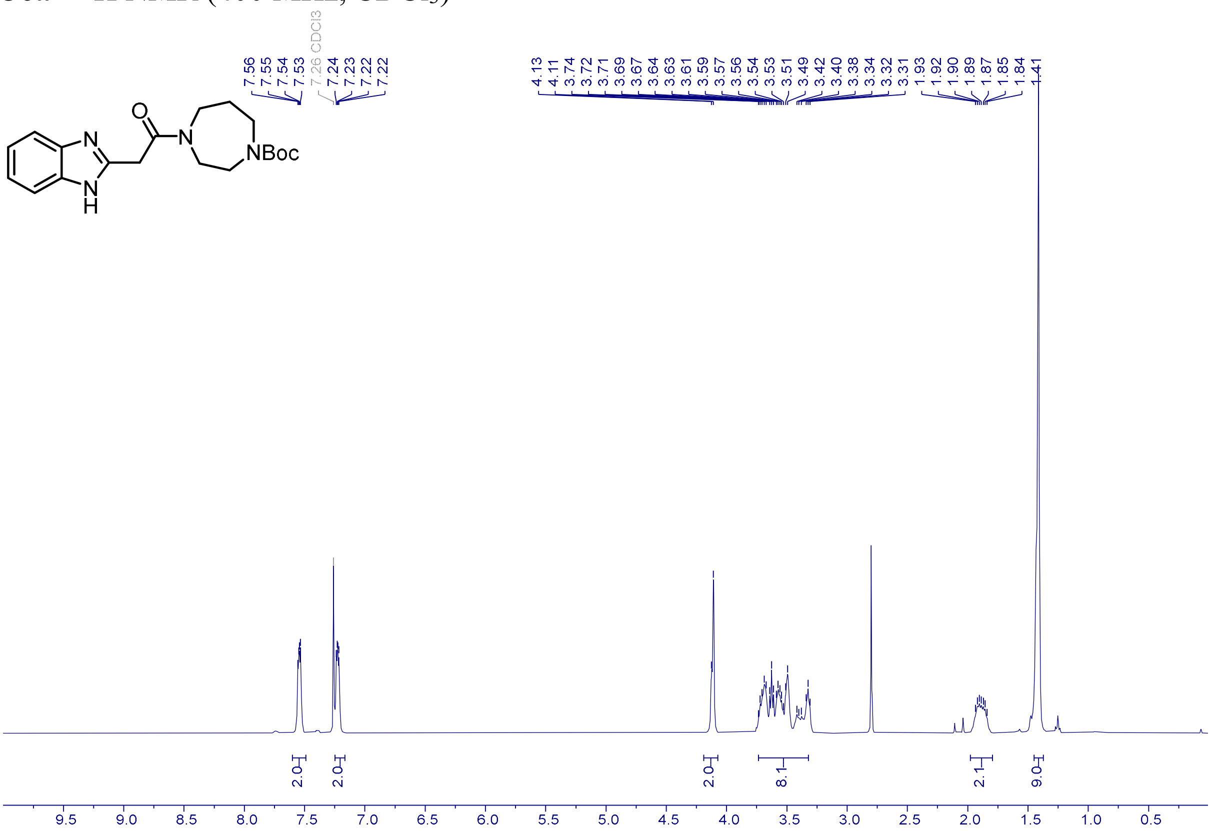

36a – ^{13}C NMR (101 MHz, $CDCl_3$)

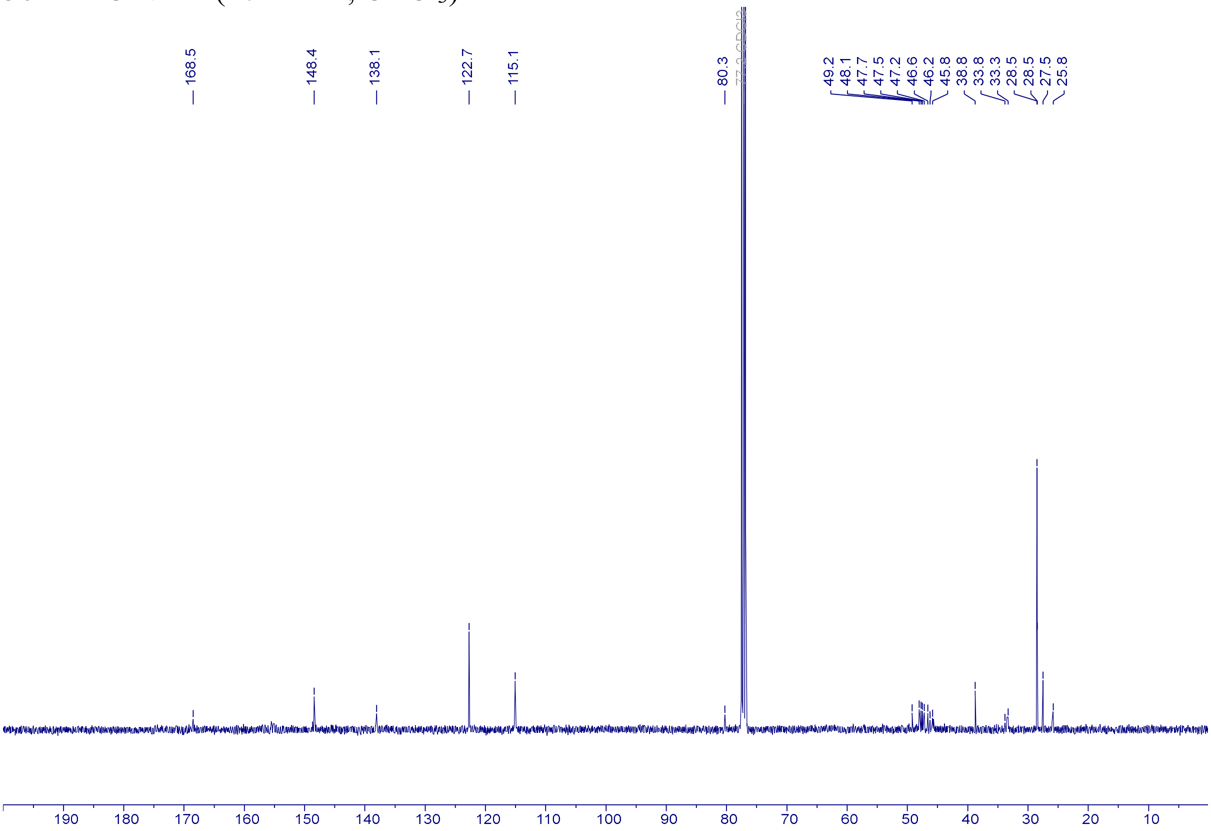

37a – ^{1}H NMR (400 MHz, $CDCl_3$)

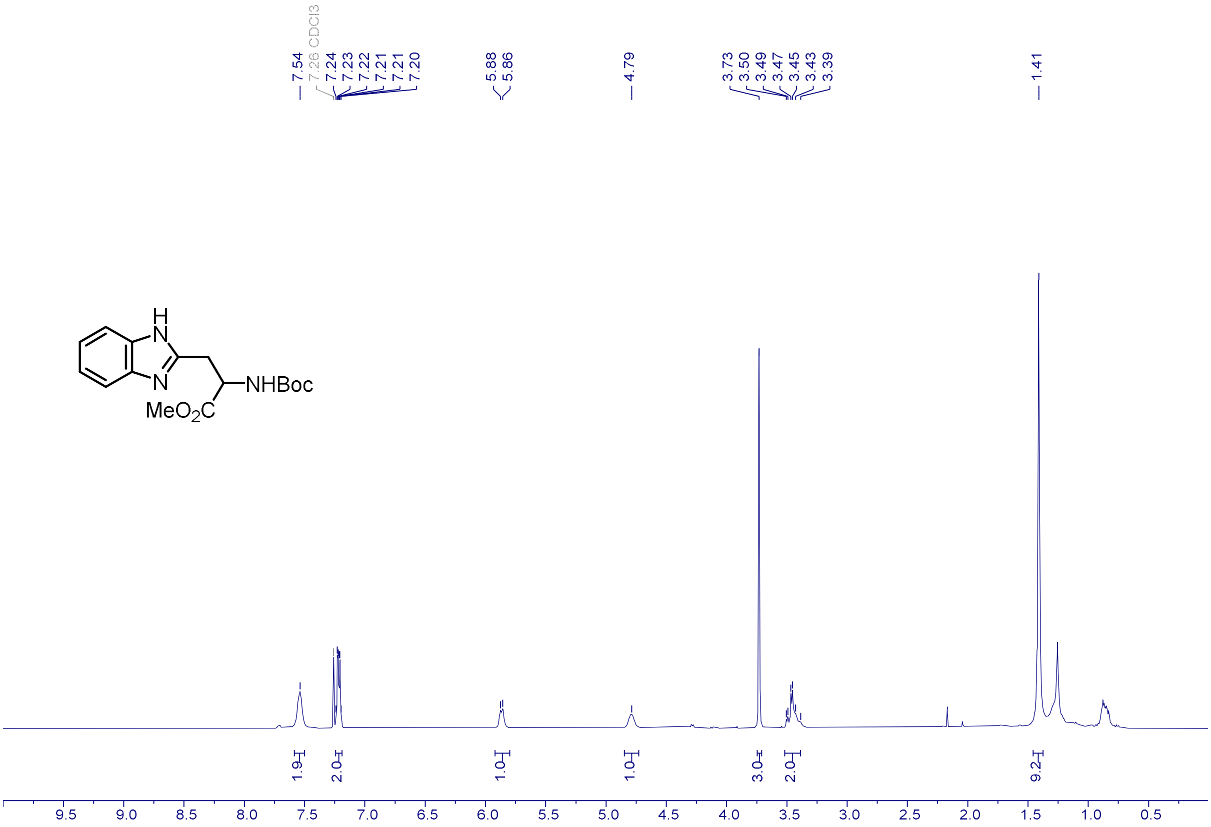

37a– ^{13}C NMR (101 MHz, $CDCl_3$)

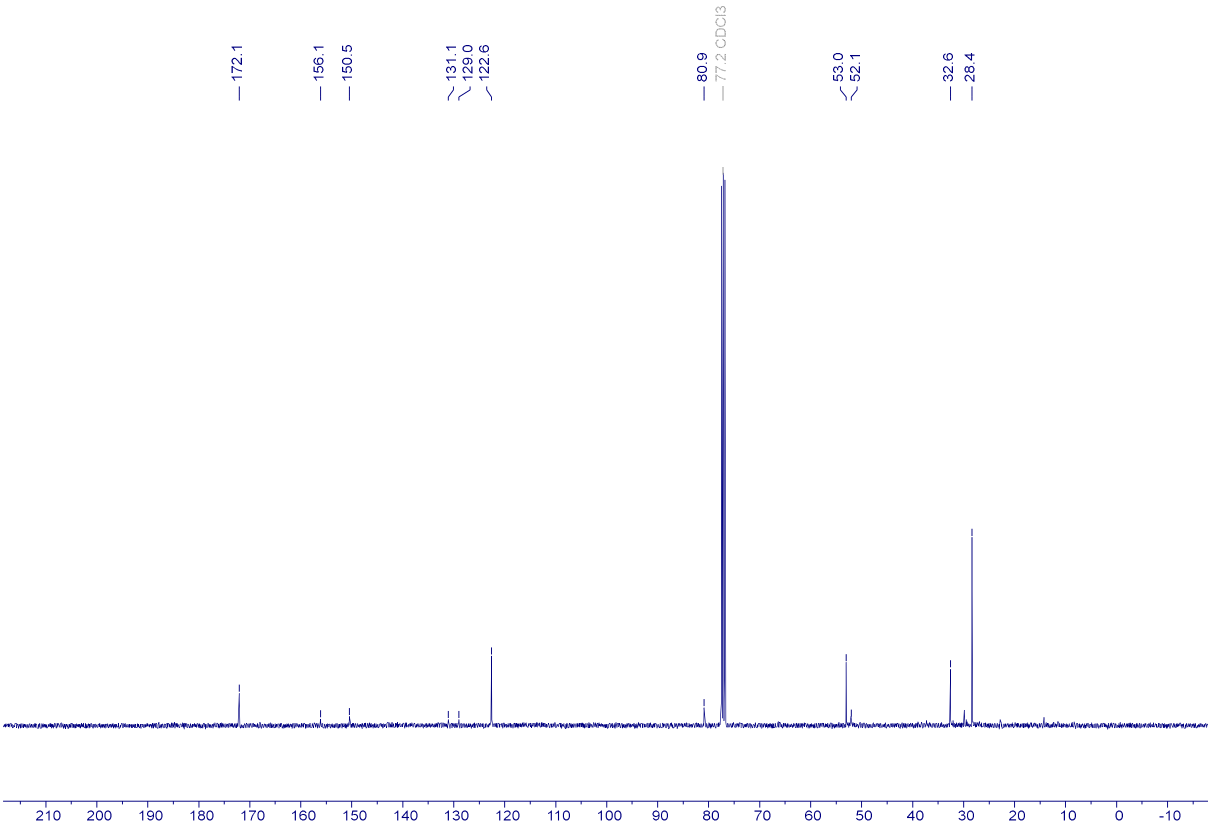

38a – ^{1}H NMR (600 MHz, $CDCl_3$)

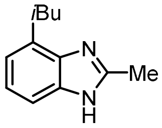

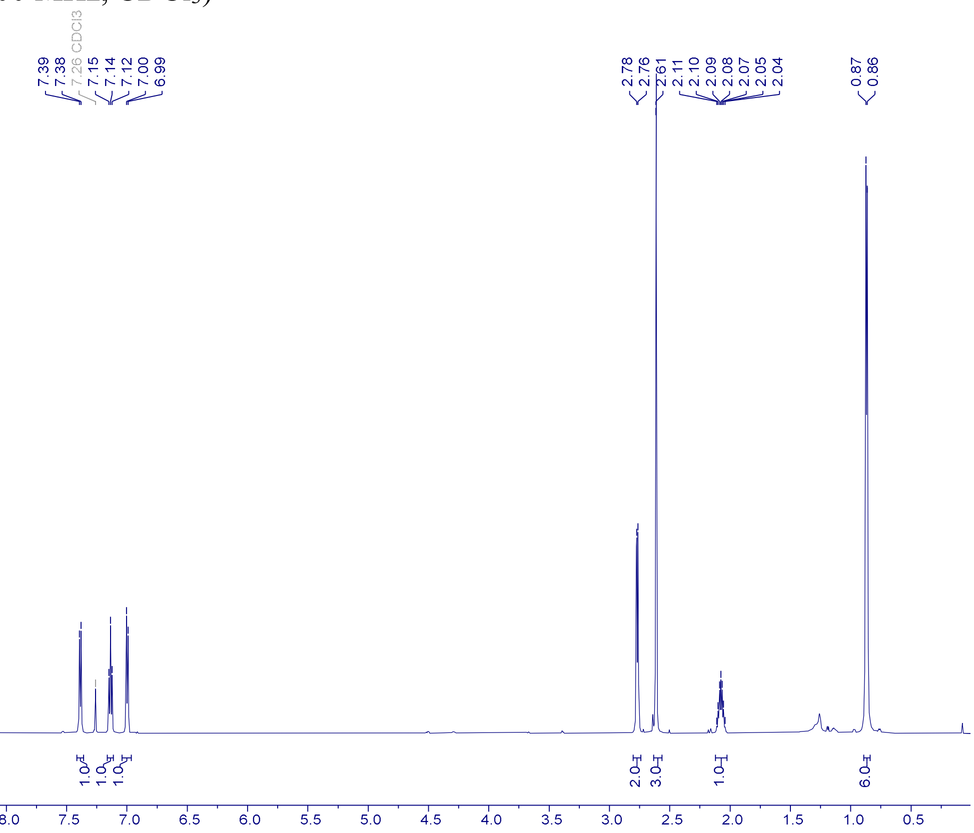

38a – ^{13}C NMR (151 MHz, $CDCl_3$)

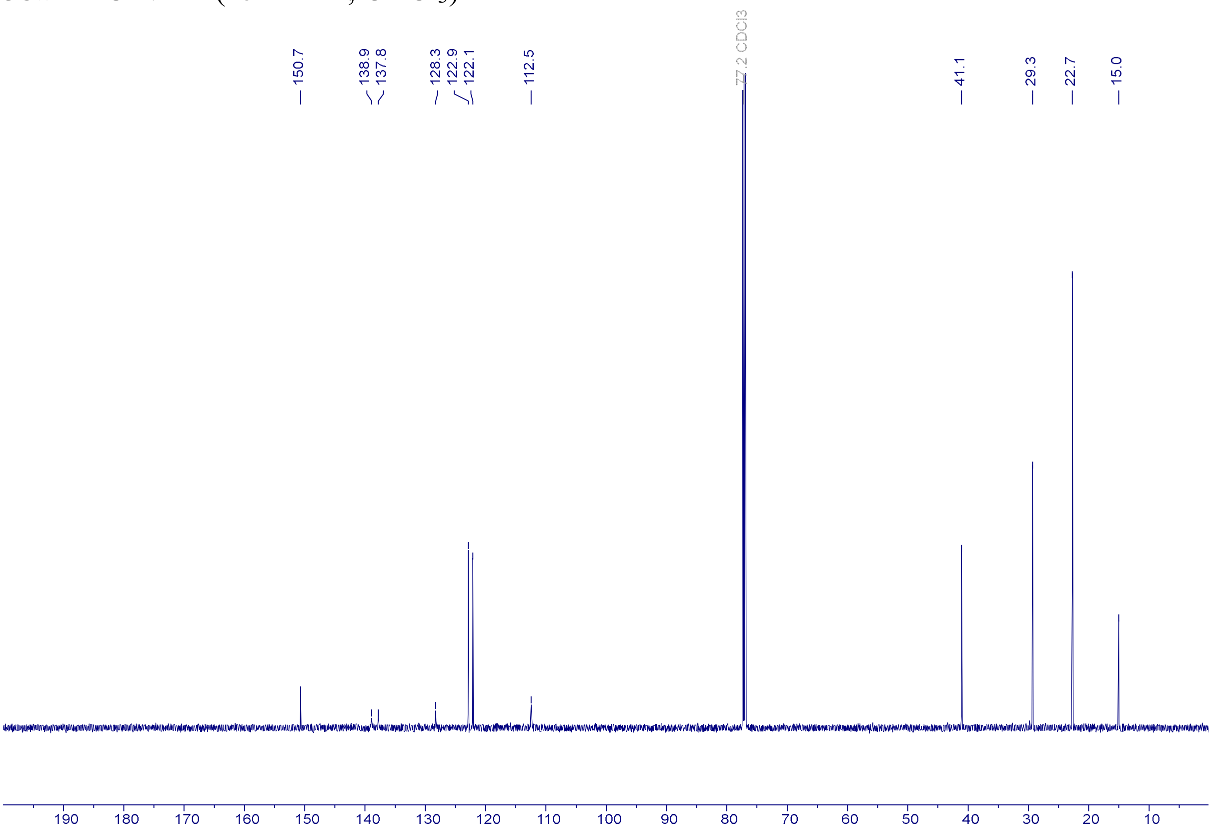

39a – ^{1}H NMR (400 MHz, $CDCl_3$)

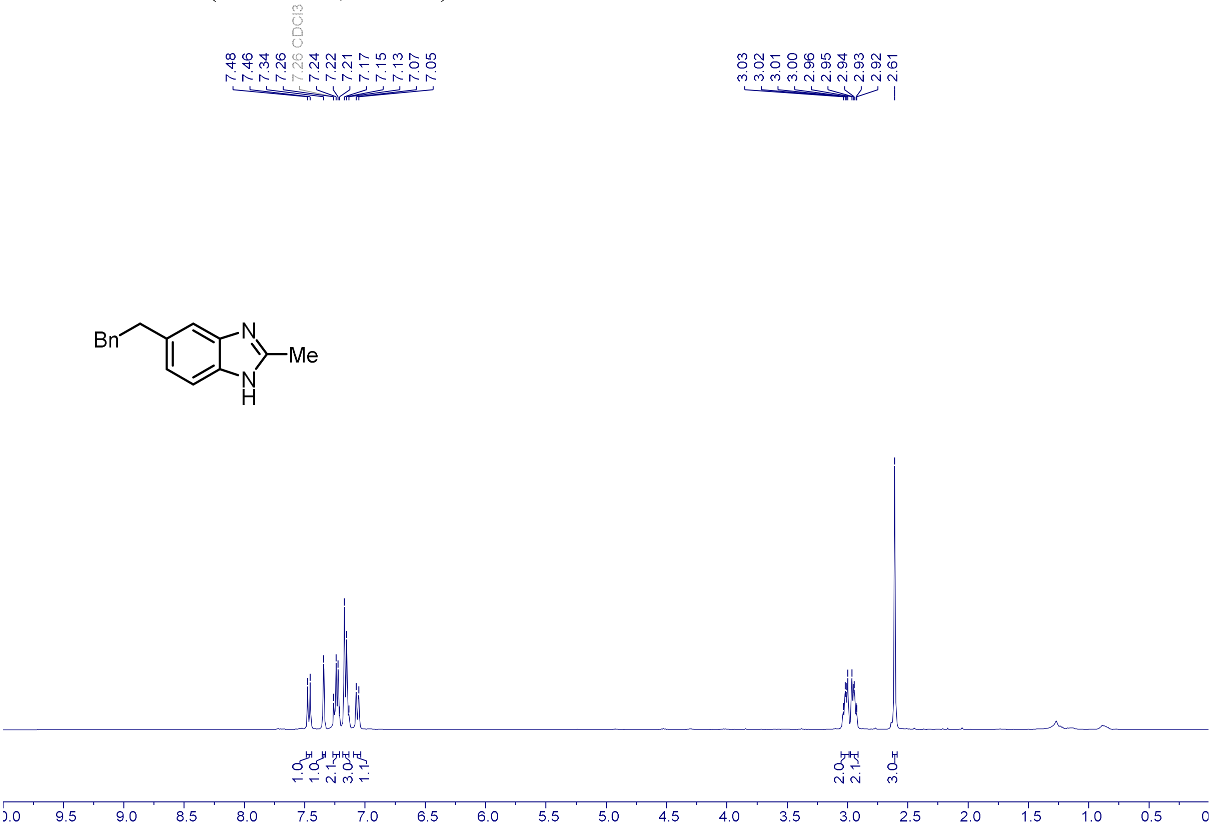

39a – ^{13}C NMR (101 MHz, $CDCl_3$)

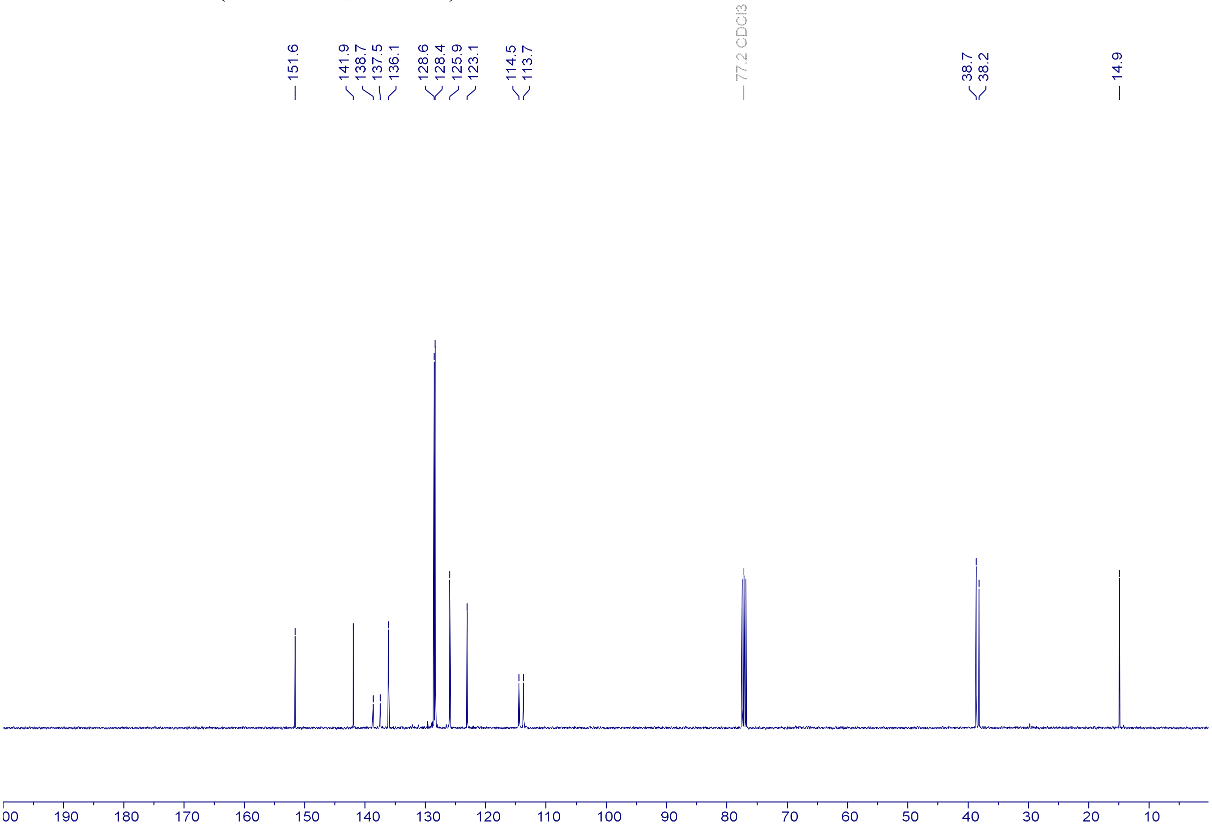

40a – ^{1}H NMR (600 MHz, $CDCl_3$)

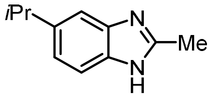

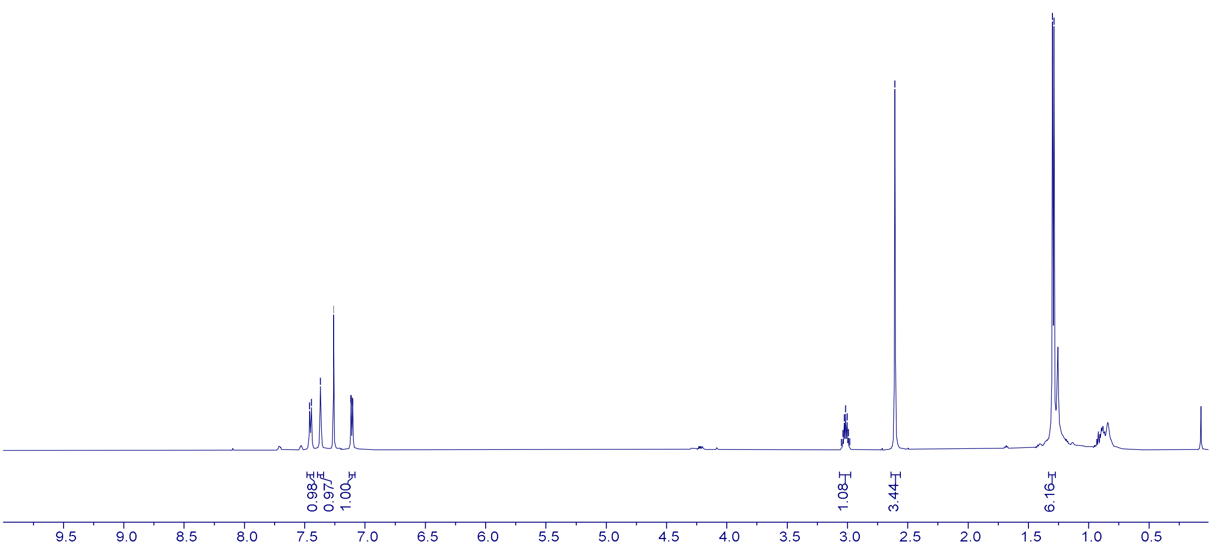

40a – ^{13}C NMR (151 MHz, $CDCl_3$)

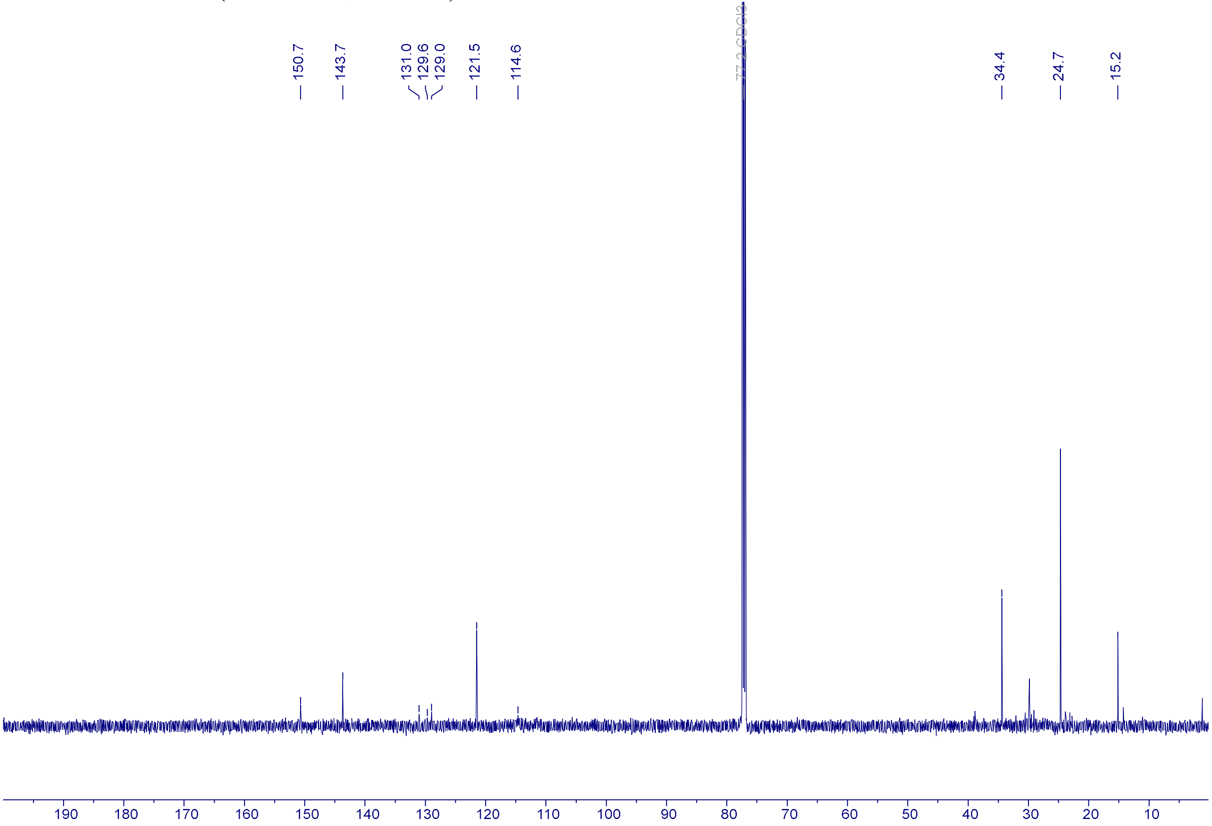

41a – ^{1}H NMR (600 MHz, $CDCl_3$)

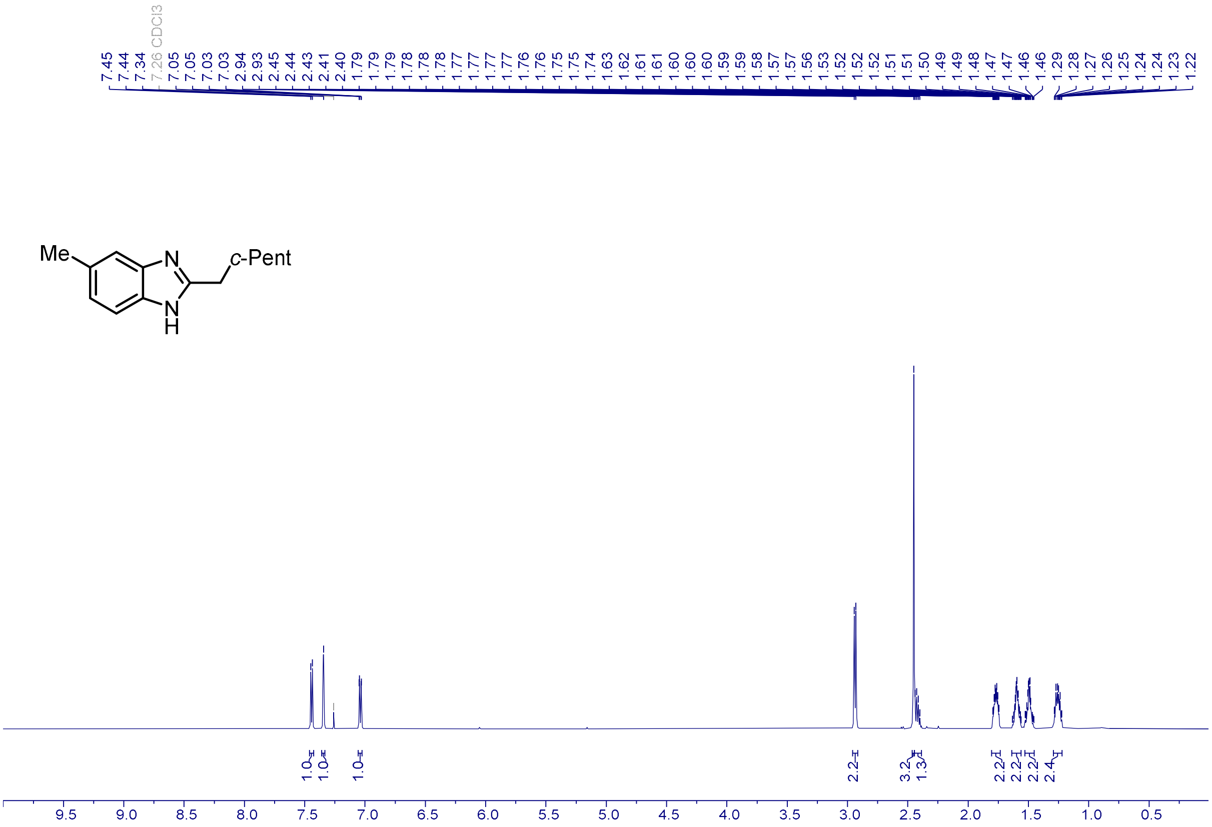

41a – ^{13}C NMR (151 MHz, $CDCl_3$)

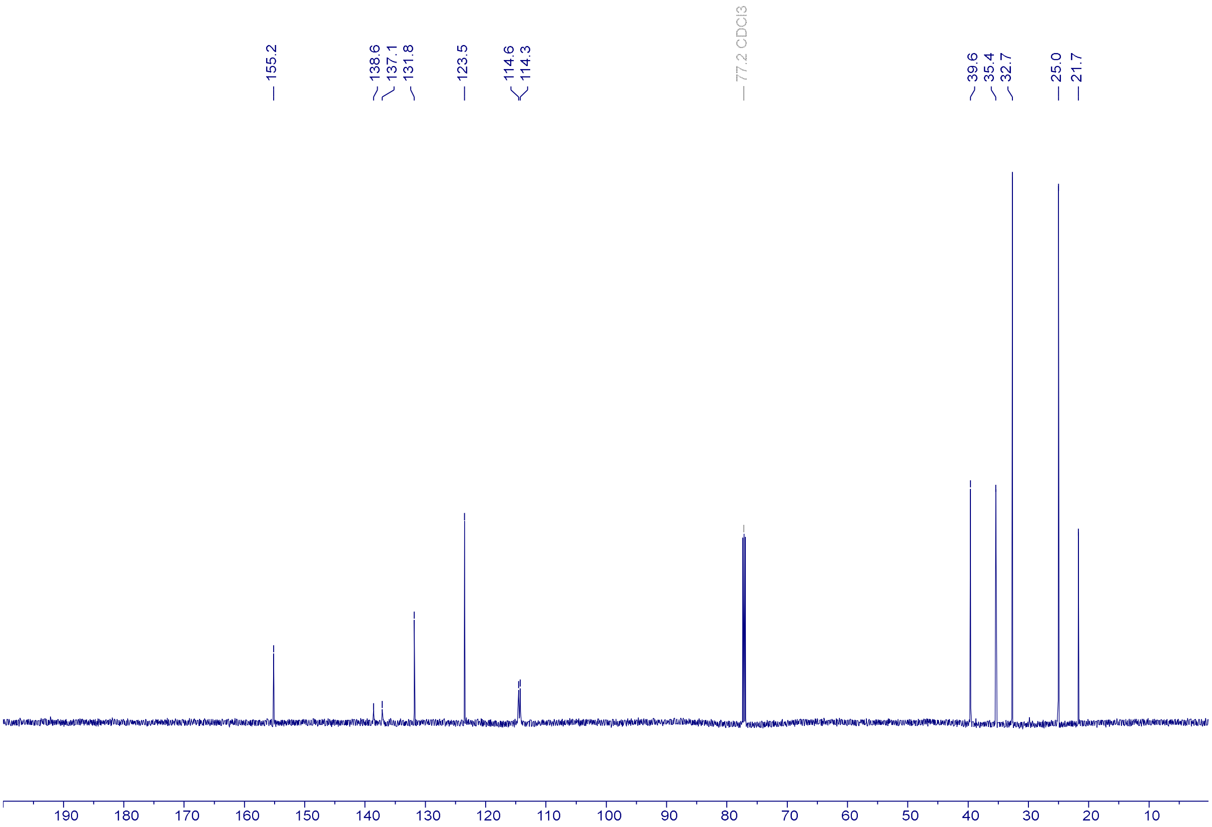

42a – ^{1}H NMR (600 MHz, $CDCl_3$)

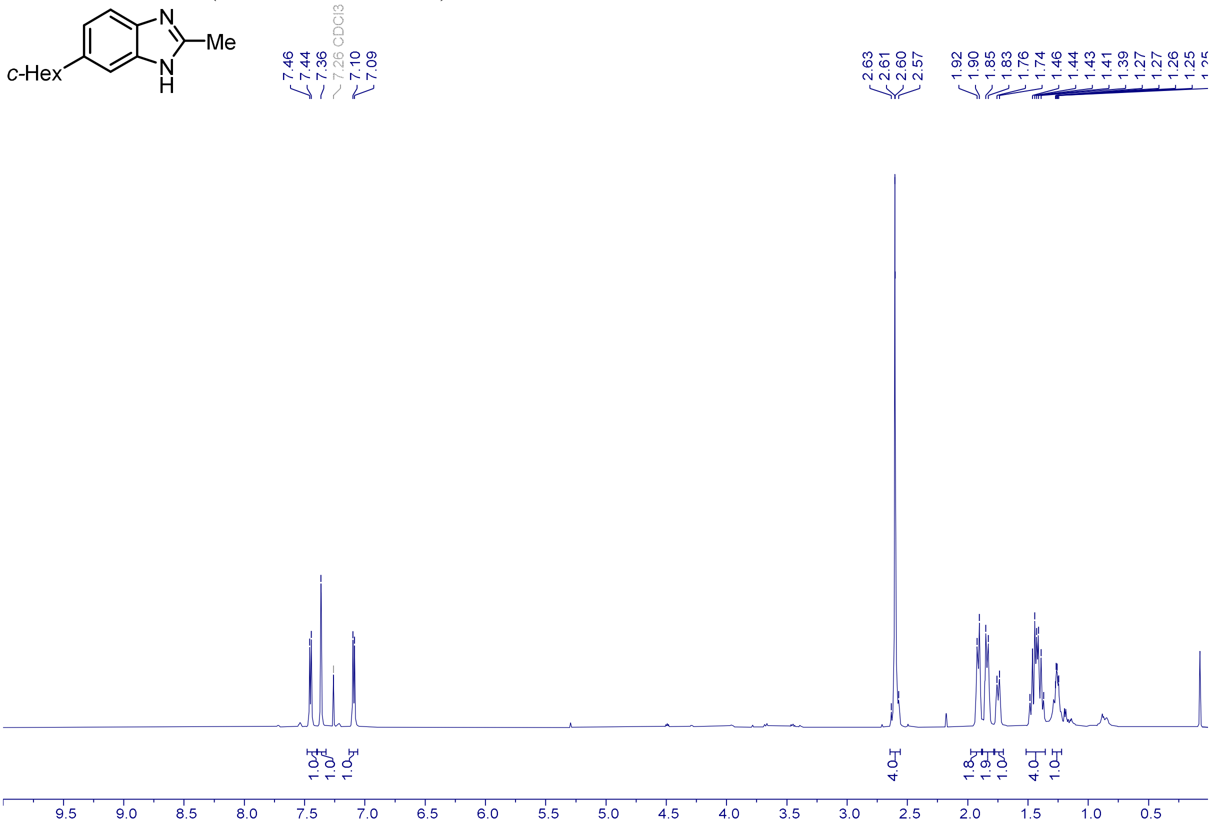

42a – ^{13}C NMR (151 MHz, $CDCl_3$)

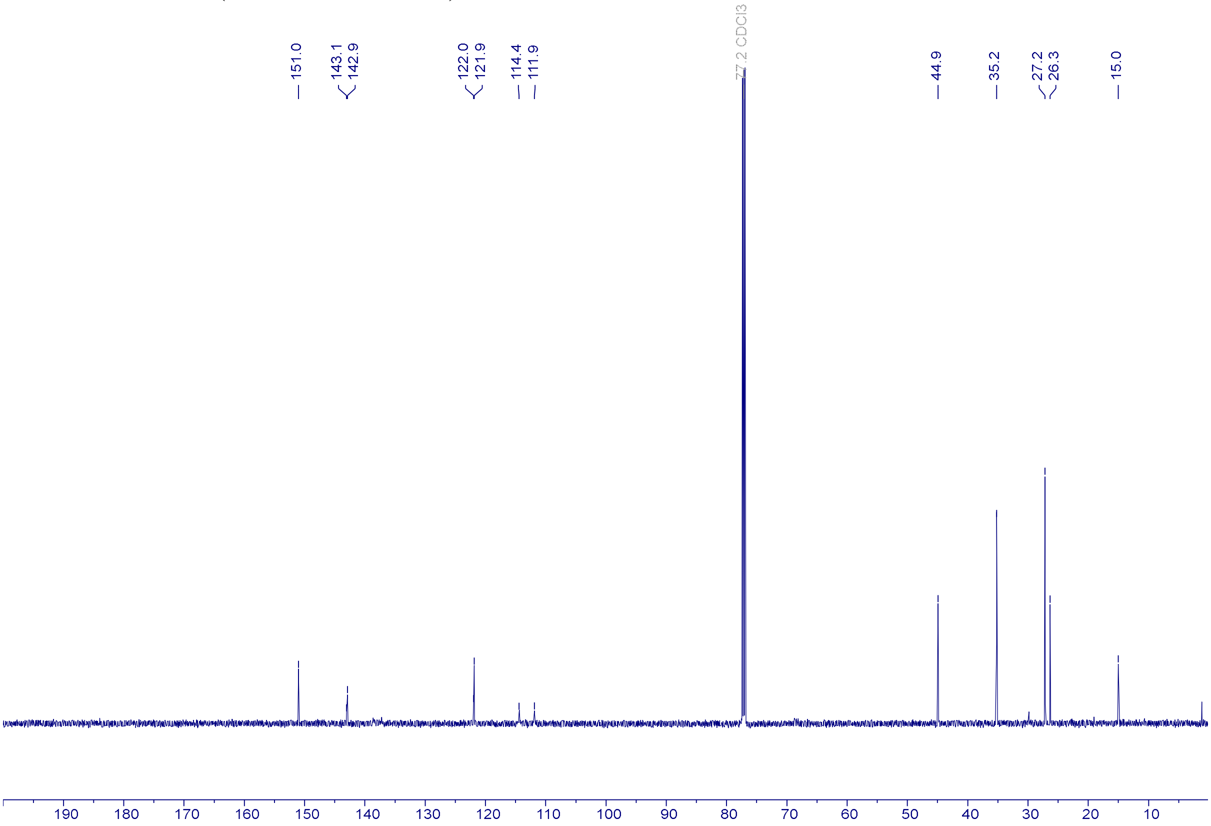

44a – ^{1}H NMR (600 MHz, $CDCl_3$)

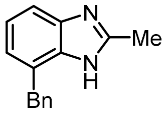

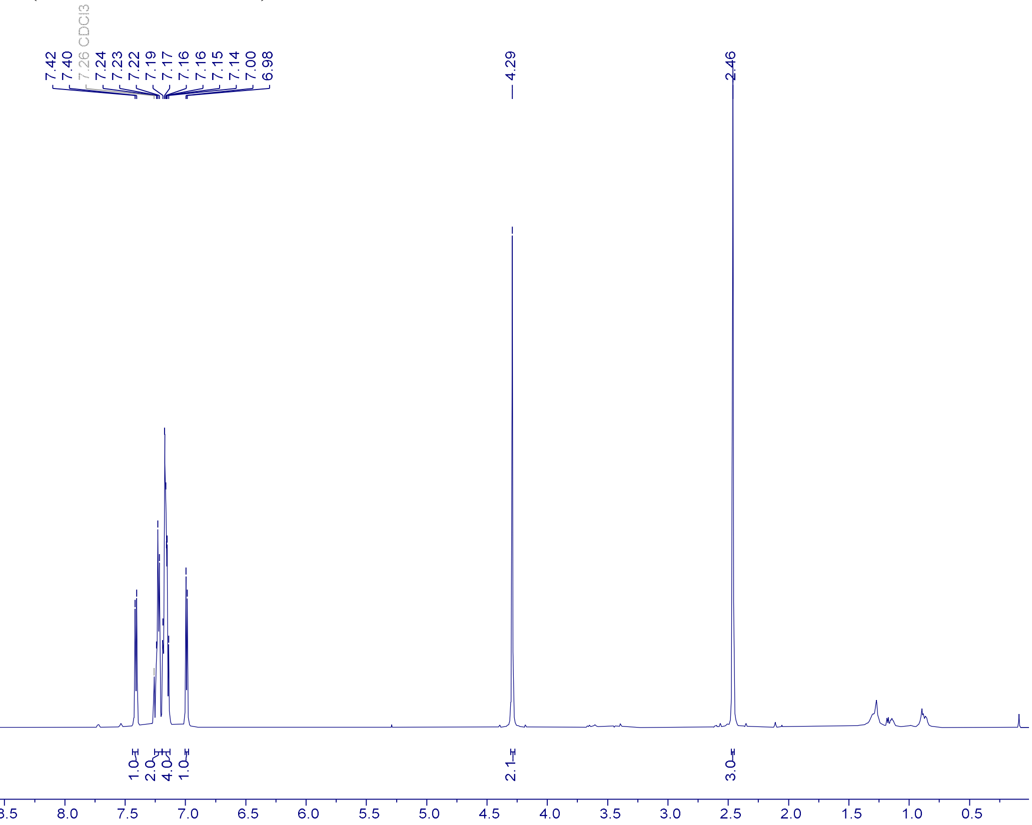

44a – ^{13}C NMR (151 MHz, $CDCl_3$)

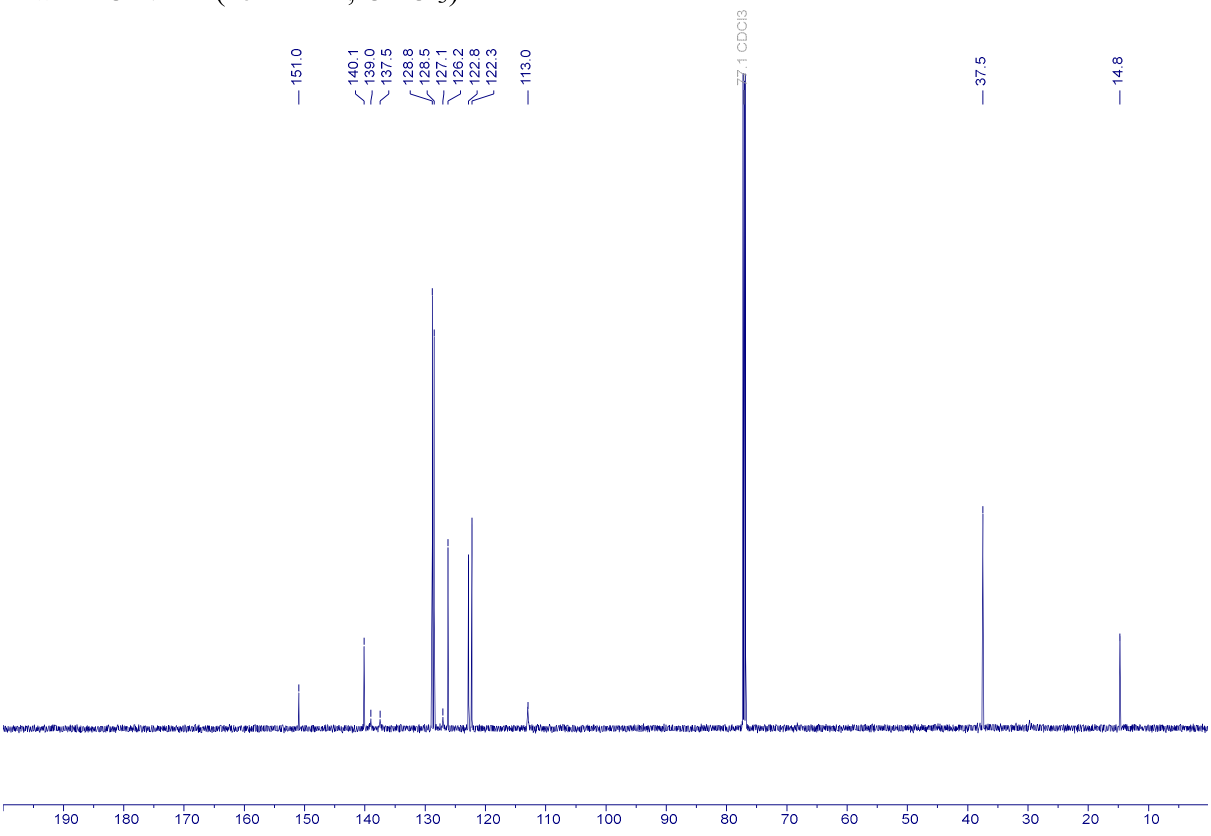

45a – ^{1}H NMR (600 MHz, DMSO-d_6)

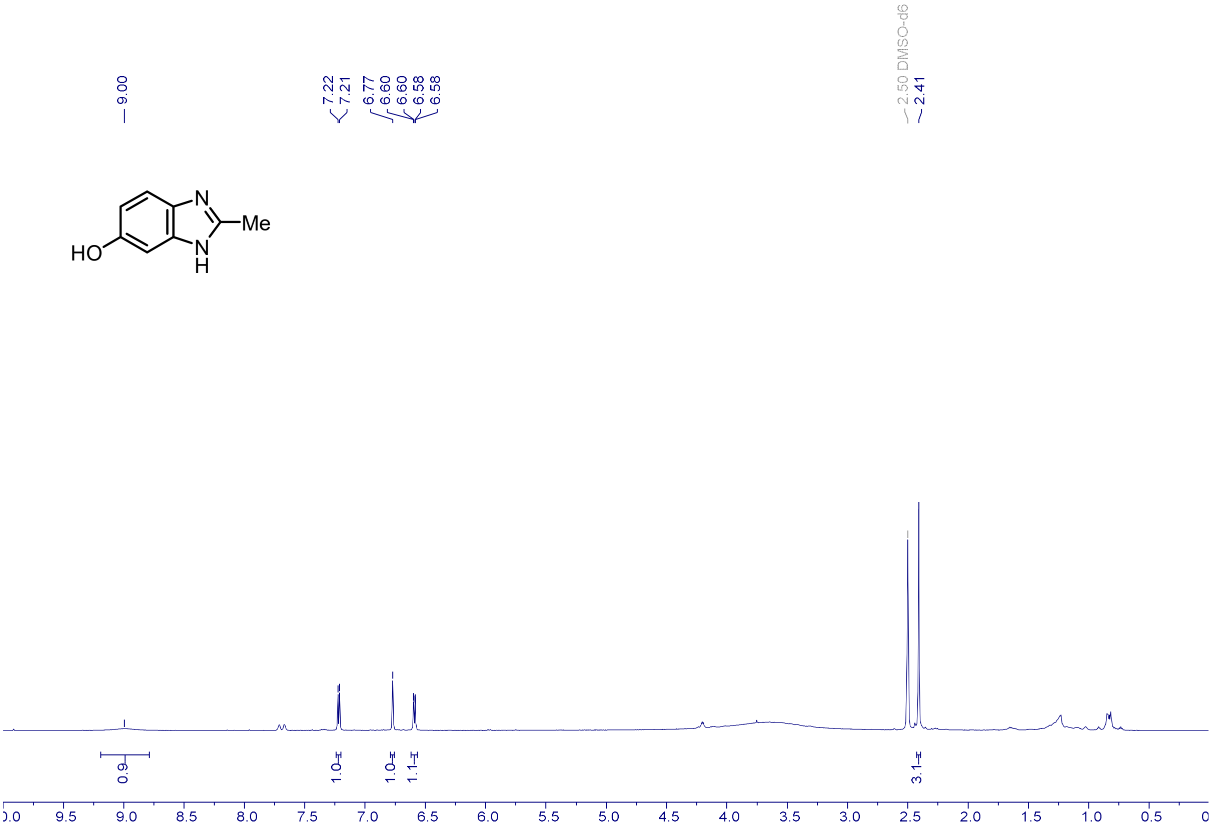

45a – ^{13}C NMR (151 MHz, DMSO-d_6)

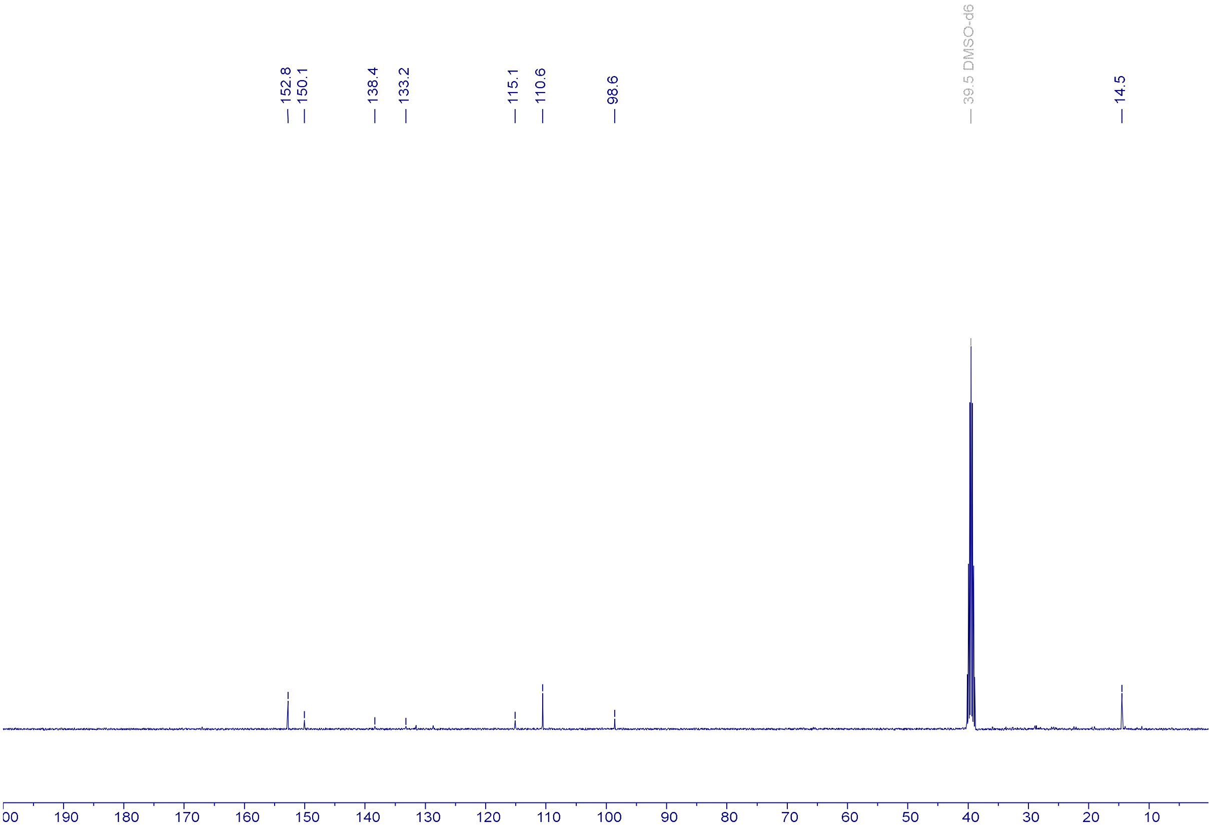

46a – ^{1}H NMR (600 MHz, CDCl$_3$)

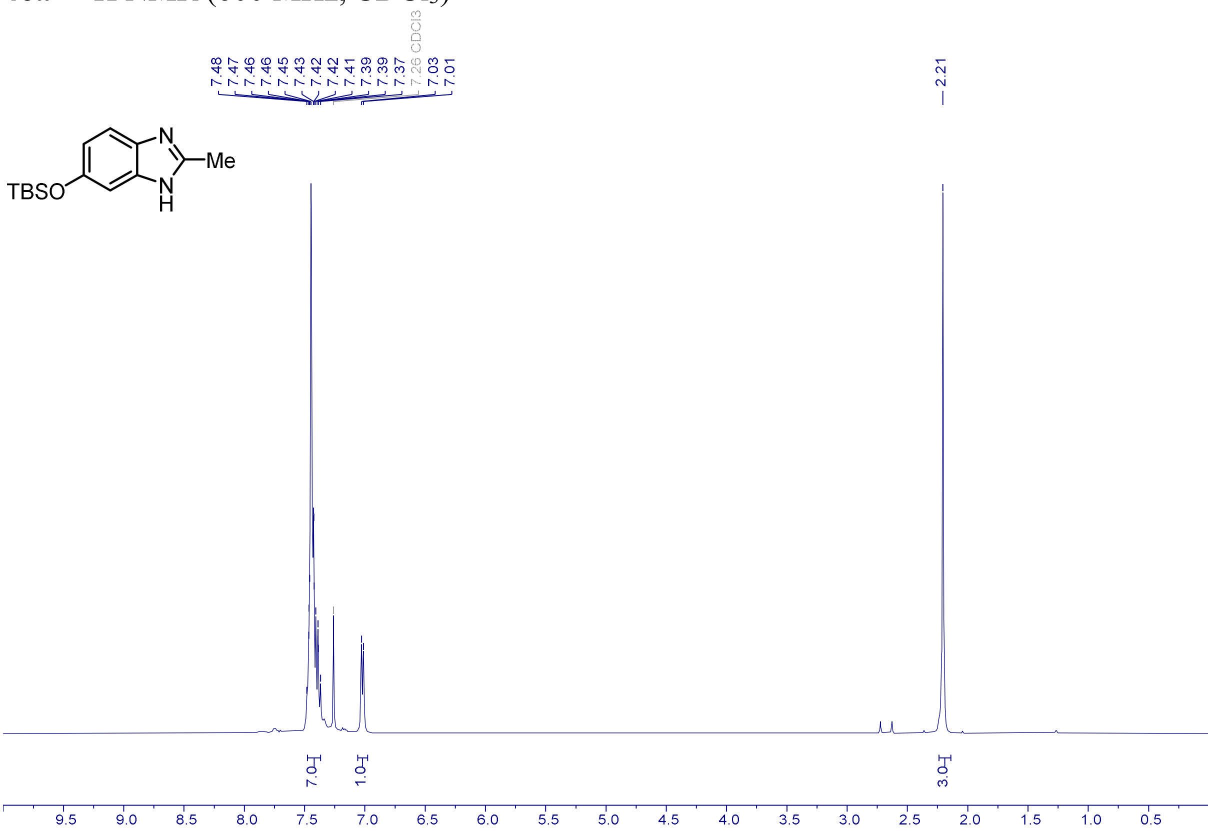

46a – ^{13}C NMR (151 MHz, CDCl$_3$)

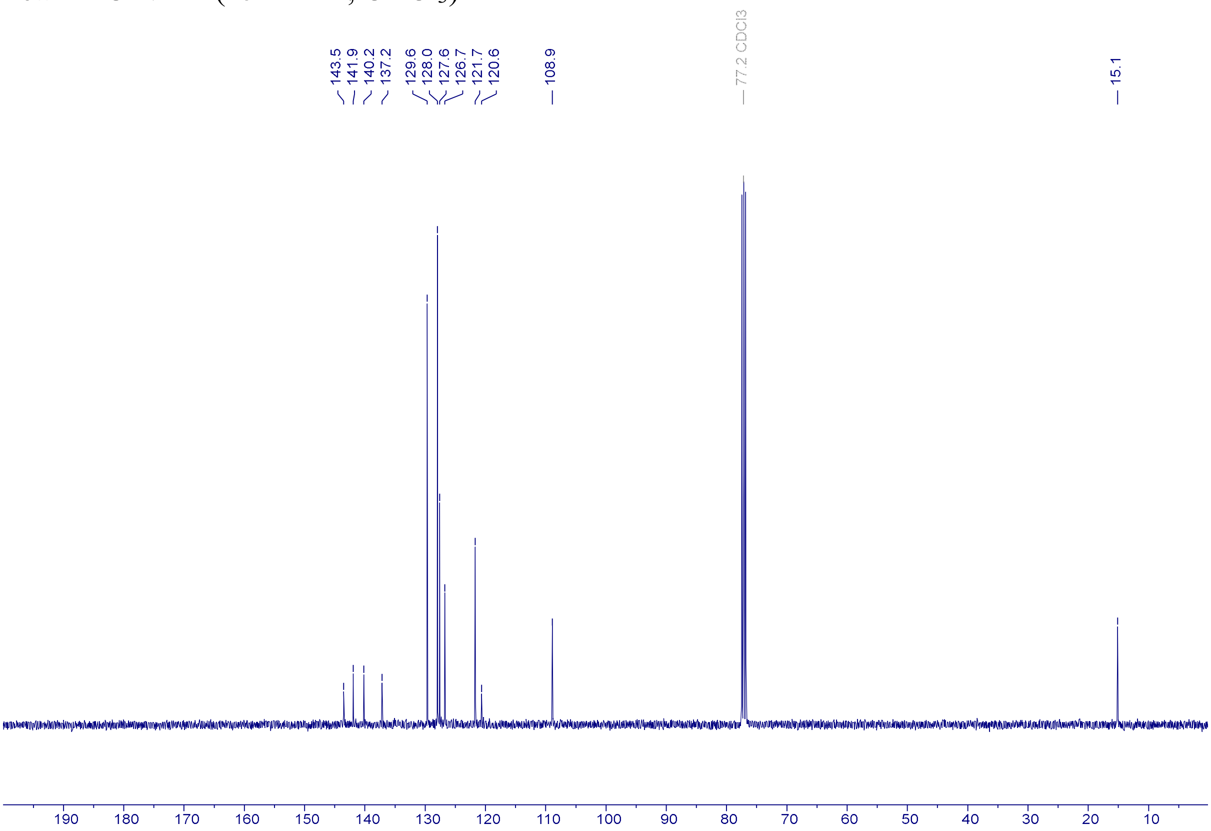

47a – ^{1}H NMR ($CDCl_3$, 600 MHz)

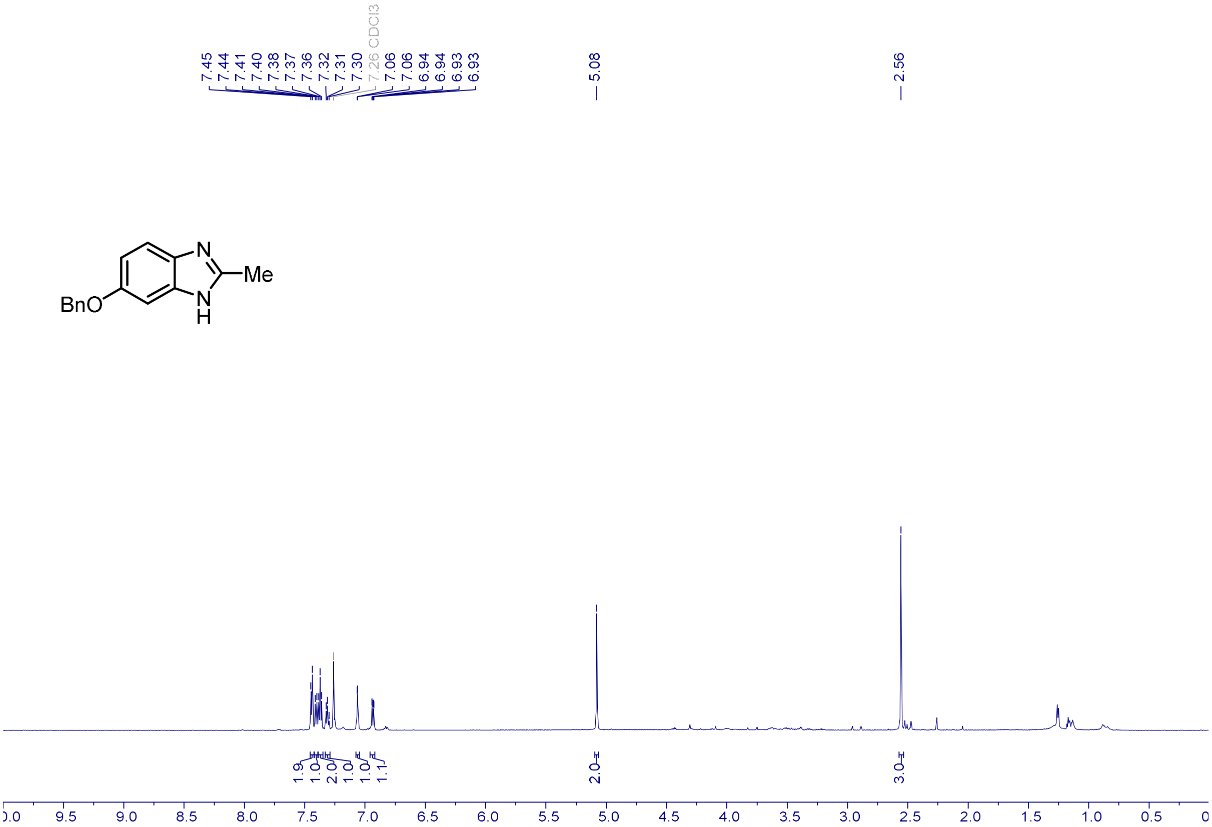

47a – ^{1}H NMR ($CDCl_3$, 151 MHz)

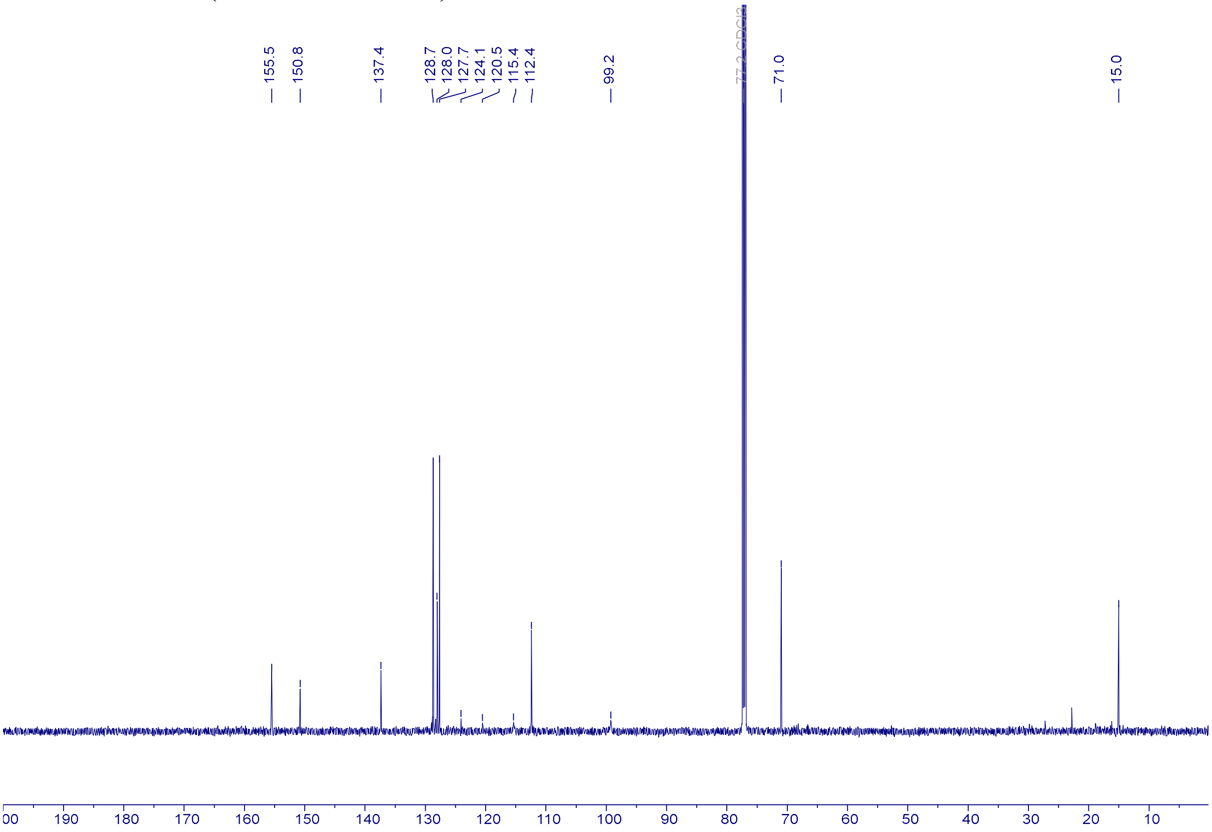

48a – ^{1}H NMR (400 MHz, $CDCl_3$)

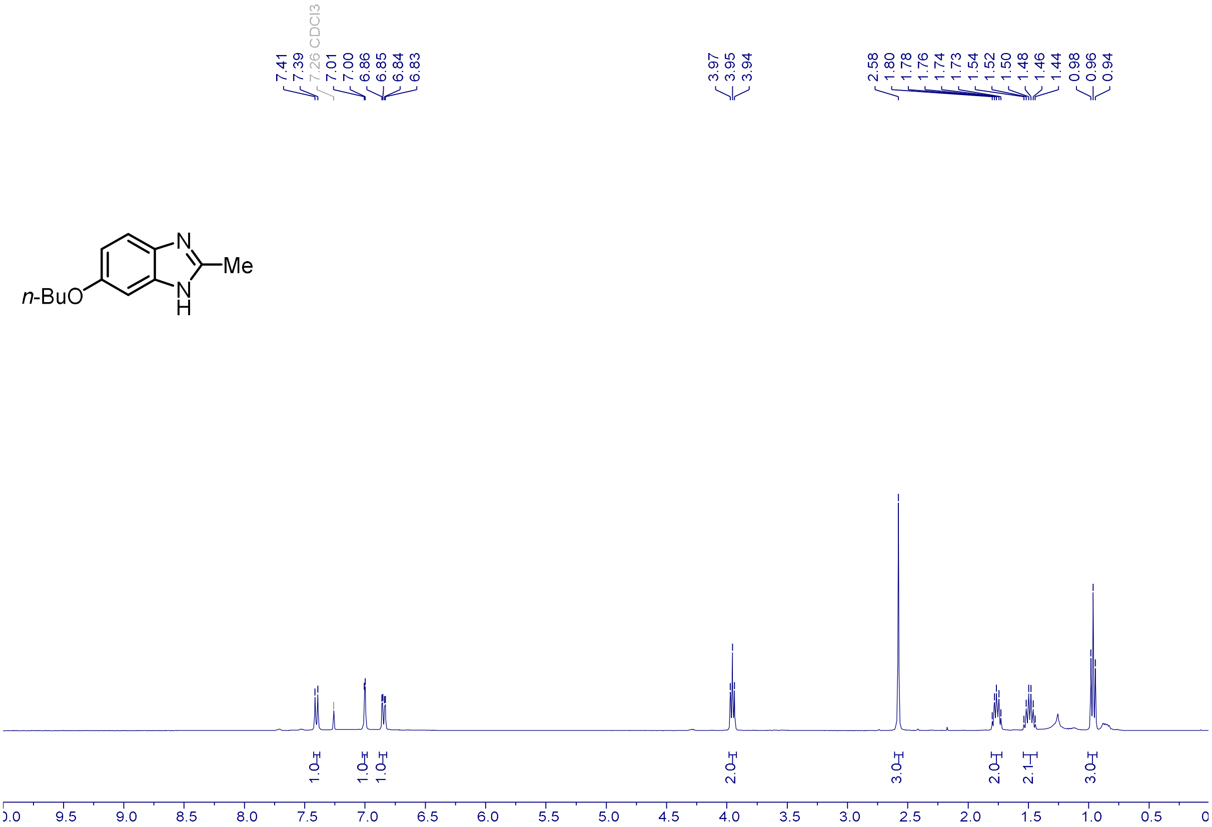

48a – ^{13}C NMR ($CDCl_3$, 101 MHz)

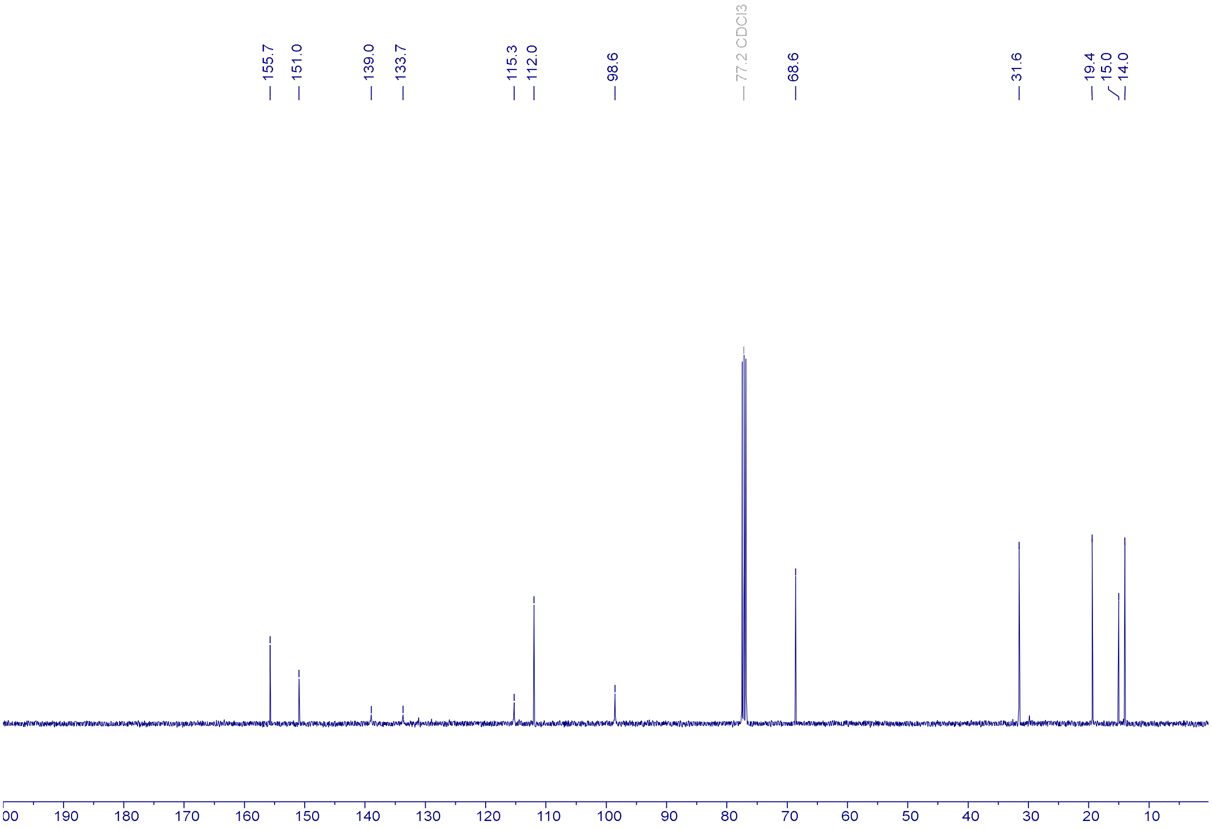

49a – ^{1}H NMR ($CDCl_3$, 400 MHz)

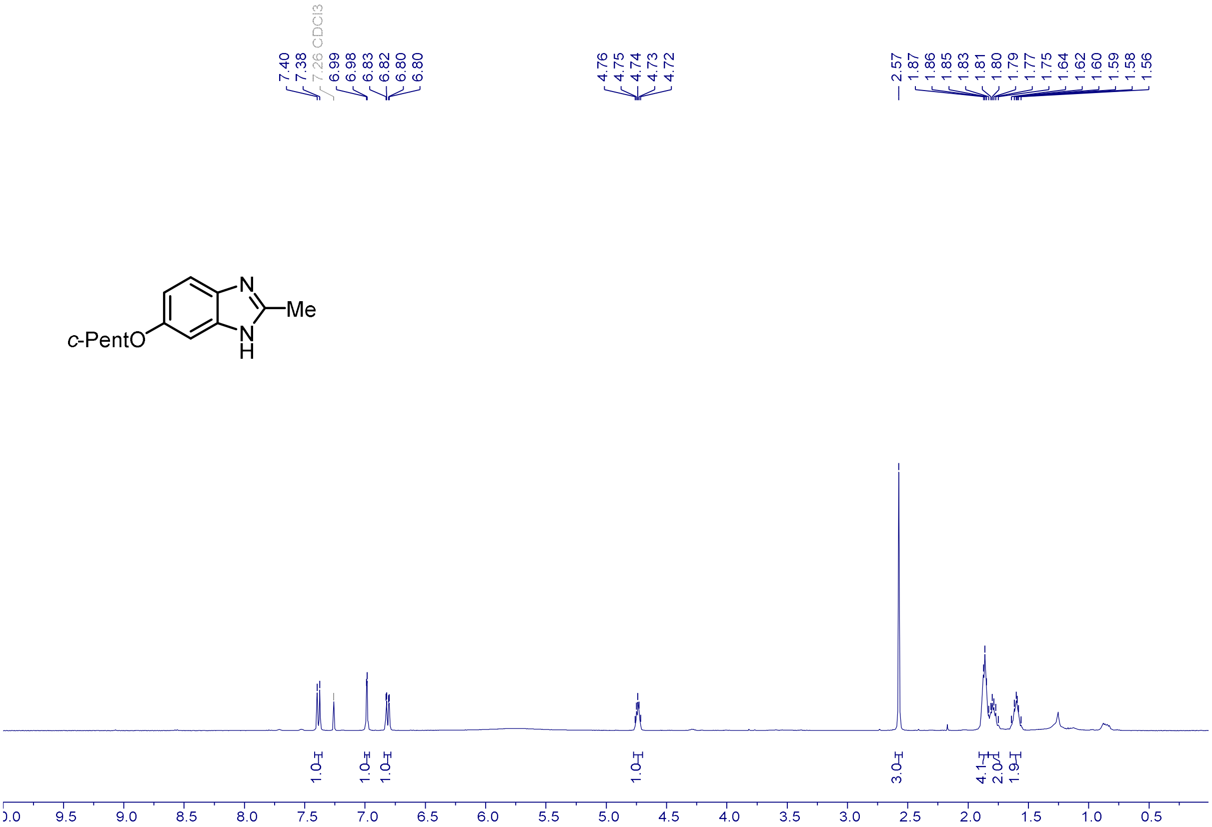

49a – ^{13}C NMR ($CDCl_3$, 101 MHz)

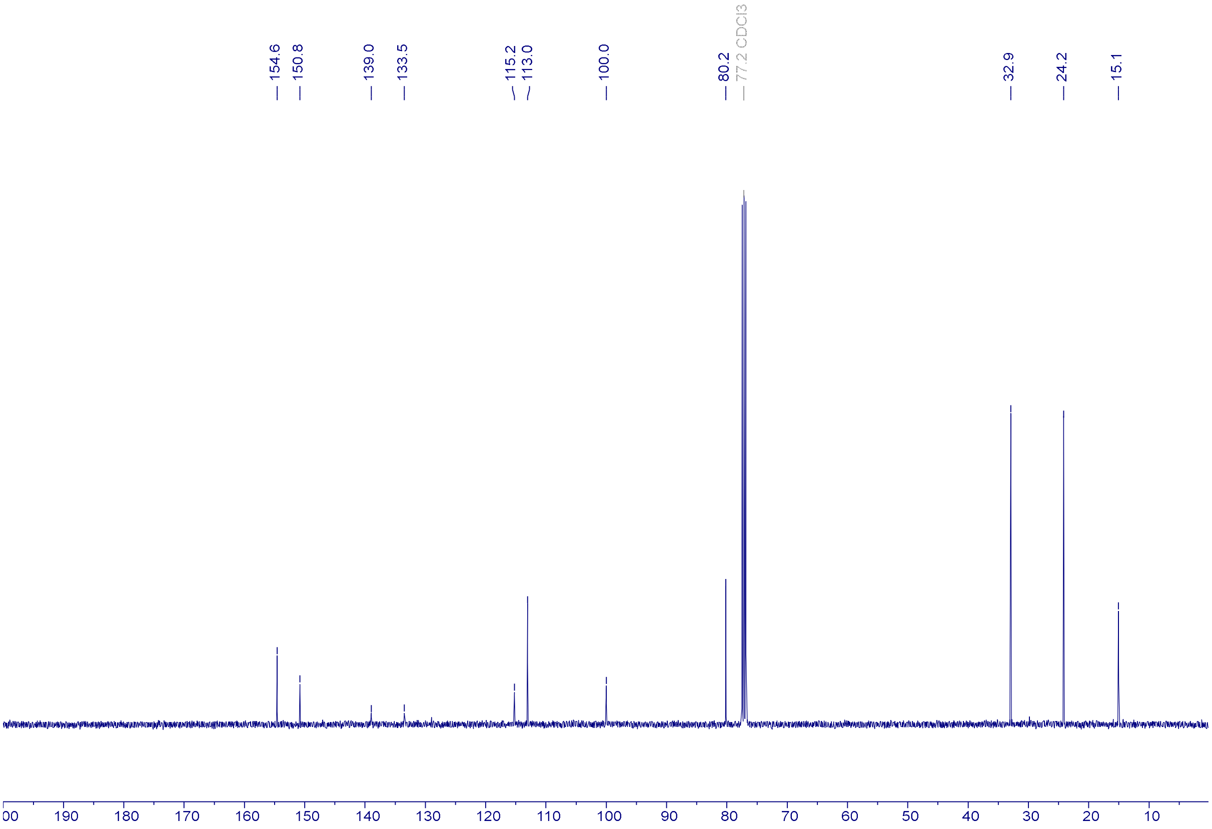

60a – ^{1}H NMR (400 MHz, $CDCl_3$)

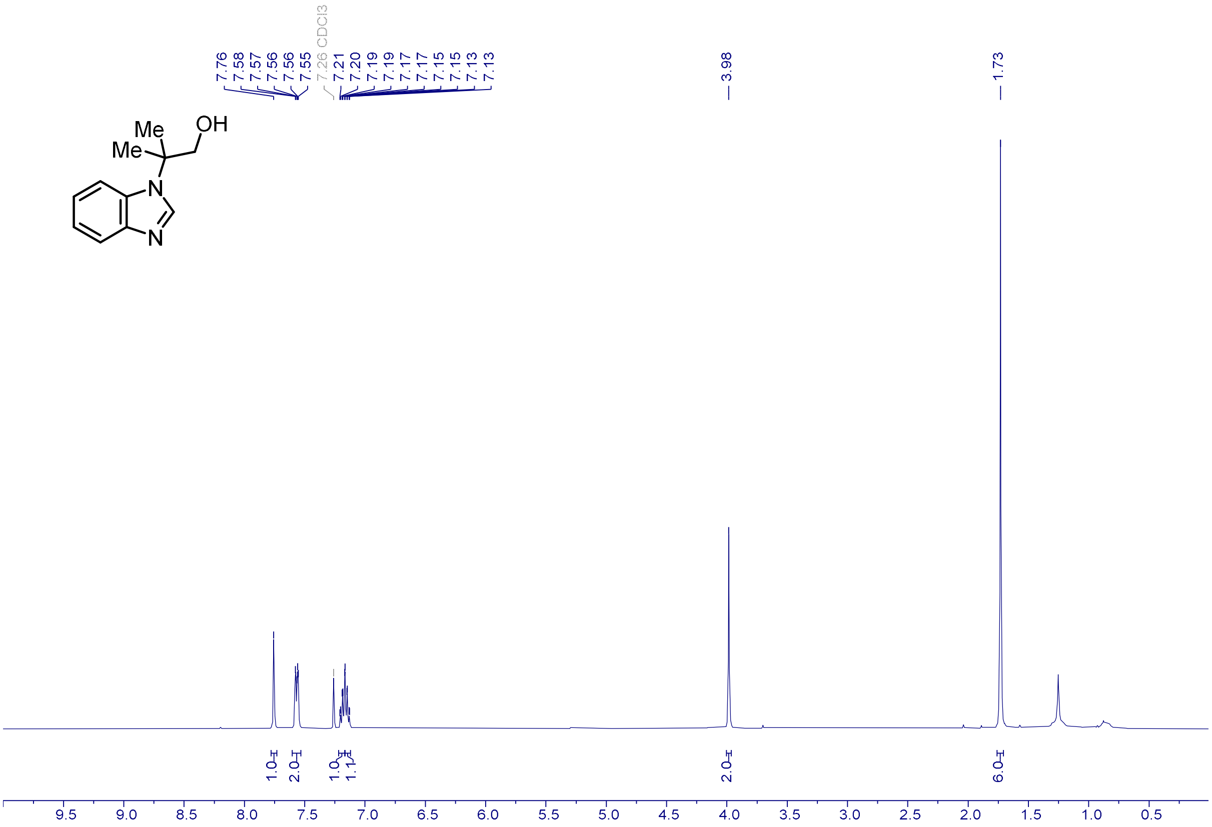

60a – ^{13}C NMR (101 MHz, $CDCl_3$)

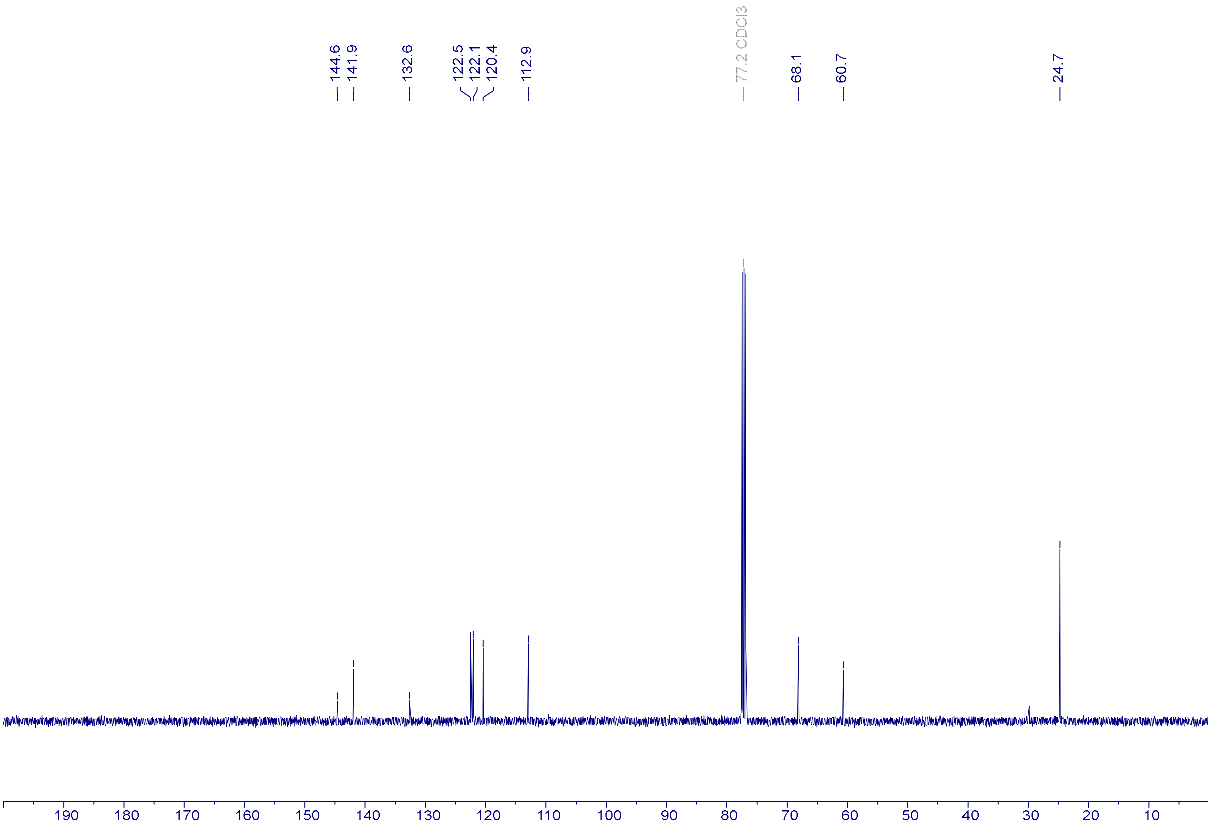

62a – ^{1}H NMR (400 MHz, $CDCl_3$)

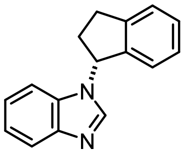

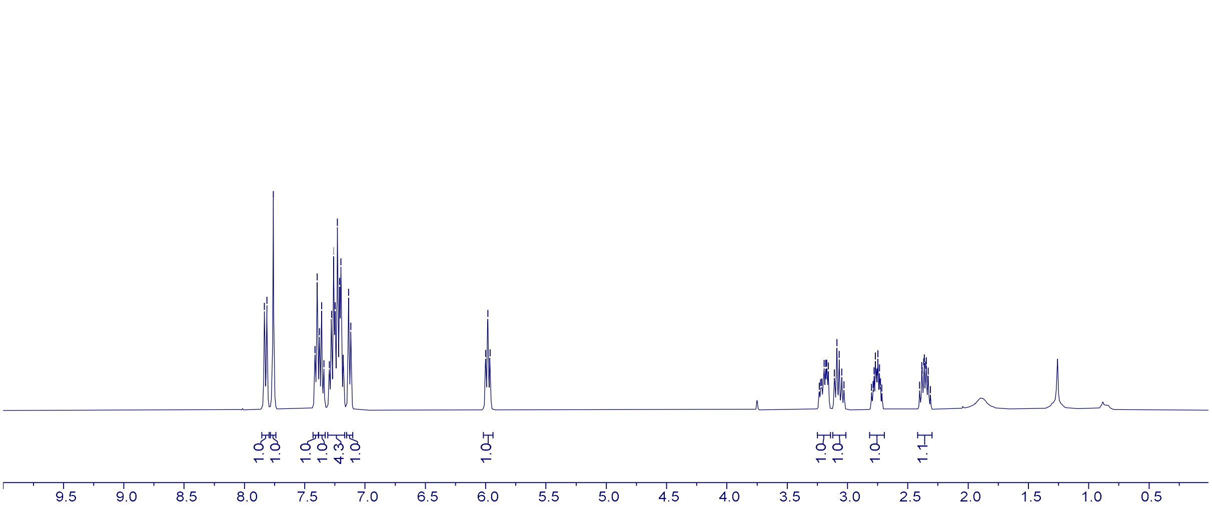

62a – ^{13}C NMR (101 MHz, $CDCl_3$)

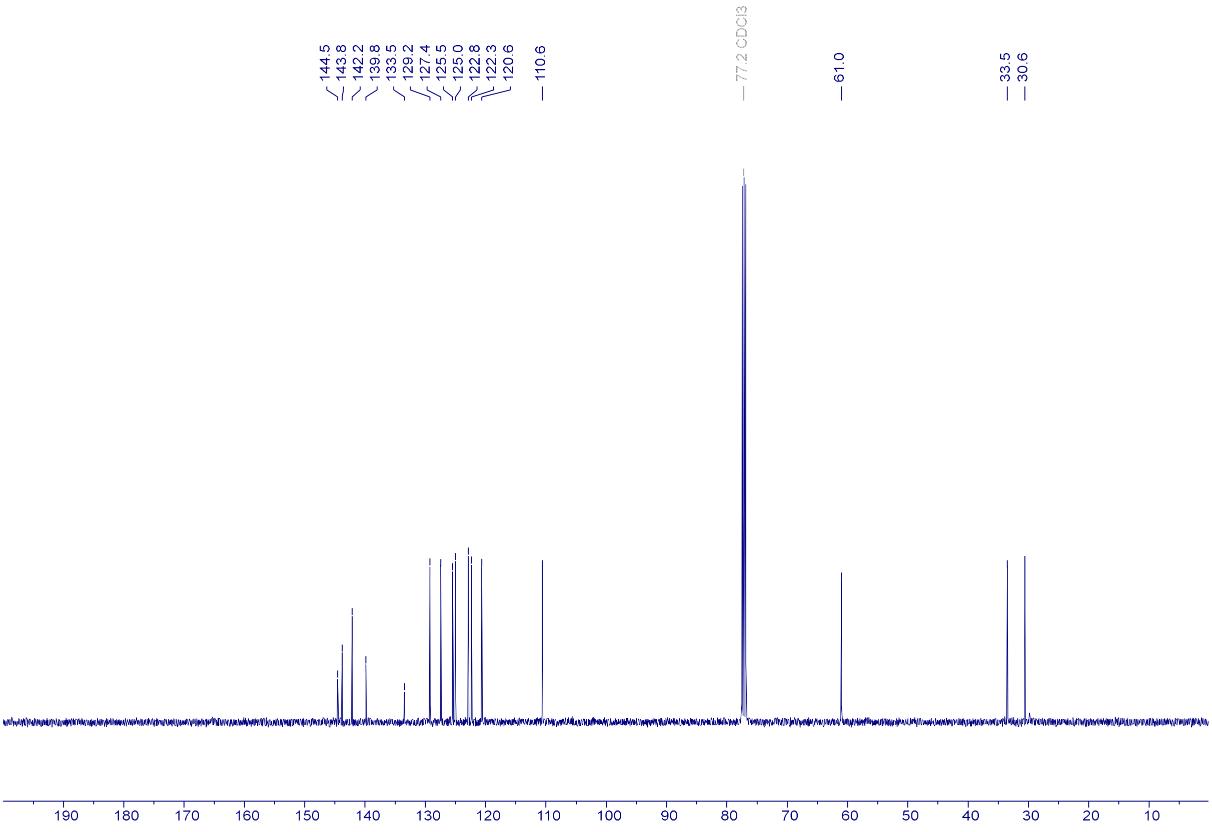

63a– ^{1}H NMR (600 MHz, $CDCl_3$)

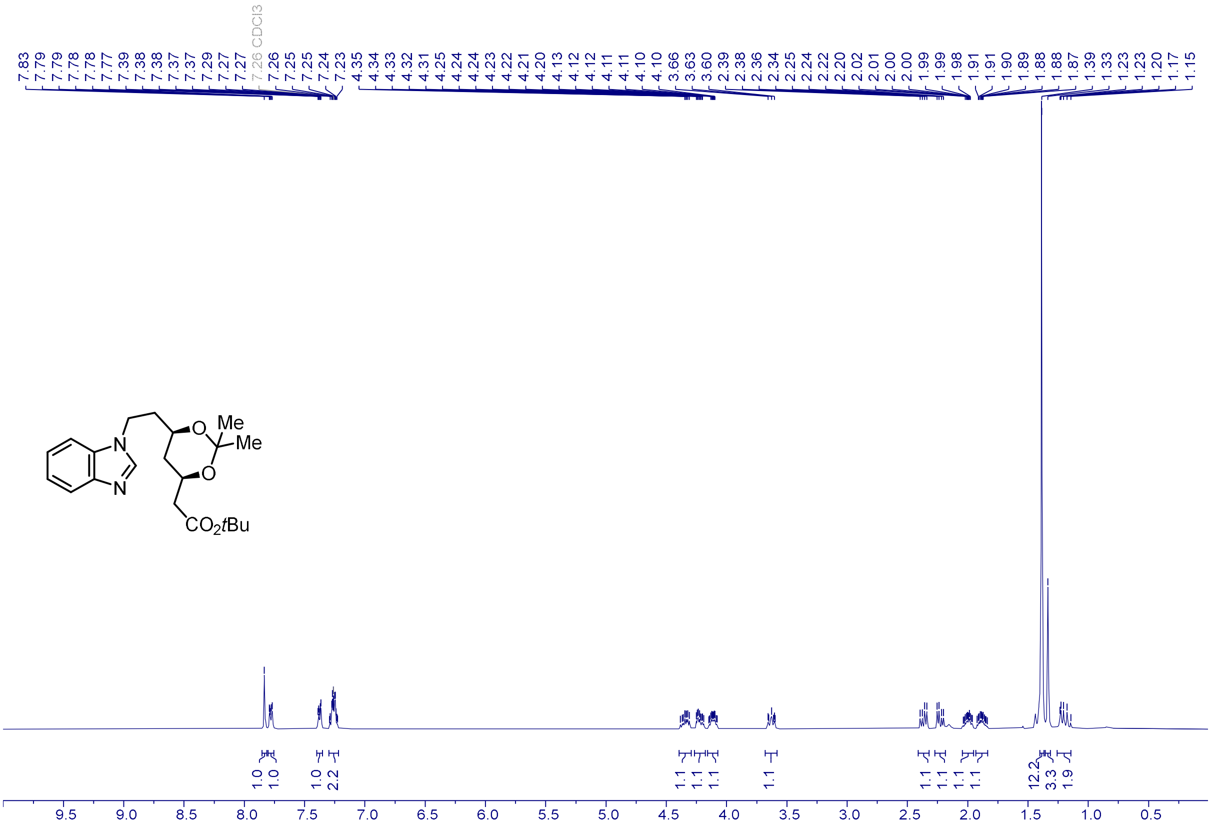

63a – ^{13}C NMR (151 MHz, $CDCl_3$)

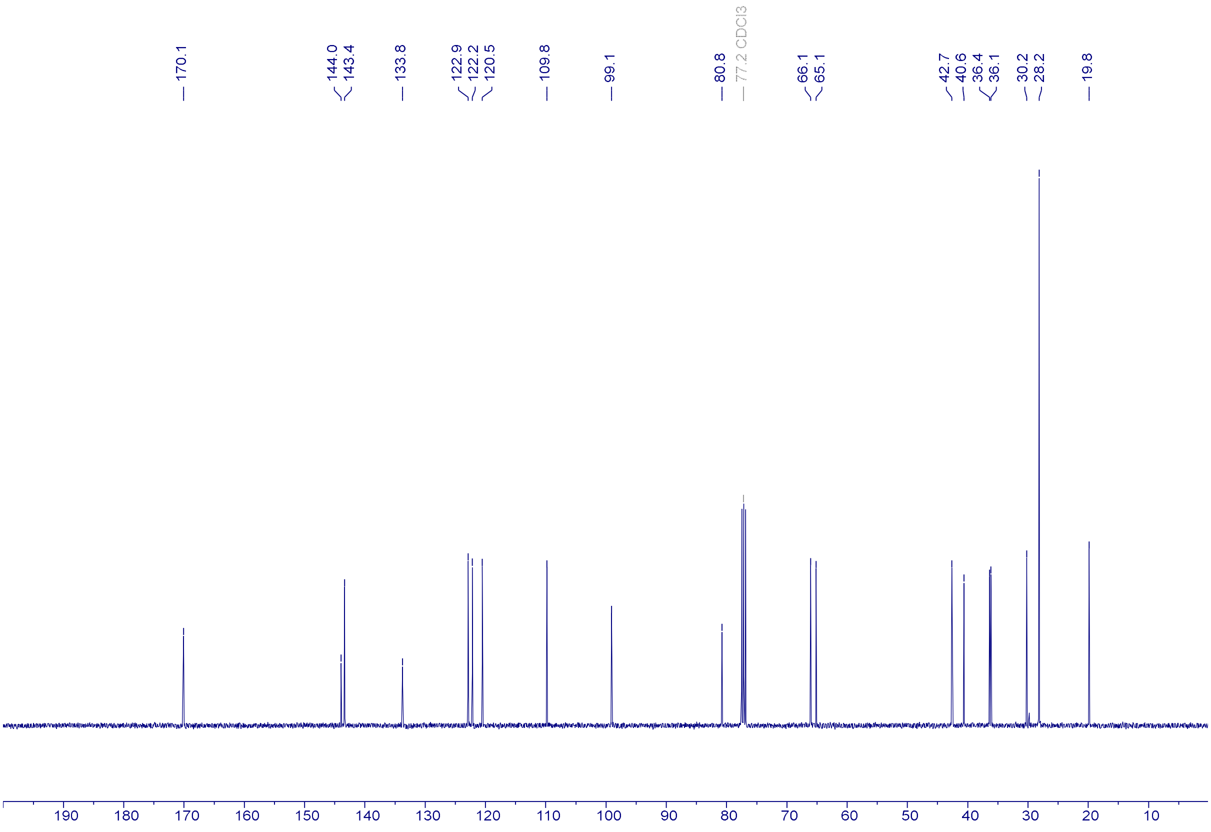

14.References

[1] Y. Tang, Y. Zheng, X. Hu, H. Zhao, S. Cui, *J. Med. Chem.* **2024**, *67*, 6638-6657.

[2] S. B. Lee, S. Y. Paek, S. K. Yoon, S. H. Yoon, J. S. Choi, (Ed.: L. L. S. LTD.), **2013**, pp. 1-246.

[3] N. Ueberschaar, Z. Xu, K. Scherlach, M. Metsä-Ketelä, T. Bretschneider, H.-M. Dahse, H. Görls, C. Hertweck, *J. Am. Chem. Soc.* **2013**, *135*, 17408–17416.

[4] J. Wang, D. Shi, Z. Wang, F. Ren, X. Li, Y. e. You, X. Liu, Y. Lou, *J. Org. Chem.* **2023**, *88*, 13049–13056.

[5] C. Shu, K. Sidhu, L. Zhang, X.-j. Wang, D. Krishnamurthy, C. H. Senanayake, *J. Org. Chem.* **2010**, *75*, 6677–6680.

[6] M. Cheung, A. Boloor, J. A. Stafford, *J. Org. Chem.* **2003**, *68*, 4093–4095.

[7] N. E. Genung, L. Wei, G. E. Aspnes, *Org. Lett.* **2014**, *16*, 3114–3117.

[8] Y.-Z. Qiu, Z. Zhang, Z.-Q. Zhong, H.-W. Hu, *Chin. J. Chem.* **1992**, *10*, 556–560.

[9] P. J. S. Y. K. A. J. L. Yeong, (Ed.: WPO), **2006**.

[10] J. Wang, D. Shi, Z. Wang, F. Ren, X. Li, Y. e. You, X. Liu, Y. Lou, *J. Org. Chem.* **2023**, *88*, 13049-13056.

[11] A. Chevalier, A. Ouahrouch, A. Arnaud, T. Gallavardin, X. Franck, *RSC Adv.* **2018**, *8*, 13121-13128.

[12] M. H. Palmer, R. H. Findlay, S. M. F. Kennedy, P. S. McIntyre, *J. Chem. Soc., Perkin Trans. 2* **1975**, 1695-1700.

[13] P. Li, C. Wu, J. Zhao, D. C. Rogness, F. Shi, *J. Org. Chem.* **2012**, *77*, 3149–3158.

[14] A. J. Liedtke, K. Kim, D. F. Stec, G. A. Sulikowski, L. J. Marnett, *Tetrahedron* **2012**, *68*, 10049–10058.

[15] A. Vázquez, E. Bonilla-Reyes, A. Sánchez-Carrillo, *Synthesis* **2020**, *52*, 3473-3479.

[16] J. L. Wang, D. M. Shi, Z. H. Wang, F. C. Ren, X. Li, Y. E. You, X. H. Liu, Y. Z. Lou, *J. Org. Chem.* **2023**, *88*, 13049-13056.

[17] A. T. Londregan, J. Clemens, E. L. Bell, *Synthesis* **2022**, *54*, 3215-3226.

[18] A. Bunnell, C. O'Yang, A. Petrica, M. J. Soth, *Synth. Commun.* **2006**, *36*, 285-293.

[19] A. Schoeggl Toledano, J. Bitai, D. Covini, J. Karolyi-Oezguer, C. Dank, H. Berger, A. Gollner, *Org. Lett.* **2024**, *26*, 1229–1232.

[20] J. S. Zhu, C. J. Li, K. Y. Tsui, N. Kraemer, J. H. Son, M. J. Haddadin, D. J. Tantillo, M. J. Kurth, *J. Am. Chem. Soc.* **2019**, *141*, 6247-6253.

[21] N. E. Genung, L. Wei, G. E. Aspnes, *Org. Lett.* **2014**, *16*, 3114-3117.

[22] B. Yu, H. Zhang, Y. Zhao, S. Chen, J. Xu, C. Huang, Z. Liu, *Green Chem.* **2013**, *15*, 95-99.

[23] J.-y. Kato, Y. Ito, R. Ijuin, H. Aoyama, T. Yokomatsu, *Org. Lett.* **2013**, *15*, 3794-3797.

[24] E. M. Bonku, H. Qin, A. Odilov, S. Abduahadi, S. D. Guma, F. Yang, F. Zhu, H. A. Aisa, J. Shen, *RSC Adv* **2024**, *14*, 6906-6916.

[25] M. A. Larsen, J. F. Hartwig, *J. Am. Chem. Soc.* **2014**, *136*, 4287–4299.

[26] S. Kumari, A. Joshi, I. Borthakur, S. Kundu, *J. Org. Chem.* **2023**, *88*, 11523–11533.

[27] J. Kim, J. Kim, H. Lee, B. M. Lee, B. H. Kim, *Tetrahedron* **2011**, *67*, 8027–8033.

[28] S. C. Fosu, C. M. Hambira, A. D. Chen, J. R. Fuchs, D. A. Nagib, *Chem* **2019**, *5*, 417-428.

[29] M. S. Mayo, X. Yu, X. Zhou, X. Feng, Y. Yamamoto, M. Bao, *Org. Lett.* **2014**, *16*, 764–767.

[30] B. B. Shaik, S. B. Mohite, S. Partap, V. Kumar, S. Vangara, M. D. Bala, P. Singh, R. Karpoormath, *Tetrahedron* **2024**, *154*.

[31] R. Chebolu, D. N. Kommi, D. Kumar, N. Bollineni, A. K. Chakraborti, *J. Org. Chem.* **2012**, *77*, 10158–10167.

[32] M. Kljajic, J. G. Puschnig, H. Weber, R. Breinbauer, *Org. Lett.* **2017**, *19*, 126–129.

[33] Y. Chen, M. Leonardi, P. Dingwall, R. Labes, P. Pasau, D. C. Blakemore, S. V. Ley, *J. Org. Chem.* **2018**, *83*, 15558–15568.

[34] L. L. R. Lorentz-Petersen, L. U. Nordstrøm, R. Madsen, *Eur. J. Org. Chem.* **2012**, *2012*, 6752-6759.

[35] X. Diao, Y. Wang, Y. Jiang, D. Ma, *J. Org. Chem.* **2009**, *74*, 7974–7977.
[36] S. Perrone, M. Capua, G. Cannazza, A. Salomone, L. Troisi, *Tetrahedron Lett.* **2016**, *57*, 1421–1424.
[37] C. M. White, S. Cazares, E. D. Gonzalez-Cortes, T. G. Driver, *J Org Chem* **2024**, *89*, 6590-6601.
[38] N. J. Taylor, E. Emer, S. Preshlock, M. Schedler, M. Tredwell, S. Verhoog, J. Mercier, C. Genicot, V. Gouverneur, *J. Am. Chem. Soc.* **2017**, *139*, 8267-8276.
[39] J. E. Gillespie, C. Morrill, R. J. Phipps, *J. Am. Chem. Soc.* **2021**, *143*, 9355-9360.
[40] Y. Dong, B. Breit, *Org Lett* **2021**, *23*, 6765-6769.
[41] Y. Dong, B. Breit, *Org. Lett.* **2021**, *23*, 6765–6769.
[42] Q. Xue, J. Xie, H. Li, Y. Cheng, C. Zhu, *Chem. Commun.* **2013**, *49*, 3700-3702.
[43] L. Wang, M. Shi, X. Chen, N. Su, W. Luo, X. Zhang, *Angew. Chem., Int. Ed.* **2023**, *62*, e202314312.
[44] F. M. Rivas, A. J. Giessert, S. T. Diver, *J. Org. Chem.* **2002**, *67*, 1708-1711.
[45] H. Li, Y. Zhang, Z. Yan, Z. Lai, R. Yang, M. Peng, Y. Sun, J. An, *Green Chem.* **2022**, *24*, 748-753.
[46] M. Noda, N. Hirota, M. Sumitani, K. Yoshihara, *J. Phys. Chem.* **1985**, *89*, 399–401.
[47] a) F. Aquilante, J. Autschbach, A. Baiardi, S. Battaglia, V. A. Borin, L. F. Chibotaru, I. Conti, L. De Vico, M. Delcey, I. Fdez Galvan, N. Ferre, L. Freitag, M. Garavelli, X. Gong, S. Knecht, E. D. Larsson, R. Lindh, M. Lundberg, P. A. Malmqvist, A. Nenov, J. Norell, M. Odelius, M. Olivucci, T. B. Pedersen, L. Pedraza-Gonzalez, Q. M. Phung, K. Pierloot, M. Reiher, I. Schapiro, J. Segarra-Marti, F. Segatta, L. Seijo, S. Sen, D. C. Sergentu, C. J. Stein, L. Ungur, M. Vacher, A. Valentini, V. Veryazov, *J. Chem. Phys.* **2020**, *152*, 214117; b) I. Fdez Galvan, M. Vacher, A. Alavi, C. Angeli, F. Aquilante, J. Autschbach, J. J. Bao, S. I. Bokarev, N. A. Bogdanov, R. K. Carlson, L. F. Chibotaru, J. Creutzberg, N. Dattani, M. G. Delcey, S. S. Dong, A. Dreuw, L. Freitag, L. M. Frutos, L. Gagliardi, F. Gendron, A. Giussani, L. Gonzalez, G. Grell, M. Guo, C. E. Hoyer, M. Johansson, S. Keller, S. Knecht, G. Kovacevic, E. Kallman, G. Li Manni, M. Lundberg, Y. Ma, S. Mai, J. P. Malhado, P. A. Malmqvist, P. Marquetand, S. A. Mewes, J. Norell, M. Olivucci, M. Oppel, Q. M. Phung, K. Pierloot, F. Plasser, M. Reiher, A. M. Sand, I. Schapiro, P. Sharma, C. J. Stein, L. K. Sorensen, D. G. Truhlar, M. Ugandi, L. Ungur, A. Valentini, S. Vancoillie, V. Veryazov, O. Weser, T. A. Wesolowski, P. O. Widmark, S. Wouters, A. Zech, J. P. Zobel, R. Lindh, *J Chem Theory Comput* **2019**, *15*, 5925-5964; c) G. Li Manni, I. Fdez Galvan, A. Alavi, F. Aleotti, F. Aquilante, J. Autschbach, D. Avagliano, A. Baiardi, J. J. Bao, S. Battaglia, L. Birnoschi, A. Blanco-Gonzalez, S. I. Bokarev, R. Broer, R. Cacciari, P. B. Calio, R. K. Carlson, R. Carvalho Couto, L. Cerdan, L. F. Chibotaru, N. F. Chilton, J. R. Church, I. Conti, S. Coriani, J. Cuellar-Zuquin, R. E. Daoud, N. Dattani, P. Decleva, C. de Graaf, M. G. Delcey, L. De Vico, W. Dobrautz, S. S. Dong, R. Feng, N. Ferre, M. Filatov Gulak, L. Gagliardi, M. Garavelli, L. Gonzalez, Y. Guan, M. Guo, M. R. Hennefarth, M. R. Hermes, C. E. Hoyer, M. Huix-Rotllant, V. K. Jaiswal, A. Kaiser, D. S. Kaliakin, M. Khamesian, D. S. King, V. Kochetov, M. Krosnicki, A. A. Kumaar, E. D. Larsson, S. Lehtola, M. B. Lepetit, H. Lischka, P. Lopez Rios, M. Lundberg, D. Ma, S. Mai, P. Marquetand, I. C. D. Merritt, F. Montorsi, M. Morchen, A. Nenov, V. H. A. Nguyen, Y. Nishimoto, M. S. Oakley, M. Olivucci, M. Oppel, D. Padula, R. Pandharkar, Q. M. Phung, F. Plasser, G. Raggi, E. Rebolini, M. Reiher, I. Rivalta, D. Roca-Sanjuan, T. Romig, A. A. Safari, A. Sanchez-Mansilla, A. M. Sand, I. Schapiro, T. R. Scott, J. Segarra-Marti, F. Segatta, D. C. Sergentu, P. Sharma, R. Shepard, Y. Shu, J. K. Staab, T. P. Straatsma, L. K. Sorensen, B. N. C. Tenorio, D. G. Truhlar, L. Ungur, M. Vacher, V. Veryazov, et al., *J Chem Theory Comput* **2023**, *19*, 6933-6991.
[48] a) T. H. Dunning, *J. Chem. Phys.* **1989**, *90*, 1007-1023; b) R. A. Kendall, T. H. Dunning, R. J. Harrison, *J. Chem. Phys.* **1992**, *96*, 6796-6806; c) K. A. Peterson, D. E. Woon, T. H. Dunning, *J. Chem. Phys.* **1994**, *100*, 7410-7415; d) F. Weigend, R. Ahlrichs, *Phys. Chem. Chem. Phys.* **2005**, *7*, 3297-3305; e) D. E. Woon, T. H. Dunning, *J. Chem. Phys.* **1993**, *98*, 1358-1371.
[49] T. Yanai, D. P. Tew, N. C. Handy, *Chem. Phys. Lett.* **2004**, *393*, 51-57.

[50] A. V. Marenich, C. J. Cramer, D. G. Truhlar, *The Journal of Physical Chemistry B* **2009**, *113*, 6378-6396.
[51] M. J. Frisch, G. W. Trucks, H. B. Schlegel, G. E. Scuseria, M. A. Robb, J. R. Cheeseman, G. Scalmani, V. Barone, G. A. Petersson, H. Nakatsuji, X. Li, M. Caricato, A. V. Marenich, J. Bloino, B. G. Janesko, R. Gomperts, B. Mennucci, H. P. Hratchian, J. V. Ortiz, A. F. Izmaylov, J. L. Sonnenberg, Williams, F. Ding, F. Lipparini, F. Egidi, J. Goings, B. Peng, A. Petrone, T. Henderson, D. Ranasinghe, V. G. Zakrzewski, J. Gao, N. Rega, G. Zheng, W. Liang, M. Hada, M. Ehara, K. Toyota, R. Fukuda, J. Hasegawa, M. Ishida, T. Nakajima, Y. Honda, O. Kitao, H. Nakai, T. Vreven, K. Throssell, J. A. Montgomery Jr., J. E. Peralta, F. Ogliaro, M. J. Bearpark, J. J. Heyd, E. N. Brothers, K. N. Kudin, V. N. Staroverov, T. A. Keith, R. Kobayashi, J. Normand, K. Raghavachari, A. P. Rendell, J. C. Burant, S. S. Iyengar, J. Tomasi, M. Cossi, J. M. Millam, M. Klene, C. Adamo, R. Cammi, J. W. Ochterski, R. L. Martin, K. Morokuma, O. Farkas, J. B. Foresman, D. J. Fox, Wallingford, CT, **2016**.
[52] J. D. Chai, M. Head-Gordon, *Phys. Chem. Chem. Phys.* **2008**, *10*, 6615-6620.
[53] S. Grimme, *Chemistry* **2012**, *18*, 9955-9964.
[54] G. Luchini, J. V. Alegre-Requena, I. Funes-Ardoiz, R. S. Paton, *F1000Research* **2020**, *9*.
